# Tailored Pyridoxal Probes Unravel Novel Cofactor‐Dependent Targets and Antibiotic Hits in Critical Bacterial Pathogens

**DOI:** 10.1002/anie.202117724

**Published:** 2022-04-12

**Authors:** Martin Pfanzelt, Thomas E. Maher, Ramona M. Absmeier, Markus Schwarz, Stephan A. Sieber

**Affiliations:** ^1^ Center for Functional Protein Assemblies (CPA) Department of Chemistry Chair of Organic Chemistry II Technical University of Munich Ernst-Otto-Fischer-Str. 8 85748 Garching Germany; ^2^ Department of Chemistry Molecular Sciences Research Hub White City Campus and Institute of Chemical Biology Molecular Sciences Research Hub White City Campus Imperial College London 82 Wood Lane London W12 0BZ UK

**Keywords:** Antibiotic Compound Screening, Cofactors, Enzyme Characterisation, Proteomics, Pyridoxal Phosphate-Dependent Enzymes

## Abstract

Unprecedented bacterial targets are urgently needed to overcome the resistance crisis. Herein we systematically mine pyridoxal phosphate‐dependent enzymes (**PLP**‐DEs) in bacteria to focus on a target class which is involved in crucial metabolic processes. For this, we tailored eight pyridoxal (**PL**) probes bearing modifications at various positions. Overall, the probes exceeded the performance of a previous generation and provided a detailed map of **PLP**‐DEs in clinically relevant pathogens including challenging Gram‐negative strains. Putative **PLP**‐DEs with unknown function were exemplarily characterized via in‐depth enzymatic assays. Finally, we screened a panel of **PLP** binders for antibiotic activity and unravelled the targets of hit molecules. Here, an uncharacterized enzyme, essential for bacterial growth, was assigned as **PLP**‐dependent cysteine desulfurase and confirmed to be inhibited by the marketed drug phenelzine. Our approach provides a basis for deciphering novel **PLP**‐DEs as essential antibiotic targets along with corresponding ways to decipher small molecule inhibitors.

## Graphical abstract

Unprecedented targets are needed to fight the bacterial pandemic. A suite of pyridoxal probes that infiltrate the cellular metabolism of critical pathogens and get incorporated in pyridoxal‐phosphate dependent enzymes is applied. Chemoproteomics facilitated the functional characterization of previously unknown members and the identification of essential targets with antibiotic hit molecules.

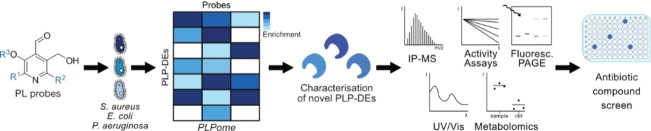

## Introduction

With the rise of antimicrobial resistance (AMR), new strategies to fight bacterial pathogens are desperately needed.[^2^, ^3^, ^4^, ^5^] Most of the currently prescribed antibiotics address a limited number of cellular targets such as cell wall, DNA and protein biosynthesis.[^6^, ^7^] Although initially effective against a broad range of bacteria, their constant use and abuse has led to the development of multiresistant strains, which bear versatile strategies to render these antibiotics ineffective. This problem is especially severe when it comes to the treatment of infections caused by Gram‐negative bacteria, where two cell membranes effectively block the uptake of small molecules.[^3^, ^8^, ^9^] The development of new treatment options against Gram‐negative strains such as *Pseudomonas aeruginosa*, for which no antibiotics have been introduced for decades, is an extremely urgent task and therefore ranked as a critical priority by the WHO.^[10]^ Thus, unprecedented modes‐of‐action beyond the limited scope of current drug targets are desperately needed in order to overcome this daunting crisis.^[4]^ However, drug discovery efforts in this direction are challenged by several obstacles. For example, a large number of bacterial proteins lack a firm functional assignment, which complicates their validation as drug targets. Additionally, the vast number of proteins with important roles for cellular physiology complicates prioritization for those being druggable and most promising for development.^[5]^ Moreover, chemical inhibitors, which have been identified in vitro often exhibit a limited uptake especially in case of Gram‐negative bacteria.^[9]^

**Figure 1:**
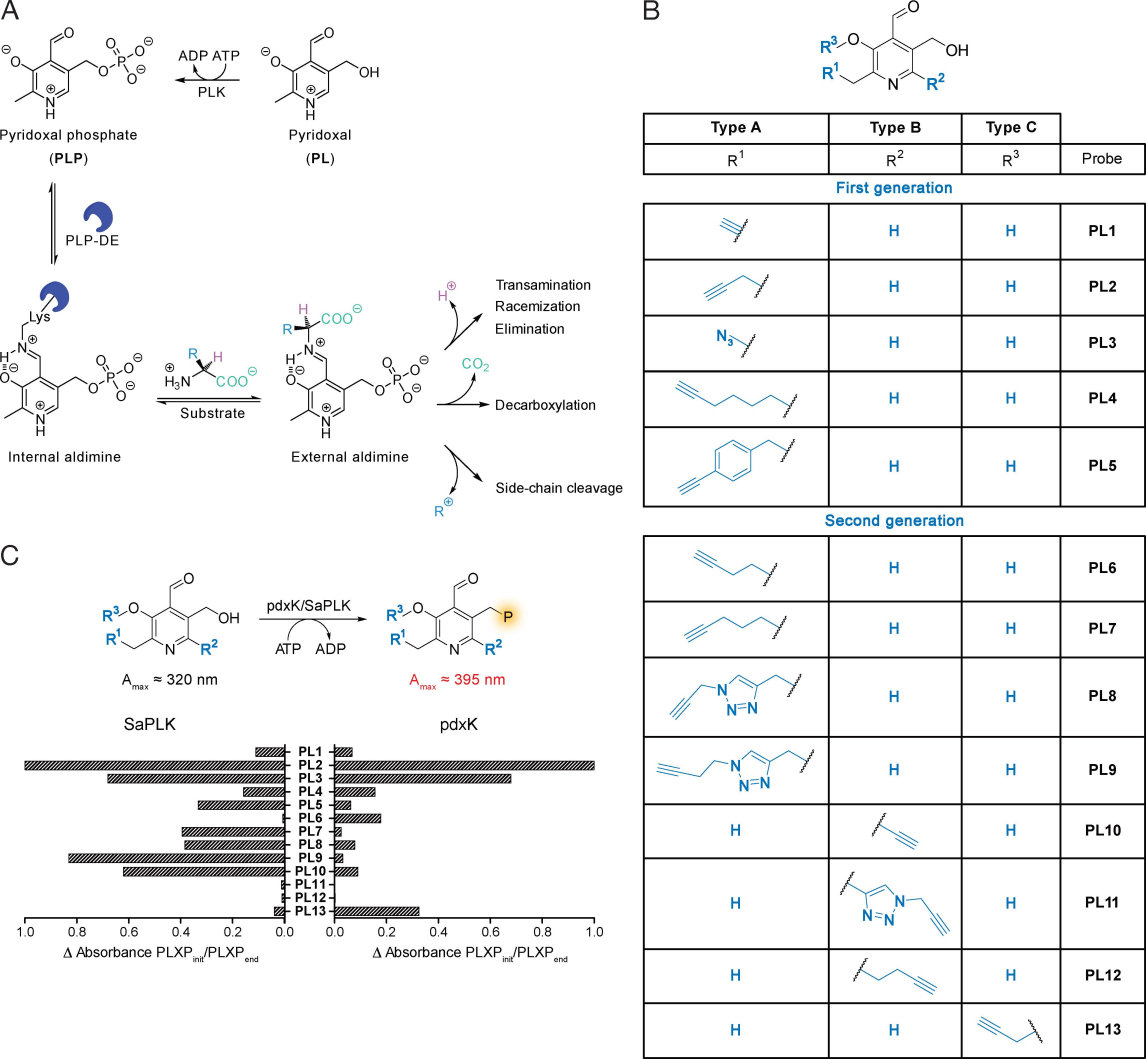
**PL** probe library and activation by pyridoxal kinase. A) Activation of **PL** occurs by phosphorylation with pyridoxal kinase (PLK) to form **PLP**, which can bind **PLP**‐DEs via an internal aldimine to an active site lysine residue. Reaction with substrate amines leads to the formation of external aldimines, which can undergo various reactions via quinonoid structures. B) Structures of first^[13]^ and second generation **PL** probes applied in this study. **Type A** probes are derivatised at the C2′, **type B** probes at the C6 and one **type C** probe is modified at the C3′. C) Activation of **PL** probes by pyridoxal kinases (*E. coli pdxK* or *S. aureus* SaPLK) were monitored by measuring UV/Vis absorbance over time (every 40 min, 20 cycles, *n*=3, mean±SEM). Phosphorylated species absorb at around 395 nm, unphosphorylated around 320 nm. Phosphorylation efficiency is depicted by comparing the absorbance [a.u.] from phosphorylated species (**PLX**, X=1–13) at the beginning (**PLXP_init_**) and at the end (**PLXP_end_**). For **PL6** SaPLK phosphorylation, the difference of initial and final absorbance at wavelengths of the unphosphorylated compound is depicted. For **PL13**, phosphorylated compound absorbed at 320 nm. Raw data of all compounds are shown in Figure SI1, 2.

A novel concept focuses on the systematic mining of cofactor dependent enzymes in bacteria to focus on a target class, which is known to bind ligands (druggability), acquires these ligands from the media (uptake) and is involved in crucial metabolic processes (relevance).[^11^, ^12^] This concept was previously introduced for pyridoxal phosphate (**PLP**), a cofactor which catalyses about 238 enzymatic reactions accounting for 4 % of all classified activities.[^13^, ^14^] **PLP**‐dependent enzymes (**PLP**‐DEs) bind their cofactor via an active site lysine residue to form a Schiff‐base (internal aldimine) which upon substrate binding is displaced to form an external aldimine.^[15]^ Here, the **PLP** cofactor acts as an electron sink facilitating a variety of chemical reactions (Figure 1A).^[16]^ Due to this versatility, **PLP** is crucial for central metabolic processes in prokaryotic and eukaryotic cells including cell wall and neurotransmitter biosynthesis, respectively.[^7^, ^17^] Accordingly, **PLP**‐DEs represent promising therapeutic targets with several drugs on the market.^[18]^ In order to obtain an inventory of **PLP**‐DEs, assign novel functions and mine for promising targets, we recently introduced modified pyridoxal (**PL**) probes, which we functionalized with alkyne or azide moieties at the C2′‐position for application in activity‐based protein profiling (ABPP) (Figure 1B).[^11^, ^13^, ^19^]

The probes were taken up by *Staphylococcus aureus* cells bearing a mutation in their de novo **PLP** biosynthesis (*pdxS* transposon, TnpdxS), phosphorylated to the corresponding **PLP** probes by pyridoxal kinase (PLK) and subsequently incorporated into **PLP**‐DEs to form the internal aldimine. Reductive amination upon cell lysis and copper‐assisted azide‐alkyne click chemistry (CuAAC) or Staudinger ligation (SL) with bio‐orthogonal biotin tags facilitated the detection of about 73 % of all known **PLP**‐DEs in *S. aureus* with high confidence. In addition, several enzymes of previously unknown function could be assigned as putative **PLP**‐DE. While this initial study showed the fidelity of the new methodology, major challenges such as the labelling in Gram‐negative bacteria, labelling in wild type (wt) strains devoid of the de novo mutation, functional assignment of unknown **PLP**‐DEs and discovery of new antibiotic compounds against essential targets remained.

We here designed and synthesized 8 new **PL** probes, which enhanced the overall coverage of **PLP**‐DEs based on their structural diversity. Interestingly, some enzymes could be enriched selectively with certain probes. Together with a refined methodology, these probes not only facilitated labelling in Gram‐negative pathogens but also in wt cells as exemplified for the critical pathogen *P. aeruginosa*. We deciphered the function of several previously unknown **PLP**‐DEs and identified antibiotically active inhibitors along with their cellular targets via competitive profiling with our probes.

## Results and Discussion

### Synthesis of a Chemically Diverse PL Probe Library

Based on a previously published crystal structure of *S. aureus* alanine racemase (*alr*) in complex with **PL1** and **PL2**,^[13]^ we hypothesized that other **PLP**‐DEs could also exhibit extra space in the cofactor binding pockets to accommodate larger substituents and thereby achieve specificity for certain enzyme subclasses. Previous pilot experiments with two probes **PL4** and **PL5** bearing large alkyl or benzyl substituents at the C2′ position of the pyridine ring, revealed that they are able to undergo phosphorylation by *S. aureus* PLK (SaPLK) (Figure SI1).^[13]^

**Scheme 1:**
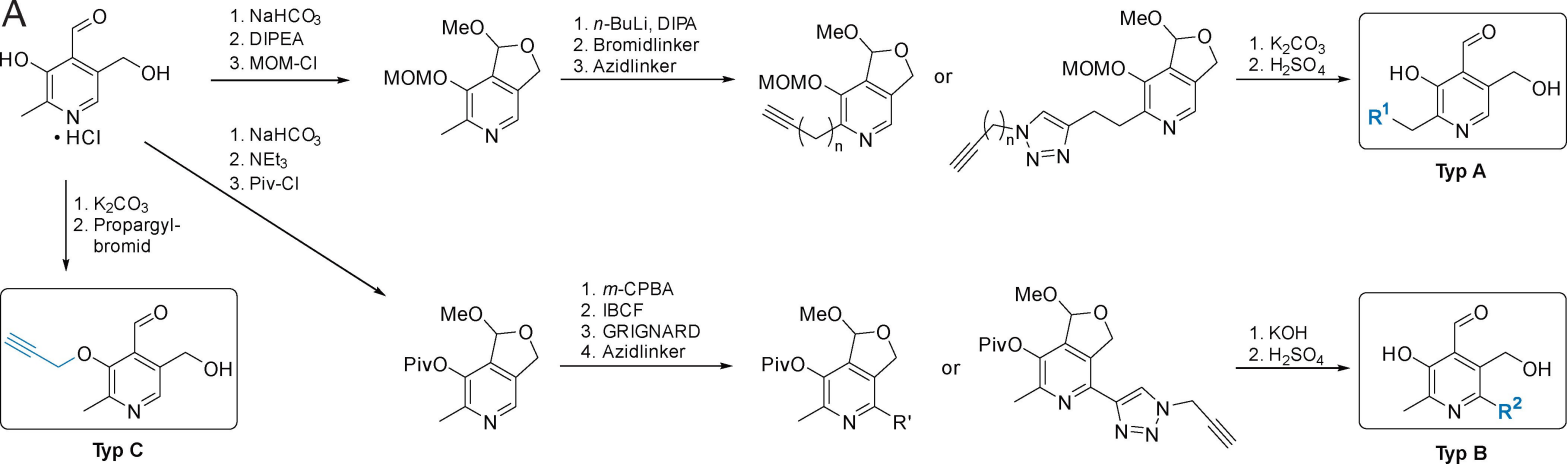
Synthesis scheme for derivatisation of PL to type A, B and C ABPP probes. (**Type A**) **PL** hydrochloride is protected with a methoxymethyl (MOM) group and as a methyl acetal, which is then alkylated at the C2′ position as described previously.[^13^, ^20^] The compound is further modified with an azide linker. Final deprotection leads to **type A** probes. (**Type B**) **PL** hydrochloride is protected to the pivaloyl (Piv) ester, oxidized to the *N*‐oxide with *meta*‐chloroperbenzoic acid (*m*‐CPBA), activated by *iso*‐butyl chloroformate (IBCF) and then reacted with an alkyne‐Grignard reagent to form C6 modified **PL** scaffolds.^[21]^ Final, basic deprotection leads to **type B** derivatives. (**Type C**) Deprotonation of **PL** hydrochloride at the C3 alcohol and substitution with propargyl bromide yields **type C** probe **PL13**.^[22]^

In order to maximize the capture of known and unknown **PLP**‐DEs and achieve sub‐class specificity, we designed and synthesized probes with diverse substitutions at the C2′, C3′ and C6 position including differently spaced alkyl and triazole linkers equipped with a terminal alkyne for CuAAC (Figure 1B). The synthesis of **type A** probes followed the procedures described by Hoegl et al. with slight modifications.[^13^, ^21^, ^23^] In addition, we exploited the modification of the pyridine C6 site by the synthesis of several alkyl and triazole probes. These **type B** derivatives were obtained by a nucleophilic substitution. Prior to that step, pivaloyl protected **PL** was activated by *iso*‐butyl chloroformate (IBCF) as described previously.^[21]^ The set was complemented by one additional probe exhibiting an alkylated C3‐hydroxy group. The synthesis of this **type C** probe, **PL13**, was accomplished by adding propargyl bromide and potassium carbonate to **PL** (Scheme 1).^[22]^

In total, we obtained 13 probes for comprehensive **PLP**‐DE detection (Figure 1B). Prior to in situ labelling studies, we tested if probes bearing structural modifications at all three sites were still recognized by SaPLK/*E. coli* PLK (*pdxK*) and phosphorylated into the corresponding **PLP** derivatives (Figure 1C). Satisfyingly, we observed phosphorylation of all probes except for **PL11** and **PL12** emphasizing that larger modifications at C6 position are not tolerated. Interestingly, differences in substrate specificity of SaPLK vs. *pdxK* were observed, e.g. in case of **PL9** and **PL13** (Figure 1C).

### Screen of Library against Gram‐Positive *S. aureus* Unravels an Enhanced Scope and Individual Target Preferences

We anticipated that lowering the concentration of endogenous **PL** in growth media would in turn maximize the incorporation of probes into **PLP**‐DEs under physiological conditions. In order to optimize our previously established labelling procedure towards this direction, we tested the effect of different media and **PL2** concentrations in a panel of bacterial strains. Chemically defined media (CDM) with a minimal **PL** supplementation of 250 nM provided reliable growth conditions for all tested strains, no matter if the wt or mutant cells devoid of the **PLP** de novo synthesis were used, i.e. *E. coli* (wt and ΔpdxJ), *P. aeruginosa* (wt) and *S. aureus* (wt and TnpdxS) (Figure SI3A–C).

**Figure 2:**
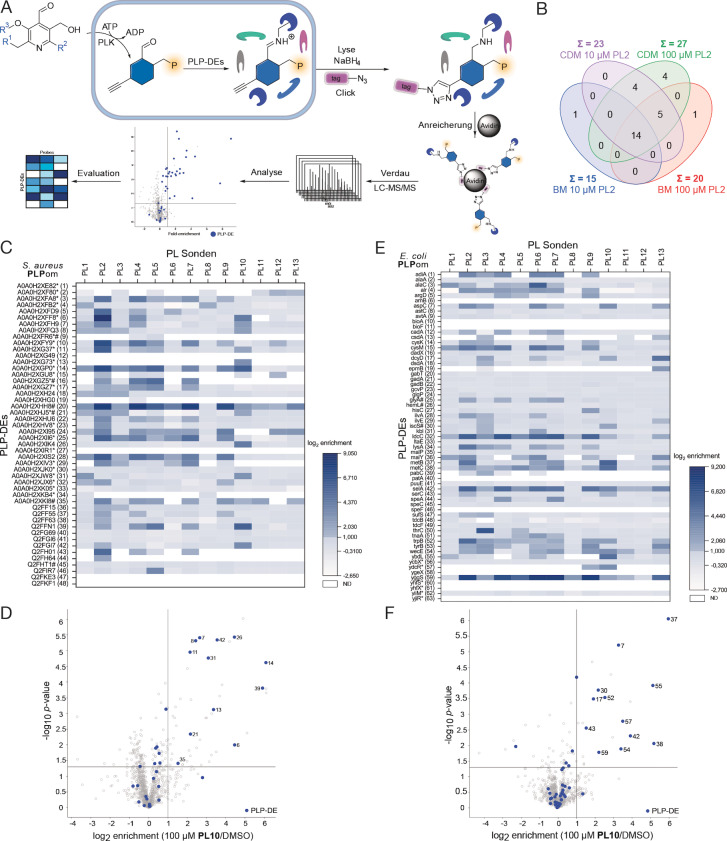
*S. aureus* and *E. coli* PLPome profiling. A) **PL** probes are taken up by the cells, get phosphorylated by PLK and subsequently function as **PLP** surrogates. After cell lysis, internal aldimines are reduced by sodium borohydride for downstream processing. Copper‐assisted azide‐alkyne click chemistry or Staudinger ligation to enrichment tags allows proteomic analysis and **PLP**ome profiling. B) Comparison of enriched **PLP**‐DEs in *S. aureus* using different media (B medium, BM or chemically defined medium, CDM) and different **PL2** concentrations (10 and 100 μM). C) Heatmap representing log_2_ enrichment values of all known or putative **PLP**‐DEs in *S. aureus* for all 13 **PL** probes (*n*=3). Enriched proteins have log_2_ enrichment values higher or equal 1. Proteins not detected (ND) at all are coloured white. **PLP**‐DEs with a putative function or which are poorly characterised are marked with an asterisk. Essential enzymes are marked with a #.^[26]^ D) Volcano plot of **PL10** enrichment at 100 μM in *S. aureus* USA300 TnpdxS compared to DMSO representing the *t*‐test results (criteria: log_2_ enrichment>1 and *p*‐value<0.05, *n*=3). Blue colours depict **PLP**‐DEs. Assigned numbers refer to Table SI1. Remaining plots are shown in Figure SI4. E) Heatmap representing log_2_ enrichment values of all known or putative **PLP**‐DEs in *E. coli* for all 13 **PL** probes (*n*=3). Enriched proteins have log_2_ enrichment values higher or equal 1. Proteins not detected (ND) at all are coloured white. **PLP**‐DEs with a putative function or which are poorly characterised are marked with an asterisk. Essential enzymes are marked with a #.^[28]^ F) Volcano plot of **PL10** enrichment at 100 μM in *E. coli* K12 ΔpdxJ compared to DMSO representing the *t*‐test results (criteria: log_2_ enrichment>1 and *p*‐value<0.05, *n*=3). Blue colours depict **PLP**‐DEs. Assigned numbers refer to Table SI2. Remaining plots are shown in Figure SI6.

We next performed quantitative labelling with the set conditions to unravel and assign the cellular inventory of **PLP**‐DEs. *S. aureus* TnpdxS cells were incubated with 100 μM probe, grown for 2 h, lysed using lysostaphin and clicked to biotin azide or ligated to biotin phosphine (in case of **PL3**)^[24]^ for subsequent enrichment of labelled proteins via avidin beads (Figure 2A). Tryptic digest, analysis via LC‐MS/MS and label‐free quantification (LFQ) revealed a characteristic pattern of significantly enriched proteins [cut‐off criteria: log_2_=1 (2‐fold enrichment) and *p*‐value<0.05] which are visualized in corresponding volcano plots (Figure SI4). First, we benchmarked our refined conditions with the previously established workflow. In total, we could increase the number of significantly enriched enzymes from 20 (B‐medium, 100 μM **PL2**) to 27 (CDM,^[25]^ 100 μM **PL2**) and demonstrate that 100 μM is an ideal concentration to achieve a high coverage (Figure 2B). We next tested all of the probes under the optimized conditions in *S. aureus* USA300 (TnpdxS), which is devoid of de novo **PLP** biosynthesis.^[26]^ Importantly, MS data analysis of the probe library not only revealed an increased overall coverage but also enhanced specificity of individual probes for certain **PLP**‐DEs. For example, cysteine synthase (13), an enzyme crucial for cysteine biosynthesis,^[27]^ was detected for the first time by **PL10** (Figure 2C, D, SI4, Table SI1).

Similarly, other important enzymes such as *A0A0H2XFH9* (7), a Cys/Met metabolism **PLP**‐dependent enzyme, and *A0A0H2XGZ5* (16), an essential gluconate operon transcriptional repressor, were detected solely with our refined labelling protocol.^[26]^

Moreover, *A0A0H2XF80* (2), an uncharacterized helix‐turn‐helix (HTH)‐type transcriptional regulator^[29]^ was only found by novel probes **PL12** and **PL13**, respectively, emphasizing the need for structural diversity to occupy different **PLP**‐binding sites.

Overall, we were able to enhance the coverage of known **PLP**‐DEs by 7 additional proteins which represents a significant improvement gained by the new probe designs as well as the refined methodology.^[30]^ With these promising tools at hand, we commenced to analyze the **PLP**ome in challenging Gram‐negative bacteria.

### Probes Label Intracellular PLP‐DEs in Gram‐Negative *Escherichia coli* and Wild Type *Pseudomonas aeruginosa*

In case of Gram‐negative bacteria, we selected an *E. coli* K12 mutant strain devoid of de novo **PLP** biosynthesis (*E. coli* K12 ΔpdxJ),^[31]^ similar to our *S. aureus* approach. First, we established growth conditions in CDM^[32]^ and found 250 nM of **PL** supplement as minimal concentration to maintain growth (Figure SI3A). Second, we applied the set conditions to monitor the growth of selected **PL** probes (Figure SI5A). Third, we exemplarily performed gel‐based labelling of probe **PL2**, **PL3** and **PL13** in *E. coli* ΔpdxJ as well as in wt cells. Upon probe treatment, CuAAC to rhodamine azide (**PL2**, **PL13**) or SL with rhodamine phosphine (**PL3**) and fluorescent SDS‐PAGE analysis, several labelled protein bands were observed (Figure SI5B). Interestingly, also in case of wt bacteria, specific bands could be detected at the highest probe concentration indicating that probe uptake could be sufficient to compete with endogenous **PLP** derived from de novo synthesis. While the intriguing perspective on wt cell labelling is followed up later in this study, we here focused on *E. coli* K12 ΔpdxJ for quantitative LC‐MS/MS analysis to compare the performance directly with *S. aureus*. For this, we utilized the refined workflow as described above and incubated all 13 probes individually with live cells at 100 μM for 2 h. A closer inspection of the corresponding volcano plots revealed a significant enrichment of 38 enzymes comprising 61 % of functionally assigned or predicted **PLP**‐DEs in *E. coli* (Figure 2E, F, Figure SI6, Table SI2).[^30^, ^33^]

Importantly, this coverage reflects the individual labelling preferences of all probes highlighting that their diversity is key to access the various binding sites. As already observed for *S. aureus*, the extent of labelling greatly varied.

Although **PL2** and **PL3** labelled the majority of **PLP**‐DEs, 29 in total (77 % of all enriched), our structurally tailored probes accessed relevant subclasses, which would otherwise have escaped our attention. Many of these enzymes serve important functions, for example, *alaC*, an aminotransferase involved in the biosynthesis of alanine,^[34]^ was solely enriched by **PL6**, *metB*, a cystathionine gamma‐synthase crucial for methionine biosynthesis^[35]^ as well as *iscS*, a cysteine desulfurase essential for iron‐sulfur cluster formation,^[36]^ were exclusively enriched by **PL10**. While **PL6** exhibits an extended linker at C2′‐position, **PL10** belongs to a group of probes bearing the tag at the C6‐position of the pyridine ring, highlighting the versatility of different structural analogues to satisfy different binding site requirements. Of note, we also identified several proteins of unknown function such as the uncharacterized HTH‐type transcriptional regulator *ydcR*, which was found with probe **PL10**. Based on the overall coverage, we rationalized that the majority of the *E. coli* and *S. aureus* labelling performance was contributed by a set of 6 probes (**PL1**, **PL2**, **PL3**, **PL6**, **PL10**, **PL13**). We therefore focused our following studies on this optimal set of compounds and commenced with the mining of the *P. aeruginosa* PAO1 **PLP**ome. Here, we deliberately used the wt strain in order to decipher if sufficient competition with endogenous **PLP** can be achieved for efficient protein labelling.

**Figure 3:**
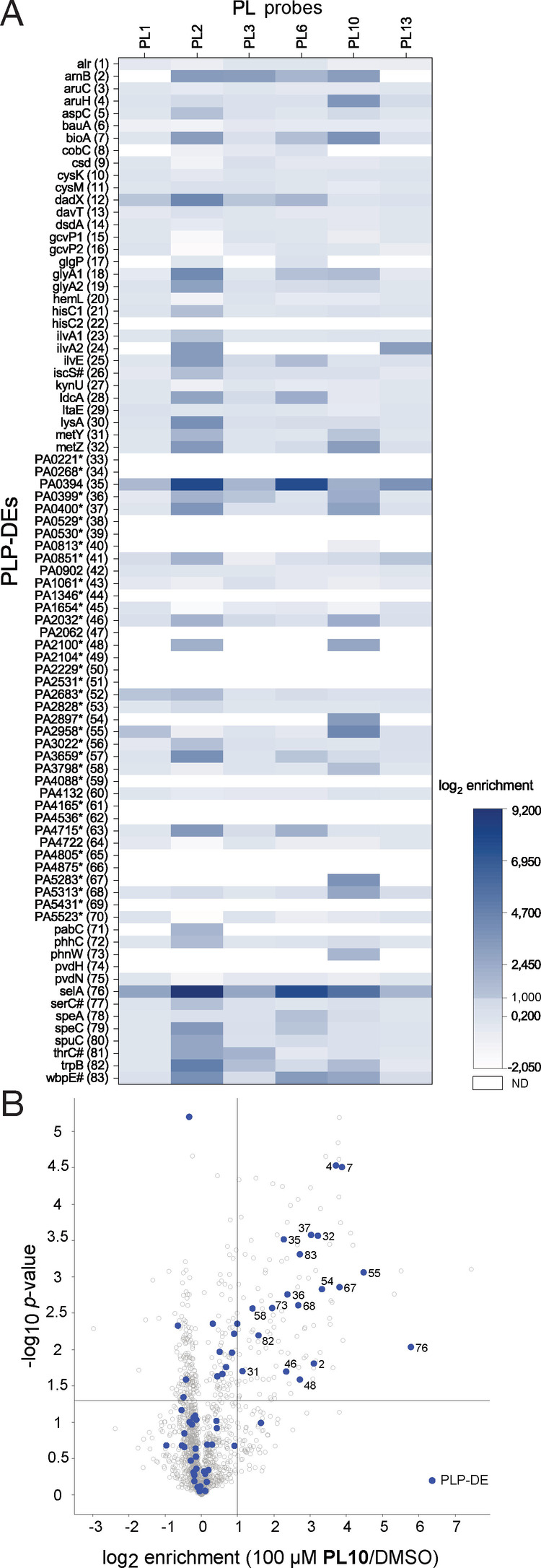
*P. aeruginosa* PLPome profiling. A) Heatmap representing log_2_ enrichment values of all known or putative **PLP**‐DEs in *P. aeruginosa* PAO1 for selected 6 **PL** probes (*n*=3). Enriched proteins have log_2_ enrichment values higher or equal 1. Proteins not detected (ND) at all are coloured white. **PLP**‐DEs with a putative function or which are poorly characterised are marked with an asterisk. Essential enzymes are marked with a #.^[37]^ Remaining plots are shown in Figure SI7. Assigned numbers refer to Table SI3. B) Volcano plot of **PL10** enrichment at 100 μM in *P. aeruginosa* compared to DMSO representing the *t*‐test results (criteria: log_2_ enrichment>1 and *p*‐value<0.05, *n*=3).

Satisfyingly, labelling with 100 μM of each probe resulted in an overall coverage of 42 significantly enriched proteins, which reflect 51 % of the known and predicted *P. aeruginosa* **PLP**‐DEs (Figure 3A, B, Figure SI7, Table SI3).^[30]^ Foremost, three probes **PL2**, **PL6** and **PL10** contributed to the majority of hits. These included e.g. *aruH*, a transaminase involved in arginine degradation^[41]^ (**PL10**), *ilvA2*, a threonine dehydratase inferred from homology^[42]^ (**PL13**), and *PA0394*, a **PLP** homeostasis protein annotated by UniRule^[43]^ (**PL2**, **PL6**), which was also highly enriched in E. coli (*yggS*) and S. aureus (*A0A0H2XHH8*). Interestingly, **PL10** again was able to enrich putative transcriptional regulators (e.g. *PA2032*, *PA2100*, *PA2897* and *PA5283*, annotation by InterPro^[29]^). It seems that the modification of the C6 position is a preferred motif for the recognition of this enzyme class. Further, *PA5313*, a probable **PLP** dependent aminotransferase (InterPro^[29]^ and GO^[42]^) was solely enriched by **PL10**.

Overall, these studies demonstrate that **PL** probes can be applied under our refined conditions to directly label **PLP**‐DEs in wt cells preventing the use of de novo biosynthesis mutants. This eases the application of this method to diverse organisms without their prior genetic manipulation.

### Functional Analysis of Uncharacterized Proteins

**Figure 4:**
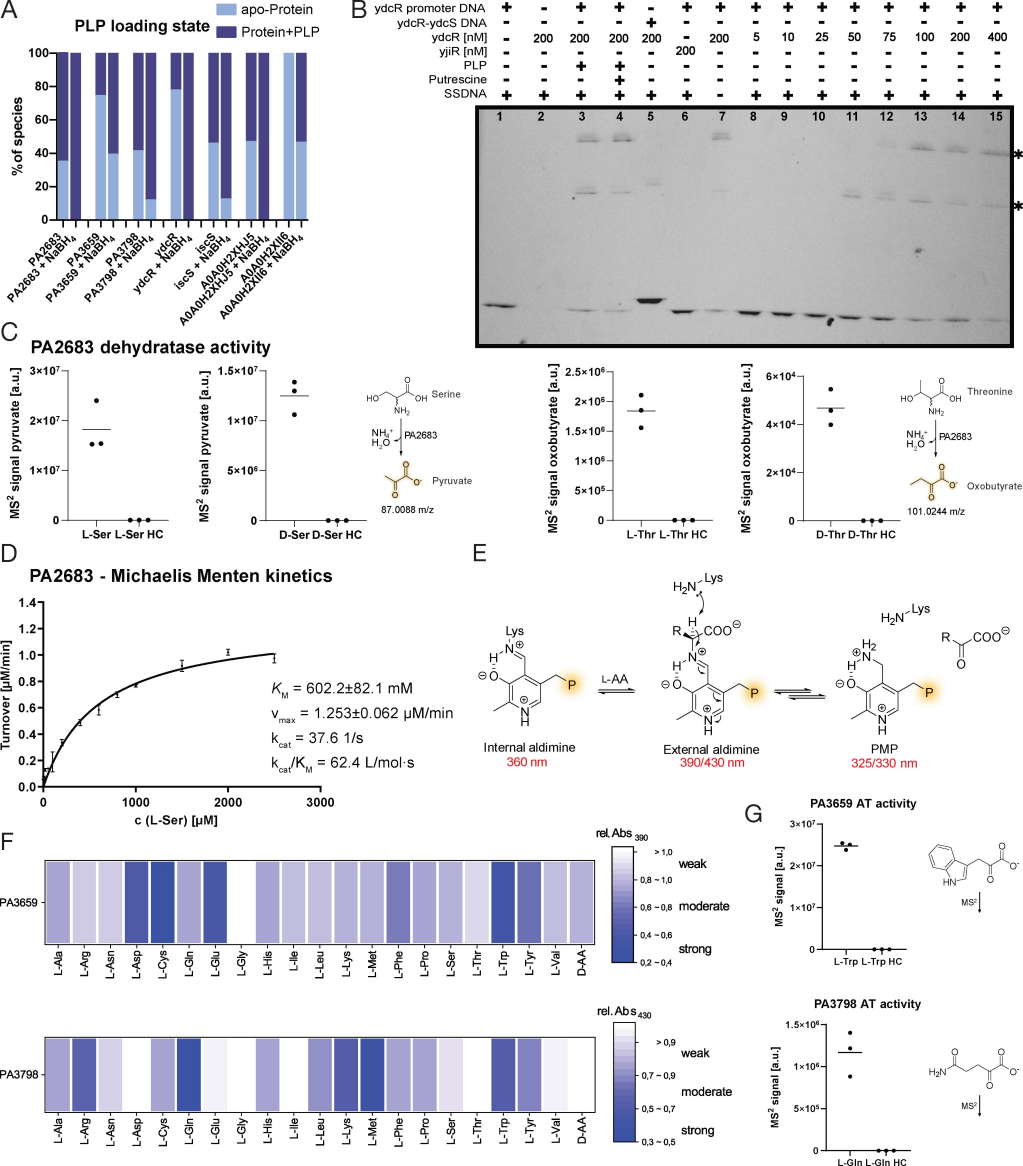
Characterization of enzymes with unknown function. A) **PLP** loading states of overexpressed proteins were determined by IP‐MS as described previously.^[13]^ B) EMSA gel under UV‐illumination. Concentrations are given in nM. The interaction of *ydcR* with the *ydcR* promoter region fragment PR_ydcR_ is clearly visible starting at 50 nM *ydcR*. The two lines (*) represent shifted DNA‐protein complexes.^[38]^ As negative controls, we included samples w/o *ydcR* (lane 1), w/o PR_ydcR_ (lane 2), w/ PR_ydcR‐ydcS_ instead of PR_ydcR_ (lane 5) and one sample with *yjiR* instead of *ydcR* (lane 6). To exclude unspecific DNA binding, we added 2 μg μL^−1^ salmon sperm DNA (SSDNA) to each sample (except lane 7). Samples containing **PLP** (lane 3, 10 eq) and **PLP** and putrescine (lane 4, 10 eq **PLP**, 400 eq) were included (see also Figure SI8). C) LC‐MS/MS analysis of products after incubation of *PA2683* with l‐Ser, d‐Ser, l‐Thr or d‐Thr and additional AMP and **PLP** at 37 °C. The MS^2^ signals of products (pyruvate or oxobutanoate) were integrated and plotted as individual values (*n*=3). Heat control (HC) samples were treated equally except for incubating the protein for 5 min at 95 °C prior to addition of substrates. D) Michaelis–Menten kinetics of *PA2683* (*n*=3, mean±SEM) were calculated in *Graphpad* Prism (see also Figure SI9). E) Spectroscopic properties of **PLP** species during transamination.[^39^, ^40^] F) UV/Vis screening from 300 to 600 nm of aminotransferases *PA3659* and *PA3798* with 20 l‐amino acids (AA) and a mixture of 11 d‐amino acids (each 10 equivalents). Changes in absorbance at 390 or 430 nm (external aldimine) were calculated and normalised. G) LC‐MS/MS analysis of transaminated products after incubation of *PA3659* with l‐Trp and *PA3798* with l‐Gln (*n*=3). Heat control (HC) samples were treated equally except for incubating the protein for 5 min at 95 °C prior to addition of substrates.

With a large panel of uncharacterized **PLP**‐binding proteins from three organisms, we selected candidates for a closer inspection on their functional role. First, **PLP** binding of all overexpressed enzymes was confirmed via intact protein MS (IP‐MS, Figure 4A). We started our studies with *E. coli ydcR* which was identified by **PL10** and is annotated as an uncharacterized HTH‐type transcriptional regulator (TR) (by InterPro^[29]^ and GO^[42]^). We not only confirmed the binding to **PLP** but also determined a high‐affinity interaction with a specific promoter region upstream of a putrescin transporter via electrophoretic mobility shift assays (EMSA) (Figure 4B, Figure SI8). These results are in line with the known roles of related TRs in the **PLP**‐dependent transcription of genes involved in e.g. *gamma*‐aminobutyric acid metabolism.^[44]^

Next, we evaluated a putative enzyme from *P. aeruginosa*, *PA2683* identified by probes **PL1** and **PL2**, which showed homology to the family of serine deaminases (by *GO* ^[42]^) (Figure SI9A).^[42]^ The enzyme was overexpressed and indeed turnover of l‐serine was observed together with NADH consumption within a coupled assay. Moreover, the formation of the expected products pyruvate and oxobutanoate was confirmed by LC‐MS/MS (Figure 4C, Figure SI9B).

*PA2683* exhibited both serine and threonine dehydratase activity, with a preference for l‐enantiomers. Moreover, we could show, that the catalysis was accelerated by adding adenosine monophosphate (AMP) and magnesium^[45]^ which enabled the measurement of Michaelis–Menten kinetics (Figure 4D).

Furthermore, two putative aminotransferases (ATs) (by *InterPro* ^[29]^ and *GO* ^[42]^), *PA3798* and *PA3659*, identified in *P. aeruginosa*, were cloned and overexpressed for a closer inspection of substrate scope. **PLP** has different absorbance properties during its catalytic cycle in transamination due to different electronic states.[^39^, ^40^] This fact can be used in order to study substrate specificities of aminotransferases, because pyridoxamine phosphate (**PMP**) is formed after the catalytic turnover, which absorbs at around 330 nm. Simultaneously, absorbance of the external aldimine at around 390–430 nm decreases (Figure 4E). Incubation of the holo‐enzymes with 20 natural l‐amino acids and a mixture of 11 selected d‐amino acids revealed substrate specificities for predominantly l‐Gln, l‐Met, l‐Trp in case of *PA3798* and l‐Asp, l‐Cys, l‐Glu, l‐Trp in case of *PA3659* via UV/Vis based detection of the external aldimine (Figure 4F). No conversion of d‐amino acids was observed. Enzymes treated with hydroxylamine to displace the cofactor were included as inactive controls. Furthermore, substrates of both aminotransferases were selected and their products quantified via LC‐MS/MS (Figure 4G, Figure SI10).

Although **PLP**‐DE mining narrowed down the number of possible reactions of uncharacterized proteins to only those catalyzed by this cofactor, follow up studies are still required for full functional assignment. Here, we could confirm previously uncharacterized enzymes or enzymes with a putative **PLP** dependency as **PLP**‐DEs and provide insights into their catalysis.

### Screening of a Pyridoxal‐Targeting Library Unravels Novel Antibiotic Hits

A major goal of our **PLP**‐DE profiling approach is the identification of novel antibacterial drug targets together with tailored antibiotic hit molecules. Given the wealth of inhibitors designed to address **PLP**‐DEs in diverse organisms including drugs such as carbidopa,^[18]^ we rationalized that repurposing of these molecules for the inhibition of bacterial **PLP**‐DEs would be an intriguing starting point for the identification of new antibacterial hit compounds. We thus collected a library of 53 molecules consisting of existing drugs as well as other previously described **PLP**‐DE inhibitors, direct binders of the **PLP** cofactor or analogues thereof (Table SI4).

**Figure 5:**
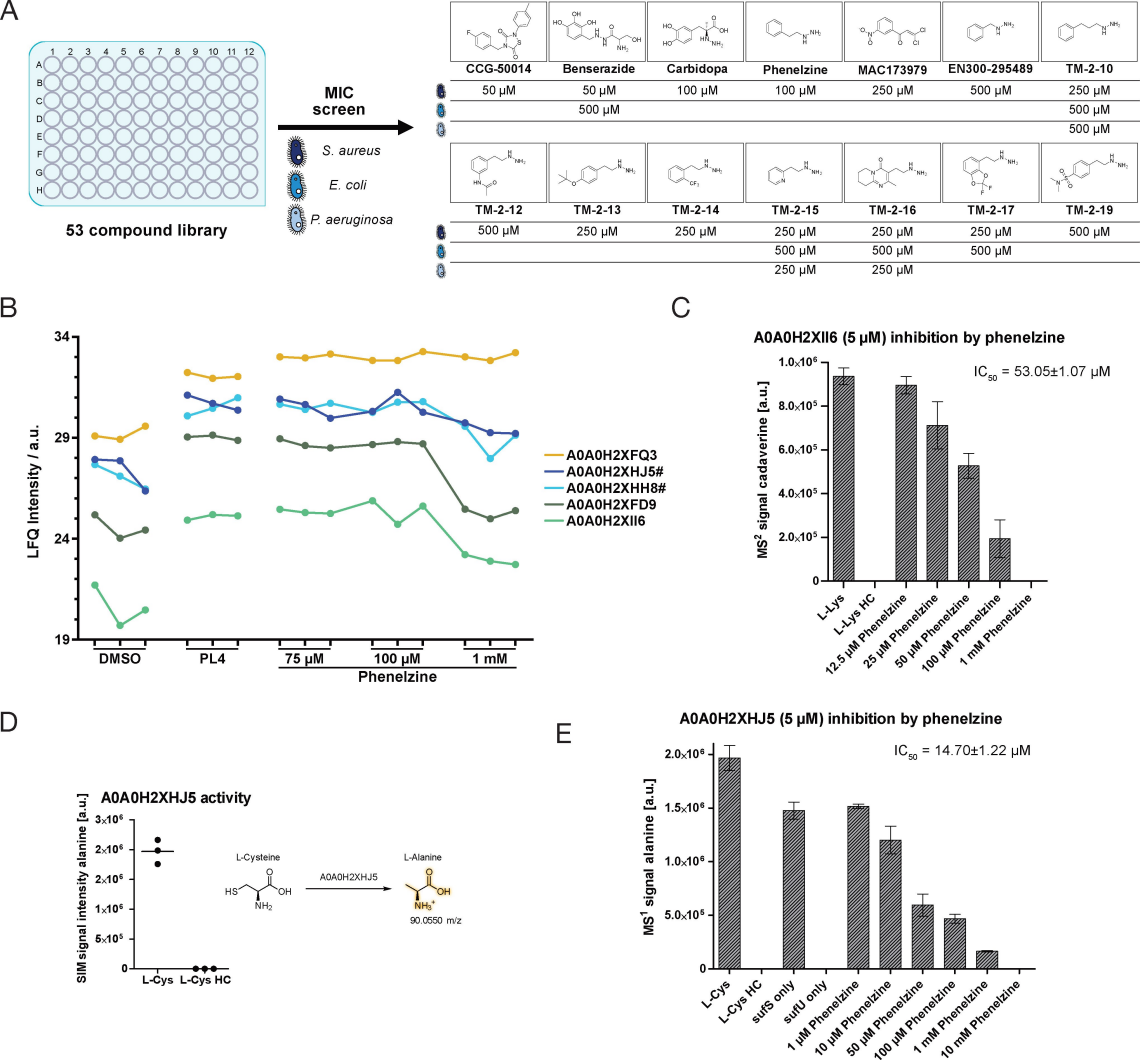
MIC screen and target validation. A) Compound MIC‐screen against *S. aureus*, *E. coli* and *P. aeruginosa*. Structures of hit‐compounds and their corresponding MIC values are shown. B) LFQ intensities of selected **PLP**‐DEs in DMSO, **PL2** treated and phenelzine treated *S. aureus* samples (*n*=3). For competitive labelling samples, cells were incubated with phenelzine for 30 min prior to 2 h **PL2** labelling. Essential proteins are marked with a #.^[26]^ Raw data are given in Table SI7–9 and Figure SI12A. C) Activity of *A0A0H2XII6* after incubation with different concentrations of phenelzine (*n*=3). Quantification of cadaverine was conducted by LC‐MS/MS (MS^2^ signal, *parallel reaction monitoring* PRM). IC_50_ values were calculated in *Graphpad* Prism 5.03 using the log(inhibitor) vs. response‐Variable slope function (four parameters). Heat control (HC) samples were treated equally except for incubating the protein for 5 min at 95 °C prior to addition of substrates. D) LC‐MS analysis of alanine after incubation of *A0A0H2XHJ5* with l‐Cys (*n*=3). Heat control (HC) samples were treated equally except for incubating the protein for 5 min at 95 °C prior to addition of substrates. E) Activity of *A0A0H2XHJ5* (*sufS*) after incubation with different concentrations of phenelzine (*n*=3). Quantification of alanine was conducted by LC/MS (MS^1^ signal, *single ion monitoring* SIM). IC_50_ values were calculated in *Graphpad* Prism 5.03 using the log(inhibitor) vs. response‐variable slope function (four parameters).

These compounds were pre‐screened against bacteria highlighted in this study at 500 μM with hits occurring in *S. aureus* USA300, *E. coli* K12 and *P. aeruginosa* PAO1 (Table SI5). Hit compounds were then fine‐screened down to 25 μM resulting in hits in all 3 strains (Figure 5A).

The approved drugs phenelzine, a nonselective monoamine oxidase inhibitor,^[18]^ benserazide, a structurally related **PLP**‐dependent DOPA decarboxylase inhibitor^[18]^ and CCG‐50014,^[46]^ a mycobacterial alanine racemase inhibitor, were shown to exhibit moderate antibiotic activity against Gram‐positive bacterium *S. aureus* (Figure 5A). Benserazide is a prodrug which forms a hydrazone with the electrophilic **PLP** aldehyde in DOPA decarboxylase.[^18^, ^47^] Similarly, phenelzine exhibits a hydrazine group, which was reported to bind **PLP** in the cell.^[48]^ Interestingly, the synthesis of 11 phenelzine derivatives bearing structural modifications at the aromatic ring position or different alkyl chain lengths were less active suggesting that structural features and not mere hydrazine reactivity are responsible for the biological effect (Figure 5A, Figure SI11, Figure SI12A Table SI6).^[49]^

In order to decipher their targets in living bacteria, we applied phenelzine, benserazide and CCG‐50014 (all three in *S. aureus*) in competitive profiling. We selected **PL2** for these studies due to its high coverage and applied the respective inhibitors in three different molar ratios. Interestingly, all competitive experiments revealed a significant, concentration‐dependent displacement of several **PLP**‐DEs, highlighting the interaction with this enzyme class (Figure 5B, Figure SI12B,C, Table SI7–SI15). For example, competition studies with phenelzine in *S. aureus* revealed strong displacement of several **PLP**‐DEs such as Orn/Lys/Arg decarboxylase (*A0A0H2XII6*). The enzyme was overexpressed and inhibition could be validated in an in vitro targeted metabolomics assay with an IC_50_ of 53.05±1.05 μM (Figure 5C).

Most importantly, among the hits was *A0A0H2XHJ5*, an uncharacterized enzyme, which is essential for cell growth.^[26]^ A closer search revealed that *A0A0H2XHJ5* exhibits homology to an essential cysteine desulfurase (*sufS*) (Figure SI13).[^42^, ^50^] In order to assign its function and test if phenelzine targets this enzyme, we cloned and overexpressed *A0A0H2XHJ5* and investigated its activity in a corresponding assay. Alanine formation was measured after incubation of *A0A0H2XHJ5* with a FeS assembly protein (*sufU*), l‐Cys, dithiothreitol (DTT) and **PLP** at 37 °C.^[51]^ Alanine formation could indeed be reliably quantified by LC‐MS confirming desulfurase activity (Figure 5D). Finally, with an assigned function and established activity assay at hand we confirmed the inhibition of *A0A0H2XHJ5* enzymatic activity by phenelzine with an IC_50_ of 14.70±1.22 μM (Figure 5E).

Given the essential role of cysteine desulfurase for bacterial viability, its inhibition by phenelzine likely contributes to the overall antibiotic effects of this compound.

The mass spectrometry proteomics data have been deposited to the ProteomeXchange consortium via the PRIDE partner repository^[1]^ with the dataset identifier PXD029832.

## Conclusion

We here demonstrate the utility of cofactor mining for discovering unprecedented antibiotic targets on the example of **PLP**‐DEs. Our probe design showcases the importance of introducing structural diversity at various positions of the **PL** scaffold to access different binding sites. With this tailored approach, we managed a coverage of up to 71 % of all **PLP**‐DEs and mapped their binding to certain structural motifs. In fact, these maps may already guide the design of improved **PL** analogues specific for a single enzyme class. Uptake was not only achieved in pathogenic *S. aureus* but also in Gram‐negative *E. coli* and *P. aeruginosa* strains. Our improved labelling conditions furthermore facilitated the detection of **PLP**‐DEs in wild type cells superseding the use of **PLP** biosynthesis mutants. This now opens new possibilities of utilizing the methodology in the profiling of diverse bacterial communities to not only decipher their **PLP**‐DE inventory but also monitor their inhibition by small molecules in situ. Our pilot screen for new **PLP**‐DE inhibitors already revealed several hits which we could trace down to the corresponding cellular target. In case of the marketed drug phenelzine, we demonstrated the inhibition of a previously uncharacterized cysteine desulfurase, an enzyme which lacks homology in human cells,[^50^, ^52^] and thus represents a prime antibiotic target along with an initial inhibitory scaffold.

Tools which allow the number of possible enzymatic transformations to be narrowed down are ideal starting points for functional annotations. Thus, we were able to exemplarily assign the function of previously uncharacterized enzymes including aminotransferases, a deaminase and a transcriptional regulator. In summary, our approach provides access to the unrestricted, comprehensive and rapid screening of new antibacterial targets in diverse pathogenic bacteria.

## Conflict of interest

The authors declare no conflict of interest.

1

## Supporting information

As a service to our authors and readers, this journal provides supporting information supplied by the authors. Such materials are peer reviewed and may be re‐organized for online delivery, but are not copy‐edited or typeset. Technical support issues arising from supporting information (other than missing files) should be addressed to the authors.

Supporting Information

## Data Availability

The data that support the findings of this study are available from the corresponding author upon reasonable request.
