# Supplementary material for: Tailored Pyridoxal Probes Unravel Novel Cofactor‐Dependent Targets and Antibiotic Hits in Critical Bacterial Pathogens
Source: Angew Chem Int Ed Engl. 2022 Apr 12;61(24):e202117724. doi: 10.1002/anie.202117724 (PMC9321722; doi:10.1002/anie.202117724)
Supplement: Supplementary file 1 — Supporting Information [file ANIE-61-0-s001.pdf]

Angewandte Chemie
Eine Zeitschrift der Gesellschaft Deutscher Chemiker

Supporting Information

Tailored Pyridoxal Probes Unravel Novel Cofactor-Dependent Targets and Antibiotic Hits in Critical Bacterial Pathogens

*M. Pfanzelt, T. E. Maher, R. M. Absmeier, M. Schwarz, S. A. Sieber**

Table of Contents

TABLE OF CONTENTS 2
SUPPLEMENTARY FIGURES 3
SUPPLEMENTARY TABLES 13
EXPERIMENTAL PROCEDURES 32
CHEMICAL SYNTHESIS 32
GENERAL METHODS 32
ANALYTICAL METHODS 32
SYNTHETIC PROCEDURES AND ANALYTICAL DATA 33
GENERAL PROCEDURES 33
PROBE SYNTHESIS 35
LINKER SYNTHESIS 37
ALKYLATIONS 40
NUCLEOPHILIC SUBSTITUTION 41
CLICK REACTIONS 42
TMS DEPROTECTION 43
ACETAL AND ETHER DEPROTECTION, FINAL PROBES 45
ACETAL AND ESTER DEPROTECTION, FINAL PROBES 47
PL13 SYNTHESIS 48
SYNTHESIS OF SCREENING COMPOUNDS 49
BIOLOGICAL METHODS 54
CULTURE MEDIA 54
CLONING AND OVEREXPRESSION OF PROTEINS 54
PCR AND GENE PURIFICATION 54
GATEWAY® CLONING 56
SITE-DIRECTED AND LIGATION INDEPENDENT MUTAGENESIS (SLIM) 56
PROTEIN OVEREXPRESSION 56
PROTEIN PURIFICATION OF STREP TAGGED PROTEINS 57
PURIFICATION OF YDCR AND YJIR 57
UV/VIS MEASUREMENTS [21] 58
PROBE PHOSPHORYLATION STUDIES WITH PDXK AND SAPLK 58
LOADING STATE STUDIES VIA INTACT PROTEIN MASS SPECTROMETRY (IP-MS) 58
INTACT PROTEIN MS 58
ASSAY FOR PA2683 ACTIVITY 58
EMSA – ELECTROPHORETIC MOBILITY SHIFT ASSAY 59
GROWTH STUDIES 59
PROTEOMICS 60
ANALYTICAL LABELLING 60
PREPARATIVE LABELLING 60
S. AUREUS USA300 TNPDXS GROWTH, LABELLING AND LYSIS 60
E. COLI K12 ΔPDXJ GROWTH, LABELLING AND LYSIS 61
P. AERUGINOSA PAO1 GROWTH, LABELLING AND LYSIS 61
REDUCTION AND BCA ASSAY 61
CLICK CHEMISTRY 61
STAUDINGER LIGATION 61
MS WORKFLOW 62
MS MEASUREMENT AND ANALYSIS 62
STATISTICAL ANALYSIS OF MS/MS DATA 62
TARGETED METABOLOMICS ASSAYS 63
SUBSTRATE SCREEN FOR PA2683 63
SUBSTRATE SCREEN FOR PA3659 AND PA3798 63
INHIBITION ASSAY FOR A0A0H2XII6 WITH PHENELZINE 63
INHIBITION ASSAY FOR A0A0H2XHJ5 WITH PHENELZINE 64
FURTHER SAMPLE PREPARATION 64
MS MEASUREMENT AND ANALYSIS 64
COMPOUND SCREEN 65
REFERENCES 66
APPENDIX 68
ABBREVIATIONS 68
NMR SPECTRA 70
AUTHOR CONTRIBUTIONS 110

Supplementary Figures

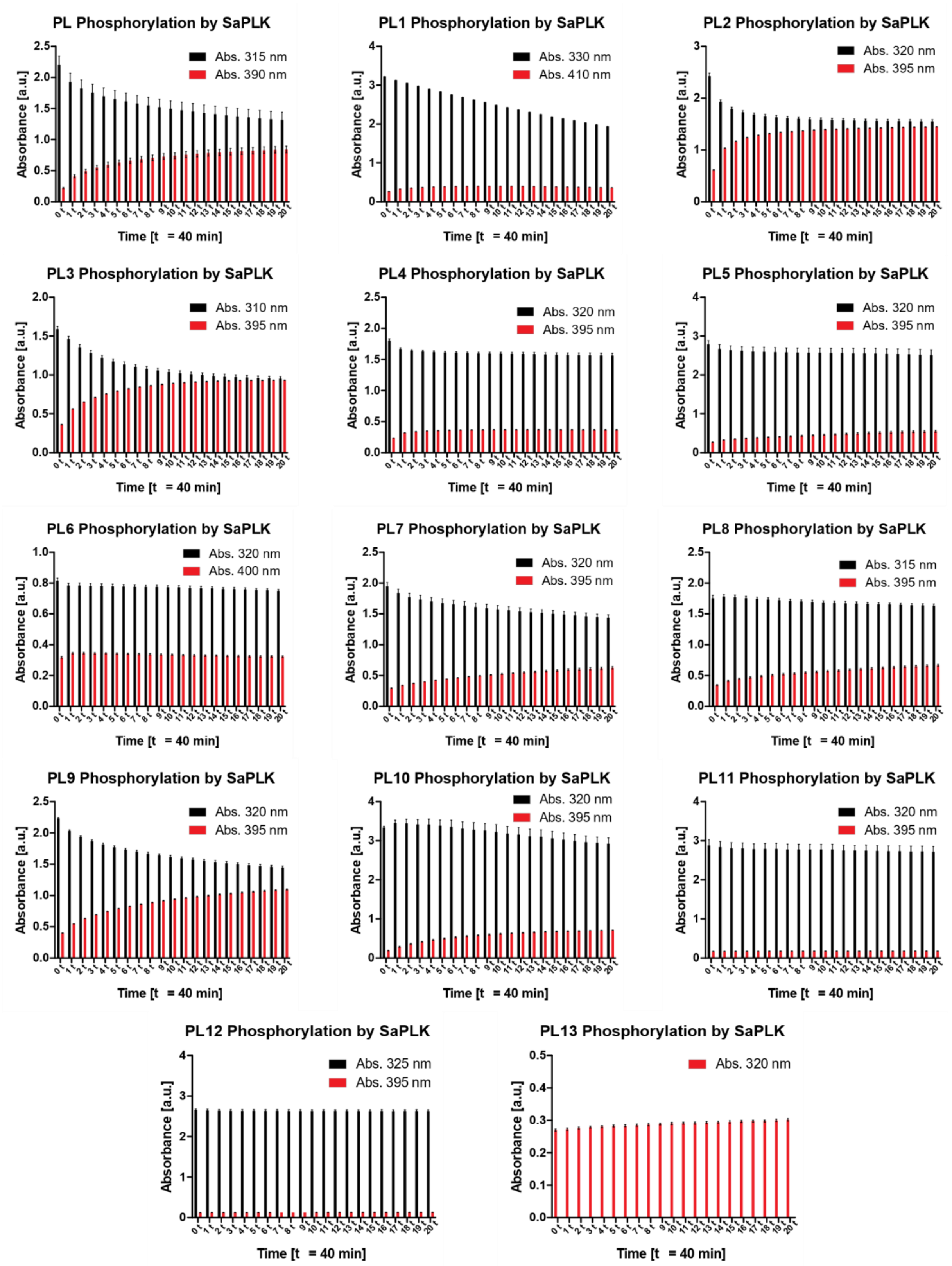

Figure SI 1: Phosphorylation of **PL** probes *in vitro* by pyridoxal kinase (*S. aureus* SaPLK) were monitored by measuring UV/Vis absorbance over time (every 40 min, 20 cycles, n = 3, mean±SEM). Phosphorylated species absorb at around 395 nm. Phosphorylated **PL13** was measured at around 320 nm due to a different absorbance maximum of the phosphorylated species.

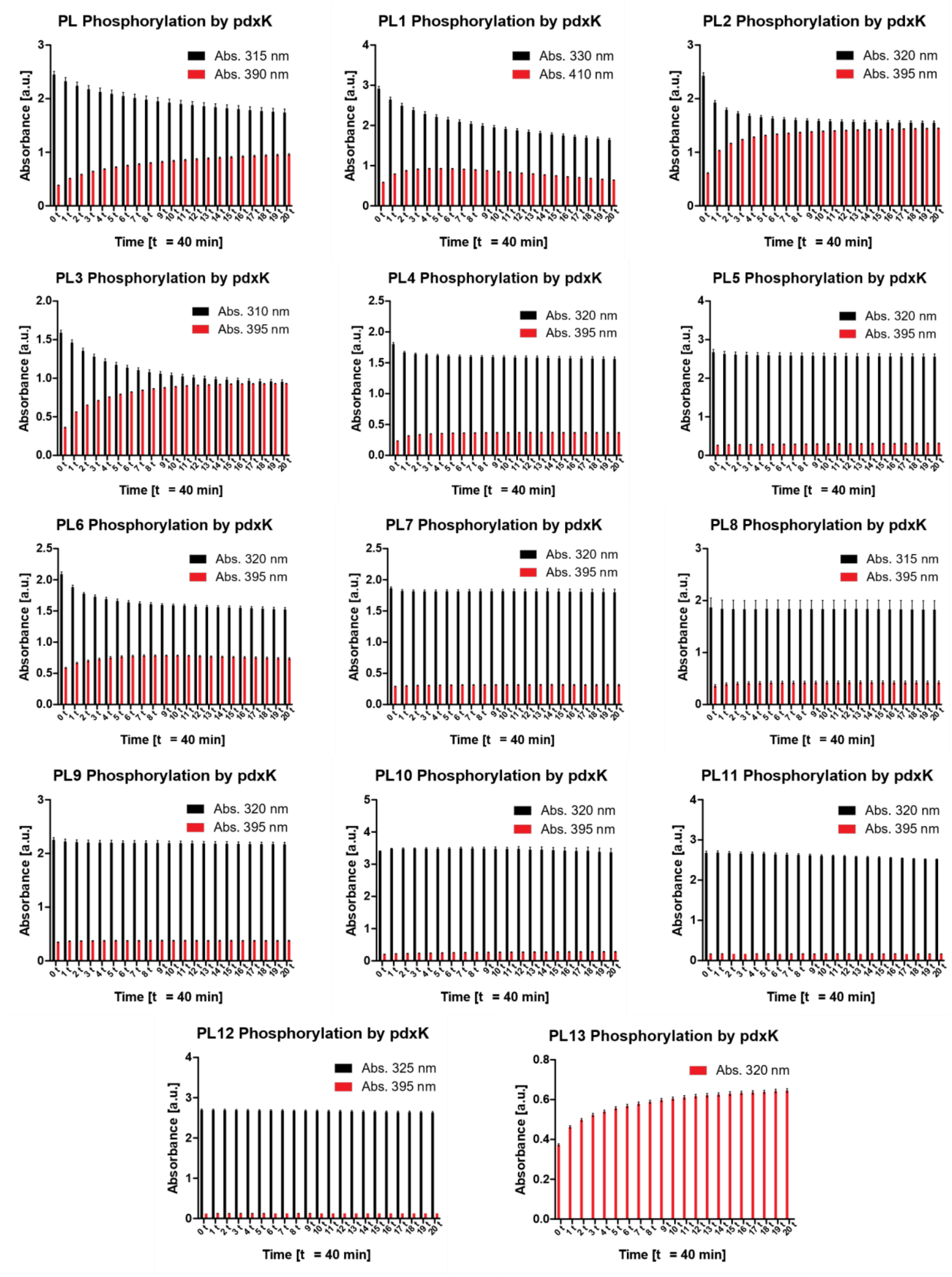

Figure SI 2: Phosphorylation of **PL** probes *in vitro* by pyridoxal kinase (*E. coli* pdxK) can be monitored by measuring UV/Vis absorbance over time (every 40 min, 20 cycles, n = 3, mean±SEM), since phosphorylated species absorb at around 395 nm. Phosphorylated **PL13** was measured at around 320 nm due to a different absorbance maximum of the phosphorylated species.

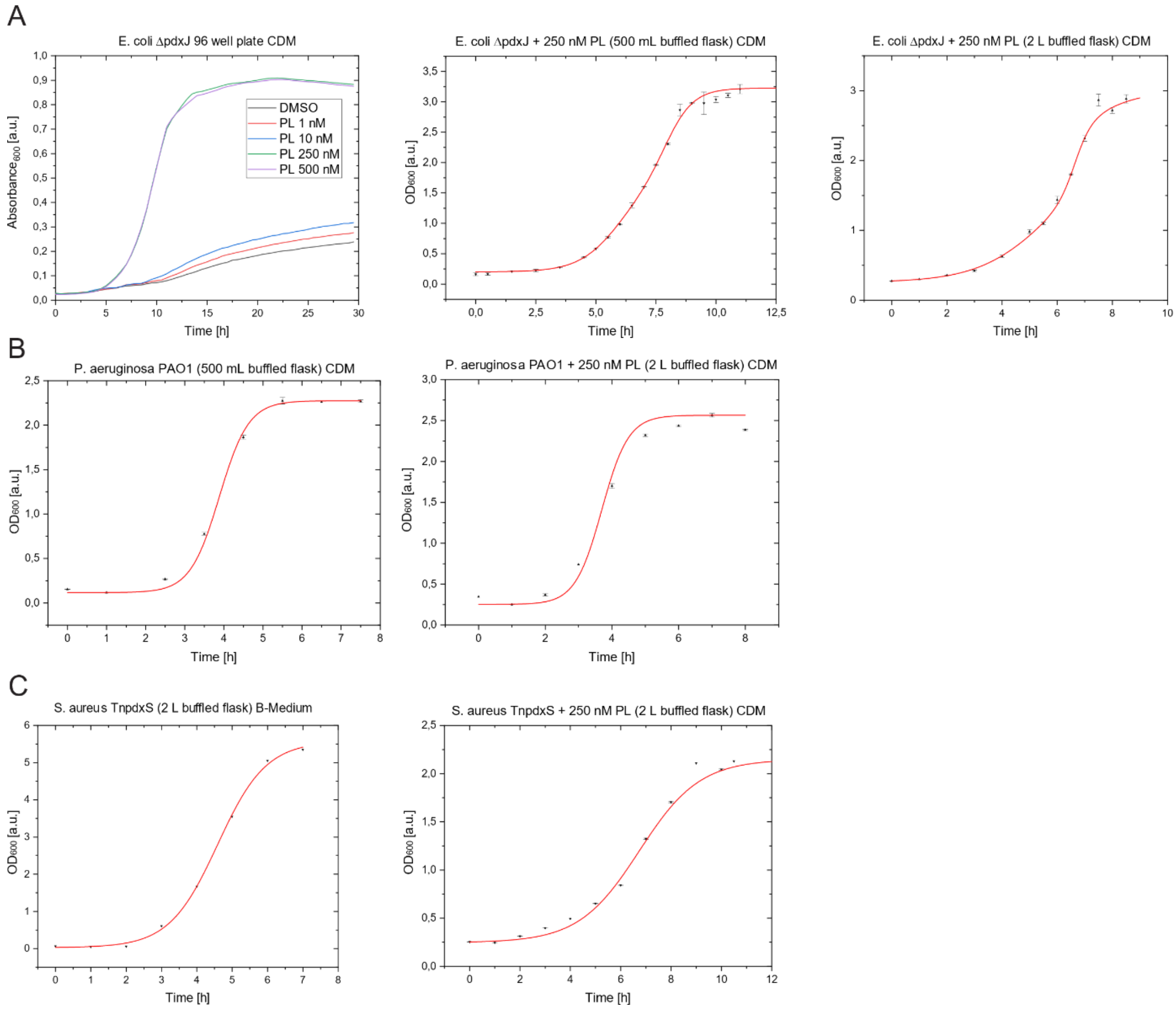

Figure SI 3: Growth Curves. (**A**) *E. coli* K12 ΔpdxJ growth curve studies. First, minimal **PL** concentration was determined for full growth in CDM (**Table SI 17**). Then, growth curves in CDM (+250 nM **PL**) were monitored for 100 mL cultures in 500 mL buffled flasks and 500 mL cultures in 2 L buffled flasks. (**B**) For *P. aeruginosa* PAO1, growth studies in CDM (+250 nM **PL**, **Table SI 17**) were performed for 100 mL cultures in 500 mL buffled flasks and 500 mL cultures in 2 L buffled flasks. (**C**) 500 mL *S. aureus* USA300 TnpdxS cultures in CDM (+250 nM **PL**, **Table SI 17**) or B-medium (**Table SI 17**) were grown in 2 L buffled flasks.

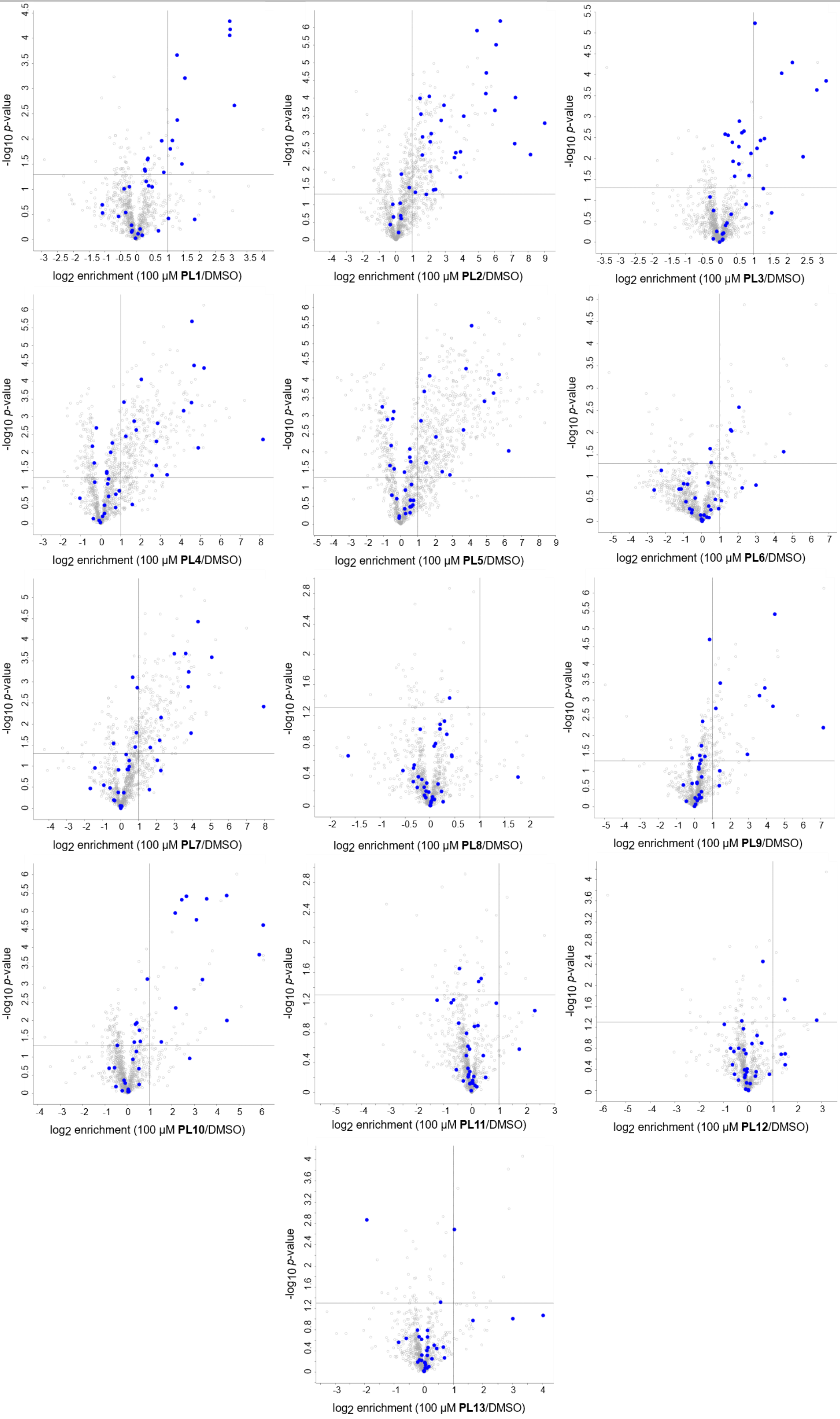

Figure SI 4: Volcano plots for **PL** probe labelling in *S. aureus* USA300 TnpdxS. Proteins depicted in blue are assigned as **PLP**-dependent (**Table SI 1**).

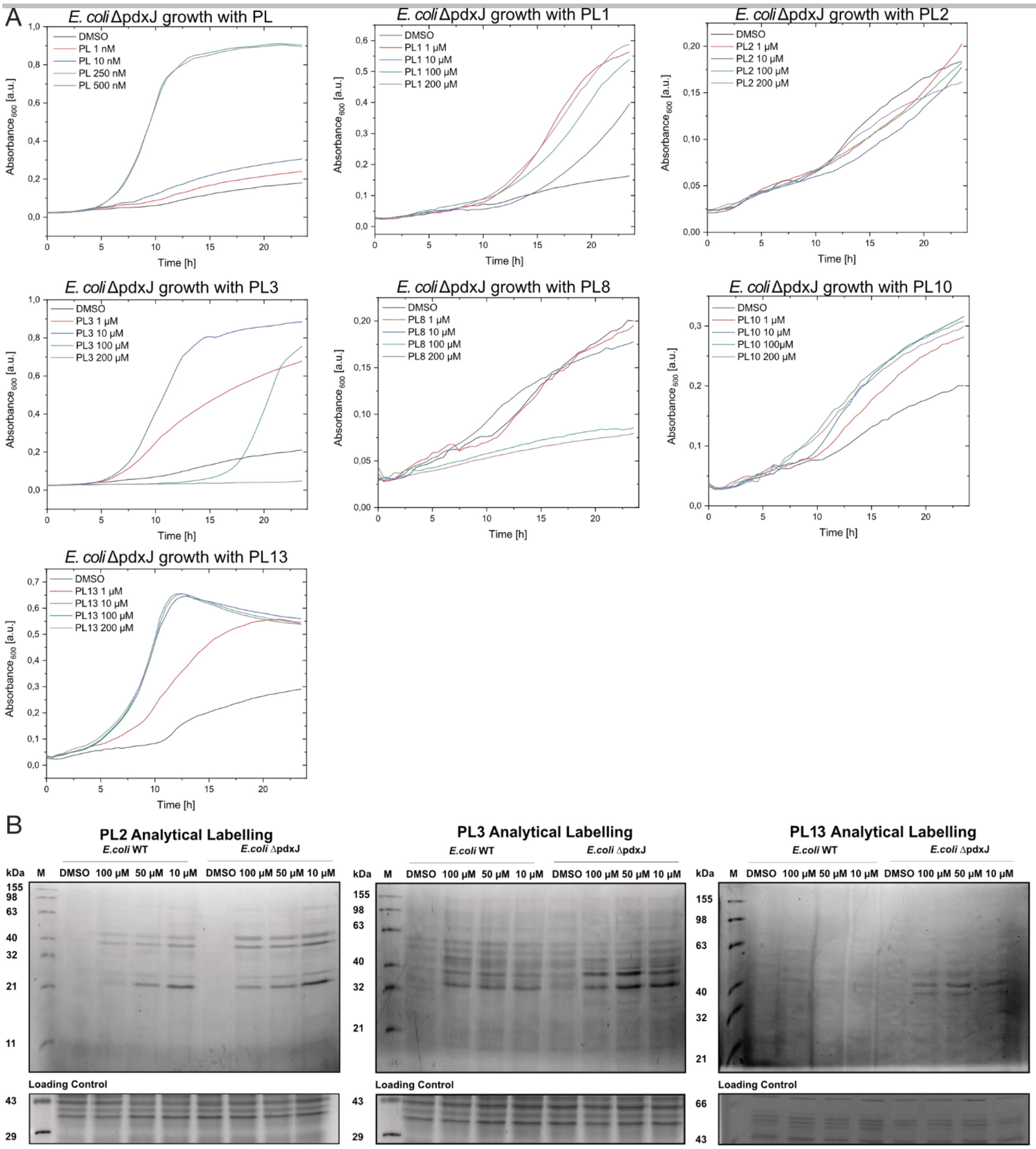

Figure SI 5: (**A**) Growth experiments in *E. coli* K12 ΔpdxJ supplemented with either **PL**, **PL1**, **PL2**, **PL3**, **PL8**, **PL10** or **PL13** at different concentrations in chemically defined medium (CDM). (**B**) Analytical, gel-based labelling with **PL2**, **PL3** and **PL13** in *E. coli* K12 wt and ΔpdxJ at different probe concentrations.

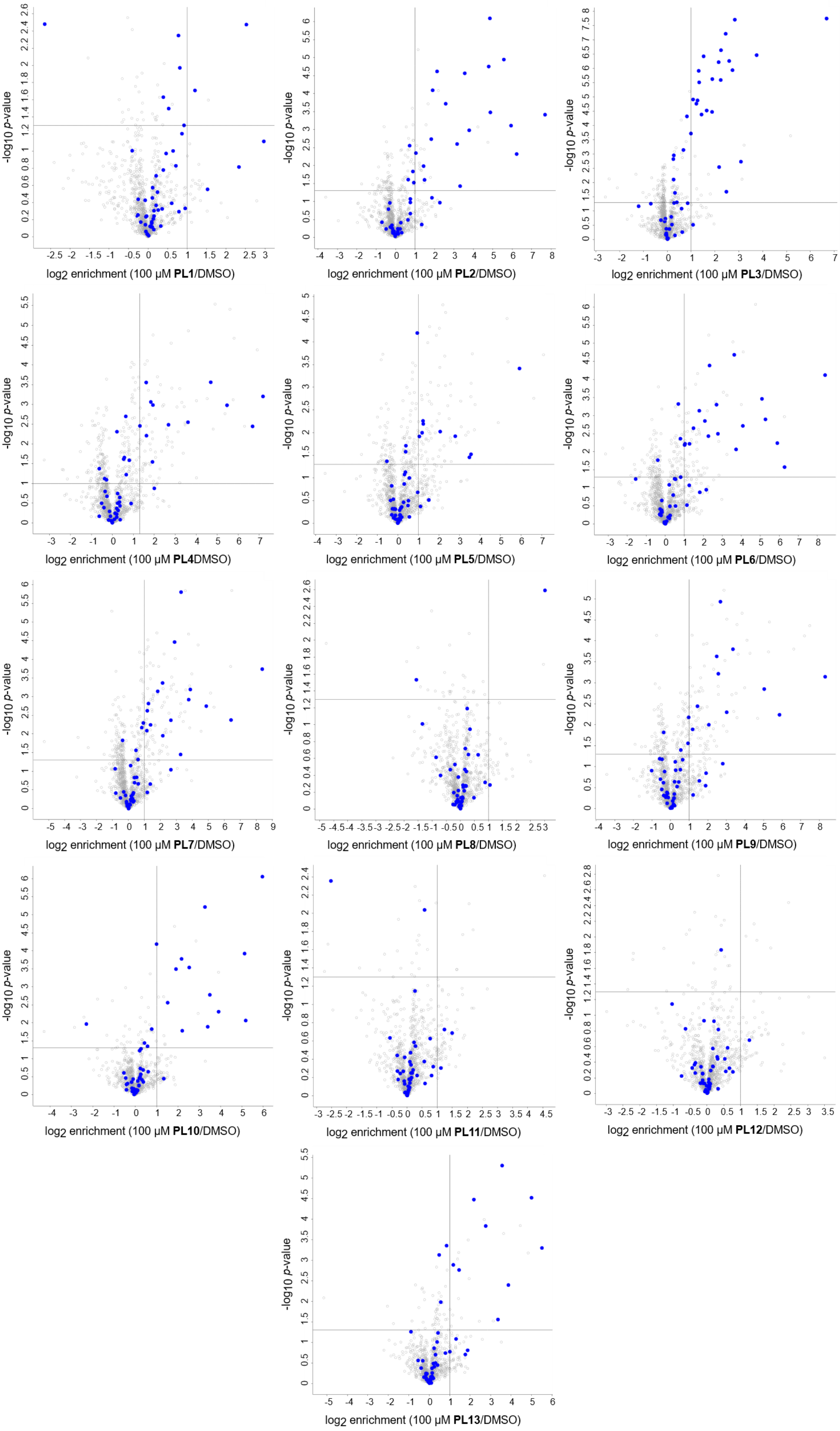

Figure SI 6: Volcano plots for **PL** probe labelling in *E. coli* K12 ΔpdxJ. Proteins depicted in blue are assigned as **PLP**-dependent (**Table SI 2**).

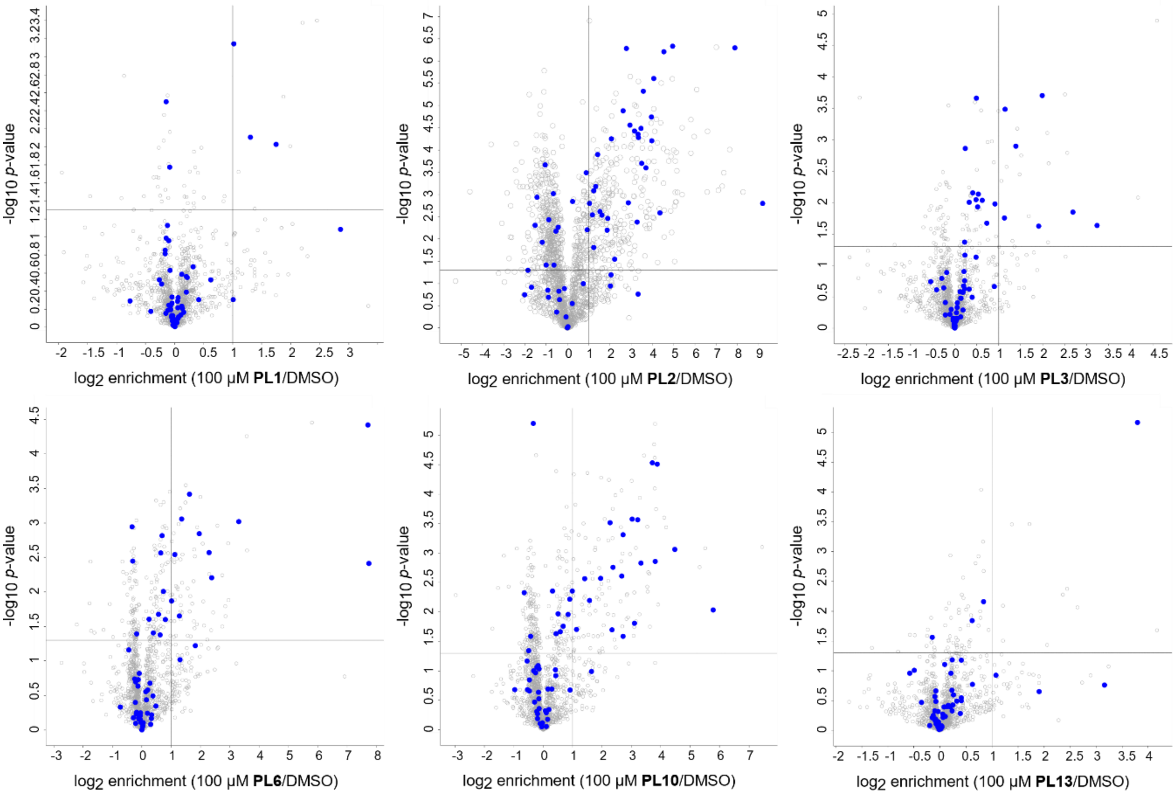

Figure SI 7: Volcano plots for **PL** probe labelling in *P. aeruginosa* PAO1 wt. Proteins depicted in blue are assigned as **PLP**-dependent (**Table SI 3**).

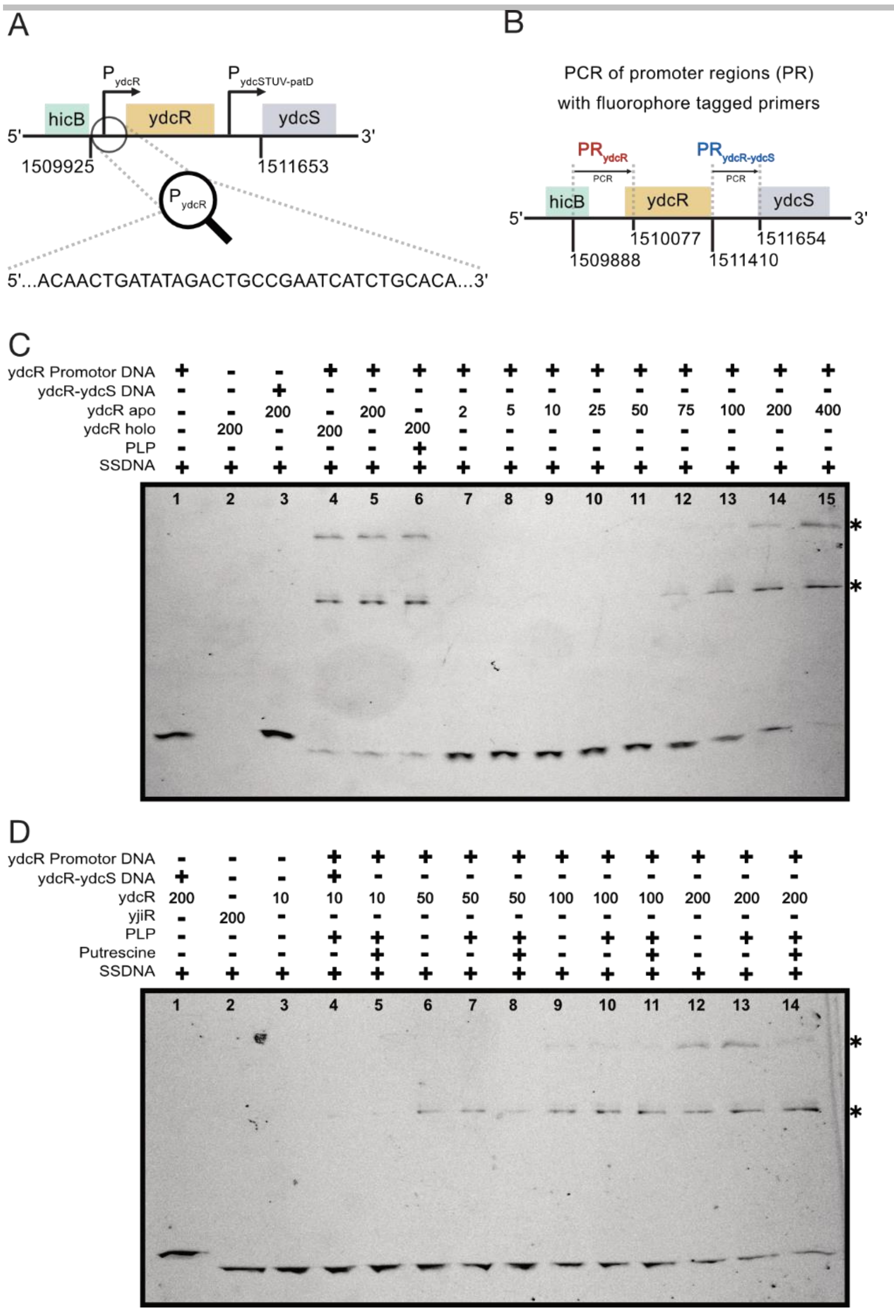

Figure SI 8: EMSA. (**A**) Proposed binding site of *ydcR* between *hicB* and *ydcR*.[2] *ydcR* binds to its own promoter region P_{ydcR}. Downstream, there is the ydcSTUV-patD operon with its own promoter region $P_{ydcSTUV\text{-}patD}$. (**B**) Fluorescent promoter regions PR_{ydcR} (189 bp containing 5'-TAMRA) and $PR_{ydcR\text{-}ydcS}$ (244 bp containing 5'-TAMRA) were generated by PCR with corresponding primers listed in **Table SI 20.** (**C**) Investigation of the influence of **PLP** on the binding of *ydcR* to its promoter P_{ydcR}. Apo-*ydcR* was incubated with 100 eq hydroxylamine for 15 min prior to the EMSA to generate the apo-enzyme. Concentrations are given in [nM]. **PLP** was added at 10 eq. (**D**) Investigation of putrescine and **PLP** concentration on the binding of *ydcR* to its promoter P_{ydcR}. Concentrations are given in [nM]. **PLP** and putrescin were added at 500 µM each. The two lines (*) most likely correspond to different protein-DNA complexes.[3]

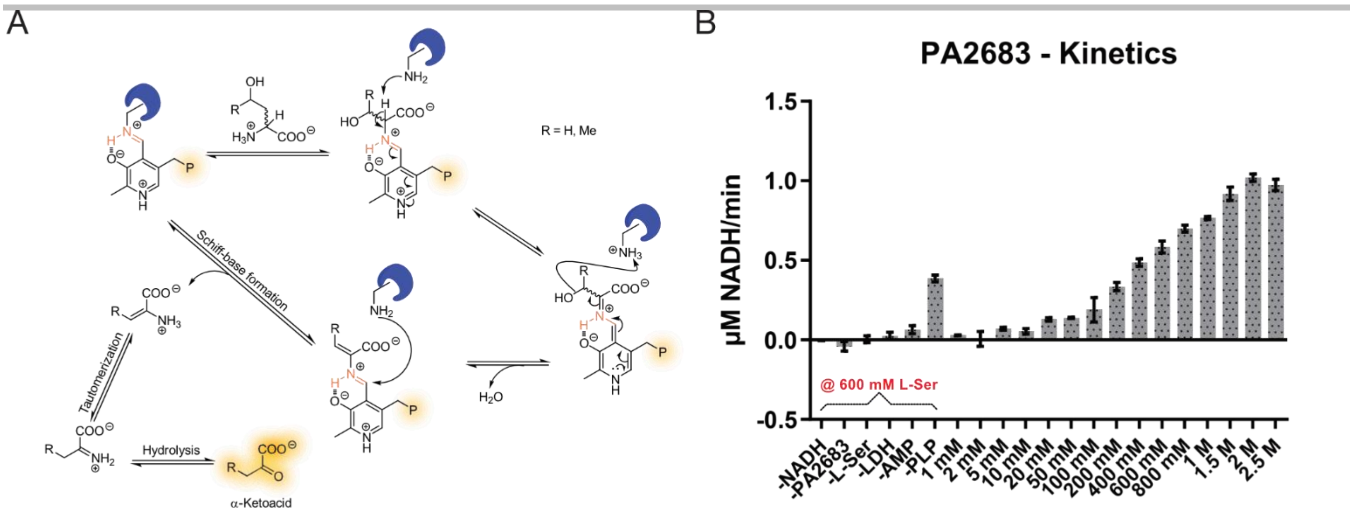

Figure SI 9: (**A**) Catalytic mechanism for serine dehydratase/threonine deaminase forming pyruvate or oxobutanoate.[4] (**B**) *PA2683* assay with control samples at 600 mM L-Ser. The enzyme contains endogenously bound **PLP** sufficient for catalysis (**Figure 4A**).

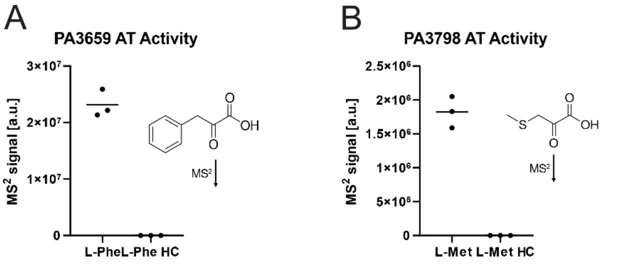

Figure SI 10: Additional substrates for *PA3659* (**A**) and *PA3798* (**B**). LC-MS/MS analysis of transaminated products after incubation of *PA3659* with L-Phe and *PA3798* with L-Met (n = 3). Heat control (HC) samples were treated equally except for incubating the protein for 5 min at 95 °C prior to addition of substrates.

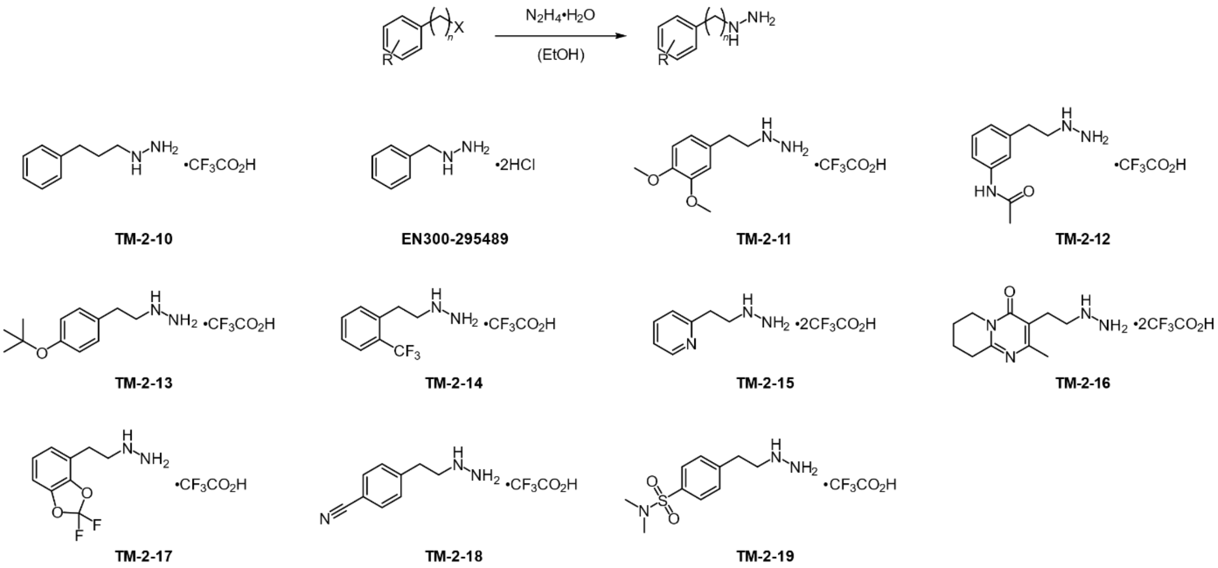

Figure SI 11: Structures of phenelzine derivatives. MIC values are given in **Table SI 6**.

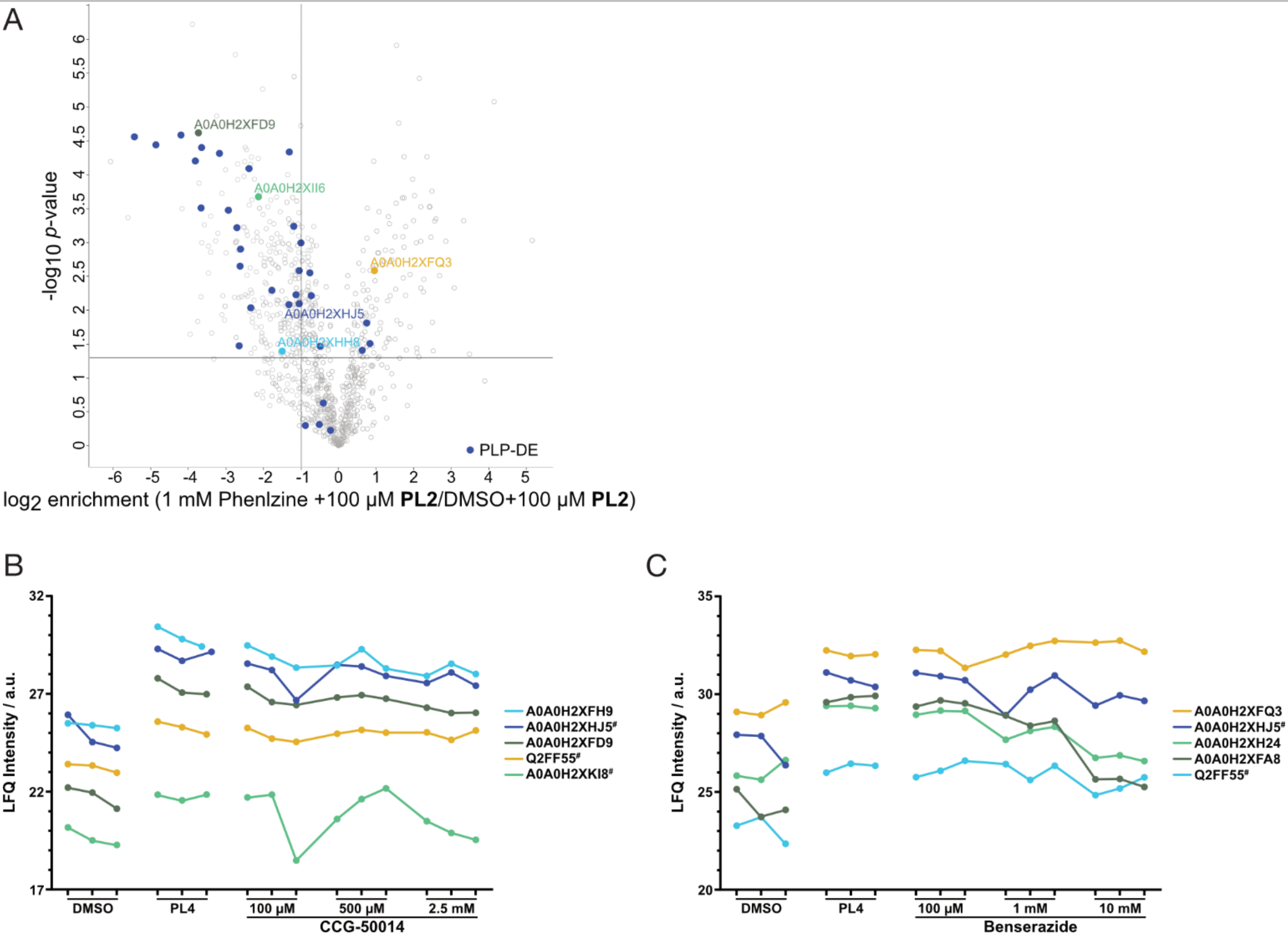

Figure SI 12: Competitive labelling experiments. (**A**) Scatter plot of competitive phenelzine profiling at 1 mM and 100 µM **PL2** in *S. aureus* compared to DMSO and 100 µM **PL2** representing the *t*-test results (criteria: $\log_2$ enrichment > -1 and *p*-value < 0.05, n = 3). (**B**) Profile Plot of selected proteins from CCG-50014 competitive labelling in *S. aureus* USA300 TnpdxS. Essential proteins are marked with a #.[5] (**C**) Profile Plot of selected proteins from benserazide competitive labelling in *S. aureus* USA300 TnpdxS. Essential proteins are marked with a #.[5]

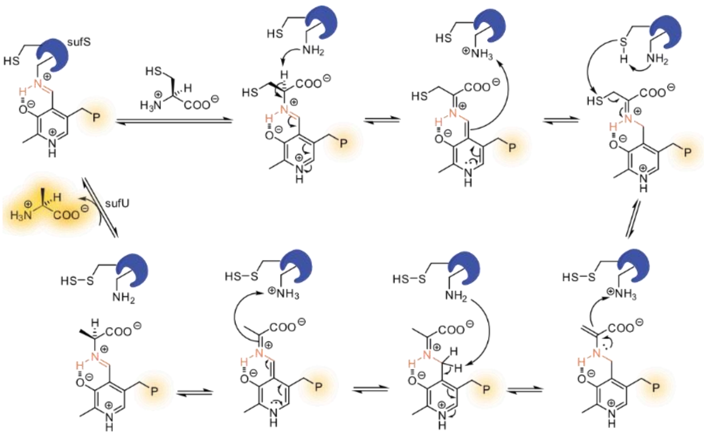

Figure SI 13: General cysteine desulfurase (*sufS*) catalytic reaction mechanism.[6] *sufU* is needed for the regeneration of the catalytic cysteine.[7]

Supplementary Tables

Table SI 1: log_2 enrichment (probe/DMSO) values for enrichment in *S. aureus* USA300 TnpdxS. Putative, uncharacterized or poorly characterized **PLP**-dependent enzymes are highlighted with a *.

	PL1	PL2	PL3	PL4	PL5	PL6	PL7	PL8	PL9	PL10	PL11	PL12	PL13
**A0A0H2XE82* (1)**	--	--	--	--	--	--	--	--	--	--	--	--	--
**A0A0H2XF80* (2)**	-1,08414	--	0,09432	--	-0,38664	--	--	-0,11502	--	--	0,9022	1,48623	1,03155
**A0A0H2XFA8* (3)**	0,25965	4,91894	0,15771	4,57367	1,67137	1,63429	2,97393	0,20509	1,42732	0,04267	-0,19543	-0,00464	0,13914
**A0A0H2XFB2* (4)**	1,84397	0,16767	-0,18846	-0,03617	-0,52175	-2,63006	-1,70015	1,77034	0,2542	0,06352	-0,74329	-0,97785	-0,20897
**A0A0H2XFD9 (5)**	0,05026	5,4698	0,92426	1,67399	0,22793	1,58091	0,80045	0,00322	0,39225	0,03659	-0,12188	-0,14407	-0,14757
**A0A0H2XFF8* (6)**	0,6943	9,01239	0,77936	3,32377	0,69914	0,27489	-0,34338	0,26473	-0,46109	4,45942	--	-0,10962	-0,85312
**A0A0H2XFH9 (7)**	1,07418	4,10839	0,56717	2,03346	-0,56259	-0,87862	0,6581	0,01911	0,13771	2,64293	0,34626	0,54366	0,56523
**A0A0H2XFQ3 (8)**	1,44219	1,60868	1,10674	0,39134	-0,40473	-0,06312	-0,0205	0,011	0,83108	2,43423	-0,07541	-0,10177	-0,07485
**A0A0H2XFR6* (9)**	--	--	--	-0,09786	0,74575	--	--	--	--	--	--	--	--
**A0A0H2XFY9* (10)**	0,38814	7,20174	1,21086	4,89199	2,85207	0,39258	3,90895	0,2895	1,37536	-0,50516	0,51396	1,50015	0,6985
**A0A0H2XG37* (11)**	0,86332	6,06741	0,5819	1,1497	1,3705	0,04322	0,92263	0,0878	0,28895	2,14205	-0,13253	0,58922	0,09997
**A0A0H2XG49 (12)**	--	--	--	--	--	--	--	--	--	--	--	--	--
**A0A0H2XG73* (13)**	--	--	--	--	--	--	--	--	--	3,36617	--	--	--
**A0A0H2XGP0* (14)**	2,97034	7,23303	2,88196	4,67439	5,71146	0,92358	3,78551	0,16266	3,89286	6,0877	-0,65593	-0,59478	0,10394
**A0A0H2XGU8* (15)**	--	1,17082	--	0,73473	0,54279	--	--	--	--	0,52906	--	1,34282	--
**A0A0H2XGZ5* (16)**	--	2,74854	-0,18988	4,54615	5,39585	--	3,61679	--	--	--	--	--	--
**A0A0H2XGZ7* (17)**	-0,22195	1,83996	1,29404	2,77666	3,64382	-1,0067	2,15642	-0,19873	0,25795	-0,81604	-0,55704	-0,56564	-0,59669
**A0A0H2XH24 (18)**	1,2883	2,0164	1,84212	0,29437	0,20957	0,51291	0,4713	0,02074	0,43946	0,36291	-0,0226	-0,06331	0,12252
**A0A0H2XHG0 (19)**	--	--	--	-0,38093	--	--	--	--	--	--	--	--	--
**A0A0H2XHH8 (20)**	3,10404	8,15499	2,47296	8,13857	6,26825	4,51037	7,94715	0,4404	7,14618	2,79565	1,73538	1,50471	4,02898
**A0A0H2XHJ5* (21)**	1,53346	2,27641	2,15774	-1,06287	0,28394	-2,23184	-1,43996	0,19746	-0,62337	2,16575	0,42214	0,31981	0,42984
**A0A0H2XHU6 (22)**	0,30801	3,54928	0,66048	1,76843	1,1772	0,37864	1,64466	0,04942	0,58017	0,54394	0,08255	0,35441	0,11282
**A0A0H2XHV8* (23)**	-0,56558	2,91549	0,33737	0,49385	0,52662	-1,1468	0,30192	-0,33681	0,29632	0,43284	0,03926	-0,15556	0,00704
**A0A0H2XI95 (24)**	1,13634	3,90824	0,21697	2,84674	2,04555	2,22231	2,23194	0,22854	4,34528	--	--	2,79051	3,00801
**A0A0H2XII6* (25)**	-0,15332	6,00101	3,15812	4,15316	1,47259	2,98356	5,06614	-0,54265	3,59542	0,06757	-1,25623	-0,72271	0,16722
**A0A0H2XIK4 (26)**	0,3511	0,33819	0,25082	-0,23606	-0,46604	-0,78807	-0,0013	0,11117	0,0298	4,44618	0,1058	-0,05108	0,02828
**A0A0H2XIR1* (27)**	--	--	--	--	--	--	--	--	--	--	--	--	--
**A0A0H2XIS2 (28)**	2,96032	6,32416	1,3271	5,18209	4,11747	2,04804	4,29555	0,3358	4,44545	0,2528	-0,10654	0,00105	-0,16732
**A0A0H2XIV3* (29)**	--	3,63244	1,54046	0,7474	0,62582	1,08259	3,75021	--	0,37715	--	--	--	--
**A0A0H2XJK0* (30)**	-0,03317	0,32365	0,11078	0,34121	0,53002	0,01027	0,47104	-0,25801	0,14236	0,8949	-0,12884	-0,20586	-0,08189
**A0A0H2XJW8* (31)**	2,9582	0,26374	0,71998	-0,31942	-0,08523	-0,37004	-0,13267	0,39267	-0,13206	3,09163	-0,06648	-0,17502	0,03691
**A0A0H2XJX6* (32)**	1,29621	2,40061	-0,00221	0,92582	0,53751	0,49646	-0,93872	-0,31568	1,3975	-0,22389	0,18851	0,8587	0,65402
**A0A0H2XK05* (33)**	-1,07728	-0,33892	0,07632	0,38771	-0,09277	0,12209	-0,38331	-0,32978	0,0609	-0,57439	-0,47075	-0,40064	-1,92921
**A0A0H2XKB4* (34)**	0,18029	--	--	--	--	--	--	0,43603	--	--	--	--	--
**A0A0H2XKI8 (35)**	0,50165	3,89091	0,8738	2,56949	2,41327	--	2,03775	-0,11891	2,92699	1,52153	2,3031	--	1,65623
**Q2FF15 (36)**	0,36573	1,51848	1,04262	0,07867	0,26254	0,46078	0,41226	-0,06433	0,35079	0,40729	0,04821	-0,1234	0,04982
**Q2FF55 (37)**	0,80413	2,08203	0,37462	1,24948	-0,23586	0,74239	0,86226	-0,02319	1,17437	-0,14581	-0,06572	0,07417	0,04789
**Q2FF63 (38)**	1,01731	1,60416	0,56884	0,17038	0,6216	-0,53566	-0,59235	-1,64615	0,14622	0,5202	-0,3018	0,28774	0,34746
**Q2FFN1 (39)**	0,11804	2,08634	0,44034	2,7823	4,85744	-1,26585	2,24198	-0,23457	0,36894	5,9089	0,13014	-0,64678	0,27374
**Q2FG69 (40)**	-0,38989	-0,19348	0,0741	-0,43152	-0,77787	-0,7007	-0,14324	-0,08113	-0,13529	0,58117	-0,18495	-0,26679	-0,07347
**Q2FGI6 (41)**	-0,15851	-0,14892	0,1722	-0,34192	-0,63137	-0,67651	-0,39591	0,0441	-0,00758	0,31277	0,25354	0,14796	0,12486
**Q2FGI7 (42)**	0,27925	1,45725	0,39609	0,28693	-1,06872	-0,02084	0,36694	0,08008	0,22787	3,54894	0,23011	-0,00542	0,00597
**Q2FH01 (43)**	--	5,4445	--	1,57587	0,56103	--	1,59325	--	--	--	--	--	--
**Q2FH64 (44)**	--	2,12949	--	--	--	--	--	--	--	--	--	--	--
**Q2FHT1 (45)**	-0,136	0,28872	-0,29209	0,18082	0,57838	0,34243	0,16558	-0,0836	0,38449	-0,08831	-0,0121	-0,07426	0,01011
**Q2FIR7 (46)**	-0,33916	0,80627	-0,09147	0,57352	3,79416	-0,56243	0,05515	-0,16487	0,23725	-0,45107	-0,44557	-0,39551	-0,21629
**Q2FKE3 (47)**	--	--	--	--	--	--	--	--	--	--	--	--	--
**Q2FKF1 (48)**	--	--	--	--	--	--	--	--	--	--	--	--	--

Table SI 2: log_2 enrichment (probe/DMSO) values for enrichment in *E. coli* K12 ΔpdxJ. Putative, uncharacterized or poorly characterized **PLP**-dependent enzymes are highlighted with a *.

	PL1	PL2	PL3	PL4	PL5	PL6	PL7	PL8	PL9	PL10	PL11	PL12	PL13
adiA (1)	--	3,77605	2,16488	3,5999	--	4,07095	3,7636	--	1,89969	--	--	--	--
alaA (2)	-0,05841	-0,28359	0,24267	-0,40858	-0,00548	-0,29424	-0,10791	-0,36247	-0,54974	-0,45286	-0,32564	-0,15729	-0,16233
alaC (3)	2,96456	1,20979	-0,27544	1,48379	1,09582	6,27226	1,16842	-0,26096	0,07501	-0,06246	0,59061	-0,7597	0,09895
alr (4)	-2,65317	3,14694	2,59375	2,67323	2,06293	2,76396	2,65239	-1,56388	2,0485	-2,29761	-2,52841	-1,03863	-0,54796
argD (5)	-0,0203	-0,45666	0,26912	1,93292	1,04638	-0,18254	0,37062	0,08641	2,56428	0,03024	0,09139	0,00745	0,13031
arnB (6)	--	--	--	--	--	--	--	--	--	--	--	--	--
aspC (7)	0,77569	3,54728	2,23612	0,53734	0,38024	3,62542	1,17758	0,24448	0,23921	3,26363	0,25732	0,20374	2,17955
astC (8)	0,25484	-0,15071	0,58774	-0,35119	0,09345	-0,05496	-0,3006	0,20058	-0,38936	0,30595	0,05794	0,30156	-0,04734
avtA (9)	0,45859	0,87522	0,67171	0,20578	-0,3538	0,48393	0,35876	-0,01748	0,64917	0,2168	-0,014	-0,13155	0,14395
bioA (10)	--	--	--	--	--	--	--	--	--	--	--	--	--
bioF (11)	--	--	--	--	--	--	--	--	--	--	--	--	--
cadA (12)	0,19853	1,47026	-1,20452	1,90305	0,11306	1,25257	2,1473	-0,04689	-0,18503	-0,41995	-0,30007	-0,19803	-0,06552
csdA (13)	--	--	1,07739	--	--	--	0,28864	--	--	--	0,32721	--	1,00418
cysK (14)	0,14693	1,82482	1,64858	0,34365	-0,19665	0,8085	0,50424	-0,11221	1,44711	0,36157	0,32721	0,0141	-0,07134
cysM (15)	2,51545	4,86738	3,74626	4,69114	0,51488	5,08407	3,86551	-0,12803	3,01287	-0,15118	0,56826	0,52792	0,78899
dadX (16)	0,10414	0,74322	0,3923	0,26596	-0,26896	0,52889	0,24467	0,62865	0,14905	0,37421	0,00773	0,06764	-0,40444
dcyD (17)	0,5261	0,24962	2,14512	-0,13773	-0,2257	1,03523	0,82	-0,19436	-0,16201	1,8998	0,0875	0,11217	4,9848
dsdA (18)	0,91984	0,30446	1,42757	0,88665	-0,29641	0,14592	-0,79822	-0,86328	0,21295	-0,16604	-0,05156	-0,06331	0,40213
epmB (19)	--	--	-0,697	--	--	--	--	--	--	--	--	--	2,75357
gabT (20)	0,09015	-0,17502	0,15882	-0,02069	0,03134	0,00803	0,135	0,08386	0,10654	0,16552	0,11	0,17059	0,05731
gadA (21)	0,13219	0,09329	-0,0712	0,12221	0,18505	-0,02751	0,17603	0,13495	0,34897	0,19193	-0,01579	0,07403	0,03967
gadB (22)	0,01455	-0,039	-0,0829	0,08795	0,11552	-0,04806	0,07497	0,05218	0,53805	0,09989	0,04275	-0,13242	-0,11466
gcvP (23)	0,07205	-0,19994	-0,04263	-0,03179	-0,1172	-0,21718	0,14982	0,18308	-0,22401	-0,0506	0,05765	0,07872	0,24034
glgP (24)	0,14908	0,92874	-0,1162	0,23985	0,0606	0,23601	0,01243	0,28473	-0,13325	0,25759	0,19642	0,17647	0,29828
glyA (25)	0,2051	2,12463	0,82052	1,60491	0,38351	1,78881	2,13055	0,17336	0,96142	0,25952	0,03251	0,04626	0,07132
hemL (26)	0,11152	-0,30588	-0,01222	-0,37113	-0,02883	-0,3982	-0,37543	-0,07381	-0,33051	0,19502	0,00922	0,05712	0,12743
hisC (27)	-0,04189	0,70694	0,2531	0,20994	0,00851	0,20851	0,13756	-0,0727	2,67599	0,99654	0,08803	-0,01695	0,47907
ilvA (28)	0,86356	2,56197	1,33491	0,56674	0,55725	1,27395	0,59603	0,1066	0,18587	0,76776	-0,07721	0,01865	0,23703
ilvE (29)	0,04728	0,8116	1,07396	0,86586	0,30926	0,52982	0,92924	0,04558	0,93287	0,56776	-0,00343	-0,037	1,17442
iscS (30)	0,81009	0,63837	1,26438	0,24885	0,3231	0,78506	0,47028	0,16016	0,20431	2,16938	0,06846	0,33864	0,4292
kbl (31)	0,70923	0,28807	1,30885	-0,06667	-0,11578	2,26237	0,1067	-0,23509	-0,26323	-0,05284	-0,23107	-0,34587	-0,14731
ldcC (32)	1,51926	6,22528	3,08163	6,67522	3,48656	5,88863	6,4106	1,05398	5,8537	1,33254	0,80595	0,33171	1,757
ltaE (33)	-0,25659	0,10068	-0,00275	-0,09985	-0,17301	-0,03406	-0,19332	-0,05834	-0,01091	-0,02623	0,1088	0,06909	-0,16069
lysA (34)	0,20636	4,78546	2,24716	1,62301	0,14356	2,08308	1,37017	0,08026	1,17941	-0,0785	-0,09748	-0,18021	0,00457
malP (35)	0,09185	0,74847	-0,04988	0,33364	0,27843	0,41273	0,1584	0,03927	0,16638	0,1006	0,27335	0,04885	0,05646
malY (36)	2,32845	3,30828	0,84771	--	1,50544	3,72664	0,38362	--	1,20154	0,60523	--	0,55577	3,34993
metB (37)	0,38925	4,84031	0,31856	0,63813	0,08622	2,32491	2,8757	0,09864	-0,20054	5,95928	0,57487	0,41756	0,55484
metC (38)	0,78562	2,26592	1,21685	1,99551	3,55889	2,14862	3,25607	0,87641	1,54293	5,17224	0,86423	0,76817	1,86785
pabC (39)	--	0,74603	1,86694	-0,51456	--	-0,04015	--	--	--	--	--	--	--
patA (40)	--	--	--	--	--	--	--	--	--	--	--	--	--
puuE (41)	-0,40892	-0,3845	0,19141	-0,6352	-0,54179	-0,27135	-0,83494	0,00313	-0,59221	-0,18773	-0,12992	-0,07783	-0,31806
selA (42)	0,94652	5,93252	1,87896	5,46203	2,79057	5,2626	4,86837	-0,19403	5,02598	3,90719	1,23625	1,25929	3,85745
serC (43)	0,63612	1,42404	0,9872	0,20112	0,02031	0,07001	0,26006	-0,01894	0,19872	1,51399	0,00566	0,08732	0,03444
speA (44)	-0,07461	0,54063	-0,04963	2,80382	0,9368	0,6822	3,28603	0,02142	-0,3741	-0,10645	-0,07822	-0,09224	0,8448
speC (45)	-0,06077	-0,04048	0,07583	-0,00479	-0,3052	-0,18735	-0,02844	0,2123	0,27154	0,10611	0,12449	0,00426	0,20385
speF (46)	--	--	--	--	--	--	--	--	--	--	--	--	--
sufS (47)	0,38689	1,0337	1,51751	-0,28198	0,09508	-0,22784	-0,13155	0,03237	-0,46951	0,03655	0,12717	0,01412	0,14103
tdcB (48)	0,32165	-0,72168	--	-0,19115	0,48518	-1,56231	-0,51108	-1,3492	-1,02081	-0,42225	-0,0754	-0,35293	-0,8989
tdcF (49)	-0,2919	0,2162	0,62266	0,19704	0,18292	0,24959	-0,09047	-0,1468	-0,48337	-0,54494	-0,56514	-0,63949	-0,26278
thrC (50)	0,3569	0,10776	6,67365	0,65027	1,18091	-0,00746	0,29842	-0,02331	0,43295	-0,0833	0,16448	-0,13199	0,07431

**tnaA (51)**	-0,02476	0,30762	0,27331	0,79921	1,23932	2,69557	1,15177	-0,03325	0,52247	0,16146	0,14665	0,09885	0,29532
**trpB (52)**	0,24009	5,56203	2,44409	1,83246	1,22585	1,01478	1,82819	-0,02474	2,47655	2,52427	0,02513	-0,11815	1,45314
**tyrB (53)**	0,10598	1,89819	2,82859	1,30526	0,37095	1,48013	1,24514	0,33821	3,34635	0,42197	0,111	0,17127	3,5521
**wecE (54)**	0,14297	1,86216	2,72861	-0,62638	0,55494	1,80855	2,65679	0,48061	2,80288	3,39486	1,49007	0,66464	1,30259
**ybdL (55)**	1,19739	0,61846	0,31913	--	0,95921	1,12839	0,33693	--	--	5,12839	0,75904	-0,44133	0,19442
**ycbX* (56)**	-0,07375	-0,07185	0,01966	-0,26851	-0,12191	-0,18387	6,19E-04	-0,09431	-0,38147	0,23133	0,2309	0,31761	0,38056
**ydcR* (57)**	--	--	--	--	--	--	--	--	1,87603	3,48933	--	--	--
**ygeX (58)**	--	--	--	--	--	--	--	--	--	--	--	--	--
**yggS (59)**	0,60256	7,68042	2,47062	7,17712	5,92414	8,40229	8,36773	3,0087	8,30433	2,19379	1,12036	0,60896	5,498
**yhfS* (60)**	--	--	--	--	--	--	--	--	--	--	--	--	--
**yhfX* (61)**	--	--	--	--	--	--	--	--	--	--	--	--	--
**yiiM* (62)**	-0,26223	-0,15132	0,01247	0,36017	-0,20599	0,21857	0,60709	-0,71043	0,0519	-0,36917	-0,32284	-0,37464	-0,13935
**yjiR* (63)**	--	--	--	--	--	--	--	--	--	--	--	--	--

Table SI 3: log_2 enrichment (probe/DMSO) values for enrichment in *P. aeruginosa* PAO1 wt. Putative, uncharacterized or poorly characterized **PLP**-dependent enzymes are highlighted with a *.

	**PL1**	**PL2**	**PL3**	**PL6**	**PL10**	**PL13**
**alr (1)**	-0,16815	-0,66912	0,00101	0,03059	-0,64842	-0,58481
**arnB (2)**	--	3,27877	3,23667	1,81675	3,09822	--
**aruC (3)**	0,01932	-0,43569	-0,10567	-0,13914	-0,16901	-0,02996
**aruH (4)**	0,0596	0,92124	0,32619	0,27009	3,70337	0,83621
**aspC (5)**	-0,10748	1,22951	0,22697	-0,04356	0,98695	-0,10197
**bauA (6)**	-0,76929	-0,92811	-0,18488	-0,29373	-0,29602	-0,35666
**bioA (7)**	-0,01476	3,15613	0,51835	1,35033	3,87506	0,22646
**cobC (8)**	--	-0,8944	-0,2112	0,29277	--	--
**csd (9)**	-0,05418	-1,00397	0,39407	-0,24724	-0,26143	-0,02942
**cysK (10)**	0,0563	-0,1456	0,04647	-0,09225	0,16138	0,04843
**cysM (11)**	-0,07683	0,22401	0,20868	-0,06056	-0,42662	-0,052
**dadX (12)**	1,0176	4,53438	1,12851	1,95851	0,09589	0,32168
**davT (13)**	-0,15201	0,235	0,02809	-0,18007	-0,14998	-0,09815
**dsdA (14)**	0,03316	-0,4136	-0,03529	-0,0997	-0,218	-0,03632
**gcvP1 (15)**	-0,02184	-1,52035	0,0448	-0,11307	-0,47126	-0,01963
**gcvP2 (16)**	0,1956	-1,86268	-0,29068	0,1984	-0,10745	-0,49451
**glgP (17)**	--	-0,00889	--	0,33352	--	--
**glyA1 (18)**	-0,41106	4,34592	-0,00714	1,29759	1,64053	-0,20341
**glyA2 (19)**	0,04714	2,94149	0,22602	0,56561	0,9026	-0,03773
**hemL (20)**	-0,08616	-1,05592	0,05933	-0,33539	-0,34011	-0,08064
**hisC1 (21)**	0,19946	1,32696	-0,03684	0,04454	0,43291	0,13262
**hisC2 (22)**	--	--	--	--	--	--
**ilvA1 (23)**	-0,01289	1,03281	-0,01432	-0,04007	0,09776	0,01839
**ilvA2 (24)**	--	3,32546	--	--	--	3,15457
**ilvE (25)**	-0,02494	3,34234	0,91617	1,61583	0,06765	0,2092
**iscS (26)**	-0,1474	1,41719	0,40887	0,38238	0,50429	-0,15443
**kynU (27)**	-0,04626	-0,88817	-0,08224	-0,31845	-0,1472	-0,08308
**ldcA (28)**	-0,03118	2,85493	0,73031	2,37394	-0,19648	-0,01916
**ltaE (29)**	0,31529	0,01862	0,14549	0,01814	-0,33108	0,1929
**lysA (30)**	0,11806	4,05411	0,48495	0,63975	0,57108	0,07157
**metY (31)**	0,00415	1,88927	0,18785	-0,10065	1,12687	-0,04386
**metZ (32)**	-0,07313	3,48521	0,12321	0,7971	3,20468	0,40957
**PA0221* (33)**	--	--	--	--	--	--
**PA0268* (34)**	--	--	--	--	--	--

**PA0394 (35)**	1,74479	7,88023	1,90571	7,70313	2,2749	3,79348
**PA0399* (36)**	-0,16847	2,055	1,14543	0,13523	2,36552	0,0334
**PA0400* (37)**	-0,03986	3,68675	0,32593	0,24082	3,01691	0,23155
**PA0529* (38)**	--	--	--	--	--	--
**PA0530* (39)**	--	--	--	--	--	--
**PA0813* (40)**	--	--	--	--	-0,54108	--
**PA0851* (41)**	0,6207	2,0508	-0,5436	0,46907	0,90357	1,07339
**PA0902 (42)**	0,05155	-0,07439	-0,03397	-0,21151	-0,22254	0,00878
**PA1061* (43)**	-0,26964	-0,64178	0,48083	-0,22806	-0,55622	-0,14863
**PA1346* (44)**	--	--	--	--	--	--
**PA1654* (45)**	0,10465	-1,69945	-0,4161	-0,17814	-0,97283	0,22948
**PA2032* (46)**	0,4095	2,01835	0,90083	0,31811	2,33683	0,40828
**PA2062 (47)**	--	--	--	--	--	--
**PA2100* (48)**	--	2,21657	--	--	2,70976	--
**PA2104* (49)**	--	--	--	--	--	--
**PA2229* (50)**	--	--	--	--	--	--
**PA2531* (51)**	--	--	--	--	--	--
**PA2683* (52)**	1,00465	1,51816	0,05199	0,61376	0,29305	0,24182
**PA2828* (53)**	-0,08198	0,87961	-0,00274	-0,07697	-0,03392	0,09776
**PA2897* (54)**	--	--	--	--	3,32392	--
**PA2958* (55)**	1,30304	-0,51847	0,13924	-0,21715	4,47515	0,38595
**PA3022* (56)**	-0,15375	1,23315	0,20113	0,18334	-0,05398	0,40938
**PA3659* (57)**	0,13102	3,96187	0,13903	1,12221	0,83921	0,2585
**PA3798* (58)**	-0,02926	-0,54025	0,0402	-0,05767	1,40585	-0,02507
**PA4088* (59)**	--	--	--	--	--	--
**PA4132 (60)**	-0,04194	-0,38379	-0,24826	-0,13591	0,14489	0,02965
**PA4165* (61)**	--	--	--	--	--	--
**PA4536* (62)**	--	--	--	--	--	--
**PA4715* (63)**	-0,10224	3,45152	0,62657	2,28252	0,1871	-0,08757
**PA4722 (64)**	-0,22994	-1,44068	0,0092	-0,44762	-0,49604	-0,08079
**PA4805* (65)**	--	--	--	--	--	--
**PA4875* (66)**	--	--	--	--	--	--
**PA5283* (67)**	--	--	--	--	3,80607	--
**PA5313* (68)**	0,15271	0,74454	0,00118	0,37663	2,66955	0,62165
**PA5431* (69)**	--	--	--	--	--	--
**PA5523* (70)**	0,06097	-2,02042	0,18645	-0,73592	-0,19936	0,15453
**pabC (71)**	--	1,86076	--	--	--	--
**phhC (72)**	-0,0572	1,61292	-0,01181	0,15731	0,67934	0,02572
**phnW (73)**	--	--	--	--	1,94571	--
**pvdH (74)**	--	--	--	--	--	--
**pvdN (75)**	-0,01682	-1,18374	-0,21261	-0,19103	-0,47165	-0,07739
**selA (76)**	2,85092	9,17781	2,68217	7,73476	5,7718	1,90309
**serC (77)**	0,11834	1,16653	0,23289	-0,00196	0,41291	-0,09932
**speA (78)**	0,03074	0,32425	0,22166	1,27054	0,63761	0,03222
**speC (79)**	-0,08091	3,57223	0,48798	1,01293	0,22675	0,05431
**spuC (80)**	-0,04864	2,76178	0,24016	0,73384	0,31613	0,08904
**thrC (81)**	-0,0357	2,61586	1,98533	-0,13058	0,28814	-0,04355
**trpB (82)**	-0,12115	4,9452	1,39072	0,6838	1,57185	-0,13414
**wbpE (83)**	0,2145	3,95289	0,53482	3,30369	2,70911	0,61301

Table SI 4: List of screen-compounds and corresponding literature.

	Compound	Structure	MW	Source	Literature	Solubility
1	EN300-213294	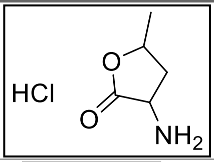	151.59	Enamine		H_2O
2	EN300-99743	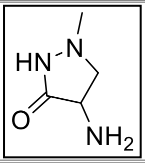	115.14	Enamine		H_2O
3	EN300-6489485	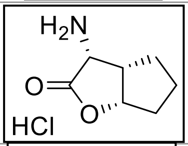	177.63	Enamine		H_2O
4	EN300-219172	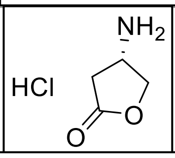	137.56	Enamine		H_2O
5	EN300-17366	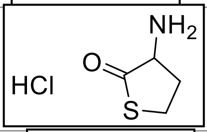	153.62	Enamine		H_2O
6	EN300-7427659	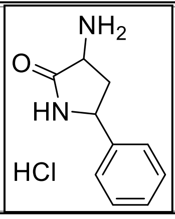	212.68	Enamine		H_2O
7	EN300-6735762	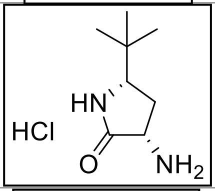	192.68	Enamine		H_2O
8	EN300-298184	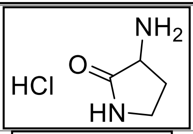	136.58	Enamine		H_2O
9	EN300-7445633	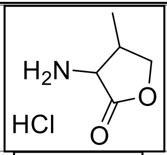	151.59	Enamine		H_2O
10	BBV-38269949	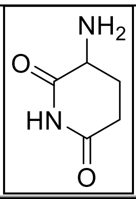	128.13	Enamine		H_2O
11	EN300-17331	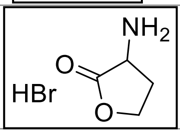	182.02	Enamine		H_2O

**12**	EN300-10694	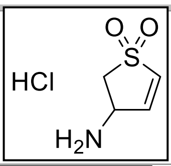	169.62	Enamine		H_2O
**13**	EN300-127487	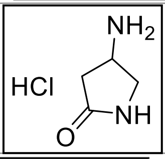	136.58	Enamine		H_2O
**14**	Isoniazid	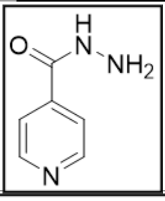	137.14	Sigma	[8]	H_2O
**15**	Mimosin	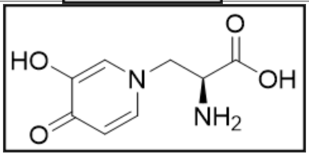	198.18	Sigma	[9]	50 mM NaOH
**16**	Gabaculin	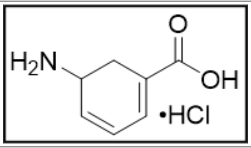	175.61	Cayman	[10,11]	DMSO
**17**	CCG-50014	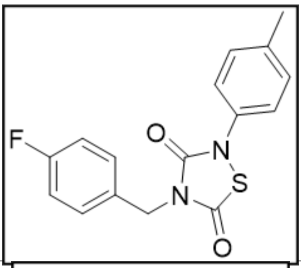	316.35	Sigma	[12]	DMSO
**18**	Alafosfalin	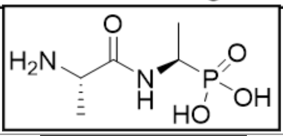	196.14	Carbosynth	[13]	H_2O
**19**	β-Chloro-L-alanine	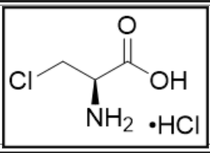	160	Carbosynth	[11,13]	H_2O
**20**	TM-2-10	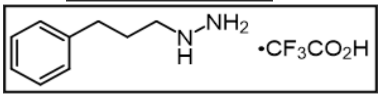	264.25	Synthesis		DMSO
**21**	Carbidopa	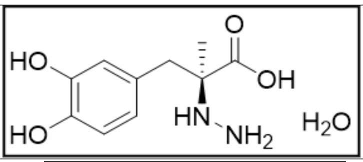	244.24	Arcos	[11]	DMSO
**22**	Aminoadipic acid	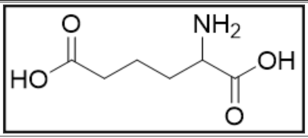	161.16	Sigma	[11,14]	DMSO
**23**	Penicillamine	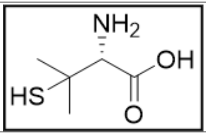	149.21	Sigma	[11]	H_2O

24	Ginkgotoxin	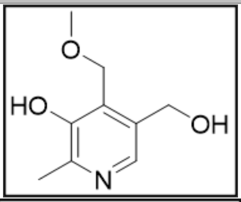	183.21	Sigma	[15]	DMSO
25	Ethanolamin-O-sulfat	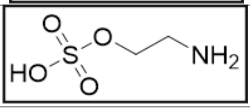	141.14	TCI	[11]	H_2O
26	Gabapentin	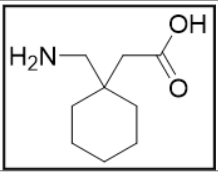	171.24	TCI	[11]	H_2O
27	O-Allyl hydroxyl amine	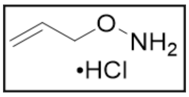	109.55	TCI	[11]	H_2O
28	Quisqualic acid	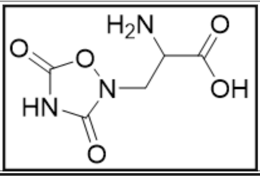	189.13	Sigma	[11,14]	50 mM NaOH
29	Benserazide	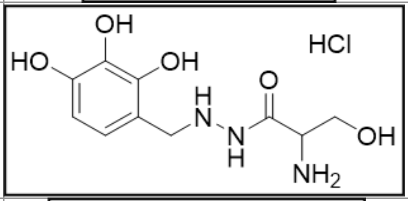	293.7	TCI	[11]	H_2O
30	Eflornithine	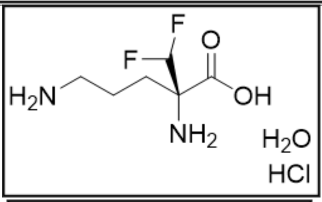	236.64	TCI	[11]	H_2O
31	Indole-3-acetyl alanine	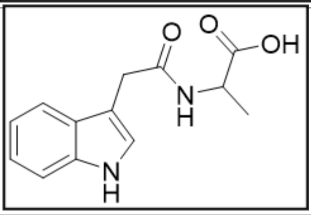	246.27	Sigma	[11,16]	DMSO
32	Canavanine	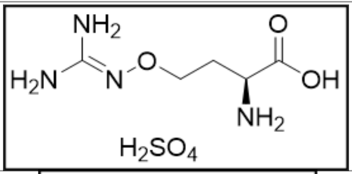	274.25	Sigma	[11,17]	H_2O
33	Canaline	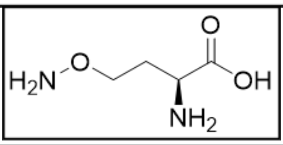	134.14	Biomol	[11,17]	H_2O

**34**	Trifluoralanine	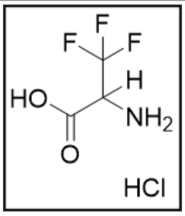	179.53	Sigma	[11]	H_2O
**35**	2-Aminobenzene sulfonate	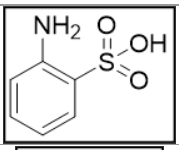	173.19	TCI	[11]	DMSO
**36**	Gyromitrin	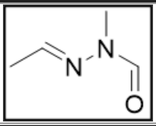	100.12	Toronto	[18]	H_2O
**37**	Indole-3-acetyl glutamate	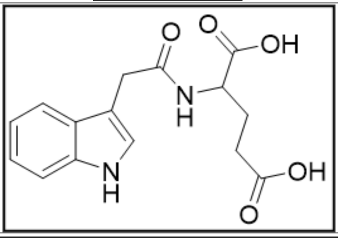	304.3	Toronto	[11,16]	DMSO
**38**	TM-2-11	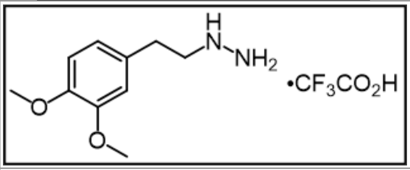	310.27	Synthesis		DMSO
**39**	Phenelzine	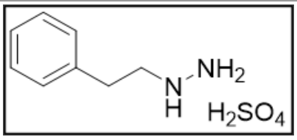	234.27	Carbosynth	[11]	H_2O
**40**	Aminoethylvinyl glycine	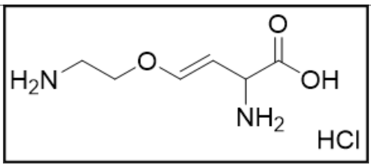	196.63	Carbosynth	[11]	H_2O
**41**	Propargylglycine	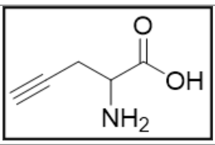	113.12	Carbosynth	[11]	H_2O
**42**	MAC173979	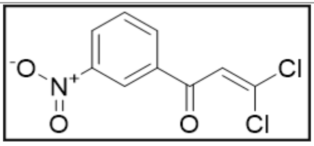	246.04	Synthesis	[19]	DMSO
**43**	TM-2-12	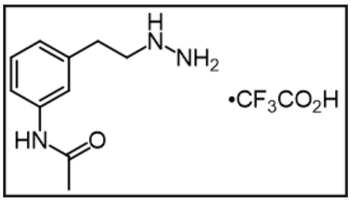	307.27	Synthesis		DMSO

**44**	TM-2-13	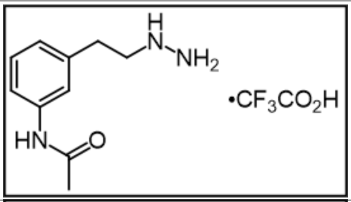	322.33	Synthesis		DMSO
**45**	TM-2-14	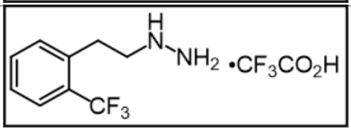	318.22	Synthesis		DMSO
**46**	PL15	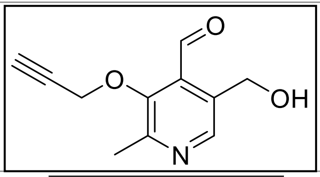	205.21	Synthesis		DMSO
**47**	PL-Methoxim	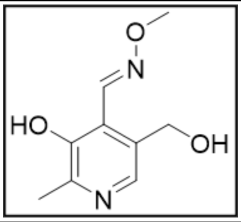	196.21	Synthesis		DMSO
**48**	EN300-295489	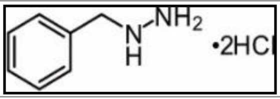	195.09	Enamine		DMSO
**49**	TM-2-15	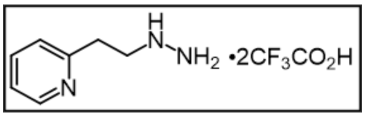	365.23	Synthesis		DMSO
**50**	TM-2-16	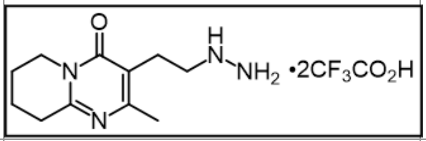	450.34	Synthesis		DMSO
**51**	TM-2-17	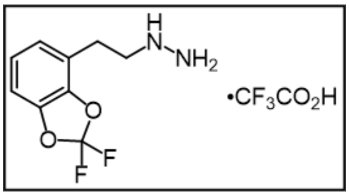	330.21	Synthesis		DMSO
**52**	TM-2-18	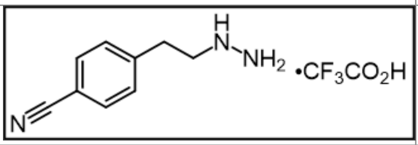	275.23	Synthesis		DMSO
**53**	TM-2-19	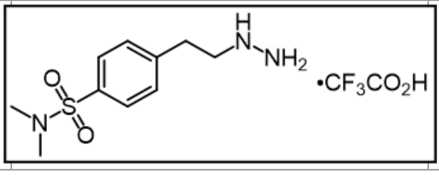	375.35	Synthesis		DMSO

Table SI 5: MIC values of screening compounds.

Strain	Hit#	MIC	Structure	Name
**S. aureus USA300**	17	50 µM	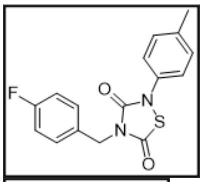	CCG-50014
	19	500 µM	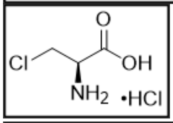	beta-Chloroalanine
	21	100 µM	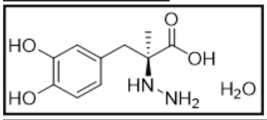	Carbidopa
	29	50 µM	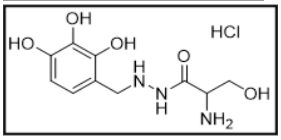	Benserazide
	39	100 µM	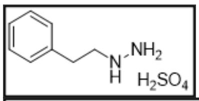	Phenelzine
	42	250 µM	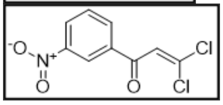	MAC173979
**E. coli K12**	29	500 µM	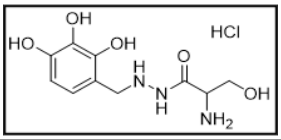	Benserazide

Table SI 6: Phenelzine analogues tested in *S. aureus* USA300 and *P. aeruginosa* PAO1.

Strain	Hit#	MIC	Structure	Name
**S. aureus USA300**	48	500 µM	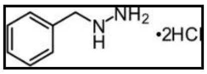	EN300-295489
	20	250 µM	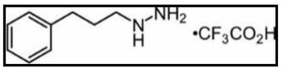	TM-2-10
	38	-	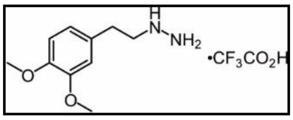	TM-2-11
	43	500 µM	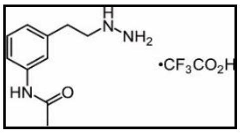	TM-2-12
	44	250 µM	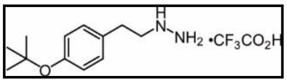	TM-2-13

	45	250 µM	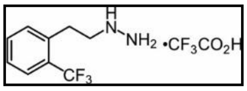	TM-2-14
	49	250 µM	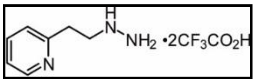	TM-2-15
	50	250 µM	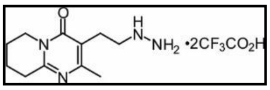	TM-2-16
	51	250 µM	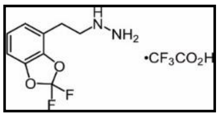	TM-2-17
	52	-	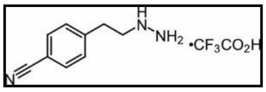	TM-2-18
	53	500 µM	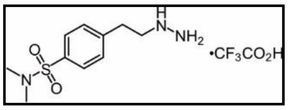	TM-2-19
**P. aeruginosa PAO1**	20	500 µM	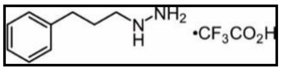	TM-2-10
	49	250 µM	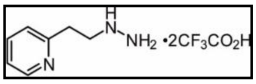	TM-2-15
	50	250 µM	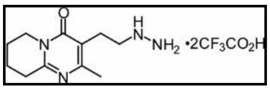	TM-2-16

Table SI 7: Raw data table for competitive ABPP experiments in *S. aureus* USA300 TnpdxS using 100 µM **PL2** and 75 µM phenelzine.

Uniprot Code	PLP-DE	Essential	PL2 100 µM Enrichment			Phenelzine 75 µM		
			**-Log Student's T-test p-value**	**Student's T-test Difference**	**Signficant**	**-Log Student's T-test p-value**	**Student's T-test Difference**	**Competition**
**A0A0H2XFB2**	+		2,911915	4,792589	+	2,798515	-4,62205	+
**Q2FFN1**	+		2,32953	3,058241	+	2,512637	-1,10572	+
**Q2FHT1**	+	+	1,590619	0,623123		1,967897	-1,06337	+
**A0A0H2XFA8**	+		3,635831	5,466553	+	1,957437	-0,83536	
**A0A0H2XFD9**	+		3,677046	4,473566	+	0,986374	-0,32885	
**A0A0H2XFF8**	+		2,899135	5,802236	+	0,908677	-0,37751	
**A0A0H2XFH9**	+		4,403541	4,001843	+	0,008181	-0,0055	
**A0A0H2XFQ3**	+		3,747202	2,874456	+	3,142535	0,970811	
**A0A0H2XFY9**	+		5,185631	7,17599	+	0,860133	-0,12571	
**A0A0H2XG37**	+		4,892505	4,379363	+	0,225327	-0,13834	

**A0A0H2XH24**	+		3,388182	3,33555	+	0,487064	0,11434	
**A0A0H2XHH8**	+		2,999274	3,31852	+	0,418216	0,187763	
**A0A0H2XHJ5**	+	+	2,42586	3,345207	+	0,241653	-0,21744	
**A0A0H2XHU6**	+		4,470736	3,513389	+	0,13163	-0,04218	
**A0A0H2XHV8**	+		5,444886	5,382275	+	2,148487	-0,8027	
**A0A0H2XII6**	+		2,789785	4,459506	+	1,153096	0,252381	
**A0A0H2XIK4**	+		3,131095	1,148045	+	0,116128	0,039349	
**A0A0H2XIS2**	+		3,911165	4,487909	+	1,920238	-0,27663	
**A0A0H2XIV3**	+		1,486455	2,071847	+	0,364	-0,26882	
**A0A0H2XJX6**	+		4,074725	3,853058	+	0,338383	-0,10145	
**A0A0H2XKI8**	+	+	1,432664	2,213551	+	0,493586	0,839298	
**Q2FF15**	+		3,262992	1,170604	+	0,492584	0,145259	
**Q2FF55**	+	+	2,759966	3,146412	+	0,119931	0,070024	
**Q2FF63**	+		3,076005	2,04505	+	0,849205	-0,88629	
**Q2FGI7**	+		4,01688	2,093948	+	0,900531	0,114589	
**Q2FH01**	+		2,569993	2,711582	+	0,549419	-0,42322	
**Q2FH64**	+		1,300518	1,67535	+	1,753522	0,992339	
**Q2FIR7**	+		2,917078	3,329039	+	1,614265	-0,48723	
**A0A0H2XGZ7**	+		0,829818	0,851405		0,742324	-0,61203	
**A0A0H2XI95**	+		0,581927	1,995541		0,394843	-0,10124	
**A0A0H2XJK0**	+		0,300259	-0,1131		0,331931	0,151664	
**A0A0H2XJW8**	+		2,406646	0,841398		1,511149	0,515059	
**Q2FG69**	+		0,757769	0,242012		0,164337	0,096378	
**Q2FGI6**	+		0,676772	-0,31141		1,117523	0,262202	

Table SI 8: Raw data table for competitive ABPP experiments in *S. aureus* USA300 TnpdxS using 100 µM **PL2** and 100 µM phenelzine.

Uniprot Code	PLP-DE	Essential	PL2 100 µM Enrichment			Phenelzine 100 µM		
			**-Log Student's T-test p-value**	**Student's T-test Difference**	**Significant**	**-Log Student's T-test p-value**	**Student's T-test Difference**	**Competition**
**A0A0H2XFB2**	+		2,911915	4,792589	+	4,934767	-4,31144	+
**Q2FFN1**	+		2,32953	3,058241	+	3,101358	-1,50988	+
**Q2FHT1**	+	+	1,590619	0,623123		2,595021	-1,00723	+
**A0A0H2XFA8**	+		3,635831	5,466553	+	3,126513	-0,93849	
**A0A0H2XFD9**	+		3,677046	4,473566	+	1,542401	-0,29392	
**A0A0H2XFF8**	+		2,899135	5,802236	+	0,896902	-0,4678	
**A0A0H2XFH9**	+		4,403541	4,001843	+	0,250881	-0,10828	
**A0A0H2XFQ3**	+		3,747202	2,874456	+	2,21485	0,903922	
**A0A0H2XFY9**	+		5,185631	7,17599	+	1,239692	-0,34416	
**A0A0H2XG37**	+		4,892505	4,379363	+	0,320409	-0,15807	
**A0A0H2XH24**	+		3,388182	3,33555	+	0,244781	-0,16212	
**A0A0H2XHH8**	+		2,999274	3,31852	+	0,342396	0,199385	
**A0A0H2XHJ5**	+	+	2,42586	3,345207	+	0,113997	-0,12138	
**A0A0H2XHU6**	+		4,470736	3,513389	+	0,47089	-0,19079	
**A0A0H2XHV8**	+		5,444886	5,382275	+	2,556003	-0,98865	

**A0A0H2XII6**	+		2,789785	4,459506	+	0,37263	0,32258	
**A0A0H2XIK4**	+		3,131095	1,148045	+	0,035069	-0,02188	
**A0A0H2XIS2**	+		3,911165	4,487909	+	1,211049	-0,7486	
**A0A0H2XIV3**	+		1,486455	2,071847	+	0,003492	0,003426	
**A0A0H2XJX6**	+		4,074725	3,853058	+	0,451162	-0,16639	
**A0A0H2XKI8**	+	+	1,432664	2,213551	+	0,564411	0,909887	
**Q2FF15**	+		3,262992	1,170604	+	0,198864	0,122362	
**Q2FF55**	+	+	2,759966	3,146412	+	0,107995	0,122477	
**Q2FF63**	+		3,076005	2,04505	+	0,96646	-1,37382	
**Q2FGI7**	+		4,01688	2,093948	+	0,248344	0,044518	
**Q2FH01**	+		2,569993	2,711582	+	0,311877	-0,23824	
**Q2FH64**	+		1,300518	1,67535	+	0,794013	0,591021	
**Q2FIR7**	+		2,917078	3,329039	+	3,390267	-0,85103	
**A0A0H2XGZ7**	+		0,829818	0,851405		0,659962	-0,86113	
**A0A0H2XI95**	+		0,581927	1,995541		0,734397	-0,1769	
**A0A0H2XJK0**	+		0,300259	-0,1131		0,303072	0,218683	
**A0A0H2XJW8**	+		2,406646	0,841398		0,51669	0,352659	
**Q2FG69**	+		0,757769	0,242012		0,256483	0,110555	
**Q2FGI6**	+		0,676772	-0,31141		2,494975	0,598986	

Table SI 9: Raw data table for competitive ABPP experiments in *S. aureus* USA300 TnpdxS using 100 µM **PL2** and 1 mM phenelzine.

Uniprot Code	PLP-DE	Essential	PL2 100 µM Enrichment			Phenelzine 1 mM		
			**-Log Student's T-test p-value**	**Student's T-test Difference**	**Significant**	**-Log Student's T-test p-value**	**Student's T-test Difference**	**Competition**
**A0A0H2XFD9**	+		3,677046	4,473566	+	4,623829	-3,74018	+
**A0A0H2XHH8**	+		2,999274	3,31852	+	1,401644	-1,5085	+
**A0A0H2XII6**	+		2,789785	4,459506	+	3,677283	-2,14159	+
**A0A0H2XIV3**	+		1,486455	2,071847	+	2,042037	-2,34272	+
**Q2FF55**	+	+	2,759966	3,146412	+	2,229908	-1,13321	+
**A0A0H2XI95**	+		0,581927	1,995541		3,243673	-1,19675	+
**A0A0H2XFA8**	+		3,635831	5,466553	+	4,445162	-4,86774	+
**A0A0H2XFB2**	+		2,911915	4,792589	+	4,5665	-5,43387	+
**A0A0H2XFF8**	+		2,899135	5,802236	+	4,094766	-2,38797	+
**A0A0H2XFH9**	+		4,403541	4,001843	+	2,656668	-2,63129	+
**A0A0H2XFY9**	+		5,185631	7,17599	+	4,404696	-3,65091	+
**A0A0H2XG37**	+		4,892505	4,379363	+	4,20669	-3,81322	+
**A0A0H2XH24**	+		3,388182	3,33555	+	3,477852	-2,93521	+
**A0A0H2XHJ5**	+	+	2,42586	3,345207	+	2,087924	-1,32709	+
**A0A0H2XHU6**	+		4,470736	3,513389	+	4,319624	-3,17976	+
**A0A0H2XHV8**	+		5,444886	5,382275	+	1,476799	-2,65147	+
**A0A0H2XIS2**	+		3,911165	4,487909	+	4,590317	-4,20052	+
**A0A0H2XJX6**	+		4,074725	3,853058	+	2,100252	-1,04894	+
**Q2FF63**	+		3,076005	2,04505	+	3,22001	-2,7116	+
**Q2FFN1**	+		2,32953	3,058241	+	2,29602	-1,77939	+

**Q2FGI7**	+		4,01688	2,093948	+	4,337881	-1,31776	+
**Q2FH01**	+		2,569993	2,711582	+	2,906787	-2,61043	+
**Q2FHT1**	+	+	1,590619	0,623123		2,586154	-1,0593	+
**Q2FIR7**	+		2,917078	3,329039	+	2,998234	-1,00035	+
**A0A0H2XFQ3**	+		3,747202	2,874456	+	2,590922	0,947496	
**A0A0H2XGZ7**	+		0,829818	0,851405		0,233245	-0,22292	
**A0A0H2XIK4**	+		3,131095	1,148045	+	2,557402	-0,76667	
**A0A0H2XJK0**	+		0,300259	-0,1131		1,41409	0,632022	
**A0A0H2XJW8**	+		2,406646	0,841398		1,472464	-0,48979	
**A0A0H2XKI8**	+	+	1,432664	2,213551	+	0,302886	-0,88723	
**Q2FF15**	+		3,262992	1,170604	+	2,218536	-0,72985	
**Q2FG69**	+		0,757769	0,242012		1,813296	0,743725	
**Q2FGI6**	+		0,676772	-0,31141		1,513221	0,828939	
**Q2FH64**	+		1,300518	1,67535	+	0,316432	-0,51713	

Table SI 10: Raw data table for competitive ABPP experiments in *S. aureus* USA300 TnpdxS using 100 µM **PL2** and 100 µM benserazide.

Uniprot Code	PLP-DE	Essential	PL2 100 µM Enrichment			Benserazide 100 µM		
			**-Log Student's T-test p-value**	**Student's T-test Difference**	**Significant**	**-Log Student's T-test p-value**	**Student's T-test Difference**	**Competition**
**A0A0H2XFA8**	+		3,635831	5,466553	+	0,895526	-0,25723	
**A0A0H2XFB2**	+		2,911915	4,792589	+	0,955869	-3,17272	
**A0A0H2XH24**	+		3,388182	3,33555	+	1,629363	-0,2787	
**A0A0H2XIS2**	+		3,911165	4,487909	+	0,192494	-0,06521	
**A0A0H2XGP0**	+		2,736212	4,26746	+	0,087183	-0,05723	
**A0A0H2XFD9**	+		3,677046	4,473566	+	0,204416	0,049622	
**A0A0H2XFF8**	+		2,899135	5,802236	+	0,204103	0,096066	
**A0A0H2XFH9**	+		4,403541	4,001843	+	0,20506	-0,05835	
**A0A0H2XFY9**	+		5,185631	7,17599	+	0,4428	-0,16423	
**A0A0H2XG37**	+		4,892505	4,379363	+	0,042764	0,025868	
**A0A0H2XHJ5**	+	+	2,42586	3,345207	+	0,296736	0,175776	
**A0A0H2XHU6**	+		4,470736	3,513389	+	0,194176	-0,0744	
**A0A0H2XHV8**	+		5,444886	5,382275	+	0,308803	-0,09124	
**A0A0H2XII6**	+		2,789785	4,459506	+	1,271189	-0,44027	
**A0A0H2XIV3**	+		1,486455	2,071847	+	0,109135	0,06868	
**A0A0H2XJX6**	+		4,074725	3,853058	+	0,035482	0,019281	
**Q2FF55**	+	+	2,759966	3,146412	+	0,148618	-0,11217	
**Q2FF63**	+		3,076005	2,04505	+	0,641368	-1,16364	
**Q2FFN1**	+		2,32953	3,058241	+	0,742836	-0,14102	
**Q2FGI7**	+		4,01688	2,093948	+	0,324782	0,087121	
**Q2FH01**	+		2,569993	2,711582	+	0,153566	-0,14925	
**Q2FIR7**	+		2,917078	3,329039	+	1,90444	-0,4921	
**A0A0H2XFQ3**	+		3,747202	2,874456	+	0,161358	-0,13297	
**A0A0H2XHH8**	+		2,999274	3,31852	+	0,19264	0,09266	
**A0A0H2XIK4**	+		3,131095	1,148045	+	0,164647	0,11725	

**A0A0H2XKI8**	+	+	1,432664	2,213551	+	0,447187	0,755213	
**Q2FF15**	+		3,262992	1,170604	+	0,375826	-0,17502	
**Q2FH64**	+		1,300518	1,67535	+	0,477135	0,360423	
**A0A0H2XGZ7**	+		0,829818	0,851405		0,267091	-0,48693	
**A0A0H2XI95**	+		0,581927	1,995541		0,013432	0,006327	
**A0A0H2XJK0**	+		0,300259	-0,1131		0,079248	-0,02328	
**A0A0H2XJW8**	+		2,406646	0,841398		0,2205	0,141897	
**Q2FG69**	+		0,757769	0,242012		0,286426	-0,1087	
**Q2FGI6**	+		0,676772	-0,31141		0,690174	0,162619	
**Q2FHT1**	+	+	1,590619	0,623123		0,676871	-0,27299	

Table SI 11: Raw data table for competitive ABPP experiments in *S. aureus* USA300 TnpdxS using 100 µM **PL2** and 1 mM benserazide.

Uniprot Code	PLP-DE	Essential	PL2 100 µM Enrichment			Benserazide 1 mM		
			**-Log Student's T-test p-value**	**Student's T-test Difference**	**Significant**	**-Log Student's T-test p-value**	**Student's T-test Difference**	**Competition**
**A0A0H2XFA8**	+		3,635831	5,466553	+	2,470979	-1,13518	+
**A0A0H2XFB2**	+		2,911915	4,792589	+	2,366294	-5,03447	+
**A0A0H2XH24**	+		3,388182	3,33555	+	2,561932	-1,32103	+
**A0A0H2XIS2**	+		3,911165	4,487909	+	2,283037	-1,17906	+
**A0A0H2XGP0**	+		2,736212	4,26746	+	1,33529	-0,53452	
**A0A0H2XFD9**	+		3,677046	4,473566	+	2,147168	-0,89133	
**A0A0H2XFF8**	+		2,899135	5,802236	+	0,032517	-0,01506	
**A0A0H2XFH9**	+		4,403541	4,001843	+	1,375273	-0,35802	
**A0A0H2XFY9**	+		5,185631	7,17599	+	3,323757	-0,72567	
**A0A0H2XG37**	+		4,892505	4,379363	+	0,470398	-0,20854	
**A0A0H2XHJ5**	+	+	2,42586	3,345207	+	0,478515	-0,69894	
**A0A0H2XHU6**	+		4,470736	3,513389	+	0,952645	-0,70264	
**A0A0H2XHV8**	+		5,444886	5,382275	+	0,690472	-0,32593	
**A0A0H2XII6**	+		2,789785	4,459506	+	0,226493	-0,22677	
**A0A0H2XIV3**	+		1,486455	2,071847	+	0,023594	-0,03392	
**A0A0H2XJX6**	+		4,074725	3,853058	+	0,337995	-0,214	
**Q2FF55**	+	+	2,759966	3,146412	+	0,170967	-0,13177	
**Q2FF63**	+		3,076005	2,04505	+	0,843274	-0,98173	
**Q2FFN1**	+		2,32953	3,058241	+	1,272951	-3,44317	
**Q2FGI7**	+		4,01688	2,093948	+	1,135419	-0,4266	
**Q2FH01**	+		2,569993	2,711582	+	0,874271	-2,19859	
**Q2FIR7**	+		2,917078	3,329039	+	2,484072	-0,86833	
**A0A0H2XFQ3**	+		3,747202	2,874456	+	0,68301	0,334218	
**A0A0H2XHH8**	+		2,999274	3,31852	+	0,295176	0,178171	
**A0A0H2XIK4**	+		3,131095	1,148045	+	0,600201	-0,41521	
**A0A0H2XKI8**	+	+	1,432664	2,213551	+	0,249006	-0,94149	
**Q2FF15**	+		3,262992	1,170604	+	1,999214	-0,48374	
**Q2FH64**	+		1,300518	1,67535	+	1,090204	0,638929	
**A0A0H2XGZ7**	+		0,829818	0,851405		0,696722	-0,83774	

**A0A0H2XI95**	+		0,581927	1,995541		0,127985	0,098735	
**A0A0H2XJK0**	+		0,300259	-0,1131		0,086692	0,073565	
**A0A0H2XJW8**	+		2,406646	0,841398		0,990936	0,279184	
**Q2FG69**	+		0,757769	0,242012		2,550267	-0,51162	
**Q2FGI6**	+		0,676772	-0,31141		0,207312	0,0881	
**Q2FHT1**	+	+	1,590619	0,623123		1,102742	-0,67609	

Table SI 12: Raw data table for competitive ABPP experiments in *S. aureus* USA300 TnpdxS using 100 µM **PL2** and 10 mM benserazide.

Uniprot Code	PLP-DE	Essential	PL2 100 µM Enrichment			Benserazide 10 mM		
			**-Log Student's T-test p-value**	**Student's T-test Difference**	**Significant**	**-Log Student's T-test p-value**	**Student's T-test Difference**	**Competition**
**A0A0H2XFA8**	+		3,635831	5,466553	+	4,866707	-4,26054	+
**A0A0H2XFB2**	+		2,911915	4,792589	+	3,199339	-5,12119	+
**A0A0H2XH24**	+		3,388182	3,33555	+	5,033894	-2,63146	+
**A0A0H2XIS2**	+		3,911165	4,487909	+	6,710828	-3,78237	+
**A0A0H2XGP0**	+		2,736212	4,26746	+	3,704917	-2,85911	+
**A0A0H2XFD9**	+		3,677046	4,473566	+	5,076107	-2,69074	+
**A0A0H2XFF8**	+		2,899135	5,802236	+	2,5289	-1,33943	+
**A0A0H2XFH9**	+		4,403541	4,001843	+	3,538034	-2,25355	+
**A0A0H2XFY9**	+		5,185631	7,17599	+	3,121432	-4,55206	+
**A0A0H2XG37**	+		4,892505	4,379363	+	4,482309	-2,86537	+
**A0A0H2XHJ5**	+	+	2,42586	3,345207	+	1,801593	-1,05695	+
**A0A0H2XHU6**	+		4,470736	3,513389	+	3,889967	-2,29852	+
**A0A0H2XHV8**	+		5,444886	5,382275	+	2,073947	-4,01032	+
**A0A0H2XII6**	+		2,789785	4,459506	+	3,802666	-2,40297	+
**A0A0H2XIV3**	+		1,486455	2,071847	+	3,188204	-2,63813	+
**A0A0H2XJX6**	+		4,074725	3,853058	+	2,324249	-1,53929	+
**Q2FF55**	+	+	2,759966	3,146412	+	1,542142	-1,00257	+
**Q2FF63**	+		3,076005	2,04505	+	2,159617	-2,18351	+
**Q2FFN1**	+		2,32953	3,058241	+	1,839155	-3,45096	+
**Q2FGI7**	+		4,01688	2,093948	+	4,115856	-1,7738	+
**Q2FH01**	+		2,569993	2,711582	+	1,673763	-2,77992	+
**Q2FIR7**	+		2,917078	3,329039	+	3,408524	-2,603	+
**A0A0H2XFQ3**	+		3,747202	2,874456	+	1,067622	0,441427	
**A0A0H2XHH8**	+		2,999274	3,31852	+	0,455117	0,231129	
**A0A0H2XIK4**	+		3,131095	1,148045	+	2,090674	-0,73293	
**A0A0H2XKI8**	+	+	1,432664	2,213551	+	0,470499	-1,25959	
**Q2FF15**	+		3,262992	1,170604	+	3,151397	-0,8837	
**Q2FH64**	+		1,300518	1,67535	+	2,221637	0,83236	
**A0A0H2XGZ7**	+		0,829818	0,851405		0,869057	-0,7335	
**A0A0H2XI95**	+		0,581927	1,995541		0,750125	-0,24017	
**A0A0H2XJK0**	+		0,300259	-0,1131		0,081568	-0,02925	
**A0A0H2XJW8**	+		2,406646	0,841398		0,608855	-0,26229	
**Q2FG69**	+		0,757769	0,242012		2,759281	-0,69383	

**Q2FGI6**	+		0,676772	-0,31141		0,292794	0,084096	
**Q2FHT1**	+	+	1,590619	0,623123		1,909392	-0,74902	

Table SI 13: Raw data table for competitive ABPP experiments in *S. aureus* USA300 TnpdxS using 100 µM **PL2** and 100 µM CCG-50014.

Uniprot Code	PLP-DE	Essential	PL2 100 µM Enrichment			CCG-50014 100 µM		
			**-Log Student's T-test p-value**	**Student's T-test Difference**	**Significant**	**-Log Student's T-test p-value**	**Student's T-test Difference**	**Competition**
**A0A0H2XFH9**	+		3,917541	4,501469	+	1,043633	-0,97873	
**Q2FIR7**	+		2,887177	2,325665	+	0,820074	-1,08965	
**A0A0H2XFD9**	+		3,728585	5,513123	+	0,554405	-0,48494	
**A0A0H2XFY9**	+		4,87032	4,982655	+	1,335816	-0,40965	
**A0A0H2XHH8**	+		2,422	8,045567	+	1,120834	-1,06612	
**A0A0H2XHJ5**	+	+	2,774298	4,140401	+	0,954516	-1,23077	
**A0A0H2XIS2**	+		4,073272	4,848108	+	0,759098	-0,52584	
**A0A0H2XKI8**	+	+	2,735126	2,097936	+	0,413969	-1,07442	
**A0A0H2XGP0**	+		3,492231	6,89933	+	0,674047	-0,61194	
**A0A0H2XFA8**	+		3,67963	4,224853	+	0,998604	-0,32922	
**A0A0H2XFQ3**	+		1,882728	2,495381	+	0,718727	0,859717	
**A0A0H2XG37**	+		4,911101	6,435907	+	0,995271	-0,58568	
**A0A0H2XH24**	+		2,987767	3,5633	+	0,485596	-0,29492	
**A0A0H2XHU6**	+		3,434236	4,114889	+	0,809211	-0,51667	
**A0A0H2XHV8**	+		3,148696	4,066203	+	0,845775	-0,61854	
**A0A0H2XII6**	+		3,538899	4,810197	+	0,773747	-0,36984	
**A0A0H2XJX6**	+		2,766526	2,080034	+	0,004006	-0,00336	
**Q2FF55**	+	+	3,025561	2,028389	+	0,684782	-0,43101	
**Q2FGI7**	+		3,598635	1,451049	+	0,467144	-0,1485	
**A0A0H2XFF8**	+		1,021824	3,437964		0,521625	-0,88605	
**A0A0H2XI95**	+		0,947552	2,46362		1,623785	-0,48449	
**A0A0H2XIK4**	+		1,894125	0,585261		0,78792	-0,22125	
**A0A0H2XIV3**	+		0,214506	-0,16295		2,269605	0,70528	
**A0A0H2XJK0**	+		0,591483	0,177837		0,223244	0,084571	
**A0A0H2XJW8**	+		1,752239	0,698915		0,185154	0,114084	
**Q2FF15**	+		2,977222	0,945261		0,114792	-0,05049	
**Q2FFN1**	+		1,939537	0,961027		0,024754	-0,02412	
**Q2FG69**	+		0,343236	0,10349		0,119937	0,048669	
**Q2FGI6**	+		1,121005	0,4761		0,115564	-0,0897	
**Q2FHT1**	+	+	0,085824	-0,08424		0,006448	-0,00778	

Table SI 14: Raw data table for competitive ABPP experiments in *S. aureus* USA300 TnpdxS using 100 µM **PL2** and 500 µM CCG-50014.

Uniprot Code	PLP-DE	Essential	PL2 100 µM Enrichment			CCG-50014 500 µM		
			**-Log Student's T-test p-value**	**Student's T-test Difference**	**Significant**	**-Log Student's T-test p-value**	**Student's T-test Difference**	**Competition**
**A0A0H2XFH9**	+		3,917541	4,501469	+	1,329666	-1,20455	+
**Q2FIR7**	+		2,887177	2,325665	+	1,204366	-0,458	

**A0A0H2XFD9**	+		3,728585	5,513123	+	0,780246	-0,44465	
**A0A0H2XFY9**	+		4,87032	4,982655	+	1,311915	-0,28419	
**A0A0H2XHH8**	+		2,422	8,045567	+	0,899143	-0,98816	
**A0A0H2XHJ5**	+	+	2,774298	4,140401	+	1,43479	-0,78127	
**A0A0H2XIS2**	+		4,073272	4,848108	+	0,876995	-0,4885	
**A0A0H2XKI8**	+	+	2,735126	2,097936	+	0,243526	-0,28901	
**A0A0H2XGP0**	+		3,492231	6,89933	+	1,093276	-0,73599	
**A0A0H2XFA8**	+		3,67963	4,224853	+	0,37697	-0,13825	
**A0A0H2XFQ3**	+		1,882728	2,495381	+	1,223536	1,286238	
**A0A0H2XG37**	+		4,911101	6,435907	+	1,431304	-0,77283	
**A0A0H2XH24**	+		2,987767	3,5633	+	0,686818	-0,32706	
**A0A0H2XHU6**	+		3,434236	4,114889	+	0,938807	-0,45147	
**A0A0H2XHV8**	+		3,148696	4,066203	+	0,703723	-0,3857	
**A0A0H2XII6**	+		3,538899	4,810197	+	1,037156	-0,48462	
**A0A0H2XJX6**	+		2,766526	2,080034	+	0,196743	0,182229	
**Q2FF55**	+	+	3,025561	2,028389	+	0,494634	-0,22369	
**Q2FGI7**	+		3,598635	1,451049	+	0,471754	-0,14458	
**A0A0H2XFF8**	+		1,021824	3,437964		0,132324	-0,23094	
**A0A0H2XI95**	+		0,947552	2,46362		0,750279	-0,18062	
**A0A0H2XIK4**	+		1,894125	0,585261		0,810625	-0,1274	
**A0A0H2XIV3**	+		0,214506	-0,16295		1,073462	0,864749	
**A0A0H2XJK0**	+		0,591483	0,177837		0,119411	0,042674	
**A0A0H2XJW8**	+		1,752239	0,698915		0,051197	-0,03459	
**Q2FF15**	+		2,977222	0,945261		0,504706	0,100689	
**Q2FFN1**	+		1,939537	0,961027		0,127454	-0,15396	
**Q2FG69**	+		0,343236	0,10349		0,116814	-0,04713	
**Q2FGI6**	+		1,121005	0,4761		0,406721	-0,2523	
**Q2FHT1**	+	+	0,085824	-0,08424		0,087838	-0,06088	

Table SI 15: Raw data table for competitive ABPP experiments in *S. aureus* USA300 TnpdxS using 100 µM **PL2** and 2.5 mM CCG-50014.

Uniprot Code	PLP-DE	Essential	PL2 100 µM Enrichment			CCG-50014 2.5 mM		
			**-Log Student's T-test p-value**	**Student's T-test Difference**	**Significant**	**-Log Student's T-test p-value**	**Student's T-test Difference**	**Competition**
**A0A0H2XFH9**	+		3,917541	4,501469	+	2,091618	-1,72843	+
**Q2FIR7**	+		2,887177	2,325665	+	2,913602	-1,4787	+
**A0A0H2XFD9**	+		3,728585	5,513123	+	1,890168	-1,16428	+
**A0A0H2XFY9**	+		4,87032	4,982655	+	2,802169	-1,09017	+
**A0A0H2XHH8**	+		2,422	8,045567	+	1,87994	-1,18627	+
**A0A0H2XHJ5**	+	+	2,774298	4,140401	+	2,096566	-1,35522	+
**A0A0H2XIS2**	+		4,073272	4,848108	+	2,170498	-1,3893	+
**A0A0H2XKI8**	+	+	2,735126	2,097936	+	2,422903	-1,78145	+
**A0A0H2XGP0**	+		3,492231	6,89933	+	1,929864	-1,40024	+
**A0A0H2XFA8**	+		3,67963	4,224853	+	2,503884	-0,93651	
**A0A0H2XFQ3**	+		1,882728	2,495381	+	0,341116	0,467168	

**A0A0H2XG37**	+		4,911101	6,435907	+	2,119029	-0,99498	
**A0A0H2XH24**	+		2,987767	3,5633	+	1,750805	-0,97221	
**A0A0H2XHU6**	+		3,434236	4,114889	+	1,595296	-0,80787	
**A0A0H2XHV8**	+		3,148696	4,066203	+	0,973099	-0,51333	
**A0A0H2XII6**	+		3,538899	4,810197	+	0,502523	-0,26283	
**A0A0H2XJX6**	+		2,766526	2,080034	+	0,138675	-0,2528	
**Q2FF55**	+	+	3,025561	2,028389	+	0,62435	-0,33229	
**Q2FGI7**	+		3,598635	1,451049	+	1,64673	-0,46024	
**A0A0H2XFF8**	+		1,021824	3,437964		0,223795	-0,38497	
**A0A0H2XI95**	+		0,947552	2,46362		1,000025	-0,49065	
**A0A0H2XIK4**	+		1,894125	0,585261		1,002244	-0,37308	
**A0A0H2XIV3**	+		0,214506	-0,16295		2,303561	1,279907	
**A0A0H2XJK0**	+		0,591483	0,177837		0,095431	0,038787	
**A0A0H2XJW8**	+		1,752239	0,698915		0,812511	-0,36094	
**Q2FF15**	+		2,977222	0,945261		0,047782	-0,01162	
**Q2FFN1**	+		1,939537	0,961027		0,944394	-0,51592	
**Q2FG69**	+		0,343236	0,10349		0,091958	-0,05639	
**Q2FGI6**	+		1,121005	0,4761		1,04485	-0,4914	
**Q2FHT1**	+	+	0,085824	-0,08424		0,243619	-0,13513	

Experimental Procedures

Chemical Synthesis

General Methods

Commercially available starting materials, reagents, screening compounds and anhydrous solvents were obtained from *Sigma Aldrich, TCI, Arcos, VWR, Thermo Scientific, Merck, Roth, Enamine, Carbosynth, Biomol* and *Alfa Aesar*, and used without further purification. All air and moisture sensitive reactions were carried out under argon atmosphere in flame-dried reaction flasks. Anhydrous solvents and water-sensitive liquid chemicals were transferred using argon flushed syringes. Percent values (%) refer to mass percent values. The solutions used are aqueous solutions, unless otherwise stated.
Column chromatography was carried out using silica gel [40-63 µm (Si 60)] from *Merck*. The mixture of solvents is given as the ratio of volume.

Analytical Methods

Analytical Thin Layer Chromatography (TLC)
Qualitative thin layer chromatography was recorded on silica gel plates (aluminium) from *Merck* (0.25 mm silica 60, F254). For visualization, TLC Plates were observed under UV-light (λ = 254 and 366 nm), and/or stained with $KMnO_4$ (3.00 g $KMnO_4$, 20.0 g K_2CO_3 and 5.00 mL 5% NaOH in 300 mL water), CAM (5.00 g Cer-(IV)-sulfate, 25.0 g ammoniummolybdate and 50.0 mL concentrated sulphuric acid in 450 ml water) or PMA (10.0 g phosphomolybdic acid hydrate in 200 mL ethanol) with subsequent heat treatment (ca. 250 °C).
High Pressure Liquid Chromatography (HPLC)
Compounds were purified by preparative, reversed-phase HPLC using a *Waters* 2545 quaternary gradient module equipped with a *Waters* 2998 photodiode array detector and fraction collector on a *YMC* Triart C18 column. Gradients are listed in **Table SI 16**, using ddH_2O and HPLC-grade acetonitrile (no TFA unless otherwise stated) as the mobile phase.

Table SI 16: HPLC Gradients.

	Method A		Method B		Method C	
**t[min]**	%-H_2O+ 0.1%TFA	%-MeCN+0.1% TFA	%-H_2O	%-MeCN	%-H_2O	%-MeCN
**0**	98	2	98	2	98	2
**1**	98	2	90	10	98	2
**12**	50	50	50	50	70	30
**13**	2	98	2	98	2	98
**14**	2	98	2	98	2	98
**15**	98	2	98	2	98	2
**17**	98	2	98	2	98	2

NMR-Spectroscopy
NMR spectra were recorded on *Bruker* AVHD-300, AVHD-400, AVHD-500 instruments or on an AV-II-500 equipped with cryo probe head. The chemical shifts (δ) are reported in parts per million (ppm) and spectra are referenced to residual proton and carbon signals of the deuterated solvents $CDCl_3$, d^6-DMSO and CD_3OD.
$CDCl_3$: δ (^1H) = 7.26 ppm, δ (^{13}C) = 77.16 ppm
d^6-DMSO: δ (^1H) = 2.50 ppm, δ (^{13}C) = 39.52 ppm
CD_3OD: δ (^1H) = 3.31, 4.87 ppm, δ (^{13}C) = 49.00 ppm
The following abbreviations are used to describe NMR coupling patterns: s-singlet; d-doublet; t-triplet; q-quartet; p-quintet; br-broad; *virt.*-virtual. The coupling constants, *J*, are reported in Hertz (Hz).

Mass-Spectrometry
HRMS spectra were acquired by ESI using a LTQ-FT Ultra mass spectrometer (*Thermo Scientific*) or a LTQ-Orbitrap XL mass spectrometer (*Thermo Scientific*). LC-MS measurements were conducted on a MSQ Plus mass spectrometer (*Thermo Scientific*) with a ESI source. Processing of MS data was performed using Xcalibur 2.2 (*Thermo Scientific*).

Synthetic Procedures and Analytical Data

General Procedures

Alkylation (GP1)[20,21]
To a dry flask containing anhydrous tetrahydrofuran at 0 °C were added diisopropylamine (2.4 eq) and *n*-butyllithium (2.5 M in hexanes, 2.2 eq), and the reaction mixture was stirred for 20 min. Upon cooling to -78 °C, the corresponding protected intermediate (1.0 eq) dissolved in anhydrous tetrahydrofuran was added dropwise. After stirring the reaction for 1 h, propargyl bromide (3.0 eq) was added at -78 °C and the reaction was allowed to warm to room temperature overnight. The reaction was quenched by adding satd. ammonium chloride solution (50 mL) at -78 °C and water (50 mL). The aqueous layer was extracted with ethyl acetate (4×60 mL), and the combined organic phases were washed with satd. sodium chloride solution (60 mL), dried over sodium sulfate and concentrated under reduced pressure. The crude product was purified by flash chromatography.

TMS-Protection of Alkynes (GP2)[22,23]
Corresponding alkyne (1.0 eq) was added to a solution of *n*-butyllithium (2.5 M in hexanes, 2.2 eq) in anhydrous tetrahydrofuran at -78 °C. The solution was then allowed to warm to -20 °C and was stirred for 30 min. Subsequently, trimethylsilyl chloride (2.5 eq) was added slowly at -78 °C and the reaction was allowed to warm to room temperature overnight. 5% acetic acid (60 mL) was then added and the mixture was stirred for 2 h at room temperature, concentrated and extracted with diethyl ether (3×50 mL). The combined organic phases were washed with satd. sodium chloride solution (150 mL), dried over sodium sulfate, filtered and concentrated. The crude product was purified by flash chromatography.

Mesylation of Alcohols (GP3)[22,24–26]
To a solution of corresponding alcohol (1.0 eq) in anhydrous dichloromethane was added triethylamine (2.0 eq). Methanesulfonyl chloride (1.2 eq) was added dropwise at -10 °C or -15 °C and the reaction was stirred for 2 h. The reaction mixture was then poured into 0.5 M hydrochloric acid (20 mL), the organic layer was separated and washed with a satd. sodium bicarbonate solution and satd. sodium chloride solution (each 30 mL). The organic phase was dried over sodium sulfate, filtered and concentrated to yield the corresponding mesylate which was used for the next reaction step without any further purification.

Bromination of Mesylates (GP4)[22,25]
To a solution of corresponding mesylate (1.0 eq) in anhydrous acetone was added lithium bromide (4.8 eq). The reaction was then stirred at 40 °C for 72 h. Upon completion, most of the solvent was removed *in vacuo* and diethyl ether (200 mL) and water (20 mL) were added. The organic layer was washed with satd. sodium sulfate solution, satd. sodium bicarbonate solution and water (each 50 mL) and subsequently dried over sodium sulfate, filtered and concentrated *in vacuo*. The crude product was purified by flash chromatography.

Substitution with Sodium Azide (GP5)[26,27]
To a solution of corresponding mesylate (1.0 eq) in anhydrous dimethyl formamide was added sodium azide (1.1 eq), and the suspension was heated to 65 °C with vigorous stirring for 3 h under argon. Then, water (20 mL) was added, the suspension was filtered through a plug of Celite©, and the Celite© was washed with diethyl ether (100 mL). The water-dimethyl formamide mixture was extracted with diethyl ether (3×30 mL), and the combined organic layers were washed with water (2×50 mL) and satd. sodium chloride solution (60 mL). The organic phase was then dried over sodium sulfate, filtered and concentrated *in vacuo*. The crude product was purified by flash chromatography.

Nucleophilic Substitution (GP6)[28]
Protected *N*-oxide (1.0 eq) was added to a dry flask containing anhydrous tetrahydrofuran and the solution was cooled to -78 °C. Subsequently, *iso*-butylchloroformate (1.0 eq) was added dropwise. The reaction was stirred for 30 min, upon which corresponding alkyne (2.0 eq) was rapidly added. The reaction was stirred at -78 °C for 1 h upon which the ice bath was removed and the reaction was allowed to warm to room temperature for 1 h. The reaction was quenched by adding satd. sodium bicarbonate solution (30 mL) at -78 °C. The aqueous layer was extracted with diethyl ether (3×30 mL). The combined organic phases were dried over sodium sulfate and concentrated under reduced pressure. The crude product was purified by flash chromatography.

Click-Reaction (GP7)[29]
To a solution of corresponding alkyne (1.0 eq) in acetonitrile/dimethyl sulfoxide (4:1) were added sodium ascorbate (0.20 eq) and copper sulfate pentahydrate (0.20 eq). Subsequently, corresponding azide (1.0 eq) was added to the reaction mixture dropwise under vigorous stirring and the reaction was stirred overnight at room temperature. Upon completion, the reaction was quenched with satd. ammonium chloride solution (10 mL), forming a white precipitate. After separation, the aqueous layer was extracted with ethyl acetate (3×30 mL). The combined organic phases were washed with satd. ammonium chloride solution (50 mL) and dried over sodium sulfate. Upon concentration under reduced pressure, the residue was purified by flash chromatography.

TMS Deprotection (GP8)[23]
To a solution of corresponding TMS-protected alkyne (1.0 eq) dissolved in anhydrous methanol was added potassium carbonate (1.0-5.0 eq) and the reaction was stirred overnight at room temperature. Upon completion, diethyl ether and water were added (each 20 mL) and the aqueous layer was extracted with diethyl ether (3×40 mL). After concentration under reduced pressure, the residue was purified by flash chromatography.

Acetal and Ether Deprotection (GP9)[21,28]

Corresponding MOM-protected acetal (1.0 eq) was dissolved in a mixture of acetone/water (1:1) containing 5% sulfuric acid and refluxed at 85 °C for 1 h. The reaction was allowed to cool to room temperature and was then concentrated under reduced pressure. The product was purified by HPLC using the given method.

TMS, Acetal and Ester Deprotection (GP10)[28]

Corresponding Piv-protected alkyne (1.0 eq) was dissolved in 2% potassium hydroxide in methanol and stirred at room temperature for 2 h. The reaction was diluted with dichloromethane (10 mL), passed through silica gel and washed with a mixture of dichloromethane/methanol (1:1). The filtrate was concentrated to dryness and the resulting brown solid was dissolved in 5% sulfuric acid in acetone/water (1:1). The mixture was heated at 85 °C for 1 h and then the reaction was allowed to cool down and acetone was removed under reduced pressure. The product was purified by HPLC using the given method.

Phenelzine Derivatisation (GP11)[30]

Procedure adapted from a previously published protocol by *Prusevich et al*.[30] Hydrazine monohydrate (10 eq)† was added to a stirred solution of the appropriate alkyl halide (1.0 eq) in ethanol (3 mL). The reaction mixture was refluxed for overnight, after which the mixture was concentrated to dryness *in vacuo*. The crude residue was dissolved in a minimum volume of acetonitrile/water and purified by preparative reversed-phase HPLC. Unless otherwise stated, the eluents as given in **Table SI 16** (Method A). The fractions containing the product were combined and dried by lyophilisation.
†CAUTION: Hydrazine monohydrate is highly toxic.

Probe Synthesis

PL1, PL2, PL3, PL4 and **PL5** were synthesized as described previously.[21,31]

1-Methoxy-6-methyl-1,3-dihydrofuro[3,4-*c*]pyridin-7-ol[28]

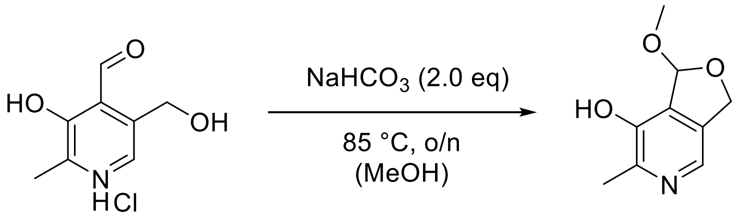

1

$C_9H_{11}NO_3$
M = 181.19 g/mol

Pyridoxal hydrochloride (7.00 g, 34.4 mmol, 1.0 eq) was suspended in anhydrous methanol (70 mL) and refluxed at 85 °C for 1 h. After cooling to room temperature, sodium bicarbonate (2.89 g, 34.4 mmol, 1.0 eq) was added. The reaction was stirred at 85 °C overnight. The obtained white mixture was filtered three times to remove precipitated sodium chloride and washed with dichloromethane (1×50 mL). Removal of the solvents caused crystallization of intermediate acetal **1**, which was used in the following reaction without further purification.

TLC: *R*f = 0.50 (10% MeOH/DCM) [KMnO4].
HRMS (ESI) (C9H11NO3 $[M+H]^+$) calcd.: 182.0817
found: 182.0811.

1-Methoxy-7-(methoxymethoxy)-6-methyl-1,3-dihydrofuro[3,4-*c*] pyridine[21,32]

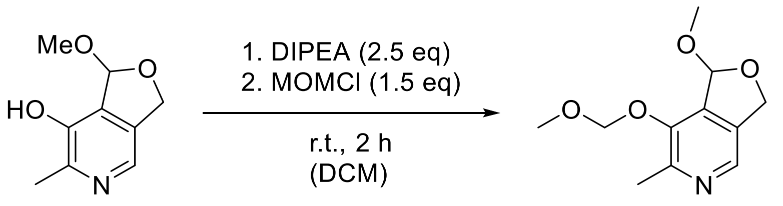

1

2

$C_{11}H_{15}NO_4$
M = 225.24 g/mol

1-Methoxy-6-methyl-1,3-dihydrofuro[3,4-*c*]pyridin-7-ol (5.49 g, 30.3 mmol, 1.0 eq) was dissolved in anhydrous dichloromethane (55 mL) and diisopropylethylamine (13.2 mL, 9.72 g, 75.8 mmol, 2.5 eq) was added while stirring. Chloromethyl methyl ether (3.45 mL, 3.66 g, 45.4 mmol, 1.5 eq) was then added dropwise at 0 °C and the reaction mixture was stirred for 2 h at room temperature. After removal of the solvent and purification by flash chromatography (MeOH/DCM, 1-4%), compound **2** (6.16 g, 27.3 mmol, 80% over 2 steps) was obtained as a yellow oil.

TLC: R_f = 0.49 (80% EtOAc/hexanes) [UV/CAM].
^{1}H-NMR (400 MHz, $CDCl_3$): δ[ppm] = 8.15 (s, 1 H), 6.26 (s, 1 H), 5.43 (d, ^4J = 6.5 Hz, 1 H), 5.18 (d, ^2J = 12.7 Hz, 1 H), 5.08 (d, ^4J = 6.5 Hz, 1 H), 5.03 (d, ^2J = 12.7 Hz, 1 H), 3.51 (s, 3 H), 3.46 (s, 3 H), 2.56 (s, 3 H).
^{13}C-NMR (100 MHz, $CDCl_3$): δ[ppm] = 150.2, 146.5, 136.0, 135.0, 134.2, 106.2, 96.3, 70.2, 56.9, 54.9, 19.4.
HRMS (ESI) ($C_{11}H_{15}NO_4$ $[M+H]^+$) calcd.: 226.1079
found: 226.1072.

1-Methoxy-6-methyl-1,3-dihydrofuro[3,4-*c*]pyridin-7-yl pivalate[28]

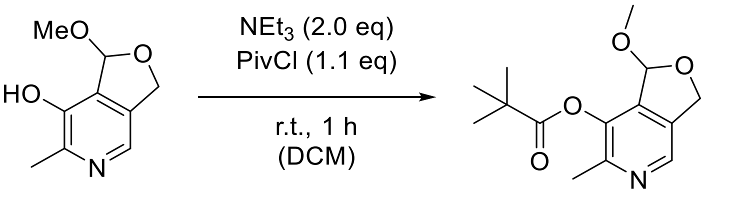

1 **3**

$C_{14}H_{19}NO_4$
M = 265.31 g/mol

To a solution of 1-methoxy-6-methyl-1,3-dihydrofuro[3,4-*c*]pyridin-7-ol (3.64 g, 20.1 mmol, 1.0 eq) in anhydrous dichloromethane (60 mL) was added triethylamine (5.26 mL, 3.87 g, 38.2 mmol, 1.9 eq). Subsequently, pivaloyl chloride (2.75 mL, 2.71 g, 22.5 mmol, 1.1 eq) was added at 0 °C and the reaction mixture was stirred for 2 h at room temperature. Upon completion, the solution was concentrated under reduced pressure to yield compound **3** as a pale yellow oil, which did not require further purification.

TLC: R_f = 0.56 (50% EtOAc/hexanes) [UV/$KMnO_4$].
^{1}H-NMR (400 MHz, $CDCl_3$): δ[ppm] = 8.35 (s, 1 H), 6.09 (s, 1 H), 5.23 (d, ^2J = 12.8 Hz, 1 H), 5.10 (d, ^2J = 12.8 Hz, 1 H), 3.37 (s, 3 H), 2.45 (s, 3 H), 1.40 (s, 9 H).
^{13}C-NMR (100 MHz, $CDCl_3$): δ[ppm] = 175.5, 150.9, 141.2, 139.6, 138.4, 135.8, 106.1, 70.8, 54.6, 39.4, 27.2, 18.9.
HRMS (ESI) ($C_{14}H_{19}NO_4$ $[M+H]^+$) calcd.: 266.1392
found: 266.1385.
The analytical data are in accordance with previous literature reports.[28]

1-Methoxy-6-methyl-7-(pivaloyloxy)-1,3-dihydrofuro[3,4-*c*]pyridine-*N*-oxide[28]

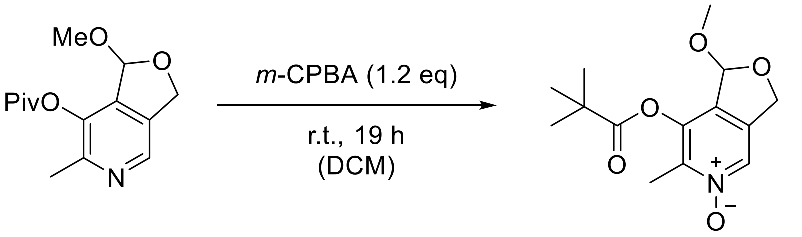

3 **4**

$C_{14}H_{19}NO_5$
M = 281.31 g/mol

Meta-chloro perbenzoic acid (77%, 9.15 g, 23.0 mmol, 1.2 eq) was added to a solution of acetal (5.23 g, 19.7 mmol, 1.0 eq) in anhydrous dichloromethane (100 mL) in five portions. After addition, the reaction was stirred overnight at room temperature. Then, the reaction mixture was quenched with satd. sodium thiosulfate solution (100 mL) and diluted with dichloromethane (80 mL). After separation of the phases, the organic layer was washed with satd. sodium bicarbonate and satd. sodium chloride solutions (each 100 mL), dried over sodium sulfate and concentrated to yield the intermediate *N*-oxide **4** as a pale yellow solid, which was used directly in the next step.

TLC: R_f = 0.44 (5% MeOH/DCM) [UV/$KMnO_4$].
^{1}H-NMR (400 MHz, $CDCl_3$): δ[ppm] = 8.19 (s, 1 H), 6.03 (s, 1 H), 5.18 (d, ^2J = 13.3 Hz, 1 H), 5.05 (d, ^2J = 13.3 Hz, 1 H), 3.38 (s, 3 H), 2.37 (s, 3 H), 1.40 (s, 9 H).
^{13}C-NMR (100 MHz, $CDCl_3$): δ[ppm] = 174.9, 160.7, 145.0, 142.8, 140.6, 136.3, 106.1, 70.5, 54.7, 39.5, 27.1, 11.6.
HRMS (ESI) ($C_{14}H_{19}NO_5$ $[M+H]^+$) calcd.: 282.1336
found: 282.1333.

The analytical data are in accordance with previous literature reports.[28]

Linker Synthesis

5-(Trimethylsilyl)pent-4-yn-1-ol[22,33]

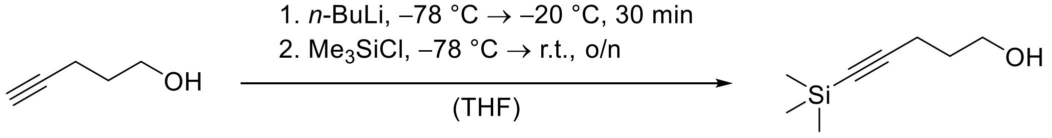

5 **6** $C_8H_{16}OSi$
M = 156.30 g/mol

Following **GP2**, alkyne **5** (2.00 g, 23.8 mmol, 1.0 eq) was converted with *n*-butyllithium (2.5 M in hexanes; 21.0 mL, 2.2 eq) and trimethylsilyl chloride (7.54 mL, 6.46 g, 59.4 mmol, 2.5 eq) in anhydrous tetrahydrofurane (40 mL). After purification by column chromatography (EtOAc/hexanes, 20-60%), TMS-protected compound **6** (3.32 g, 21.3 mmol, 90%) was obtained as a pale yellow oil.

TLC: R_f = 0.76 (50% EtOAc/hexanes) [CAM].
^{1}H-NMR (400 MHz, $CDCl_3$): δ[ppm] = 3.76 (t, ^3J = 6.5 Hz, 2 H), 2.35 (t, ^3J = 6.5 Hz, 2 H), 1.77 (p, ^3J = 6.5 Hz, 2 H), 0.14 (s, 9 H).
^{13}C-NMR (100 MHz, $CDCl_3$): δ[ppm] = 106.6, 85.3, 62.0, 31.2, 16.6, 0.10.

The analytical data are in accordance with previous literature reports.[22]

3-(Trimethylsilyl)prop-2-yn-1-yl methanesulfonate[24]

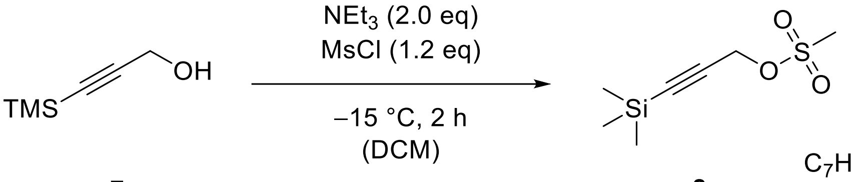

7 **8** $C_7H_{14}O_3SSi$
M = 206.33 g/mol

Following **GP3**, alcohol **7** (1.16 mL, 1.00 g, 7.98 mmol, 1.0 eq) was converted with triethylamine (2.16 mL, 1.58 g, 15.6 mmol, 2.0 eq) and methanesulfonyl chloride (6.48 mL, 5.53 g, 50.9 mmol, 2.5 eq) in anhydrous dichloromethane (40 mL). After concentration *in vacuo*, mesylate **8** (1.23 g, 5.96 mmol, 75%) was obtained and used for the next reaction step without any further purification.

TLC: R_f =0.67 (20% EtOAc/hexanes) [$KMnO_4$].

4-(Trimethylsilyl)but-3-yn-1-yl methanesulfonate[22,25,26]

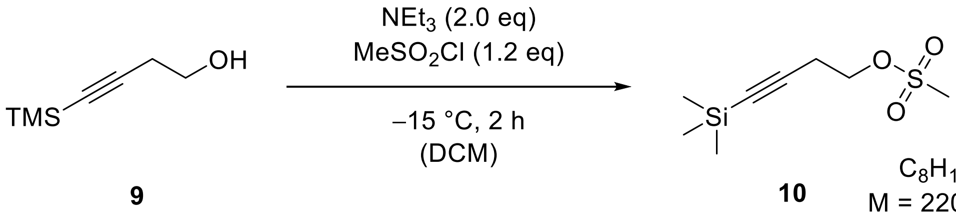

9 **10** $C_8H_{16}O_3SSi$
M = 220.36 g/mol

Following **GP3**, alcohol **9** (2.34 mL, 2.00 g, 14.1 mmol, 1.0 eq) was converted with triethylamine (3.91 mL, 2.85 g, 28.1 mmol, 2.0 eq) and methanesulfonyl chloride (1.31 mL, 1.93 g, 16.9 mmol, 1.2 eq) in anhydrous dichloromethane (50 mL). After concentration *in vacuo*, mesylate **10** (3.12 g, 14.1 mmol, 100%) was obtained and used for the next reaction step without any further purification.

TLC: R_f = 0.83 (50% EtOAc/hexanes) [CAM].

5-(Trimethylsilyl)pent-4-yn-1-yl methanesulfonate[22,25]

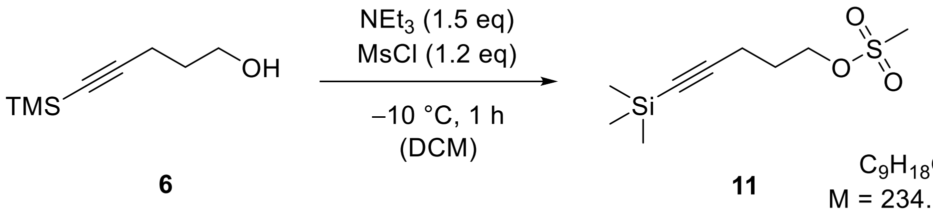

6 **11** $C_9H_{18}O_3SSi$ M = 234.39 g/mol

Following **GP3**, alcohol **6** (3.48 mL, 3.00 g, 19.2 mmol, 1.0 eq) was converted with triethylamine (3.99 mL, 2.91 g, 28.8 mmol, 1.5 eq) and methanesulfonyl chloride (1.78 mL, 2.64 g, 23.0 mmol, 1.2 eq) in anhydrous dichloromethane (50 mL) at -10 °C. After concentration *in vacuo*, mesylate **11** (4.41 g, 18.8 mmol, 98%) was obtained and used for the next reaction step without any further purification.

TLC: R_f = 0.87 (50% EtOAc/hexanes) [CAM].

(4-Bromobut-1-yn-1-yl)trimethylsilane[22]

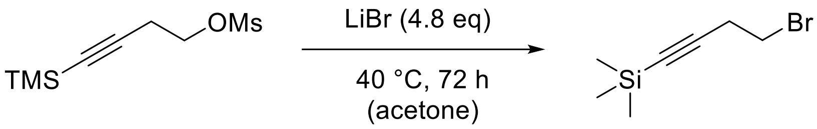

10 **12** $C_7H_{13}BrSi$ M = 205.17 g/mol

Following **GP4**, mesylate **8** (3.12 g, 14.1 mmol, 1.0 eq) was converted with lithium bromide (5.87 g, 67.7 mmol, 4.8 eq) in anhydrous acetone (30 mL). The crude product was purified by flash chromatography (hexanes) to yield bromide **12** (2.69 g, 13.1 mmol, 93%) as a clear oil.

TLC: R_f = 0.58 (hexanes) [CAM].
^{1}H-NMR (400 MHz, $CDCl_3$): δ[ppm] = 3.43 (t, ^3J = 7.5 Hz, 2 H), 2.77 (t, ^3J = 7.5 Hz, 2 H), 0.16 (s, 9 H).
^{13}C-NMR (100 MHz, $CDCl_3$): δ[ppm] = 103.3, 87.2, 29.3, 24.5, 0.1.

The analytical data are in accordance with previous literature reports.[22]

(5-Bromopent-1-yn-1-yl)trimethylsilane[22,25]

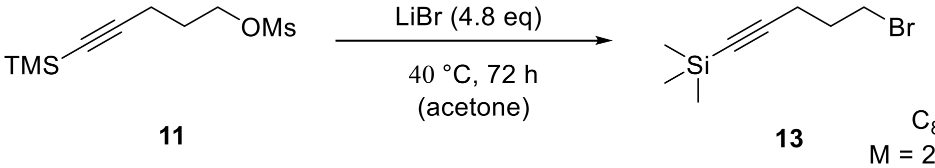

11 **13** $C_8H_{15}BrSi$ M = 219.20 g/mol

Following **GP4**, mesylate **11** (4.40 g, 18.8 mmol, 1.0 eq) was converted with lithium bromide (7.83 g, 90.2 mmol, 4.8 eq) in anhydrous acetone (50 mL). The crude product was purified by flash chromatography (hexanes) to yield bromide **13** (3.46 g, 15.8 mmol, 85%) as a clear oil.

TLC: R_f = 0.42 (hexanes) [CAM].
^{1}H-NMR (400 MHz, $CDCl_3$): δ[ppm] = 3.51 (t, ^3J = 6.7 Hz, 2 H), 2.41 (t, ^3J = 6.7 Hz, 2 H), 2.04 (p, ^3J = 6.7 Hz, 2 H), 0.15 (s, 9 H).
^{13}C-NMR (100 MHz, $CDCl_3$): δ[ppm] = 105.2, 85.9, 32.4, 31.6, 18.8, 0.24.

The analytical data are in accordance with previous literature reports.[22]

(3-Azidoprop-1-yn-1-yl)trimethylsilane[27]

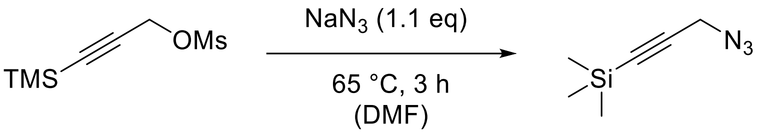

8 **14** $C_6H_{11}N_3Si$
M = 153.26 g/mol

Following **GP5**, mesylate **8** (1.23 g, 5.96 mmol, 1.0 eq) was converted with sodium azide (426 mg, 6.56 mmol, 1.1 eq) in anhydrous dimethyl formamide (20 mL). The crude product was purified by flash chromatography (EtOAc/hexanes, 5-30%) to yield azide **14** (366 mg, 2.39 mmol, 41%) as a clear oil.

TLC: R_f = 0.80 (10% EtOAc/hexanes) [$KMnO_4$].
^{1}H-NMR (400 MHz, $CDCl_3$): δ[ppm] = 3.92 (s, 2 H), 0.20 (s, 9 H).
^{13}C-NMR (100 MHz, $CDCl_3$): δ[ppm] = 97.1, 93.2, 40.8, -0.1.

(4-Azidobut-1-yn-1-yl)trimethylsilane[26,27]

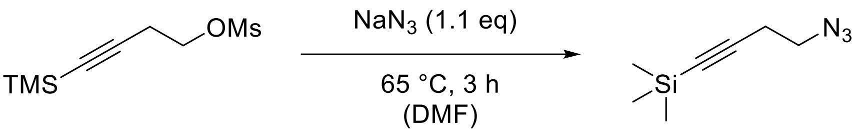

10 **15** $C_7H_{13}N_3Si$
M = 167.29 g/mol

Following **GP5**, mesylate **10** (3.41 g, 15.5 mmol, 1.0 eq) was converted with sodium azide (1.11 g, 17.0 mmol, 1.1 eq) in anhydrous dimethyl formamide (40 mL). The crude product was purified by flash chromatography (EtOAc/hexanes, 0-30%) to yield azide **15** (1.89 g, 11.3 mmol, 73%) as a clear oil.

TLC: R_f = 0.88 (10% EtOAc/hexanes) [$KMnO_4$].
^{1}H-NMR (400 MHz, $CDCl_3$): δ[ppm] = 3.38 (t, ^3J = 6.9 Hz, 2 H), 2.52 (t, ^3J = 6.9 Hz, 2 H), 0.16 (s, 9 H).
^{13}C-NMR (100 MHz, $CDCl_3$): δ[ppm] = 102.8, 87.4, 49.9, 21.1, 0.04.

The analytical data are in accordance with previous literature reports.[26]

3-(Trimethylsilyl)prop-2-yn-1 magnesium bromide[34]

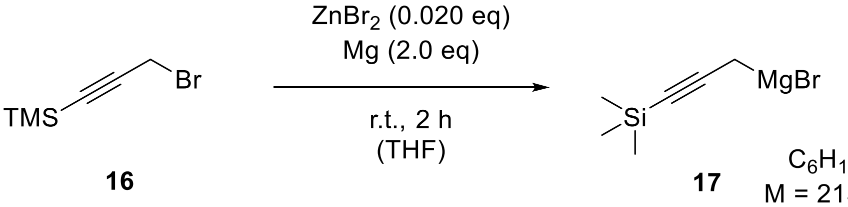

16 **17** $C_6H_{11}BrMgSi$
M = 215.45 g/mol

To a dry three-necked flask (100 mL) equipped with a condenser and a dropping funnel were added zink(II)bromide (100 mg, 442 µmol, 2 mol%), magnesium (977 mg, 40.2 mmol, 2.0 eq) and anhydrous diethyl ether (12 mL). Then, (3-bromoprop-1-yn-1-yl)trimethylsilane (3.40 mL, 4.00 g, 20.1 mmol, 1.0 eq) in anhydrous diethyl ether (15 mL) was added dropwise using a dropping funnel. The addition began at room temperature and continued for 30 min until an exothermic reaction was observed. Following this observation, the mixture was cooled to 0 °C and stirred at this temperature for an additional 2 h. The freshly generated reagent was directly used in the following GRIGNARD-reaction.

4-(Trimethylsilyl)but-3-yn-1 magnesium bromide[35]

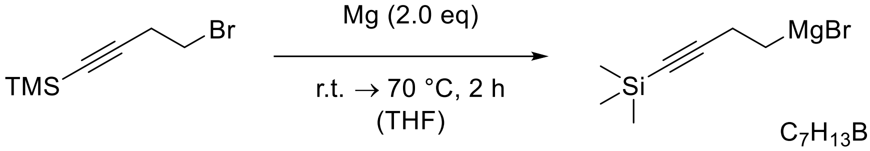

12 **18** $C_7H_{13}BrMgSi$ M = 229.48 g/mol

To a dry three-necked flask (100 mL) equipped with a condenser and a dropping funnel, were added magnesium (391 mg, 16.1 mmol, 1.5 eq) and anhydrous tetrahydrofurane (6 mL). Then (4-bromobut-1-yn-1-yl)trimethylsilane (2.20 g, 10.7 mmol, 1.0 eq) in anhydrous tetrahydrofurane (8 mL) was added dropwise using a dropping funnel. The addition began at room temperature and continued under reflux at 70 °C for 1 h until an exothermic reaction was observed. Following this observation, the mixture was stirred for an additional 1 h. The freshly generated reagent was directly used in the following GRIGNARD-reaction.

Alkylations

1-Methoxy-7-(methoxymethoxy)-6-(pent-4-yn-1-yl)-1,3-dihydrofuro[3,4-*c*]pyridine[21]

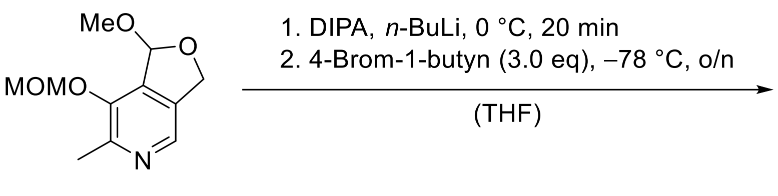

2 **19** $C_{15}H_{19}NO_4$ M = 277.32 g/mol

Following **GP1**, protected PL **2** (300 mg, 1.33 mmol, 1.0 eq) was converted with diisopropylamine (451 µL, 323 mg, 3.19 mmol, 2.4 ,eq), *n*-butyllithium (2.5 M in hexanes; 1.17 mL, 188 mg, 2.93 mmol, 2.2 eq) and 4-bromobut-1-yne (375 µL, 531 mg, 3.99 mmol, 3.0 eq) in anhydrous tetrahydrofuran (9 mL). The crude product was purified by flash chromatography (EtOAc/hexanes, 20-60%) to yield alkylated compound **19** (48.0 mg, 173 µmol, 14%) as a pale yellow oil.

TLC: R_f = 0.66 (50% EtOAc/hexanes) [UV/$KMnO_4$].
^{1}H-NMR (400 MHz, $CDCl_3$): δ[ppm] = 8.18 (s, 1 H), 6.26 (s, 1 H), 5.44 (d, ^4J = 6.5 Hz, 1 H), 5.19 (d, ^2J = 12.8 Hz, 1 H), 5.08 (d, ^4J = 6.5 Hz, 1 H), 5.02 (d, ^2J = 12.8 Hz, 1 H), 3.52 (s, 3 H), 3.47 (s, 3 H), 3.00 (t, ^3J = 7.3 Hz, 2 H), 2.29 (td, $^{3,4}J$ = 7.3, 2.5 Hz, 2 H), 1.97 (p, ^3J = 7.3 Hz, 2 H), 1.96 (t, ^4J = 2.5 Hz, 1 H).
^{13}C-NMR (100 MHz, $CDCl_3$): δ[ppm] = 152.8, 146.3, 135.9, 135.7, 133.8, 106.3, 96.4, 84.5, 70.1, 68.6, 57.0, 55.0, 31.7, 27.5, 18.5.
HRMS (ESI) ($C_{13}H_{15}NO_3$ $[M+H]^+$) calcd.: 278.1387
found: 278.1382.

1-Methoxy-7-(methoxymethoxy)-6-[6-(trimethylsilyl)hex-5-yn-1-yl]-1,3-dihydrofuro[3,4-*c*]pyridine[21]

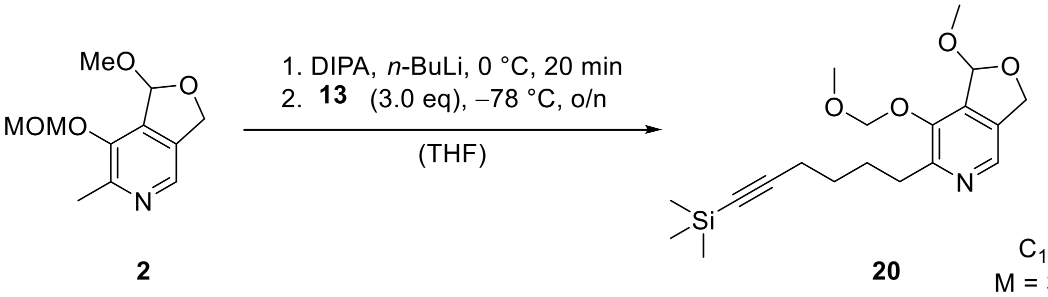

2 **20** $C_{19}H_{29}NO_4Si$ M = 363.53 g/mol

Following **GP1**, protected PL **2** (300 mg, 1.33 mmol, 1.0 eq) was converted with diisopropylamine (451 µL, 323 mg, 3.19 mmol, 2.4 ,eq), *n*-butyllithium (2.5 M in hexanes; 1.17 mL, 188 mg, 2.93 mmol, 2.2 eq) and (5-bromopent-1-yn-1-yl)trimethylsilane (875 mg, 3.99 mmol,

3.0 eq) in anhydrous tetrahydrofuran (9 mL). The crude product was purified by flash chromatography (EtOAc/hexanes, 20-60%) to yield alkylated compound **20** (265 mg, 729 µmol, 55%) as a pale yellow oil.
TLC: R_f = 0.61 (50% EtOAc/hexanes) [UV/KMnO$_4$].
^{1}H-NMR (400 MHz, CDCl$_3$): δ[ppm] = 8.19 (s, 1 H), 6.28 (s, 1 H), 5.48 (d, ^4J = 6.4 Hz, 1 H), 5.20 (d, ^2J = 13.5 Hz, 1 H), 5.09 (d, ^4J = 6.4 Hz, 1 H), 5.05 (d, ^2J = 13.5 Hz, 1 H), 3.52 (s, 3 H), 3.48 (s, 3 H), 2.99 (s, 2 H), 2.27 (t, ^3J = 7.5 Hz, 2 H), 1.83 (p, ^3J = 7.5 Hz, 2 H), 1.62 (p, ^3J = 7.5 Hz, 2 H), 0.13 (s, 9 H).
^{13}C-NMR (100 MHz, CDCl$_3$): δ[ppm] = 153.1, 146.8, 136.3, 134.5, 106.2, 96.4, 84.6, 77.4, 70.0, 68.3, 57.1, 54.9, 34.5, 28.7, 28.2, 19.9, 0.32.
HRMS (ESI) ($C_{19}H_{29}NO_4Si$ [M+H]$^+$) calcd.: 364.1944
found: 364.1936.

Nucleophilic Substitution

4-Ethynyl-1-methoxy-6-methyl-1,3-dihydrofuro[3,4-*c*]-pyridin-7-ylpivalate[28]

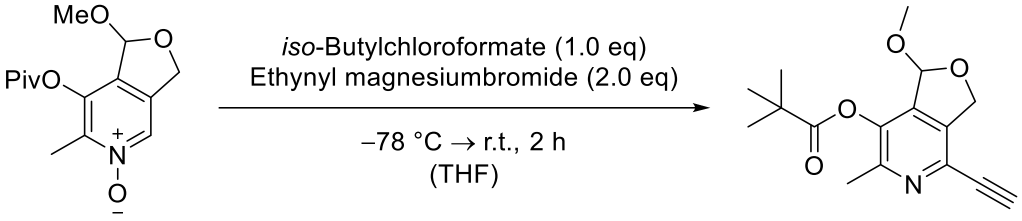

4 **21** $C_{16}H_{19}NO_4$
M = 289.33 g/mol

Following **GP6**, protected *N*-oxide **4** (619 mg, 2.20 mmol, 1.0 eq) was converted with *iso*-butylchloroformate (286 µL, 301 mg, 2.20 mmol, 1.0 eq) and ethynyl magnesiumbromide (0.5 M in tetrahydrofuran; 8.80 mL, 569 mg, 4.40 mmol, 2.0 eq) in anhydrous tetrahydrofuran (7 mL). The crude product was purified by flash chromatography (EtOAc/hexanes, 10-40%) to yield alkylated compound **21** (184 mg, 636 µmol, 29%) as a brown-yellow solid.

TLC: R_f = 0.58 (20% EtOAc/hexanes) [KMnO$_4$].
^{1}H-NMR (400 MHz, CDCl$_3$): δ[ppm] = 6.09 (d, ^4J = 1.8 Hz, 1 H), 5.22 (dd, $^{2,4}J$ = 13.7, 1.8 Hz, 1 H), 5.10 (d, ^2J = 13.7 Hz, 1 H), 3.36 (s, 3 H), 3.31 (s, 1 H), 2.44 (s, 3 H), 1.38 (s, 9 H).
^{13}C-NMR (100 MHz, CDCl$_3$): δ[ppm] = 175.3, 152.1, 141.2, 139.2, 138.4, 132.2, 106.6, 81.0, 80.0, 71.4, 54.6, 39.4, 27.1, 19.0.
HRMS (ESI) ($C_{16}H_{19}NO_4$ [M+H]$^+$) calcd.: 290.1387
found: 290.1380.

1-Methoxy-6-methyl-4-[4-(trimethylsilyl)but-3-yn-1-yl]-1,3-dihydrofuro[3,4-*c*]pyridin-7-yl pivalate[28]

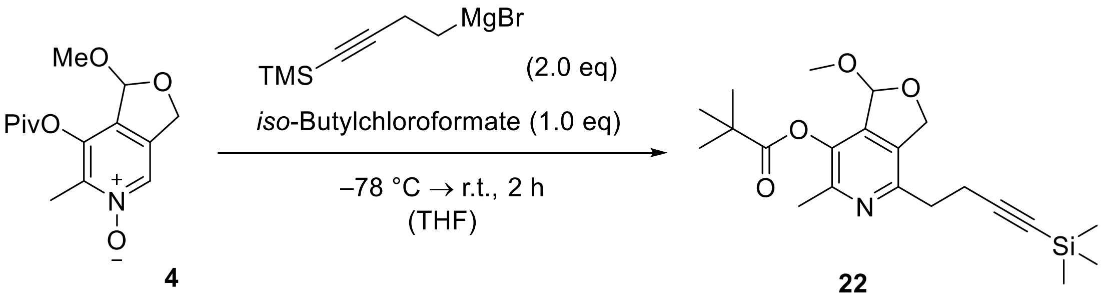

4 **22**

$C_{21}H_{31}NO_4Si$
M = 389.57 g/mol

Following **GP6**, protected *N*-oxide **4** (700 mg, 2.49 mmol, 1.0 eq) was converted with *iso*-butylchloroformate (323 µL, 340 mg, 2.49 mmol, 1.0 eq) and freshly prepared 4-(trimethylsilyl)but-3-yn-1-magnesiumbromide in anhydrous tetrahydrofuran (7 mL). The

crude product was purified by flash chromatography (EtOAc/hexanes, 5-40%) to yield a brown-yellow solid containing compound among a mixture of products that could not be separated

TLC: R_f = 0.51 (20% EtOAc/hexanes) [UV/KMnO$_4$].
HRMS (ESI) ($C_{21}H_{31}NO_4Si$ [M+H]$^+$) calcd.: 390.2095
found: 390.2093.

Click Reactions

1-Methoxy-7-(methoxymethoxy)-6-{2-{1-[3-(trimethylsilyl)prop-2-yn-1-yl]-1*H*-1,2,3-triazol-4-yl}ethyl}-1,3-dihydrofuro[3,4-*c*]pyridine[29]

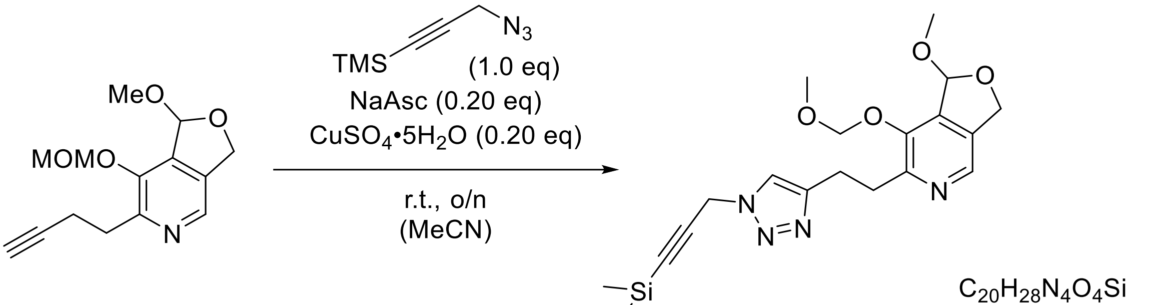

23 **24**

Following **GP7**, protected alkyne from **PL2** synthesis **23** (220 mg, 836 µmol, 1.0 eq) was converted with sodium ascorbate (33.1 mg, 167 µmol, 0.20 eq), copper sulfate pentahydrate (41.8 mg, 167 µmol, 0.20 eq) and (3-azidoprop-1-yn-1-yl)trimethylsilane (128 mg, 0.836 mmol, 1.0 eq) in acetonitrile/dimethyl sulfoxide (4:1, 10 mL). The crude product was purified by flash chromatography (MeOH/DCM, 2-6%) to yield **24** (232 mg, 558 µmol, 67%) as a yellow oil.

TLC: R_f = 0.61 (10% MeOH/DCM) [UV/KMnO$_4$].
^{1}H-NMR (400 MHz, CDCl$_3$): δ[ppm] = 8.18 (s, 1 H), 7.50 (s, 1 H), 6.24 (d, ^4J = 1.6 Hz, 1 H), 5.42 (d, ^4J = 6.6 Hz, 1 H), 5.17 (d, ^2J = 13.3 Hz, 1 H), 5.10 (s, 2 H), 5.04 (d, ^4J = 6.6 Hz, 1 H), 5.01 (d, ^2J = 13.3 Hz, 1 H), 3.48 (s, 3 H), 3.46 (s, 3 H), 3.31-3.22 (m, 2 H), 3.22-3.14 (m, 2 H), 0.18 (s, 9 H).
^{13}C-NMR (100 MHz, CDCl$_3$): δ[ppm] = 152.0, 148.0, 146.3, 136.1, 135.5, 133.7, 120.8, 106.3, 96.5, 96.1, 92.8, 70.0, 57.0, 54.9, 40.7, 32.2, 24.4, −0.30.
HRMS (ESI) ($C_{20}H_{28}N_4O_4Si$ [M+H]$^+$) calcd.: 417.1953
found: 417.1943.

1-Methoxy-7-(methoxymethoxy)-6-{2-{1-[4-(trimethylsilyl)but-3-yn-1-yl]-1*H*-1,2,3-triazol-4-yl}ethyl}-1,3-dihydrofuro[3,4-*c*]pyridine[29]

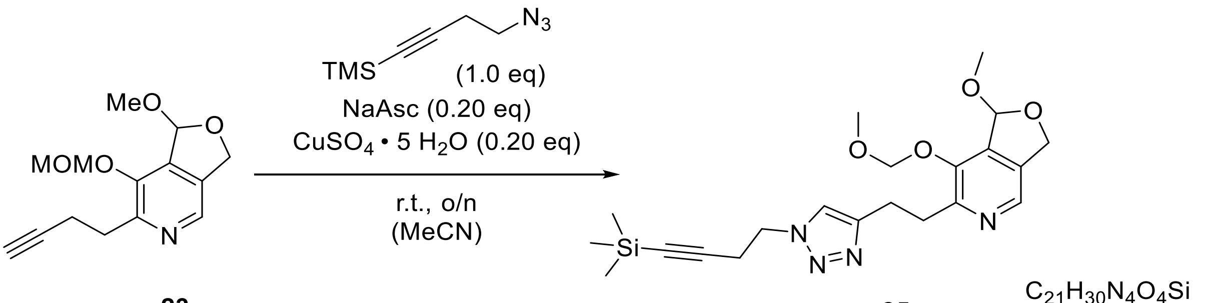

23 **25**

Following **GP7**, protected alkyne from **PL2** synthesis **23** (210 mg, 798 µmol, 1.0 eq) was converted with sodium ascorbate (31.6 mg, 0.160 mmol, 0.20 eq), copper sulfate pentahydrate (39.8 mg, 160 µmol, 0.20 eq) and (4-azidobut-1-yn-1-yl)trimethylsilane (133 mg, 798 µmol, 1.0 eq) in acetonitrile/dimethyl sulfoxide (4:1, 10 mL). The crude product was purified by flash chromatography (MeOH/DCM, 2-6%) to yield **25** (229 mg, 532 µmol, 67%) as a yellow oil.

TLC: R_f = 0.34 (EtOAc) [UV/KMnO$_4$].
^{1}H-NMR (400 MHz, CDCl$_3$): δ[ppm] = 8.18 (s, 1 H), 7.39 (s, 1 H), 6.25 (d, ^4J = 1.6 Hz, 1 H), 5.42 (d, ^4J = 6.6 Hz, 1 H), 5.18 (d, ^2J = 12.7 Hz, 1 H), 5.04 (d, ^4J = 6.6 Hz, 1 H), 5.02 (d, ^2J = 12.7 Hz, 1 H), 4.44 (t, ^3J = 6.9 Hz, 2 H), 3.48 (s, 3 H), 3.46 (s, 3 H), 3.30-3.23 (m, 2 H), 3.21-3.13 (m, 2 H), 2.77 (t, ^3J = 6.9 Hz, 2 H), 0.13 (s, 9 H).
^{13}C-NMR (100 MHz, CDCl$_3$): δ[ppm] = 152.1, 147.7, 146.3, 136.1, 135.5, 133.6, 121.5, 106.3, 102.1, 96.1, 88.2, 70.1, 57.0, 55.0, 48.8, 32.2, 24.5, 22.2, 0.03.
HRMS (ESI) ($C_{21}H_{30}N_4O_4Si$ [M+H]$^+$) calcd.: 431.2109
found: 431.2103.

1-Methoxy-6-methyl-4-{1-[3-(trimethylsilyl)prop-2-yn-1-yl]-1H-1,2,3-triazol-4-yl}-1,3-dihydrofuro[3,4-c]pyridin-7-yl pivalate[29]

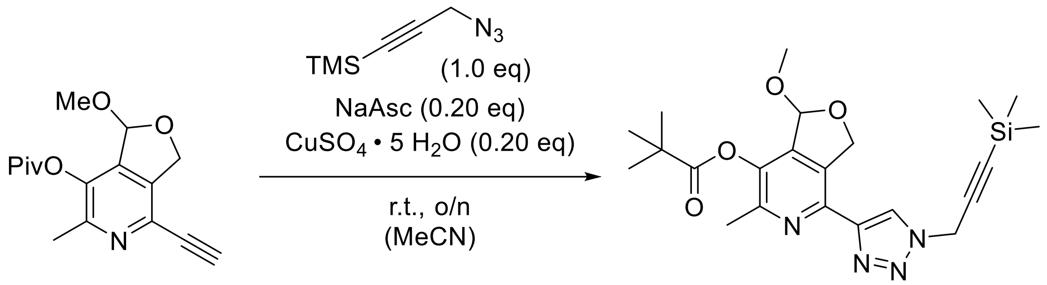

21 **26** $C_{22}H_{30}N_4O_4Si$
M = 442.59 g/mol

Following **GP7**, protected alkyne **21** (100 mg, 346 µmol, 1.0 eq) was converted with sodium ascorbate (13.7 mg, 0.0691 mmol, 0.20 eq), copper sulfate pentahydrate (17.3 mg, 0.0691 mmol, 0.20 eq) and (3-azidoprop-1-yn-1-yl)trimethylsilane (53.0 mg, 0.346 mmol, 1.0 eq) in acetonitrile/dimethyl sulfoxide (4:1, 5 mL). The crude product was purified by flash chromatography (MeOH/DCM, 2-6%) to yield a yellow oil containing compound among a mixture of products that could not be separated.

TLC: R_f = 0.49 (20% EtOAc/hexanes) [UV/KMnO$_4$].
HRMS (ESI) ($C_{22}H_{30}N_4O_4Si$ [M+H]$^+$) calcd.: 443.2109
found: 443.2108.

TMS Deprotection

6-(Hex-5-yn-1-yl)-1-methoxy-7-(methoxymethoxy)-1,3-dihydrofuro[3,4-*c*]pyridine[23]

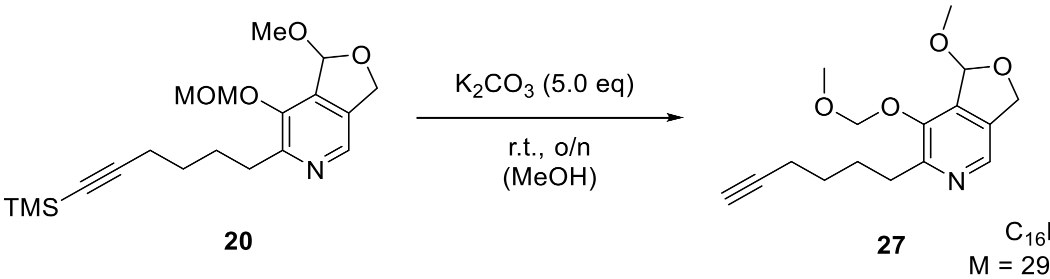

20 **27** $C_{16}H_{21}NO_4$
M = 291.35 g/mol

Following **GP8**, TMS-protected alkyne **20** (207 mg, 569 µmol, 1.0 eq) was converted with potassium carbonate (388 mg, 2.85 mmol, 5.0 eq) in anhydrous methanol (15 mL). The crude product was purified by flash chromatography (EtOAc/hexanes, 30-70%) to yield **27** (154 mg, 529 µmol, 93%) as a yellow oil.

TLC: R_f = 0.56 (50% EtOAc/hexanes) [UV/CAM].
^{1}H-NMR (400 MHz, CDCl$_3$): δ[ppm] = 8.18 (s, 1 H), 6.27 (s, 1 H), 5.46 (d, ^4J = 6.5 Hz, 1 H), 5.19 (d, ^2J = 12.9 Hz, 1 H), 5.09 (d, ^4J = 6.5 Hz, 1 H), 5.03 (d, ^2J = 12.9 Hz, 1 H), 3.52 (s, 3 H), 3.47 (s, 3 H), 2.95 (t, ^3J = 7.6 Hz, 2 H), 2.24 (td, $^{3,4}J$ = 7.6, 2.7 Hz, 2 H), 1.92 (t, ^4J = 2.7 Hz, 1 H), 1.84 (p, ^3J = 7.6 Hz, 2 H), 1.63 (p, ^3J = 7.6 Hz, 2 H).
^{13}C-NMR (100 MHz, CDCl$_3$): δ[ppm] = 153.1, 146.5, 136.3, 134.5, 106.3, 96.4, 84.6, 77.4, 70.1, 68.4, 57.1, 55.0, 31.8, 28.5, 28.0, 18.5.

HRMS (ESI) ($C_{16}H_{21}NO_4$ $[M+H]^+$) calcd.: 292.1549
found: 292.1542.

1-Methoxy-7-(methoxymethoxy)-6-{2-[1-(prop-2-yn-1-yl)-1*H*-1,2,3-triazol-4-yl]ethyl}-1,3-dihydrofuro[3,4-*c*]pyridine[23]

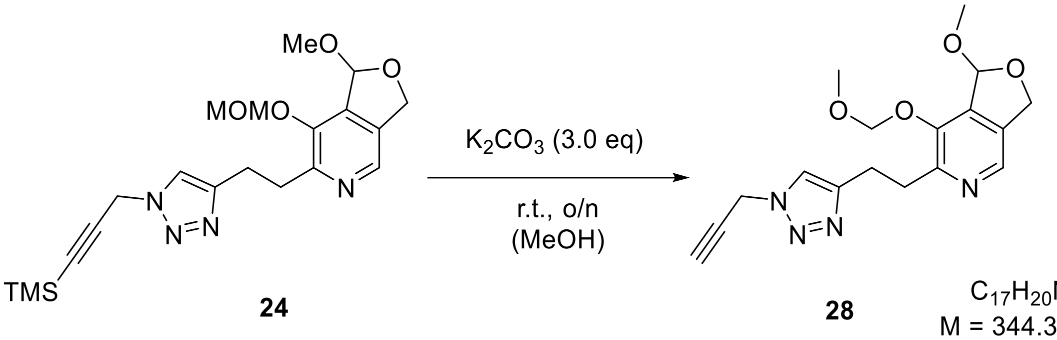

24 **28** $C_{17}H_{20}N_4O_4$
M = 344.37 g/mol

Following **GP8**, TMS-protected alkyne **24** (210 mg, 504 µmol, 1.0 eq) was converted with potassium carbonate (210 mg, 1.52 mmol, 3.0 eq) in anhydrous methanol (20 mL). The crude product was purified by flash chromatography (MeOH/DCM, 2-6%) to yield **28** (169 mg, 491 µmol, 98%) as a yellow oil.

TLC: R_f = 0.51 (EtOAc) [UV/KMnO$_4$].
^{1}H-NMR (400 MHz, CDCl$_3$): δ[ppm] = 8.18 (s, 1 H), 7.49-7.41 (m, 1 H), 6.26 (d, ^4J = 1.7 Hz, 1 H), 5.65 (d, ^4J = 6.6 Hz, 1 H), 5.43 (d, ^3J = 6.6 Hz, 1 H), 5.19 (d, ^2J = 13.3 Hz, 1 H), 5.12 (s, 2 H), 5.05 (d, ^4J = 6.6 Hz, 1 H), 5.03 (d, ^2J = 13.3 Hz, 1 H), 3.49 (s, 3 H), 3.47 (s, 3 H), 3.31-3.25 (m, 2 H), 3.23-3.15 (m, 2 H).
^{13}C-NMR (100 MHz, CDCl$_3$): δ[ppm] = 201.6, 152.0, 148.5, 146.4, 136.2, 135.5, 133.7, 118.9, 106.3, 98.7, 96.2, 88.8, 70.1, 57.0, 55.0, 32.1, 24.4.
HRMS (ESI) ($C_{17}H_{20}N_4O_4$ $[M+H]^+$) calcd.: 345.1557
found: 345.1551.

6-{2-[1-(But-3-yn-1-yl)-1*H*-1,2,3-triazol-4-yl]ethyl}-1-methoxy-7-(methoxymethoxy)-1,3-dihydrofuro[3,4-*c*]pyridine[23]

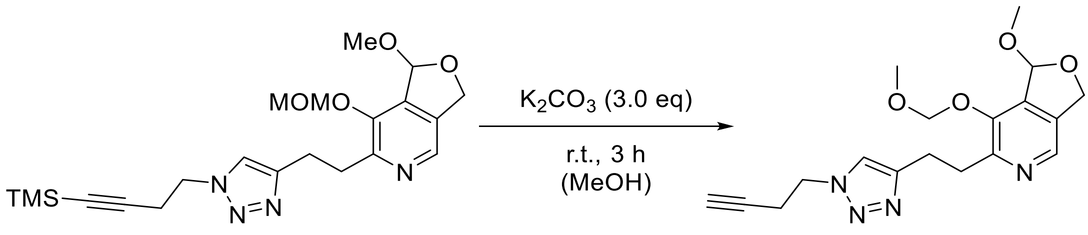

25 **29** $C_{18}H_{22}N_4O_4$
M = 358.39 g/mol

Following **GP8**, TMS-protected alkyne **25** (200 mg, 464 µmol, 1.0 eq) was converted with potassium carbonate (190 mg, 1.39 mmol, 3.0 eq) in anhydrous methanol (20 mL). The crude product was purified by flash chromatography (MeOH/DCM, 2-6%) to yield **29** (155 mg, 433 µmol, 93%) as a yellow oil.

TLC: R_f = 0.25 (EtOAc) [UV/KMnO$_4$].
^{1}H-NMR (400 MHz, CDCl$_3$): δ[ppm] = 8.19 (s, 1 H), 7.38 (s, 1 H), 6.25 (d, ^4J = 1.6 Hz, 1 H), 5.41 (d, ^4J = 6.6 Hz, 1 H), 5.19 (d, ^2J = 12.8 Hz, 1 H), 5.05 (d, ^4J = 6.6 Hz, 1 H), 5.03 (d, ^2J = 12.8 Hz, 1 H), 4.45 (t, ^3J = 6.8 Hz, 2 H), 3.49 (s, 3 H), 3.47 (s, 3 H), 3.31-3.23 (m, 2 H), 3.23-3.15 (m, 2 H), 2.75 (td, $^{3,4}J$ = 6.8, 2.7 Hz, 2 H), 2.05 (t, ^4J = 2.7 Hz, 1 H).
^{13}C-NMR (100 MHz, CDCl$_3$): δ[ppm]=152.2, 147.8, 146.4, 136.1, 135.6, 133.7, 121.6, 106.3, 96.2, 79.8, 71.5, 70.1, 57.0, 55.0, 48.7, 32.2, 24.5, 20.8.
HRMS (ESI) ($C_{18}H_{22}N_4O_4$ $[M+H]^+$) calcd.: 359.1719
found: 359.1710.

Acetal and Ether Deprotection, Final Probes

3-Hydroxy-5-(hydroxymethyl)-2-(pent-4-yn-1-yl)isonicotinaldehyde bisulfate[28]
PL6

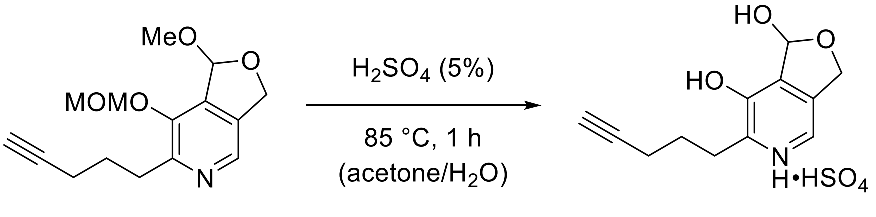

19 **30** $C_{12}H_{15}NO_7S$ M = 317.31 g/mol

Following **GP9**, MOM-protected acetal **19** (48.0 mg, 173 µmol, 1.0 eq) was deprotected in acetone/water (1:1; 5 mL) containing 5% sulfuric acid. After purification by HPLC (Method B), probe **PL6** (10.0 mg, 31.5 µmol, 19%) was obtained as a fluffy white solid.

TLC: R_f = 0.12 (50% EtOAc/hexanes) [UV/$KMnO_4$].
HPLC: t_R = 8.3 min (Method B).
^{1}H-NMR (400 MHz, d^6 DMSO): δ[ppm] = 9.60 (s, 1 H), 7.96 (s, 1 H), 6.64 (d, ^3J = 7.8 Hz, 1 H), 6.41 (d, ^3J = 7.8 Hz, 1 H), 5.04 (d, ^2J = 12.9 Hz, 1 H), 4.86 (d, ^2J = 12.9 Hz, 1 H), 2.81 (t, ^3J = 7.1 Hz, 2 H), 2.77 (t, ^4J = 2.6 Hz, 1 H), 2.21 (td, $^{3,4}J$ = 7.1, 2.6 Hz, 2 H), 1.81 (p, ^3J = 7.1 Hz, 2 H).
^{13}C-NMR (100 MHz, d^6 DMSO): δ[ppm] = 148.0, 145.7, 135.1, 133.7, 132.5, 98.5, 84.5, 71.3, 68.8, 30.3, 26.8, 17.6.
HRMS (ESI) ($C_{12}H_{13}NO_3$ $[M+H]^+$) calcd.: 220.0974
found: 220.0967.

2-(hex-5-yn-1-yl)-3-hydroxy-5-(hydroxymethyl)isonicotinaldehyde bisulfate[28]
PL7

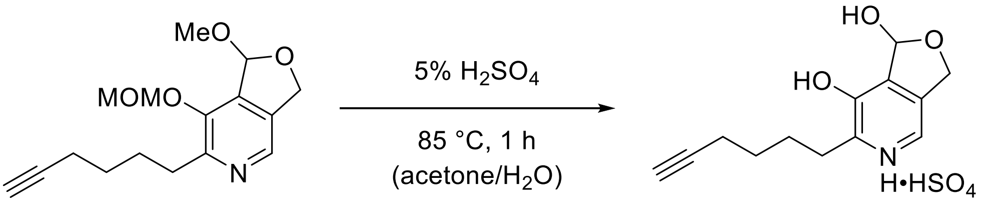

27 **31** $C_{13}H_{17}NO_7S$ M = 331.34 g/mol

Following **GP9**, MOM-protected acetal **27** (50.0 mg, 172 µmol, 1.0 eq) was deprotected in acetone/water (1:1; 10 mL) containing 5% sulfuric acid. After purification by HPLC (Method B), probe **PL7** (30.0 mg, 90.5 µmol, 55%) was obtained as a fluffy white solid.

TLC: R_f = 0.13 (50% EtOAc/hexanes) [UV/CAM].
HPLC: t_R = 6.0 min (Method B).
^{1}H-NMR (400 MHz, d^6 DMSO): δ[ppm] = 10.4 (s, 1 H), 8.03 (s, 1 H), 6.77 (br. s, 1 H), 6.45 (s, 1 H), 5.07 (d, ^2J = 13.0 Hz, 1 H), 4.89 (d, ^2J = 13.0 Hz, 1 H), 2.79 (t, ^3J = 7.4 Hz, 2 H), 2.73 (t, ^4J = 2.7 Hz, 1 H), 2.18 (td, $^{3,4}J$ = 7.4, 2.7 Hz, 2 H), 1.71 (p, ^3J = 7.4 Hz, 2 H), 1.48 (p, ^3J = 7.4 Hz, 2 H).
^{13}C-NMR (100 MHz, d^6 DMSO): δ[ppm] = 148.0, 146.3, 135.8, 135.3, 131.1, 98.4, 84.5, 71.2, 68.9, 30.1, 27.8, 27.1, 17.6.
HRMS (ESI) ($C_{13}H_{15}NO_3$ $[M+H]^+$) calcd.: 234.1125
found: 234.1121.

3-Hydroxy-5-(hydroxymethyl)-2-{2-[1-(prop-2-yn-1-yl)-1*H*-1,2,3-triazol-4-yl]ethyl} isonicotinaldehyde bisulfate[28]
PL8

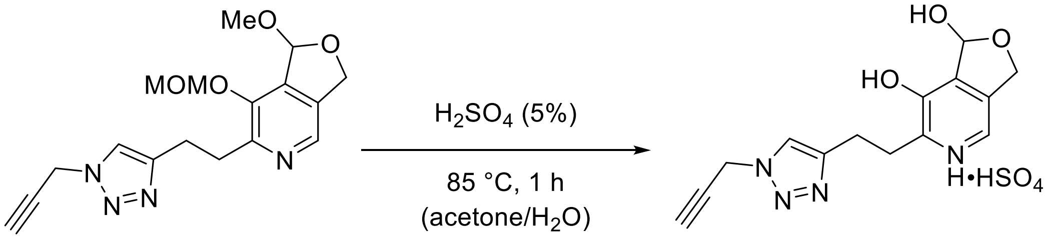

28 **32** $C_{14}H_{16}N_4O_7S$
M = 384.36 g/mol

Following **GP9**, MOM-protected acetal **28** (160 mg, 465 µmol, 1.0 eq) was deprotected in acetone/water (1:1; 10 mL) containing 5% sulfuric acid. After purification by HPLC (Method C), probe **PL8** (100 mg, 260 µmol, 56%) was obtained as a fluffy white solid.

TLC: R_f = 0.10 (EtOAc) [UV/CAM].
HPLC: t_R = 8.4 min (Method C).
^{1}H-NMR (400 MHz, d^6 DMSO): δ[ppm] = 9.70 (s, 1 H), 7.98 (s, 1 H), 7.91 (s, 1 H), 7.77 (t, ^3J = 6.5 Hz, 1 H), 6.67 (d, ^3J = 7.7 Hz, 1 H), 6.42 (dd, $^{3,4}J$ = 7.7,1.7 Hz, 1 H), 5.84 (d, ^3J = 6.5 Hz, 2 H), 5.05 (d, ^2J = 13.0 Hz, 1 H), 4.87 (d, ^2J = 13.0 Hz, 1 H), 3.15-3.05 (m, 2 H), 3.05-2.97 (m, 2 H).
^{13}C-NMR (100 MHz, d^6 DMSO): δ[ppm] = 201.3, 147.7, 147.5, 145.8, 135.3, 133.8, 132.5, 120.0, 98.5, 97.6, 88.9, 68.8, 30.9, 23.6.
HRMS (ESI) ($C_{14}H_{14}N_4O_3$ $[M+H]^+$) calcd.: 287.1144
found: 287.1136.

2-{2-[1-(but-3-yn-1-yl)-1*H*-1,2,3-triazol-4-yl]ethyl}-3-hydroxy-5-(hydroxymethyl) isonicotinaldehyde bisulfate[28]
PL9

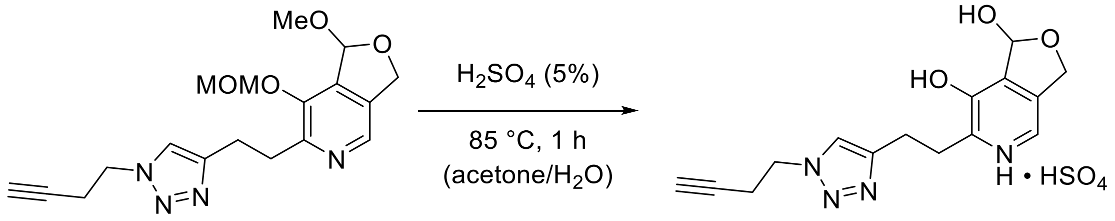

29 **33** $C_{15}H_{18}N_4O_7S$
M = 398.39 g/mol

Following **GP9**, MOM-protected acetal **29** (150 mg, 419 µmol, 1.0 eq) was deprotected in acetone/water (1:1; 10 mL) containing 5% sulfuric acid. After purification by HPLC (Method C), probe **PL9** (86.0 mg, 216 µmol, 52%) was obtained as a fluffy white solid

TLC: R_f = 0.10 (EtOAc) [UV/$KMnO_4$].
HPLC: t_R = 8.3 min (Method C).
^{1}H-NMR (400 MHz, d^6 DMSO): δ[ppm] = 9.70 (s, 1 H), 7.99 (s, 1 H), 7.88 (s, 1 H), 6.67 (d, ^3J = 6.9 Hz, 1 H), 6.42 (d, ^3J = 6.9 Hz, 1 H), 5.05 (d, ^2J = 12.9 Hz, 1 H), 4.87 (d, ^2J = 12.9 Hz, 1 H), 4.42 (t, ^3J = 6.8 Hz, 2 H), 3.13-3.02 (m, 2 H), 3.02-2.05 (m, 2 H), 2.90 (t, ^4J = 2.7 Hz, 1 H), 2.75 (td, $^{3,4}J$ = 6.8, 2.7 Hz, 2 H).
^{13}C-NMR (100 MHz, d^6 DMSO): δ[ppm] = 147.6, 146.6, 145.7, 135.3, 133.8, 132.6, 122.0, 98.5, 80.7, 73.3, 68.8, 47.8, 31.2, 23.8, 19.2.
HRMS (ESI) ($C_{15}H_{16}N_4O_3$ $[M+H]^+$) calcd.: 301.1301
found: 301.1293.

Acetal and Ester Deprotection, Final Probes

2-Ethynyl-5-hydroxy-3-(hydroxymethyl)-6-methyl isonicotinaldehyde bisulfate[28]
PL10

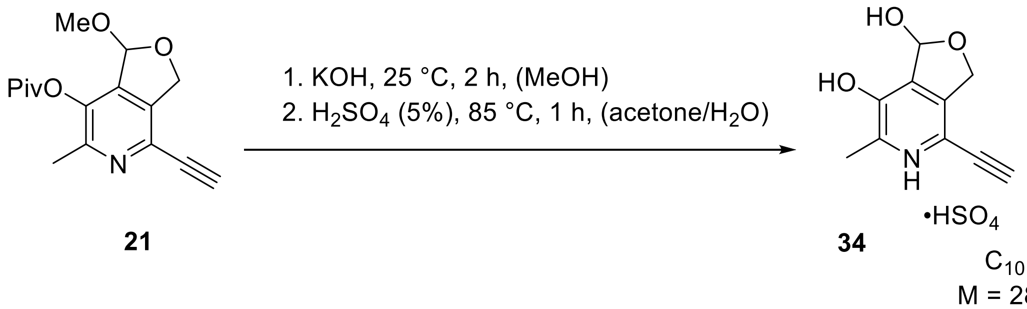

21 **34** $C_{10}H_{11}NO_7S$ M = 289.26 g/mol

Following **GP10**, Piv-protected acetal **21** (169 mg, 584 µmol, 1.0 eq) was deprotected first in 2% potassium hydroxide in methanol (5 mL) followed by deprotection in acetone/water (1:1; 10 mL) containing 5% sulfuric acid. After purification by HPLC (Method C), probe **PL10** (78.0 mg, 270 µmol, 47%) was obtained as a fluffy white solid.

TLC: R_f = 0.16 (10% MeOH/DCM) [UV/$KMnO_4$].
HPLC: t_R = 6.2 min (Method C).
^{1}H-NMR (400 MHz, d^6 DMSO): δ[ppm] = 10.2 (br. s, 1 H), 6.76 (br. s, 1 H), 6.42 (s, 1 H), 5.03 (d, ^2J = 13.4 Hz, 1 H), 4.85 (d, ^2J = 13.4 Hz, 1 H), 4.27 (s, 1 H), 2.35 (s, 3 H).
^{13}C-NMR (100 MHz, d^6 DMSO): δ[ppm] = 147.0, 146.6, 138.7, 133.3, 124.1, 99.1, 81.4, 81.0, 69.2, 18.9.
HRMS (ESI) ($C_{10}H_9NO_3$ $[M+H]^+$) calcd.: 192.0661
found: 192.0654.

3-Hydroxy-5-(hydroxymethyl)-2-methyl-6-[1-(prop-2-yn-1-yl)-1*H*-1,2,3-triazol-4-yl] isonicotinaldehyde bisulfate[28]
PL11

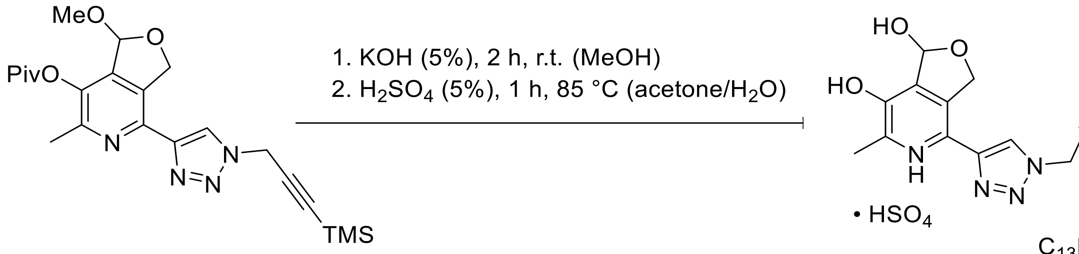

26 **35** $C_{13}H_{14}N_4O_7S$ M = 370.34 g/mol

Following **GP10**, crude Piv-protected acetal **26** (1.0 eq) was deprotected first in 5% potassium hydroxide in methanol (7 mL) followed by deprotection in acetone/water (1:1; 8 mL) containing 5% sulfuric acid. After purification by HPLC (Method B), probe **PL11** (17.0 mg, 45.9 µmol, 14% over 2 steps) was obtained as a fluffy white solid.

TLC: R_f = 0.39 (50% EtOAc/hexanes) [UV/$KMnO_4$].
HPLC: t_R = 9.6 min (Method B).
^{1}H-NMR (400 MHz, d^6 DMSO): δ[ppm] = 9.84 (s, 1 H), 8.42 (s, 1 H), 7.84 (t, ^4J = 5.2 Hz, 1 H), 6.69 (d, ^3J = 8.0 Hz, 1 H), 6.45 (dd, $^{3,4}J$ = 8.0, 1.7 Hz, 1 H), 5.92 (d, ^3J = 5.2 Hz, 2 H), 5.33 (d, ^2J = 13.8 Hz, 1 H), 5.18 (d, ^2J = 13.8 Hz, 1 H), 2.42 (s, 3 H).
^{13}C-NMR (100 MHz, d^6 DMSO): δ[ppm] = 201.7, 148.0, 145.9, 145.8, 134.6, 132.8, 131.9, 119.9, 98.1, 97.5, 89.3, 70.7, 18.9.
HRMS (ESI) ($C_{13}H_{12}N_4O_3$ $[M+H]^+$) calcd.: 273.0988
found: 273.0980.

2-(But-3-yn-1-yl)-5-hydroxy-3-(hydroxymethyl)-6-methylison icotinaldehyde bisulfate[28]
PL12

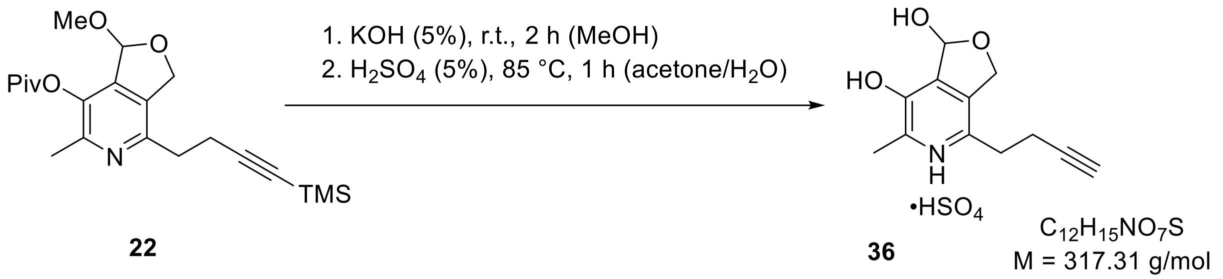

22 **36**

Following **GP10**, Piv-protected acetal **22** (400 mg, 1.03 mmol, 1.0 eq) was deprotected first in 2% potassium hydroxide in methanol (8 mL) followed by deprotection in acetone/water (1:1; 10 mL) containing 5% sulfuric acid. After purification by HPLC (Method B), probe **PL12** (78.0 mg, 246 µmol, 24%) was obtained as a fluffy white solid.

TLC: R_f = 0.08 (20% EtOAc/hexanes) [UV/$KMnO_4$].
HPLC: t_R = 5.5 min (Method B).
^{1}H-NMR (400 MHz, d^6 DMSO): δ[ppm] = 9.42 (s, 1 H), 6.68 (d, ^3J = 6.7 Hz, 1 H), 6.41 (d, ^3J = 6.7 Hz, 1 H), 5.07 (d, ^2J = 12.8 Hz, 1 H), 4.90 (d, ^2J = 12.8 Hz, 1 H), 2.74 (t, ^4J = 2.6 Hz, 1 H), 2.69 (t, ^3J = 7.4 Hz, 2 H), 2.49-2.44 (m, 2 H), 2.35 (s, 3 H).
^{13}C-NMR (100 MHz, d^6 DMSO): δ[ppm] = 144.9, 144.7, 141.6, 134.0, 133.0, 98.8, 84.2, 71.4, 68.9, 33.1, 18.7, 17.3.
HRMS (ESI) ($C_{12}H_{13}NO_3$ $[M+H]^+$) calcd.: 220.0974
found: 220.0967.

PL13 Synthesis

6-methyl-7-(prop-2-yn-1-yloxy)-1,3-dihydrofuro[3,4-c]pyridin-1-ol[36]
PL13

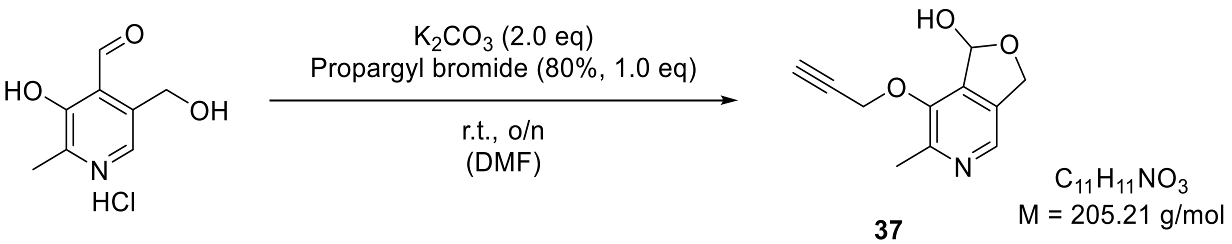

37

Pyridoxal hydrochloride (500 mg, 2.46 mmol, 1.0 eq) was dissolved in anhydrous dimethyl formamide (25 mL). Then, potassium carbonate (635 mg, 4.91 mmol, 2.0 eq) was added and the mixture was stirred for 15 min prior to the addition of propargyl bromide (80% in hexanes, 274 µL, 365 mg, 2.46 mmol, 1.0 eq). After stirring the reaction overnight, the solvent was evaporated under reduced pressure and the crude mixture was purified by column chromatography (MeOH/DCM, 5-10%) to yield **PL13** (126 mg, 614 µmol, 25%) as an off-white solid.

TLC: R_f = 0.44 (10% MeOH/DCM) [UV/CAM].
^{1}H-NMR (400 MHz, d^6 DMSO): δ[ppm] = 8.15 (s, 1 H), 7.10 (d, ^3J = 7.7 Hz, 1 H), 6.58 (dd, $^{3,4}J$ = 7.7, 1.7 Hz, 1 H), 5.07 (d, ^2J = 13.0 Hz, 1 H), 4.95 (dd, $^{4,4}J$ = 2.4, 1.1 Hz, 2 H), 4.92 (d, ^2J = 13.0 Hz, 1 H), 3.62 (t, ^4J = 2.4 Hz, 1 H).
^{13}C-NMR (100 MHz, d^6 DMSO): δ[ppm] = 149.1, 146.5, 136.5, 136.0, 135.7, 98.9, 79.1, 78.8, 68.6, 58.9, 19.3.
HRMS (ESI) ($C_{11}H_{12}NO_3$ $[M+H]^+$) calcd.: 206.0812
found: 206.0811.

The analytical data are in accordance with previous literature reports.[36]

Synthesis of Screening Compounds

Trimethyl{[1-(3-nitrophenyl)vinyl]oxy}silane[37]

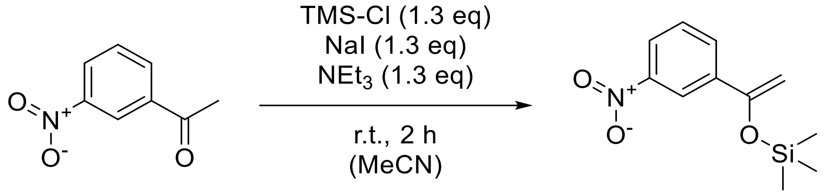

38 **39**

$C_{11}H_{15}NO_3Si$
M = 237.33 g/mol

m-Nitroacetophenone (991 mg, 6.00 mmol, 1.0 eq), trimethylsilyl chloride (951 µL, 7.50 mmol, 1.3 eq), and triethylamine (1.05 mL, 7.50 mmol, 1.3 eq) were added to a flame-dried flask. The mixture was stirred at room temperature and a solution of sodium iodide (1.12 g, 7.50 mmol, 1.3 eq) in acetonitrile (7.50 mL, 1.0 M) was added dropwise. After stirring the reaction for 2 h, it was cooled to 0 °C and hexanes (6 mL) was added. After adding water (6 mL) the phases were separated, and the aqueous layer was extracted with hexanes (2×6 mL). The combined organic phases were washed with water (2×6 mL), dried over magnesium sulfate and concentrated under reduced pressure. The intermediate trimethylsilyl enol ether **39** (973 mg, 4.10 mmol, 69%) was obtained as a yellow oil that was used without further purification.

3,3-Dichloro-1-(3-nitrophenyl)prop-2-en-1-one (MAC173979)[37]

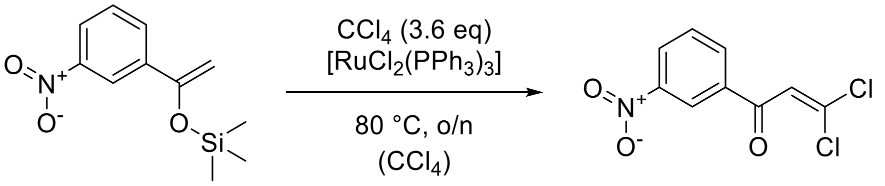

39 **40**

$C_9H_5Cl_2NO_3$
M = 246.04 g/mol

Carbon tetrachloride (1.40 mL, 14.5 mmol, 3.6 eq) was added to a flame-dried pressure tube, and the vessel was sealed with a silicon septum. The solvent was frozen at – 78 °C and vacuum was applied to 0.3 mbar. The solvent was then allowed to thaw under vacuum before the vessel was filled with argon. This freeze-pump-thaw process was repeated twice. Ruthenium(II) tris-triphenylphosphine dichloride (46.0 mg, 480 µmol, 1 mol%) and crude trimethyl{[1-(3-nitrophenyl)vinyl]oxy}silane (973 mg, 4.10 mmol, 1.0 eq) were added. The vessel was sealed and heated at 80 °C for 17 h while stirring. Upon completion, the reaction was cooled down and directly purified by column chromatography (EtOAc/hexanes, 5-20%) to yield dichlorovinyl **40** (415 mg, 1.69 mmol, 42%) as a white powder.
TLC: R_f = 0.54 (20% EtOAc/hexanes) [UV/$KMnO_4$].
^{1}H-NMR (400 MHz, d^6 DMSO): δ[ppm] = 8.69 (*virt.* t, $^3J \approx {}^3J$ = 1.9 Hz, 1 H), 8.50 (ddd, $^{3,4,4}J$ = 8.2, 2.3, 1.0 Hz, 1 H), 8.44 (ddd, $^{3,4,4}J$ = 7.8, 1.7, 1.1 Hz, 3 H), 8.03 (s, 1 H), 7.83-7.89 (m, 1 H).
^{13}C-NMR (100 MHz, d^6 DMSO): δ[ppm] = 184.7, 148.2, 137.6, 134.7, 134.6, 130.8, 128.0, 124.7, 122.9.

The analytical data are in accordance with previous literature reports.[37]

(3-Phenylpropyl)hydrazine trifluoroacetate salt (TM-2-10)

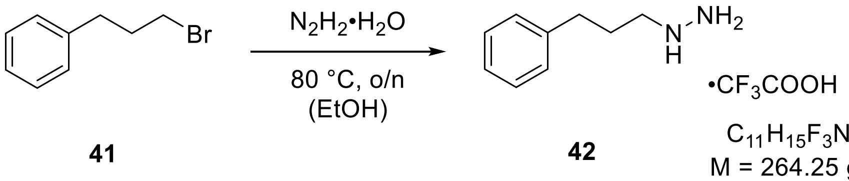

41 **42**

$C_{11}H_{15}F_3N_2O_2$
M = 264.25 g/mol

The title compound was synthesized from 3-phenylpropyl bromide (76.0 µL, 99.5 mg, 500 µmol, 1.0 eq) according to **GP11**. The crude product was purified by preparative reversed-phase HPLC, with an applied gradient as following: 2% MeCN (+0.1% TFA) to 98% in 12 min, 98% MeCN (+0.1% TFA) for 2 min, to 2% MeCN (+0.1% TFA) in 1 min, 2% MeCN (+0.1% TFA) for 2 min yielding **42** (32.6 mg, 123 µmol, 25%) as an off-white solid.

HPLC: t_R = 6.0 min (Method A modified).
^{1}H-NMR (300 MHz, CD_3OD): δ[ppm] = 7.27-7.31 (m, 2 H), 7.17-7.23 (m, 3 H), 3.03 (t, ^3J = 7.8 Hz, 2 H), 2.71 (t, ^3J = 7.6 Hz, 2 H), 1.96 (q, ^3J = 7.8 Hz, 2 H).
^{13}C-NMR (125 MHz, CD_3OD): δ[ppm] = 161.6 (q, J = 34 Hz, CF_3CO_2H)†, 140.5, 128.2, 128.0, 125.9, 116.8 (q, J = 290 Hz, CF_3CO_2H)†, 50.6, 32.2, 26.5.
^{19}F-NMR (377 MHz, CD_3OD): δ[ppm] = -76.9 (s, CF_3CO_2H).
HRMS (ESI) ($C_9H_{15}N_2$ $[M+H]^+$) calcd.: 151.1230
found: 151.1232.

The analytical data are in accordance with previous literature reports.[30]
†CF_3CO_2H peaks poorly characterised due to low signal intensity

(3,4-Dimethoxyphenethyl)hydrazine trifluoroacetate salt (TM-2-11)

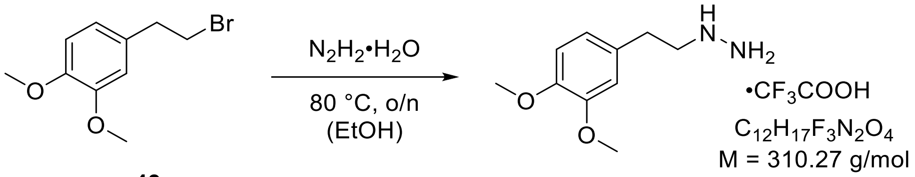

43 **44**

The title compound was synthesized from 4-(2-bromoethyl)-1,2-dimethoxybenzene (40.0 mg, 163 µmol, 1.0 eq) according to **GP11**. Purification by preparative reversed-phase HPLC, with an applied gradient as following: 25% MeCN (+0.1% TFA) to 50% in 12 min, 50% MeCN (+0.1% TFA) for 2 min, to 2% MeCN (+0.1% TFA) in 1 min, 2% MeCN (+0.1% TFA) for 2 min yielding **44** (16.5 mg, 53.0 µmol, 33%) as an off-white solid.

HPLC: t_R = 4.0 min (Method A modified).
^{1}H-NMR (300 MHz, CD_3OD): δ[ppm] = 8.29 (s, 1 H), 7.92 (s, 1 H), 6.79-6.82 (m, 1 H), 6.59-6.66 (m, 2 H), 4.51 (t, ^3J = 6.9 Hz, 2 H), 3.77 (s, 3 H), 3.73 (s, 3 H), 3.12 (t, ^3J = 6.9 Hz, 2 H).
^{13}C-NMR (125 MHz, CD_3OD): δ[ppm] = 149.1, 148.1, 130.0, 120.9, 112.1, 111.6, 55.0, 54.9, 45.4, 35.0.
^{19}F-NMR (377 MHz, CD_3OD): δ[ppm] = -76.9 (s, CF_3CO_2H).

N-(3-(2-Hydrazineylethyl)phenyl)acetamide ditrifluoroacetate salt (TM-2-12)

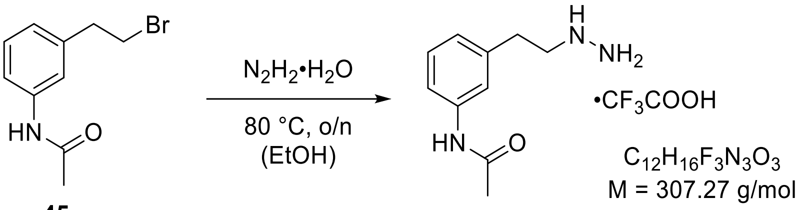

45 **46**

The title compound was synthesized from N-(3-(2-bromoethyl)phenyl)acetamide (40.0 mg, 165 µmol, 1.0 eq) according to **GP11**. Purification by preparative reversed-phase HPLC yielded **46** (19.8 mg, 64.0 µmol, 39%) as a yellow oil.

HPLC: t_R = 6.0 min (Method A).
^{1}H-NMR (400 MHz, CD_3OD): δ[ppm] = 7.58 (s, 1 H), 7.24-7.33 (m, 2 H), 7.00-7.03 (m, 1 H), 3.27 (t, ^3J = 7.6 Hz, 2 H), 2.93 (t, ^3J = 7.6 Hz, 2 H), 2.12 (s, 3 H).
^{13}C-NMR (125 MHz, CD_3OD): δ[ppm] = 170.4, 138.9, 137.7, 128.9, 124.1, 120.2, 118.5, 52.0, 31.4, 22.4.
^{19}F-NMR (377 MHz, CD_3OD): δ[ppm] = -76.9 (s, CF_3CO_2H).
HRMS (ESI) ($C_{10}H_{16}N_3O$ $[M+H]^+$) calcd.: 194.1288
found: 194.1294.

((4-(tert-Butoxy)phenethyl)hydrazine trifluoroacetate salt (TM-2-13)

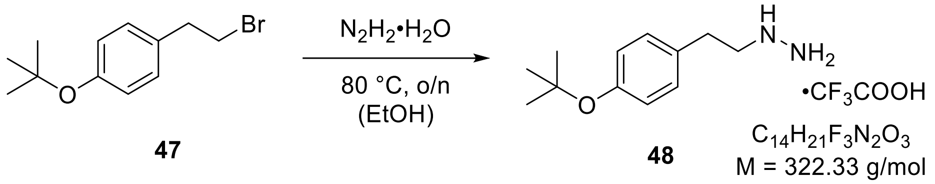

47 **48**

$C_{14}H_{21}F_3N_2O_3$
M = 322.33 g/mol

The title compound was synthesized from 1-(2-bromoethyl)-4-(tert-butoxy)benzene (40.0 mg, 156 µmol, 1.0 eq) according to **GP11**. Purification by preparative reversed-phase HPLC yielded **48** (36.1 mg, 112 µmol, 72%) as a white solid.

HPLC: t_R = 9.0 min (Method A).
^{1}H-NMR (400 MHz, CD_3OD): δ[ppm] = 7.17-7.20 (m, 2 H), 6.95-6.98 (m, 2 H), 3.24 (t, ^3J = 8.0 Hz, 2 H), 2.91 (t, ^3J = 8.0 Hz, 2 H), 1.32 (s, 9 H).
^{13}C-NMR (125 MHz, CD_3OD): δ[ppm] = 161.7 (q, J = 35 Hz, CF_3CO_2H)†, 154.1, 132.0, 128.9, 124.3, 116.8 (q, J = 290 Hz, CF_3CO_2H)†, 78.2, 52.2, 30.5, 27.8.
^{19}F-NMR (377 MHz, CD_3OD): δ[ppm] = -77.0 (s, CF_3CO_2H).
HRMS (ESI) ($C_{12}H_{21}N_2O$ $[M+H]^+$) calcd.: 209.1648
found: 209.1654.
†CF_3CO_2H peaks poorly characterised due to low signal intensity

(2-(Trifluoromethyl)phenethyl)hydrazine trifluoroacetate salt (TM-2-14)

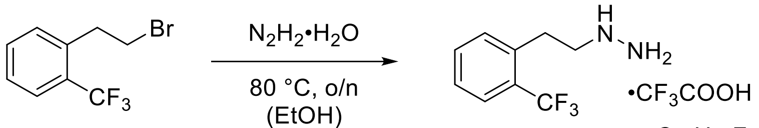

49 **50**

$C_{11}H_{12}F_6N_2O_2$
M = 318.22 g/mol

The title compound was synthesized from 1-(2-bromoethyl)-2-(trifluoromethyl)benzene (40.0 mg, 158 µmol, 1.0 eq) according to **GP11**. Purification by preparative reversed-phase HPLC yielded **50** (27.2 mg, 86.0 µmol, 54%) as a white solid.
HPLC: t_R = 8.5 min (Method A).
^{1}H-NMR (400 MHz, CD_3OD): δ[ppm] = 7.70-7.72 (m, 1 H), 7.59-7.63 (m, 1 H), 7.43-7.51 (m, 2 H), 3.22-3.26 (m, 2 H), 3.11-3.15 (m, 2 H).
^{13}C-NMR (125 MHz, CD_3OD): δ[ppm] = 161.5 (q, J = 35 Hz, CF_3CO_2H), 135.8, 132.3, 131.5, 128.4 (q, J = 30 Hz, CF_3), 127.1, 125.8 (q, J = 5 Hz, CF_3), 124.7 (q, J = 270 Hz, CF_3), 116.7 (q, J = 289 Hz, CF_3CO_2H), 51.5, 28.6.
^{19}F-NMR (377 MHz, CD_3OD): δ[ppm] = -60.9, -77.0.
HRMS (ESI) ($C_9H_{12}F_3N_2$ $[M+H]^+$) calcd.: 205.0947
found: 205.0952.

2-(2-Hydrazineylethyl)pyridine ditrifluoroacetate salt (TM-2-15)

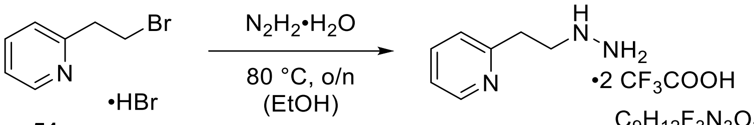

51 **52**

$C_9H_{12}F_3N_3O_2$
M = 251.21 g/mol

The title compound was synthesized from 2-(2-bromoethyl)pyridine hydrogenbromide (40.0 mg, 150 µmol, 1.0 eq) according to **GP11**. Purification by preparative reversed-phase HPLC yielded **52** (46.1 mg, 126 µmol, 84%) as a red solid.
HPLC: t_R = 3.0 min (Method A).
^{1}H-NMR (400 MHz, CD_3OD): δ[ppm] = 8.72 (d, ^3J = 5.6 Hz, 1 H), 8.32 (td, $^{3,4}J$ = 8.0, 1.6 Hz, 1 H), 7.87 (d, ^3J = 8.0 Hz, 1 H), 7.77 (t, ^3J = 5.6 Hz, 1 H), 3.49 (t, ^3J = 6.8 Hz, 2 H), 3.35 (t, ^3J = 6.8 Hz, 2 H)†.
^{13}C-NMR (125 MHz, CD_3OD): δ[ppm] = 161.7 (q, J = 34 Hz, CF_3CO_2H), 155.6, 144.2, 143.0, 126.2, 124.1, 115.6 (q, J = 291 Hz, CF_3CO_2H), 48.7, 31.2.
^{19}F-NMR (377 MHz, CD_3OD): δ[ppm] = -76.9 (s, CF_3CO_2H).
HRMS (ESI) ($C_7H_{12}N_3$ $[M+H]^+$) calcd.: 138.1026
found: 138.1021.

†Overlap of compound peak with CD_3OD reference quintet prevents solvent peak from being observed.

3-(2-Hydrazineylethyl)-2-methyl-6,7,8,9-tetrahydro-4H-pyrido[1,2-a]pyrimidin-4-one ditrifluoroacetate salt (TM-2-16)

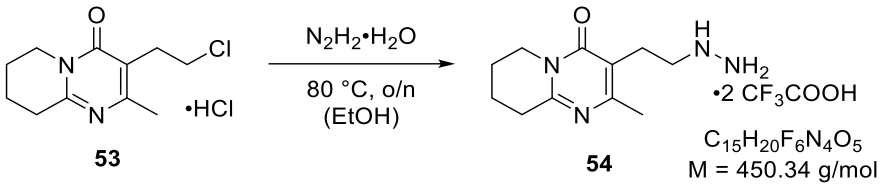

53 **54**

The title compound was synthesized from 3-(2-chloroethyl)-2-methyl-4*H*,6*H*,7*H*,8*H*,9*H*-pyridol[1,2-a]pyrimidin-4-one hydrogenchloride (40.0 mg, 152 µmol, 1.0 eq) according to **GP11**. Purification by preparative reversed-phase HPLC, with fraction collection utilising the 275 nm trace, yielded **54** (58.3 mg, 130 µmol, 85%) as a pale yellow oily residue.

HPLC: t_R = 4.5 min (Method A).

^{1}H-NMR (400 MHz, CD_3OD): δ[ppm] = 3.97 (t, ^3J = 6.0 Hz, 2 H), 3.25 (t, ^3J = 6.8 Hz, 2 H), 3.10 (t, ^3J = 6.8 Hz, 2 H), 2.92 (t, ^3J = 6.8Hz, 2 H), 2.43 (s, 3 H), 2.02-2.08 (m, 2 H), 1.92-1.99 (m, 2 H).

^{13}C-NMR (125 MHz, CD_3OD): δ[ppm] = 161.6 (q, J = 34 Hz, CF_3CO_2H), 161.2, 160.6, 152.8, 117.8, 116.8 (q, J = 290 Hz, CF_3CO_2H), 48.6, 43.6, 21.9, 20.5, 17.1, 17.0, 16.8.

^{19}F-NMR (377 MHz, CD_3OD): δ[ppm] = -76.9–76.8 (m, CF_3CO_2H).

HRMS (ESI) ($C_{11}H_{19}N_4O$ $[M+H]^+$) calcd.: 223.1553
found: 223.1561.

(2-(2,2-Difluorobenzo[d][1,3]dioxol-4-yl)ethyl)hydrazine trifluoroacetate salt (TM-2-17)

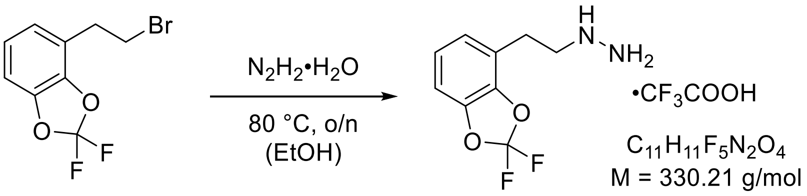

55 **56**

The title compound was synthesized from 4-(2-bromoethyl)-2,2-difluoro-1,3-dioxaindane (40.0 mg, 151 µmol, 1.0 eq) according to **GP11**. Purification by preparative reversed-phase HPLC yielded **56** (33.3 mg, 101 µmol, 67%) as a white solid.

HPLC: t_R = 9.5 min (Method A).

^{1}H-NMR (400 MHz, CD_3OD): δ[ppm] = 7.04-7.16 (m, 3 H), 3.29 (t, ^3J = 7.6 Hz, 2 H), 3.02 (t, ^3J = 7.6 Hz, 2 H).

^{13}C-NMR (125 MHz, CD_3OD): δ[ppm] = 161.7 (q, J = 35 Hz, CF_3CO_2H), 143.4, 142.0, 131.4 (t, J = 251 Hz, CF_2), 124.7, 124.0, 120.2, 116.8 (q, J = 290 Hz, CF_3CO_2H), 108.0, 49.5, 26.4.

^{19}F-NMR (377 MHz, CD_3OD): δ[ppm] = -52.0 (s, 2 F), -77.0 (s, 3 F).

HRMS (ESI) ($C_9H_{11}F_2N_2O_2$ $[M+H]^+$) calcd.: 217.0783
found: 217.0788.

(4-(2-Hydrazineylethyl)benzonitrile trifluoroacetate salt (TM-2-18)

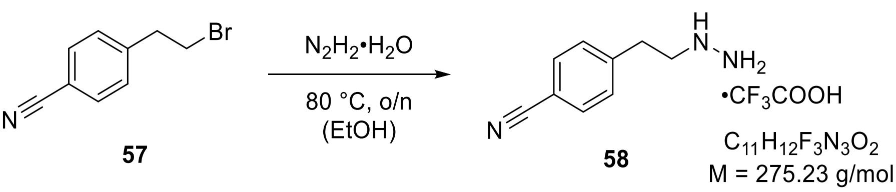

57 **58**

The title compound was synthesized from 4-(2-bromoethyl)benzonitrile (40.0 mg, 190 µmol, 1.0 eq) according to **GP11**. Purification by preparative reversed-phase HPLC yielded **58** (20.1 mg, 73.0 µmol, 38%) as an off-white, yellow solid.

HPLC: t_R = 6.8 min (Method A).

^{1}H-NMR (300 MHz, CD_3OD): δ[ppm] = 7.69-7.72 (m,2 H), 7.45-7.48 (m, 2 H), 3.27 (t, ^3J = 7.8 Hz, 2 H), 3.02 (t, ^3J = 7.8 Hz, 2 H).

^{13}C-NMR (125 MHz, CD_3OD): δ[ppm] = 132.2, 129.5, 125.4, 118.2, 110.5, 50.9, 33.1.

^{19}F-NMR (377 MHz, CD_3OD): δ[ppm] = -76.9 (s, CF_3CO_2H).
HRMS (ESI) ($C_9H_{12}N_3$ $[M+H]^+$) calcd.: 162.1026
found: 162.1025.

(4-(2-Hydrazineylethyl)-N,N-dimethylbenzenesulfonamide trifluoroacetate salt (TM-2-19)

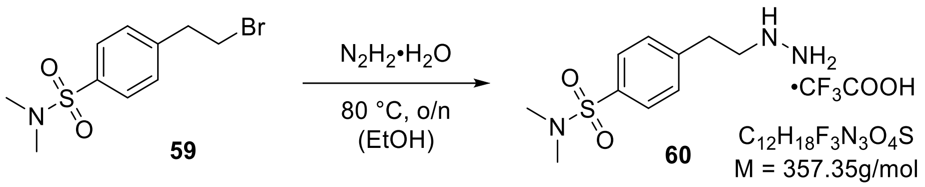

The title compound was synthesized from 4-(2-bromoethyl)-*N,N*-dimethylbenzene-1-sulfonamide (40.0 mg, 137 µmol, 1.0 eq) according to **GP11**. Purification by preparative reversed-phase HPLC yielded **58** (36.3 mg, 102 µmol, 74%) as a white solid.
HPLC: t_R = 7.0 min (Method A).
^{1}H-NMR (400 MHz, CD_3OD): δ[ppm] = 7.75 (d, ^3J = 8.0 Hz, 2 H), 7.53 (d, ^3J = 8.0 Hz, 2 H), 3.29 (t, ^3J = 7.6 Hz, 2 H), 3.04 (t, ^3J = 7.6 Hz, 2 H), 2.68 (s, 6 H).
^{13}C-NMR (125 MHz, CD_3OD): δ[ppm] = 161.6 (q, J = 35 Hz, CF_3CO_2H), 143.2, 133.7, 129.3, 128.0, 116.8 (q, J = 289 Hz, CF_3CO_2H), 108.0, 51.1, 36.9, 31.4.
^{19}F-NMR (377 MHz, CD_3OD): δ[ppm] = -77.0 (s, CF_3CO_2H).
HRMS (ESI) ($C_{10}H_{18}N_3O_2S$ $[M+H]^+$) calcd.: 244.1114
found: 244.1122.

PL-Methoxim[38]

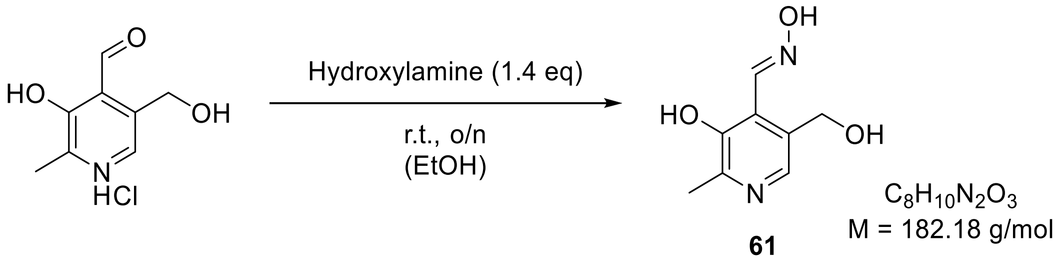

Hydroxylamine hydrochloride (478 mg, 6.88 mmol,1.4 eq) was added to a stirred solution of pyridoxal hydrochloride (1.00 g, 4.91 mmol, 1.0 eq) in ethanol (100 mL). The reaction was stirred at room temperature overnight. Upon completion, the solvent was removed under reduced pressure and the residue was purified by HPLC (Method C) to yield methoxim **61** (508 mg, 2.79 mmol, 57%) as a white solid.
TLC: R_f = 0.69 (10% MeOH/DCM) [UV/CAM].
HPLC: t_R = 1.6 min (Method C).
^{1}H-NMR (400 MHz, d^6 DMSO): δ[ppm] = 8.64 (s, 1 H), 8.20 (s, 1 H), 4.73 (s, 2 H), 4.06 (s, 3 H), 3.78 (s, 1 H), 2.62 (s, 3 H).
HRMS (ESI) ($C_8H_{11}N_2O_3$ $[M+H]^+$) calcd.: 183.0765
found: 183.0763.

Biological Methods

Culture Media

Table SI 17: Buffer and media.

Buffer/Medium	Ingredients
**LB-Medium**	Peptone (10 g), NaCl (5 g), yeast extract (5 g) in 1 L ddH_2O<br>pH = 7.5
**B-Medium**	Peptone (10 g), NaCl (5 g), yeast extract (5 g), K_2PO_4 (1 g) in 1 L ddH_2O<br>pH = 7.5
**CDM_{SA}**	Adapted from [39]
**CDM_{EC}**	Adapted from [40]
**CDM_{PA}**	Adapted from [41]

Cloning and Overexpression of Proteins

PCR and Gene Purification

S. aureus, *E. coli* and *P. aeruginosa* genes were amplified by polymerase chain reaction (PCR) according to **Table SI 18** and

Table SI *19*. Primers and their annealing temperatures are given in **Table SI 20**. After verification of the reactions by an 1% agarose gel, PCR Products were purified using a E.Z.N.A.® MicroElute Cycle Pure Kit (*Omega*) according to the manufacturers protocol.

Table SI 18: PCR conditions.

	Volume
**Forward Primer (10 µM)**	2.5 µL
**Reverse Primer (10 µM)**	2.5 µL
**dNTPs (10 mM)**	1 µL
**Genomic DNA (150 ng/µL)**	1 µL
**5x Phusion HF Buffer**	10 µL
**Phusion DNA Polymerase**	0.5 µL
**DMSO**	(1.5 µL if necessary)
**ddH_2O**	Fill up to 50 µL

Table SI 19: Thermocyclic conditions.

Step	Temperature [° C]	Time [s]	Cycles
**Initial Denaturation**	98	30	1
**Denaturation**	98	10	
**Annealing**	See **Table SI 20**	30	35
**Extension**	72	30	
**Final Extension**	72	10 min	1
**Hold**	4	∞	

Table SI 20: Primer list.

Primer	Gene	Organism	Sequence	Annealing Temperature [° C]	Company
**Gateway pdxK fwd**	pdxK	E. coli	ggggacaagtttgtacaaaaaagcaggcttt AGTAGTTTGTTGTTGTTTAACGATAAG	61	Eurofins
**Gateway pdxK rev**	pdxK	E. coli	ggggaccactttgtacaagaaagctgggtg TTATGCTTCCGCCAGCG		Eurofins
**Gateway ydcR fwd**	ydcR	E. coli	ggggacaagtttgtacaaaaaagcaggcttt AAAAAATACCAGCAGCTTGC	60	Eurofins
**Gateway ydcR rev**	ydcR	E. coli	ggggaccactttgtacaagaaagctgggtg TTACAGCCGTTCTTGAATAAG		Eurofins
**Gateway yjiR fwd**	yjiR	E. coli	ggggacaagtttgtacaaaaaagcaggcttt TACGCGTTATCAACATCTGG	59	Eurofins
**Gateway yjiR rev**	yjiR	E. coli	ggggaccactttgtacaagaaagctgggtg TTATTCCATTGCCCGATACAC		Eurofins
**SLIM MBP tailed**			GCCCTGAAAATAAAGATTCTCGCC AGAAGTCTGCGCGTCTTTCAG	68	Sigma Aldrich
**SLIM MBP short**			AGTCTGCGCGTCTTTCAGGGCTTC		Sigma Aldrich
**SLIM ydcR tailed**		E. coli	TCTGGCGAGAATCTTTATTTTCAGGG CAAAAAATACCAGCAGCTTGC	68	Sigma Aldrich
**SLIM ydcR short**		E. coli	AAAAAATACCAGCAGCTTGC		Sigma Aldrich
**PRydcR-ydcS fwd**	ydcR-ydcS intergenic region	E. coli	[TAMRA]TAGCGTTTAATTTAATTCCTCTTAG	59	Sigma Aldrich
**PRydcR-ydcS rev**	ydcR-ydcS intergenic region	E. coli	[TAMRA]TAAGGTCTTACTCCTGTCG		Sigma Aldrich
**PRydcR fwd**	ydcR promotor region	E. coli	[TAMRA]GGCGCTTGGCAAAGAGTTATC	59	Sigma Aldrich
**PRydcR rev**	ydcR promotor region	E. coli	[TAMRA]GCAACGAAGGCAAACGATCG		Sigma Aldrich
**Gateway iscS fwd**	iscS	E. coli	ggggacaagtttgtacaaaaaagcaggcttt AAATTACCGATTTATCTCGACTACTC	61	Sigma Aldrich
**Gateway iscS rev**	iscS	E. coli	ggggaccactttgtacaagaaagctgggtg TTAATGATGAGCCCATTCGATG		Sigma Aldrich
**Gateway iscU fwd**	iscU	E. coli	ggggacaagtttgtacaaaaaagcaggcttt GCTTACAGCGAAAAAGTTATC	59	Sigma Aldrich
**Gateway iscU rev**	iscU	E. coli	ggggaccactttgtacaagaaagctgggtg TTATTTTGCTTCACGTTTGC		Sigma Aldrich
**Gateway A0A0H2XHJ5 fwd**	sufS	S. aureus	ggggacaagtttgtacaaaaaagcaggcttt GCCGAACACTCATTTGAC	61	Sigma Aldrich
**Gateway A0A0H2XHJ5 rev**	sufS	S. aureus	ggggaccactttgtacaagaaagctgggtg TTAAAATTCATAAGAGAAAAACTCCTTC		Sigma Aldrich

**Gateway sufU fwd**	sufU	S. aureus	ggggacaagtttgtacaaaaaagcaggcttt AATTTTAATAATCTAGATCAATTATATAGATCTG	59	Sigma Aldrich
**Gateway sufU rev**	sufU	S. aureus	ggggaccactttgtacaagaaagctgggtg CTATTCTTCTTCAGTCGTACC		Sigma Aldrich
**Gateway A0A0H2XII6 fwd**	A0A0H2XII6	S. aureus	Ggggacaagtttgtacaaaaaagcaggcttt AAGCAACCTATTTTAAATAAATTAGAAAG	55	Sigma Aldrich
**Gateway A0A0H2XII6 rev**	A0A0H2XII6	S. aureus	ggggaccactttgtacaagaaagctgggtg TTATTCATCCTCAACTAAAATTTTATTATTTTTA		Sigma Aldrich
**Gateway PA2683 fwd**	PA2683	P. aeruginosa	ggggacaagtttgtacaaaaaagcaggcttt CACGACCTGCCTACCTACGACG	68	Sigma Aldrich
**Gateway PA2683 rev**	PA2683	P. aeruginosa	ggggaccactttgtacaagaaagctgggtg CTAGCCGCCGAGCAAGGC		Sigma Aldrich
**Gateway PA3659 fwd**	PA3659	P. aeruginosa	ggggacaagtttgtacaaaaaagcaggcttt AATACCGCACTCGACAACCTGC	68	Sigma Aldrich
**Gateway PA3659 rev**	PA3659	P. aeruginosa	ggggaccactttgtacaagaaagctgggtg TCAGCGCGCCCGCAG		Sigma Aldrich
**Gateway PA3798 fwd**	PA3798	P. aeruginosa	ggggacaagtttgtacaaaaaagcaggcttt ATTCAGAGCAAGCTGCCGAATG	66	Sigma Aldrich
**Gateway PA3798 rev**	PA3798	P. aeruginosa	ggggaccactttgtacaagaaagctgggtg TCAGATCGCGCATAGCTTTTCC		Sigma Aldrich

Gateway® Cloning

Cloning was performed using the *Invitrogen* Gateway® cloning system with pDONR™201$_{Kan}$ (*Invitrogen*) or pDONR™207$_{Gen}$ (*Invitrogen*) as the donor vectors and pET-55-DEST™$_{Amp}$ (*Invitrogen*), pETG-41K$_{Kan}$ (*EMBL*) or pDEST007$_{Amp}$ (custom made) as the destination vectors. Protocols were adopted from *Invitrogen*[42]. Expression vectors were transformed into *E. coli* BL21 (DE3).

Site-directed and Ligation Independent Mutagenesis (SLIM)

SLIM was chosen to introduce a TEV (Tobacco Etch Virus) protease cleavage site between the maltose binding protein (MBP) and the protein ydcR. For this, two PCR reactions were performed: one with a tailed MBP primer (SLIM MBP tailed) and the short one for ydcR (SLIM ydcR short) and one PCR with the short MBP primer (SLIM MBP short) and the long one for ydcR (SLIM ydcR tailed). PCR reactions were conducted as discussed before with additional DMSO (3%), an annealing temperature of 68 °C and an elongated extension time of 2.5 min. PCR products were verified by 1% agarose gels and sequencing (*Genewiz*) and purified as described in section 0.
The destination vector was digested with 1 µL *Dpn I* in 5 µL of CutSmart® buffer (*NEB*) at 37 °C for 1 h. Then, complementary TEV sites were hybridized by taking 20 µL of the respective two PCR fragments and 10 µL of H buffer (750 mM NaCl, 125 mM TRIS,100 mM EDTA, pH = 9.0). The reaction was conducted at 99 °C for 3 min and two cycles at 65 °C for 5 min, followed by 30 °C for 40 min. The final vector was transformed into *E. coli* BL21 (DE3).

Protein Overexpression

LB-media containing 0.1 mg/mL ampicillin (100 mg/mL stock in ethanol/ddH_2O = 1/1) or 25 µg/mL kanamycin (25 mg/mL stock in ddH_2O) was inoculated with 1:100 overnight culture of the corresponding vector containing *E. coli* BL21 (DE3) strain. After growth at 37 °C to an OD_{600} of 0.5-0.6, 1 mM *iso*-propyl-1-thio-β-galactopyranoside (IPTG, 1 M stock in ddH_2O) except otherwise stated for pET-55-DEST™Amp and pETG-41K$_{Kan}$ or 2 µg/mL anhydrotetracycline (aTet, 2 mg/mL stock in ddH_2O) for pDEST007$_{Amp}$ were added and the protein expression was carried out at the temperature corresponding to **Table SI 21**. Bacteria were then harvested (6000 rpm, 10 min, 4 °C, rotor SLA-3000; Sorvall RC 6+, *Thermo Scientific*) and washed with 30 mL PBS once prior to cell lysis and protein purification.

Table SI 21: Proteinexpression conditions.

Protein Name	Gene Name	Uniprot ID	Overexpression	MW [Da] found (-Met)	Tags
**Pyridoxine/pyridoxal/pyridoxamine kinase**	pdxK	P40191	18 °C, o/n	33,137.0	N-STREP-II
**Uncharacterized HTH-type transcriptional regulator**	ydcR	P77730	18 °C, o/n, 0.25 mM IPTG	95,690.5	ydcR-TEV-MBP-C-His
**Uncharacterized HTH-type transcriptional regulator**	yjiR	P39389	18 °C, o/n, 0.25 mM IPTG	95,942.0	yjiR-MBP-C-His
**Cysteine desulfurase**	iscS	P0A6B7	18 °C, o/n	47,422.5	N-STREP-II
**Iron-sulfur cluster assembly scaffold protein**	iscU	P0ACD4	18 °C, o/n 2 µg/mL aTet	16,126.0	N-STREP-II
**Probable cysteine desulfurase**	sufS	A0A0H2XHJ5	18 °C, o/n	48,565.0	N-STREP-II
**NifU family SUF system FeS assembly protein**	SAUSA300_0821	A0A0H2XJC0	18 °C, o/n 2 µg/mL aTet	19,294.5	N-STREP-II
**Orn/Lys/Arg decarboxylase**	SAUSA300_0458	A0A0H2XII6	18 °C, o/n	53,370.5	N-STREP-II
**Probable serine/threonine dehydratase, degradative**	PA2683	Q9I0F5	18 °C, o/n	37,099.0	N-STREP-II
**Probable aminotransferase**	PA3659	Q9HXY0	25 °C, o/n	47,685.0	N-STREP-II
**Probable aminotransferase**	PA3798	Q9HXJ9	18 °C, o/n	45,736.0	N-STREP-II

Protein Purification of STREP Tagged Proteins

Cell pellets from 2 L culture were resuspended in 20 mL STREP binding buffer (50 mM NaH_2PO_4, 300 mM NaCl, pH = 7.9) and lysed by sonication (7 min 30%, 3 min 70%, 2 cycles; *Bandelin* Sonolus HD 2070). The lysate was clarified by centrifugation (18000 rpm, 45 min, 4 °C, SLA-3000) and loaded onto a with STREP binding buffer pre-equilibrated StrepTrap column (*GE Healthcare*) integrated into a Äkta purifier10 FPLC system (*GE Healthcare*). Untagged proteins were removed by washing the column with 10 column volumes STREP binding buffer at a flow of 3 mL/min. Elution of STREP-tagged proteins was conducted by applying 5 column volumes STREP elution buffer (STREP binding buffer, 2.5 mM desthiobiotin, pH = 7.9). The eluted fractions were collected, concentrated with a suitable centrifugal mass filter (MWCO: 3/10/30/50 kDa, *Millipore*) and desalted into STREP binding buffer by loading the proteins onto a HiTrap Desalting column (*GE Healthcare*). Eluates were combined, concentrated and protein aliquots were snap frozen in liquid nitrogen and stored at -80 °C. Protein concentrations were measured at 280 nm on a *Tecan* Infinite® M Nano plate reader in a NanoQuant plate™.

Purification of ydcR and yjiR

Protein purification of recombinant ydcR and yjiR using a HisTrap HP 5 mL column (*GE Healthcare*) for Ni-NTA affinity purification and a HiTrap Desalting 5 mL column (*GE Healthcare*) for desalting. First, the HisTrap HP 5 mL column was equilibrated with His-Tag purification buffer A (50 mM NaH_2PO_4, 300 mM NaCl, 5 mM DTT, 20 mM imidazole, pH = 8.0). After loading, non-specifically bound proteins were removed by washing with 14 column volumes buffer A. Then, the tagged proteins were eluted with 4 column volumes 100% His-Tag purification buffer B (50 mM NaH_2PO_4, 300 mM NaCl, 5 mM DTT, 300 mM imidazole, pH = 8.0). The protein containing elution fractions were concentrated (MWCO: 50 kDa) and loaded onto equilibrated HiTrap Desalting 5 ml column with buffer C (50 mM NaH_2PO_4, 300 mM NaCl, 5 mM DTT, pH = 8.0). Proteins were eluted with five column volumes of desalting buffer. Eluates were combined, concentrated and protein aliquots were snap frozen in liquid nitrogen and stored at -80 °C. Protein concentrations were measured at 280 nm on a *Tecan* Infinite® M Nano plate reader in a NanoQuant plate™.

UV/VIS Measurements [21]

PLP-dependent enzymes which are bound to PLP absorb at wavelengths which depend on the protonation state of the internal imine.[21,43] First, the enzymes were diluted to 100 µM in PBS and their absorption was recorded at room temperature on an Infinite® M Nano by *Tecan Trading AG* from 300 to 600 nm with 5 nm increments. To determine possible substrates such as amino acids, proteins were incubated with 500 µM of PLP and 1 mM of substrate for 15 min prior to the measurement. A control sample with 25 mM hydroxylamine for generation of the apo-enzymes was performed, too.

Probe Phosphorylation Studies with pdxK and SaPLK

Qualitative phosphorylation of probes by pdxK and SaPLK was determined by UV/Vis-spectroscopy measuring the decrease of absorbance of unphosphorylated species at around 320 nm and the increase of absorbance of phosphorylated species at around 395 nm.
To a solution containing 2 µM pdxK or SaPLK and 1 mM PL-probe, ATP was added to a final concentration of 1 mM in 100 µL kinase-buffer (50 mM HEPES, 50 mM KCl, 10 mM $MgCl_2$, pH = 7.9). The assays were conducted in a 96-well plate (Nunclon™ Delta Surface, *Thermo Scientific*) and absorbance spectra were recorded from 285 to 600 nm every 40 min on a *Tecan* Infinite® M Nano plate reader at 37 °C.

Loading State Studies via Intact Protein Mass Spectrometry (IP-MS)

To 10 µM protein in PBS (25 µL), 2 µL of 250 mM $NaBH_4$ (fresh in 0.1 M NaOH) or PBS (enzyme only) were added, and the mixture was incubated for 30 min at room temperature. The reaction was stopped by adding 8 µL 0.5% formic acid and neutralized with 5 µL 0.1 M NaOH. All samples were then diluted to 50 µL with PBS in order to obtain 5 µM protein solutions. Measurements were conducted as described in the next paragraph.

Intact Protein MS

IP-MS measurements were carried out on an Ultimate 3000 RSLC system (*Thermo Scientific*) coupled to an LTQ Orbitrap XL mass spectrometer (*Thermo Scientific*).
Protein desalting was performed using a MassPREP desalting column (*Waters*) at 25 °C. Gradient elution was carried out with 0.1% formic acid (LC-MS grade, *Fisher Analytic*) in water (LC-MS grade, *Fisher Analytic*) (A) and 0.1% formic acid in acetonitrile (MeCN, LC-MS grade, *Fisher Analytic*) (B). After 2 min pre-equilibration with 6% B, protein samples were injected and eluted with a linear gradient from 6% to 95% B over 1.5 min and 2 min at 95% B at 300 µL/min flow rate. The column was re-equilibrated with 6% B for 1 min.
Mass spectrometric measurements were conducted in HESI positive mode (H-ESI-II source, *Thermo Scientific*) with the following parameters: 4.0 kV capillary voltage, 350 °C capillary temperature, 31 V capillary voltage, 110 V tube lens, 30 L/h sheath gas, 15 L/h aux gas. Full scan measurements were accomplished in a range from 300-2000 m/z in profile mode in the orbitrap at a resolution of 100,000. Raw spectra were processed with *UniDec* 2.6.7 for deconvolution.

Assay for PA2683 Activity

Kinetics of PA2683 were measured by a coupled assay, where the product pyruvate gets reduced by lactate dehydrogenase (LDH) while consuming one equivalent of nicotinamide adenine dinucleotide (NADH), causing a decrease of NADH absorbance at 340 nm.
Reaction solutions of 2 µM PA2683, 25 µg LDH (5 mg/mL stock solution, *Roche*), 100 µM PLP (1 µL of a 10 mM stock in ddH_2O), 6 mM adenosine monophosphate (AMP, 3 µL of a 300 mM stock in ddH_2O), 2.5 mM NADH (5 µL of a 50 mM stock in ddH_2O) and varying concentrations of L-Ser (1, 2, 5, 10, 20, 50, 100, 200, 400, 600, 800, 1000, 1500, 2000, 2500 mM, stocks in ddH_2O) in a total volume of each 100 µL in buffer (10 mM TRIS, 1 mM $MgCl_2$, pH = 7.5), were transferred into a 96-well plate (Nunclon™ Delta Surface, *Thermo Scientific*) and absorbance at 340 nm was recorded every 30 s on a *Tecan* Infinite® M Nano plate reader at 37 °C with shaking intervals 5 s prior to measurement. All conditions were performed in triplicates with a series of control experiments (at 600 mM L-Ser): w/o NADH, w/o PA2638, w/o L-Ser, w/o LDH, w/o AMP, w/o PLP.
Reaction rates and MICHAELIS-MENTEN kinetics were calculated in *Graphpad* Prism 5.03 considering the following parameters:

$E = \varepsilon \cdot c \cdot d \leftrightarrow c = E/(d \cdot \varepsilon)$
for NADH $1/(d \cdot \varepsilon)$ = 535.91 $\mu mol/L$ at 340 nm with $\varepsilon = 6.22\ 1/M \cdot cm$ [44]
with d = 0.3 cm for 100 µL in 96-well plate

Graphpad Prism 5.03 output table:

Michaelis-Menten	
Best-fit values	
Vmax	1,253
Km	602,2
Std. Error	
Vmax	0,06191
Km	82,11
95% Confidence Intervals	
Vmax	1,119 to 1,387
Km	424,8 to 779,6
Goodness of Fit	
Degrees of Freedom	13
R square	0,9881
Absolute Sum of Squares	0,02312
Sy.x	0,04217
Constraints	
Km	Km > 0,0
Number of points	
Analyzed	15

EMSA – Electrophoretic Mobility Shift Assay

The protocol was adapted from *Kunzmann et al*. with modifications.[45]
The TAMRA labelled ydcR promoter fragment PR_{ydcR} and the intergenic region fragment $PR_{ydcR\text{-}ydcS}$ (see **Table SI 20**) of *E. coli* K12 were generated as described in chapter 0. Proteins were diluted in 11 µL EMSA binding buffer (100 mM TRIS, 150 mM NaCl, 1 mM EDTA, 5% v/v glycerol, pH = 8.0) to the following concentrations: 2, 5, 10, 25, 50, 75, 100, 200, 400 nM. Control samples were performed at 200 nM protein concentration. PLP control samples were diluted in 10.8 µL instead of 11 µL. Then, 0.5 µL salmon sperm DNA (4 mg/mL stock in ddH_2O, *Carl Roth*) and 0.5 µL PR_{ydcR} or $PR_{ydcR\text{-}ydcS}$ (20 ng/µL stock in ddH_2O, 10 ng total) were added. To PLP control samples, 0.2 µL PLP (120 µM sock in ddH_2O, 2 µM total) were further added. After incubation for 20 min at room temperature, 2 µL of loading buffer (EMSA binding buffer, 0.1% bromphenol blue) were added to the samples and they were loaded onto a NativePAGE™ 3-12% Bis-Tris polyacrylamide gel (*Invitrogen*, 150 V, 90 min). Fluorescence imaging was conducted on a *GE Healthcare* ImageQuant™ LAS-4000 equipped with a 575DF20 Cy3 filter.

Growth Studies

Table SI 22: Bacterial strains.

Strain	Source	Antibiotic Pressure
***S. aureus* USA300 TnpdxS**	Nebraska Transposon Mutant Library within the Network on Antimicrobial Resistance in Staphylococcus aureus (NARSA)[5]	Erythromycin 5 µg/mL
***E. coli* K12 MG1655 ΔpdxJ**	Kindly provided by Prof. Kirsten Jung from the LMU Originated from the Keio Collection[46]	Kanamycin 25 µg/mL
***P. aeruginosa* PAO1**	Institute Pasteur in France	/
***S. aureus* USA300**	Nebraska Transposon Mutant Library within the Network on Antimicrobial Resistance in Staphylococcus aureus (NARSA)[5]	/
***E. coli* K12 MG1655**	Keio Collection[46]	/

First, minimal concentration of PL or probe supplementation in chemically defined media (CDM) in order to reach normal growth was determined. Therefore, an overnight culture of *E .coli* K12 ΔpdxJ was grown in LB-medium containing 25 µg/mL kanamycin (25 mg/mL stock in ethanol) and washed with CDM_{EC} containing kanamycin (2 x 5 mL) the next day. Bacteria were resuspended in CDM_{EC} containing kanamycin to an OD_{600} = 0.08. To each well containing 198 µL bacterial suspension in a 96-well plate (Nunclon™ Delta Surface, *Thermo Scientific*), different concentrations of PL (2 µL; 1, 10, 250, 500 nM final) or probe (2 µL; 1, 10, 100, 250 µM final) were added and growth was recorded by measuring the absorbance at 600 nm on a *Tecan* Infinite® M Nano plate reader at 37 °C over 24 h with shaking intervals prior to the measurements. All conditions were performed in quadruplicates. Sterile controls (duplicates) were added for each condition as blanks.
Growth curves for larger cultures (100 mL in 500 mL flasks or 500 mL in 2 L buffled flasks containing 250 nM PL and the corresponding antibiotic as given in **Table SI 22**) were recorded manually by measuring the OD_{600} every hour. Flasks were incubated at 37 °C with shaking (200 rpm). Early stationary phases were reached in CDM after 8 h for *E. coli* K12 ΔpdxJ, after 6 h for *P. aeruginosa* PAO1 and after 10 h for *S. aureus* USA300 TnpdxS. For comparison reasons, growth curves for *S. aureus* USA300 TnpdxS in B-medium were also performed. Here, early stationary phase was reached after 6 h.

Proteomics

Analytical Labelling

Analytical labelling experiments were conducted as described previously with modifications.[21]
E. coli K12 ΔpdxJ were inoculated from an overnight culture 1:100 in 100 mL CDM_{EC} containing kanamycin (25 µg/mL) and 0.25 µM pyridoxal (25 µL of a 1 mM stock in DMSO). Bacteria were grown for 10 h at 37 °C, harvested (6000 x *g*, 4 °C, 10 min) and washed with CDM_{EC} containing kanamycin (2 x 5 mL). Pelletised bacteria were then resuspended in CDM_{EC}, adjusted to an OD_{600} = 40 and 200 µL of the suspension was either incubated with 2 µL probe (10 mM stock in DMSO) or DMSO for 2 h at 37 °C with shaking. Labelled cells were harvested (6000 x *g*, 4 °C, 10 min) and washed with cold PBS (2 x 500 µL). For the lysis, cells were resuspended in 200 µL PBS and sonicated (30 s at 80%, 3 cycles; *Bandelin* Sonolus HD 2070). Lysate was clarified by centrifugation (21,000 x *g*, 30 min, 4 °C) and the supernatant (150 µL) was transferred into a fresh tube for reduction with sodium borohydride (6 µL of 500 mM stock, freshly made in 0.1 M NaOH). After incubation for 30 min at room temperature, proteins were precipitated by adding 600 µL cold acetone (-80 °C). Proteins were then pelletized by centrifugation (21,000 x *g*, 4 °C, 20 min) and washed with cold methanol (2 x 500 µL) with resuspending steps by sonication (10 s at 10%, 1 cycle). Protein pellets were finally resuspended in 100 µL 0.4% SDS in PBS and incubated for 30 min at room temperature with a click chemistry mix (5 µL 10 mM BTTAA in ddH_2O, 2 µL 50 mM $CuSO_4$ in ddH_2O, 2 µL 5 mM rhodamine-azide in DMSO and 2 µL 100 mM sodium ascorbate freshly prepared in ddH_2O). Before loading 50 µL to a 12.5% SDS-polyacrylamide gel (150 V, 3.5 h), samples were treated with 100 µL loading buffer.

Preparative Labelling

Proteomics were conducted as described previously with modifications.[21]

S. aureus USA300 TnpdxS Growth, Labelling and Lysis

S. aureus USA300 TnpdxS overnight cultures were grown in 300 mL flasks containing 50 mL B-medium and 5 µg/mL erythromycin (25 µL of a 10 mg/mL stock in ethanol). After harvesting (6000 x *g*, 4 °C, 10 min), pellets were washed with CDM_{SA} containing 5 µg/mL erythromycin (2 x 10 mL) and adjusted to an OD_{600} = 6. Large cultures of 500 mL CDM_{SA} containing 5 µg/mL erythromycin and 0.25 µM PL (50 µL of a 2.5 mM stock in DMSO) were then inoculated with 1:100 overnight culture and incubated for 10 h at 37 °C with shaking. Bacteria were harvested (6000 x *g*, 4 °C, 10 min), washed with CDM_{SA} containing 5 µg/mL erythromycin (2 x 20 mL) and diluted with CDM_{SA} to an OD_{600} = 40. To 1 mL bacterial suspension, 100 µM probe (10 µL of a 10 mM stock in DMSO) or 10 µL DMSO were added and incubated for 2 h at 37 °C with shaking. After labelling, bacteria were harvested (6000 x *g*, 4 °C, 10 min) and washed with cold PBS (2 x 1 mL). Lysis was performed by addition of lysostaphin (5 µL of a 10 mg/mL stock in 20 mM NaOAc, pH = 4.5, *Sigma Aldrich*, Lysostaphin from *Staphylococcus simulans*) and incubation for 1 h at 37 °C and 1400 rpm shaking (*Eppendorf* Thermomixer® R). The resulting lysate was clarified by centrifugation (20,000 x *g*, 30 min, 4 °C) and the supernatant (900 µL) was transferred into a fresh 15 mL falcon prior to reduction.
For competitive labelling experiments, bacteria were treated with phenelzine (75, 100, 1000 µM, from 20 x stocks in ddH_2O), benserazide (0.1, 1, 10 mM, from 20 x stocks in ddH_2O) or CCG-50014 (100, 500, 2500 µM, from 100 x stocks in DMSO) for 30 min at 37 °C with shaking, prior to probe labelling.

E. coli K12 ΔpdxJ Growth, Labelling and Lysis

E. coli K12 ΔpdxJ overnight cultures were grown in 300 mL flasks containing 50 mL LB-medium and 25 µg/mL kanamycin (50 µL of a 25 mg/mL stock in ddH_2O). After harvesting (6000 x *g*, 4 °C, 10 min), pellets were washed with CDM_{EC} containing 25 µg/mL kanamycin (2 x 10 mL) and adjusted to an OD_{600} = 20. Large cultures of 500 mL CDM_{EC} containing 25 µg/mL kanamycin and 0.25 µM PL (50 µL of a 2.5 mM stock in DMSO) were then inoculated with 1:100 overnight culture and incubated for 8 h at 37 °C with shaking. Bacteria were harvested (6000 x *g*, 4 °C, 10 min), washed with CDM_{EC} containing 25 µg/mL kanamycin (2 x 20 mL) and diluted with CDM_{EC} to an OD_{600} = 60. To 1 mL bacterial suspension, 100 µM probe (10 µL of a 10 mM stock in DMSO) or 10 µL DMSO were added and incubated for 2 h at 37 °C with shaking. After labelling, bacteria were harvested (6000 x *g*, 4 °C, 10 min) and washed with cold PBS (2 x 1 mL). Lysis was performed by sonication (30 s at 80%, 4 cycles; *Bandelin* Sonolus HD 2070) and the resulting lysate was clarified by centrifugation (20,000 x *g*, 30 min, 4 °C). The supernatant (900 µL) was transferred into a fresh 15 mL falcon prior to reduction.

P. aeruginosa PAO1 Growth, Labelling and Lysis

P. aeruginosa PAO1 wt overnight cultures were grown in 300 mL flasks containing 50 mL LB-medium. After harvesting (6000 x *g*, 4 °C, 10 min), pellets were washed with CDM_{PA} and adjusted to an OD_{600} = 20. Large cultures of 500 mL CDM_{PA} containing 0.25 µM PL (50 µL of a 2.5 mM stock in DMSO) were then inoculated with 1:100 overnight culture and incubated for 6 h at 37 °C with shaking. Bacteria were harvested (6000 x *g*, 4 °C, 10 min), washed with CDM_{PA} (2 x 20 mL) and diluted with CDM_{PA} to an OD_{600} = 40. To 1 mL bacterial suspension, 100 µM probe (10 µL of a 10 mM stock in DMSO) or 10 µL DMSO were added and incubated for 2 h at 37 °C with shaking. After labelling, bacteria were harvested (6000 x *g*, 4 °C, 10 min) and washed with cold PBS (2 x 1 mL). Lysis was performed by sonication (30 s at 80%, 4 cycles; *Bandelin* Sonolus HD 2070) and the resulting lysate was clarified by centrifugation (20,000 x *g*, 30 min, 4 °C). The supernatant (900 µL) was transferred into a fresh 15 mL falcon prior to reduction.

Reduction and BCA Assay

Lysates were reduced by adding 10 mM sodium borohydride (2 µL of 500 mM stock per 100 µL lysate freshly prepared in 0.1 m NaOH) and incubation for 30 min at room temperature. Subsequently, proteins were precipitated by adding 4x volume cold acetone (-80 °C) and stored at -20 °C for at least 2 h.
After pelletation of precipitated proteins by centrifugation (10,000 rpm,4 °C, 15 min, rotor SLA-3000; Sorvall RC 6+, *Thermo Scientific*), proteins were washed with cold methanol (-80 °C, 2 x 1 mL). Then, protein pellets were solubilised by adding 1 mL 0.4% SDS in PBS (w/v) and they were adjusted to the same concentration by a BCA assay (ROTI®Quant, *Roth*) in a total volume of 1 mL 0.4% SDS in PBS (w/v).

Click Chemistry

Samples were treated with 10 µL biotin-azide (10 mM stock in DMSO, *Sigma Aldrich*), 50 µL BTTAA (10 mM stock in ddH_2O, *Jena Bioscience*), 20 µL $CuSO_4$ (50 mM in ddH_2O) and 20 µL sodium ascorbate (100 mM stock freshly prepared in ddH_2O) and incubated for 1 h at room temperature. Proteins were precipitated by adding 4x volume cold acetone (-80 °C) and stored at -20 °C for at least 2 h. After pelletation of precipitated proteins by centrifugation (20,000 x *g*, 4 °C, 15 min), proteins were washed with cold methanol (-80 °C, 2 x 1 mL).

Staudinger Ligation

Samples labelled with **PL3** were treated with 20 µL EZ-Link™ Phosphine-PEG3-Biotin (10 mM stock in DMSO, *Sigma Aldrich*) and samples were incubated for 4 h at 37 °C with shaking, followed by 20 h at room temperature. Proteins were precipitated by adding 4x volume cold acetone (-80 °C) and stored at -20 °C for at least 2 h. After pelletation of precipitated proteins by centrifugation (20,000 x *g*, 4 °C, 15 min), proteins were washed with cold methanol (-80 °C, 2 x 1 mL).

MS Workflow

Protein pellets were resuspended in 1 mL 0.4% SDS in PBS (w/v) and centrifuged (20,000 x *g*, room temperature, 15 min) prior to avidin bead enrichment. 50 µL of avidin-agarose bead slurry (*Sigma Aldrich*) were pre-washed with 0.4% SDS in PBS (w/v) (3 x 1 mL, 400 x *g*, room temperature, 5 min) prior to protein loading and incubation for 1.5 h at room temperature with rotation. Avidin beads with bound proteins were washed with 0.4% SDS in PBS (w/v) (3 x 1 mL), 6 M urea in MS-H_2O (2 x 1 mL) and PBS (3 x 1 mL). After removal of supernatants, beads were resuspended in 200 µL X-buffer (7 M urea, 2 M thiourea in 20 mM 2-[4-(2-Hydroxyethyl)piperazin-1-yl]ethane-1-sulfonic acid (HEPES) buffer, pH = 7.5) and proteins were reduced with 5 mM tris(2-carboxyethyl)phosphine (TCEP, 2 µL of a 500 mM stock in MS-H_2O) for 1 h at 37 °C with shaking. Then, alkylation with 10 mM iodoacetamide [IAA, 4 µL of a 500 mM stock in 50 mM triethylammonium bicarbonate (TEAB) buffer] was conducted for 30 min at 25 °C before quenching samples with 10 mM dithiothreitol (DTT, 4 µL of a 500 mM stock in MS-H_2O) for 30 min at 25 °C. Proteins were then enzymatically digested using 1 µL LysC (0.5 µg/µL, MS-grade, *Fujifilm*) for 2 h at 25 °C, followed by dilution with 600 µL TEAB (50 mM) and digestion using 1.5 µL trypsin (0.5 µg/mL in 50 mM acetic acid buffer, sequencing grade, *Promega*) for additional 16 h at 37 °C. Reactions were stopped by adding 10 µL formic acid (Pierce™, *Thermo Scientific*) and beads were removed by centrifugation (17,000 x *g*, room temperature, 5 min). Prior to desalting using Sep-Pak® C18 cartridges (50 mg, *Waters*), cartridges were first pre-equilibrated with MeCN (1 x 1 mL), 80% MeCN containing 0.5% formic acid (2 x 1 mL) and 0.1% trifluoroacetic acid (3 x 1 mL). After sample loading, cartridges were washed with 0.1% trifluoroacetic acid (3 x 1 mL), 0.5% formic acid (1 x 500 µL) and peptides were eluted with 80% MeCN containing 0.5% formic acid (750 µL). Solvents were removed *in vacuo* (centrifugal vacuum concentrator, *Eppendorf*) and lyophilized peptides were dissolved in 25 µL 1% formic acid (v/v) and filtered through Ultrafree-MC centrifugal filters (*Merck*) prior to the transfer into LC-MS vials.
All proteomics experiments were simultaneously conducted in three biological replicates.

MS Measurement and Analysis

Proteomics samples were analysed on an Orbitrap Fusion mass spectrometer (*Thermo Scientific*) coupled to an Ultimate3000 nano-HPLC (*Thermo Scientific*, *Dionex*) equipped with an Acclaim™ PepMap™100 C18 75 µm x 2 cm trap (*Thermo Scientific*) and an Acclaim™ PepMap™ RSLC C18 separation column (75 µm x 50 cm; *Thermo Scientific*), both heated to 50 °C, coupled to an EASY-spray™ source. Samples were loaded onto the trap at a flow rate of 5 µL/min with 0.1% trifluoroacetic acid before being transferred to the separation column at 0.4 µL/min. Separation of samples was performed as following: Starting from 5% buffer B (0.1% formic acid in MeCN) and 95% buffer A (0.1% formic acid in H_2O) for 10 min, a linear gradient was applied from 5% to 22% buffer B in 105 min, followed by an increase of buffer B from 22% to 32% in 10 min and a subsequent increase to 90% buffer B in 10 min, which was kept at 90% buffer B for 10 min. After this isocratic flow, concentration of buffer B was decreased back to 5% within 0.1 min and held at this concentration for another 10 min (total time 152 min). MS full scans were recorded at 120,000 resolution in the orbitrap with the following parameters: Ion transfer tube temperature 275 °C, RF lens amplitude 60%, 350-1500 m/z scan range, $2.0e^5$ AGC target, 3 s cycle time and 50 ms maximal injection time. Peptides with an higher intensity than $5e^3$ and charge states from 2-7 were selected and fragmented in the higher-energy collisional dissociation (HCD) cell at 30% collision energy and analysed in the ion trap using the rapid scan rate. In the ion trap, following parameters were adjusted: isolation window 1.6 m/z, AGC target $1.0e^4$ and a maximal injection time of 100 ms.

Statistical Analysis of MS/MS Data

Proteomics raw data were analysed using MaxQuant (ver. 1.6.2.10)[47] which uses the Andromeda search engine.[48] Settings were default except for LFQ-quantification and match between runs, which were activated during search. The following UniProtKB databases were used for the searches: *E. coli* K12 (taxon identifier: 83333, downloaded 09.04.2019), *P. aeruginosa* PAO1 (taxon identifier: 208964, downloaded 14.04.2020) and *S. aureus* USA300 (taxon identifier: 367830, downloaded 04.11.2020).
Statistical analysis was conducted in Perseus (ver. 1.6.5.0). Protein-groups textfiles from the MaxQuant analysis were loaded into Perseus and first, LFQ-intensities were transformed (log_2). Further, protein contaminants, ones only identified by site modification and reverse hits were removed from the matrix. Sample replicates were then equally annotated and the matrix was filtered for 2 out of 3 valid values in at least one group. Missing values were imputated for the whole matrix using the following settings: width 0.3, down shift 1.8. Finally, *p*-values were calculated by a two-sided two sample *t*-test using a BENJAMINI-HOCHBERG false discovery rate correction (FDR, 0.05). Visualisation of data was realised using a scatter-plot [x-axis: student's *t*-test difference (probe/control or competitor plus probe/probe); y-axis: -log student's *t*-test *p*-value (probe/control or competitor plus probe/probe)].
Heatmaps were created in OriginPro® (ver. 9.7.0.185) by plotting LFQ-intensities against PLP-dependent enzymes.

Targeted Metabolomics Assays

Substrate Screen for PA2683

A 5 µM PA2683 solution in buffer (10 mM TRIS, 1 mM $MgCl_2$, pH = 7.5) was incubated at 37 °C for 30 min with 10 mM L-Ser, D-Ser, L-Thr or D-Thr (100 mM stocks in ddH_2O), 6 mM AMP (60 mM stock in ddH_2O) and 100 µM PLP (10 mM stock in ddH_2O) in a total volume of 100 µL. All conditions were conducted in triplicates and heat controls (protein pre-incubation at 95 °C for 5 min) were performed as negative controls. Samples were further treated as described in Further Sample Preparation.

Substrate Screen for PA3659 and PA3798

Substrate conversion was determined by quantifying transaminated aminoacids (α-ketoaminoacids) by LC-MS/MS. A 5 µM protein solution in buffer (10 mM TRIS, 1 mM $MgCl_2$, pH = 7.5) was incubated at 37 °C for 30 min with 10 mM L-Trp or L-Phe for PA3659 and L-Met or L-Gln for PA3798 (100 mM stocks in ddH_2O), 5 mM 2-ketoglutarate (100 mM stock in ddH_2O) and 100 µM PLP (10 mM stock in ddH_2O) in a total volume of 100 µL. All conditions were conducted in triplicates and heat controls (protein pre-incubation at 95 °C for 5 min) were performed as negative controls. Samples were further treated as described in Further Sample Preparation.

Inhibition Assay for A0A0H2XII6 with Phenelzine

Inhibition was determined by quantifying the enzymes product cadaverine by LC-MS/MS. For this, protein A0A0H2XII6 (5 µM) was preincubated for 40 min at 37 °C with different concentrations of phenelzine (1 mM, 100 µM, 50 µM, 25 µM, 12.5 µM; from 100x stocks in ddH_2O) in buffer (10 mM TRIS, 1 mM $MgCl_2$, pH = 7.5). To the solutions, 10 mM L-Lys (100 mM stock in ddH_2O) and 100 µM PLP (10 mM stock in ddH_2O) were added (final volume 100 µL) and the reactions were incubated at 37 °C for 30 min. All conditions were conducted in triplicates and a heat control (protein pre-incubation at 95 °C for 5 min) was performed as negative control. Samples were further treated as described in Further Sample Preparation.
Graphpad Prism 5.03: log(inhibitor) vs. response -- Variable slope (four parameters) output table:

Y=Bottom + (Top-Bottom)/(1+10^((LogIC50-X)*HillSlope))

log(inhibitor) vs. response -- Variable slope (four parameters)	
Best-fit values	
Bottom	= 0,0
Top	= 100,0
LogIC50	1,725
HillSlope	1,896
IC50	53,05
Span	= 100,0
Std. Error	
LogIC50	0,02970
HillSlope	0,2463
95% Confidence Intervals	
LogIC50	1,661 to 1,789
HillSlope	1,364 to 2,428
IC50	45,77 to 61,49
Goodness of Fit	
Degrees of Freedom	13
R square	0,9594
Absolute Sum of Squares	786,6
Sy.x	7,779
Constraints	
Bottom	Bottom = 0,0
Top	Top = 100,0
Number of points	
Analyzed	15

Inhibition Assay for A0A0H2XHJ5 with Phenelzine

Inhibition was determined by quantifying the enzymes product alanine by LC-MS. For this, protein A0A0H2XHJ5 (5 µM) was preincubated for 40 min at 37 °C with different concentrations of phenelzine (10 mM, 1 mM, 100 µM, 50 µM, 10 µM, 1 µM; from 100x stocks in ddH_2O) in buffer [50 mM 3-(morpholin-4-yl)propane-1-sulfonic acid (MOPS), pH = 7.4]. To the solutions, 2 µM protein A0A0H2XJC0, 10 mM L-Cys (500 mM stock in ddH_2O), 5 mM DTT (500 mM stock in ddH_2O) and 100 µM PLP (10 mM stock in ddH_2O) were added (final volume 100 µL) and the reactions were incubated at 37 °C for 30 min. All conditions were conducted in triplicates. A heat control (protein pre-incubation at 95 °C for 5 min) and samples with only one of each of the two proteins were performed as control experiments. Samples were further treated as described in Further Sample Preparation.
Graphpad Prism 5.03: log(inhibitor) vs. response -- Variable slope (four parameters) output table:

Y=Bottom + (Top-Bottom)/(1+10^((LogIC50-X)*HillSlope))

log(inhibitor) vs. response -- Variable slope (four parameters)	
Best-fit values	
Bottom	= 0,0
Top	= 100,0
LogIC50	1,167
HillSlope	0,5761
IC50	14,70
Span	= 100,0
Std. Error	
LogIC50	0,08771
HillSlope	0,06951
95% Confidence Intervals	
LogIC50	0,9815 to 1,353
HillSlope	0,4287 to 0,7234
IC50	9,582 to 22,56
Goodness of Fit	
Degrees of Freedom	16
R square	0,9284
Absolute Sum of Squares	1043
Sy.x	8,073
Constraints	
Bottom	Bottom = 0,0
Top	Top = 100,0
Number of points	
Analyzed	18

Further Sample Preparation

After incubation, reactions were stopped by adding 400 µL cold acetone (-80 °C) and the samples were stored for 2 h at -20 °C to complete protein precipitation. Proteins were then removed by centrifugation (21,000 x *g*, 4 °C, 20 min) and the supernatants were transferred into fresh 1.5 mL tubes for subsequent evaporation of solvents (centrifugal vacuum concentrator, *Eppendorf*). Dried samples were reconstituted in 50 µL H_2O/MeCN = 2/1 + 0.1% formic acid, sonicated for 5 min and clarified by centrifugation (17,000 x *g*, room temperature, 10 min). Prior to MS, samples were transferred into LC-MS-vials.

MS Measurement and Analysis

LC-MS/MS and LC-MS measurements were carried out on an Ultimate™ 3000 RSLC system (*Thermo Scientific*) coupled to an LTQ Orbitrap XL mass spectrometer (*Thermo Scientific*). Chromatographic separation was performed using a Accucore™ HILIC column (No. 17526-152130, 150x2.1 mm, 2.6 µm, *Thermo Scientific*) at 25 °C. Gradient elution was carried out with 0.1% formic acid (LC-MS grade, *Fisher Analytic*) and 5 mM ammonium acetate (Optima™ LC-MS grade, *Fisher Scientific*) in H_2O/MeCN = 50/50 (LC-MS grade, *Fisher Scientific*) (buffer A) and 0.1% formic acid and 5 mM ammonium acetate in H_2O/MeCN = 5/95 (buffer B). After 2 min pre-equilibration

with 5% buffer A, samples were injected and eluted with a linear gradient from 5% to 50% buffer A over 12 min and from 50% to 100% buffer A over 3 min followed by backflushing the column to 5% buffer A over 3 min and re-equilibration at 5% buffer A for 2 min at 400 µL/min flow rate. Mass spectrometric measurements were conducted in HESI positive or negative mode (H-ESI-II source, *Thermo Scientific*) with the following parameters:

Table SI 23: Mass spectrometry settings.

Educt Compound	Polarity	Capillary Voltage [kV]	Capillary Temperature [°C]	Capillary Voltage 2 [V]	Tube Lens [V]	Sheat Gas [L/h]	Aux Gas [L/h]	Quantification Method	Scan range full [m/z]
Serine	-	3.80	350	-2.00	-40.00	50	10	PRM	50-600
Threonine	-	3.70	350	-16.00	-30.00	60	15	PRM	50-650
Tryptophane	-	3.80	350	-6.00	-75.00	65	0	PRM	50-1000
Tyrosine	-	3.50	350	-1.00	-65.00	34	0	PRM	50-1000
Phenylalanine	-	3.50	350	-8.00	-40.00	34	0	PRM	50-1000
Methionine	-	3.80	350	-2.00	-30.00	35	30	PRM	50-1000
Glutamine	-	3.80	350	-26.00	-50.00	45	0	PRM	50-1000
Lysine	+	3.00	350	5.00	30.00	22	5	PRM	50-1000
Cysteine	+	3.50	350	6.00	25.00	40	0	SIM	50-500

Full scan measurements were accomplished in profile mode in the orbitrap at a resolution of 60.000, PRM or SIM methods at 30.000 in the orbitrap. Fragmentation was accomplished by collision-induced dissociation (CID) with parameters given in **Table SI 24**. Raw spectra were processed with *Thermo Scientific* XCalibur™ 1.2.

Table SI 24: Settings for MS/MS methods.

Educt Compound	Scan range PRM [m/z]	Parent Mass [m/z]	Collision Energy	Activation Time [ms]
Serine	50-500	87.01	35.00	30.00
Threonine	50-500	101.02	35.00	30.00
Tryptophane	55-300	202.05	35.00	30.00
Tyrosine	50-300	179.03	35.00	30.00
Phenylalanine	50-300	163.04	35.00	30.00
Methionine	50-200	147.01	35.00	30.00
Glutamine	50-200	144.03	35.00	30.00
Lysine	50-250	103.12	35.00	30.00

Compound Screen

5 mL overnight cultures of wild type strains *S. aureus* USA300 (B-medium), *E. coli* K12 (LB-medium) and *P. aeruginosa* PAO1 (LB-medium) were diluted 1:10000 in their corresponding media, distributed in a 96-well plate (Nunclon™ Delta Surface, *Thermo Scientific*) and compounds (**Table SI 4**) were first applied at 500 µM (from 50 mM stock, solvent see **Table SI 4**) to a final volume of

100 µL. Hit compounds were then fine-screened at 250, 100, 50 and 25 µM. All experiments were carried out in triplicates and solvent-only and sterile samples were added as controls.

References

[1] Y. Perez-Riverol, A. Csordas, J. Bai, M. Bernal-Llinares, S. Hewapathirana, D. J. Kundu, A. Inuganti, J. Griss, G. Mayer, M. Eisenacher et al., *Nucleic Acids Res.* **2019**, *47*, D442-D450.
[2] A. Tramonti, C. Nardella, M. L. Di Salvo, A. Barile, F. D'Alessio, V. de Crécy-Lagard, R. Contestabile, *EcoSal Plus* **2021**, *9*, eESP-0004-2021.
[3] A. Tramonti, C. Nardella, M. L. Di Salvo, S. Pascarella, R. Contestabile, *FEBS J.* **2018**, *285*, 3925–3944.
[4] a) K. D. Schnackerz, J. H. Ehrlich, W. Giesemann, T. A. Reed, *Biochemistry* **1979**, *18*, 3557–3563; b) J. Jin, U. Hanefeld, *Chem. Commun.* **2011**, *47*, 2502–2510.
[5] P. D. Fey, J. L. Endres, V. K. Yajjala, T. J. Widhelm, R. J. Boissy, J. L. Bose, K. W. Bayles, *mBio* **2013**, *4*, e00537-12.
[6] a) M. Blahut, C. E. Wise, M. R. Bruno, G. Dong, T. M. Makris, P. A. Frantom, J. A. Dunkle, F. W. Outten, *J. Biol. Chem.* **2019**, *294*, 12444–12458; b) H. Mihara, N. Esaki, *Appl. Microbiol. Biotechnol.* **2002**, *60*, 12–23.
[7] A. G. Albrecht, F. Peuckert, H. Landmann, M. Miethke, A. Seubert, M. A. Marahiel, *FEBS Lett.* **2011**, *585*, 465–470.
[8] M. P. Wilson, B. Plecko, P. B. Mills, P. T. Clayton, *J. Inherited Metab. Dis.* **2019**, *42*, 629–646.
[9] H. B. Lin, R. Falchetto, P. J. Mosca, J. Shabanowitz, D. F. Hunt, J. L. Hamlin, *J. Biol. Chem.* **1996**, *271*, 2548–2556.
[10] S. A. Shah, B. W. Shen, A. T. Brünger, *Structure* **1997**, *5*, 1067–1075.
[11] A. Amadasi, M. Bertoldi, R. Contestabile, S. Bettati, B. Cellini, M. L. Di Salvo, C. Borri-Voltattorni, F. Bossa, A. Mozzarelli, *Curr. Med. Chem.* **2007**, *14*, 1291–1324.
[12] a) M. Ciustea, S. Mootien, A. E. Rosato, O. Perez, P. Cirillo, K. R. Yeung, M. Ledizet, M. H. Cynamon, P. A. Aristoff, R. A. Koski et al., *Biochem. Pharmacol.* **2012**, *83*, 368–377; b) Y. Lee, S. Mootien, C. Shoen, M. Destefano, P. Cirillo, O. A. Asojo, K. R. Yeung, M. Ledizet, M. H. Cynamon, P. A. Aristoff et al., *Biochem. Pharmacol.* **2013**, *86*, 222–230.
[13] M. A. Azam, U. Jayaram, *J. Enzyme Inhib. Med. Chem.* **2016**, *31*, 517–526.
[14] R. Schwarcz, R. Pellicciari, *J. Pharmacol. Exp. Ther.* **2002**, *303*, 1–10.
[15] A. K. Gandhi, J. V. Desai, M. S. Ghatge, M. L. Di Salvo, S. Di Biase, R. Danso-Danquah, F. N. Musayev, R. Contestabile, V. Schirch, M. K. Safo, *PloS one* **2012**, *7*, e40954.
[16] A. Marabotti, P. Cozzini, A. Mozzarelli, *Biochim. Biophys. Acta, Protein Struct. Mol. Enzymol.* **2000**, *1476*, 287–299.
[17] A. K. Bence, D. R. Worthen, V. R. Adams, P. A. Crooks, *Anti-cancer drugs* **2002**, *13*, 313–320.
[18] D. Nagel, L. Wallcave, B. Toth, R. Kupper, *Cancer Res.* **1977**, *37*, 3458–3460.
[19] S. Zlitni, L. F. Ferruccio, E. D. Brown, *Nat. Chem. Biol.* **2013**, *9*, 796–804.
[20] E. M. Kaiser, W. R. Thomas, T. E. Synos, J. R. McClure, T. S. Mansour, J. R. Garlich, J. E. Chastain, *J. Organomet. Chem.* **1981**, *213*, 405–417.
[21] A. Hoegl, M. B. Nodwell, V. C. Kirsch, N. C. Bach, M. Pfanzelt, M. Stahl, S. Schneider, S. A. Sieber, *Nat. Chem.* **2018**, *10*, 1234–1245.
[22] E. C. Davison, I. T. Forbes, A. B. Holmes, J. A. Warner, *Tetrahedron* **1996**, *52*, 11601–11624.
[23] S. Caddick, V. Delisser, V. Doyle, S. Khan, A. Avent, S. Vile, *Tetrahedron* **1999**, *55*, 2737–2754.
[24] B. M. Trost, H. C. Shen, D. B. Horne, F. D. Toste, B. G. Steinmetz, C. Koradin, *Chemistry* **2005**, *11*, 2577–2590.
[25] U. Groth, N. Richter, A. Kalogerakis, *Eur. J. Org. Chem.* **2003**, *2003*, 4634–4639.
[26] J. Egger, C. Weckerle, B. Cutting, O. Schwardt, S. Rabbani, K. Lemme, B. Ernst, *J. Am. Chem. Soc.* **2013**, *135*, 9820–9828.
[27] Y. Saito, K. Matsumoto, S. S. Bag, S. Ogasawara, K. Fujimoto, K. Hanawa, I. Saito, *Tetrahedron* **2008**, *64*, 3578–3588.
[28] Y.-C. Kim, K. A. Jacobson, *Synthesis* **2000**, *2000*, 119–122.
[29] F. Himo, T. Lovell, R. Hilgraf, V. V. Rostovtsev, L. Noodleman, K. B. Sharpless, V. V. Fokin, *J. Am. Chem. Soc.* **2005**, *127*, 210–216.
[30] P. Prusevich, J. H. Kalin, S. A. Ming, M. Basso, J. Givens, X. Li, J. Hu, M. S. Taylor, A. M. Cieniewicz, P.-Y. Hsiao et al., *ACS Chem. Biol.* **2014**, *9*, 1284–1293.
[31] A. Fux, M. Pfanzelt, V. C. Kirsch, A. Hoegl, S. A. Sieber, *Cell chem. biol.* **2019**, *26*, 1461-1468.e7.
[32] T. Katoh, O. Ohmori, K. Iwasaki, M. Inoue, *Tetrahedron* **2002**, *58*, 1289–1299.
[33] B. Witulski, U. Bergsträßer, M. Gößmann, *Tetrahedron* **2000**, *56*, 4747–4752.
[34] E. Alicea-Matías, J. A. Soderquist, *Org. Lett.* **2017**, *19*, 336–339.
[35] C. Sibbersen, J. Palmfeldt, J. Hansen, N. Gregersen, K. A. Jørgensen, M. Johannsen, *Chem. Commun.* **2013**, *49*, 4012–4014.
[36] M. Saha, M. S. Hossain, S. Bandyopadhyay, *Angew. Chem. Int. Ed.* **2021**, *60*, 5220–5224.
[37] J. M. Thiede, S. L. Kordus, B. J. Turman, J. A. Buonomo, C. C. Aldrich, Y. Minato, A. D. Baughn, *Sci. Rep.* **2016**, *6*, 38083.
[38] T. J. Dale, A. C. Sather, J. Rebek, *Tetrahedron Lett.* **2009**, *50*, 6173–6175.
[39] M. Liebeke, K. Dörries, D. Zühlke, J. Bernhardt, S. Fuchs, J. Pané-Farré, S. Engelmann, U. Völker, R. Bode, T. Dandekar et al., *Mol. Biosyst.* **2011**, *7*, 1241–1253.
[40] Arie Geerlof, "M9 mineral medium", can be found under https://www.helmholtz-muenchen.de/fileadmin/PEPF/Protocols/M9-medium_150510.pdf, **2010**.
[41] A. E. LaBauve, M. J. Wargo, *Curr. Protoc. Microbiol.* **2012**, *Chapter 6*, Unit 6E.1.

[42] Thermo Fisher Scientific, "Gateway Cloning Protocols", can be found under https://www.thermofisher.com/de/de/home/life-science/cloning/gateway-cloning/protocols.html.

[43] a) A. Mozzarelli, S. Bettati, *Chem. Rec.* **2006**, *6*, 275–287; b) W. R. Griswold, M. D. Toney, *J. Am. Chem. Soc.* **2011**, *133*, 14823–14830.

[44] G. T. Höfler, E. Fernández-Fueyo, M. Pesic, S. H. Younes, E.-G. Choi, Y. H. Kim, V. B. Urlacher, I. W. C. E. Arends, F. Hollmann, *ChemBioChem* **2018**, *19*, 2344–2347.

[45] M. H. Kunzmann, N. C. Bach, B. Bauer, S. A. Sieber, *Chem. Sci.* **2014**, *5*, 1158–1167.

[46] T. Baba, T. Ara, M. Hasegawa, Y. Takai, Y. Okumura, M. Baba, K. A. Datsenko, M. Tomita, B. L. Wanner, H. Mori, *Mol. Syst. Biol.* **2006**, *2*, 2006.0008.

[47] S. Tyanova, T. Temu, J. Cox, *Nat. Protoc.* **2016**, *11*, 2301–2319.

[48] J. Cox, N. Neuhauser, A. Michalski, R. A. Scheltema, J. V. Olsen, M. Mann, *J. Proteome Res.* **2011**, *10*, 1794–1805.

Appendix

Abbreviations

**ADP**	Adenosine diphosphate
**Asc**	Ascorbate
**ATP**	Adenosine triphosphate
**B Medium**	Broth Medium
**BTTAA**	2-(4-((bis((1-(tert-butyl)-1H-1,2,3-triazol-4-yl)methyl)amino)methyl)-1H-1,2,3-triazol-1-yl)acetic acid
**Calcd**	Calculated
**CAM**	Ceric ammonium molybdate
**CDM**	Chemically defined medium
**DCM**	Dichloromethane
**DIPA**	Diisopropylamine
**DIPEA**	Diisopropylethylamine
**DMF**	Dimethyl formamide
**DMSO**	Dimethylsulfoxide
**DNA**	Deoxyribonucleic acid
**DTT**	Dithiothreitol
**EDTA**	Ethylenediaminetetraacetic acid
**EMSA**	Electrophoretic mobility shift assay
**Et**	Ethyl
**ESI**	Electrospray ionization
**FDR**	False discovery rate
**HC**	Heat control
**HEPES**	(4-(2-hydroxyethyl)-1-piperazineethanesulfonic acid)
**HESI**	Heated electrospray ionization
**HILIC**	Hydrophilic interaction chromatography
**HPLC**	High-performance liquid chromatography
**HRMS**	High-resolution mass spectrometry
**IBCF**	Isobutyl chloroformate
**IPMS**	Intact protein mass spectrometry
**IPTG**	Isopropyl β-D-1-thiogalactopyranoside
**LB**	Lysogeny broth
**LC**	Liquid chromatography
**LDH**	Lactate dehydrogenase
***m*-CPBA**	Metachloro perbenzoic acid

**Me**	Methyl
**MeCN**	Acetonitrile
**MIC**	Minimum inhibitory concentration
**MOM**	Methoxymethyl
**MS**	Mass spectrometry
**Ms**	Mesylate
**MW**	Molecular Weight
**NADH**	Nicotinamide adenine dinucleotide
***n*-BuLi**	*n*-Butyllithium
**NMR**	Nuclear magnetic resonance
**OD**	Optical density
**o/n**	Overnight
**PBS**	Phosphate-buffered saline
**PCR**	Polymerase chain reaction
**Piv**	Pivalate
**PMA**	Phosphomolybdic acid
**PRM**	Parallel reaction monitoring
**satd.**	Saturated
**SIM**	Single ion monitoring
**TAMRA**	Carboxytetramethylrhodamine
**TCEP**	Tris(2-carboxyethyl)phosphine
**TEAB**	Triethylamonium bicarbonat
**TEV**	Tobacco etch virus
**TFA**	Trifluoro acetic acid
**THF**	Tetrahydrofuran
**TLC**	Thin-layer chromatography
**TMS**	Trimethylsilyl
**TRIS**	Tris(hydroxymethyl)aminomethane
**UV**	Ultraviolet
**Vis**	Visible
**wt**	Wild type

NMR Spectra

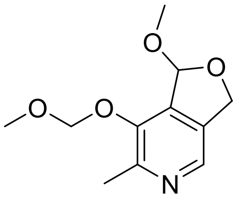

2

$C_{11}H_{15}NO_4$
M = 225.24 g/mol

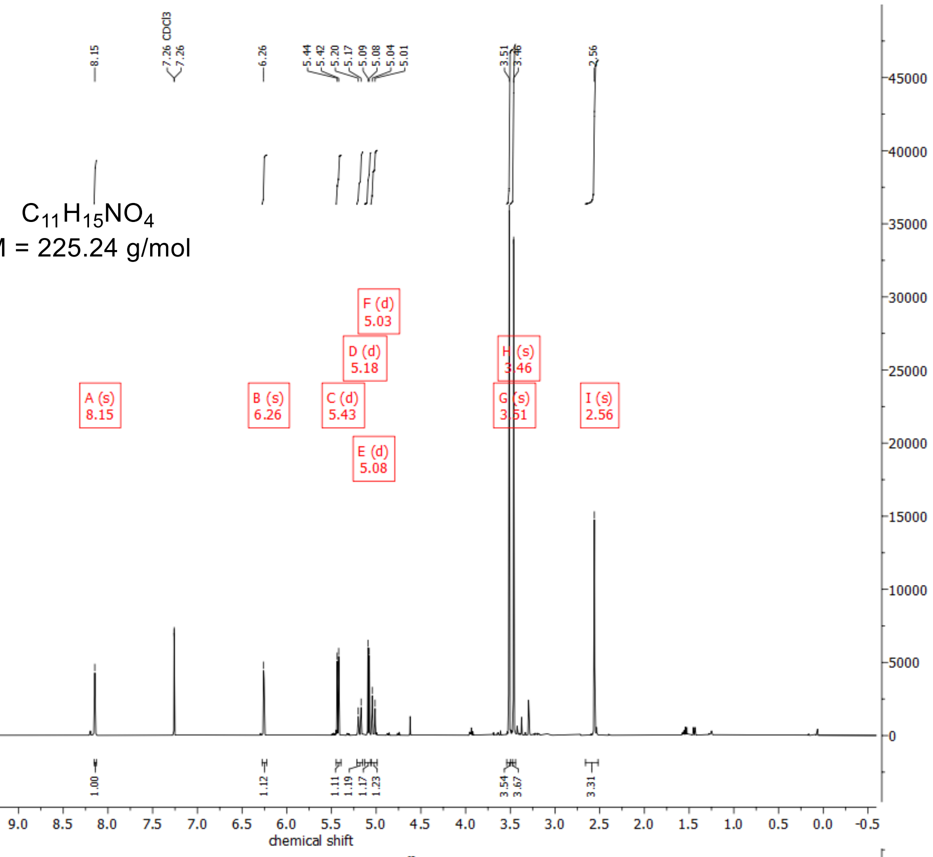

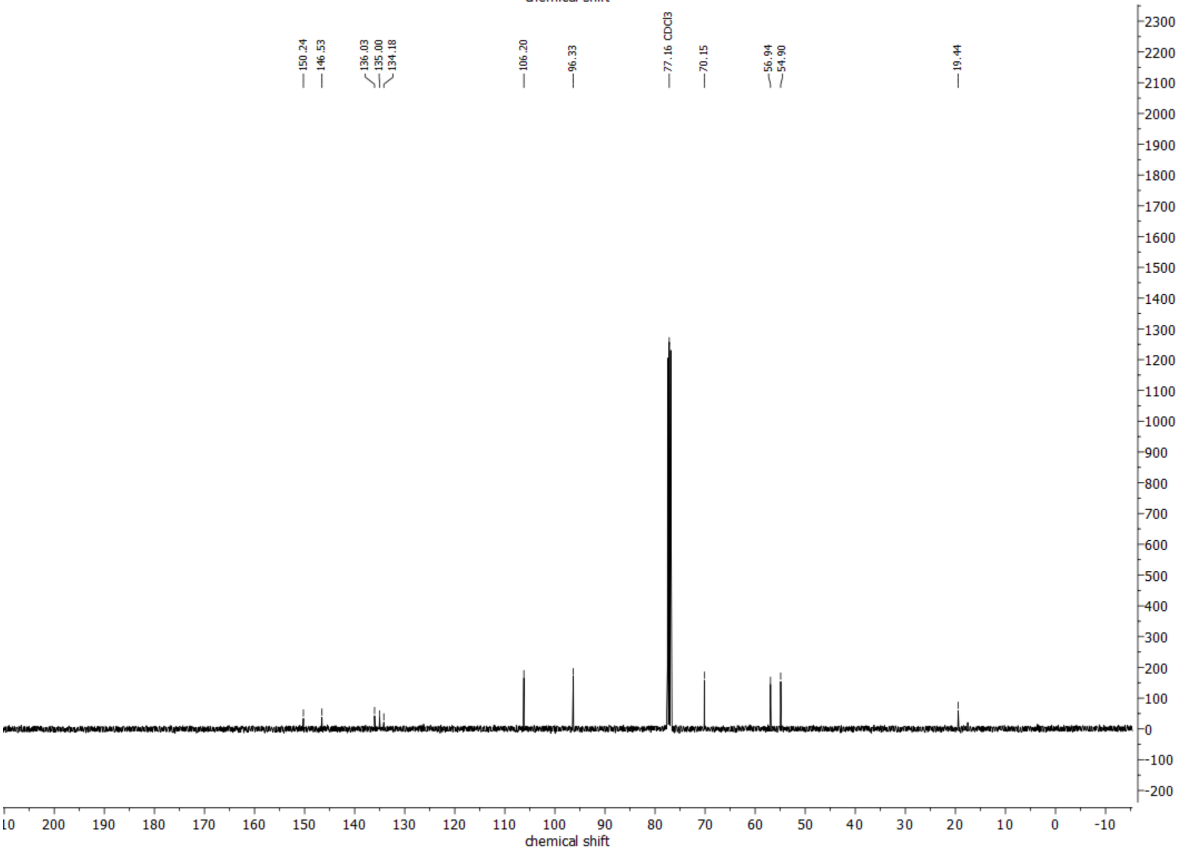

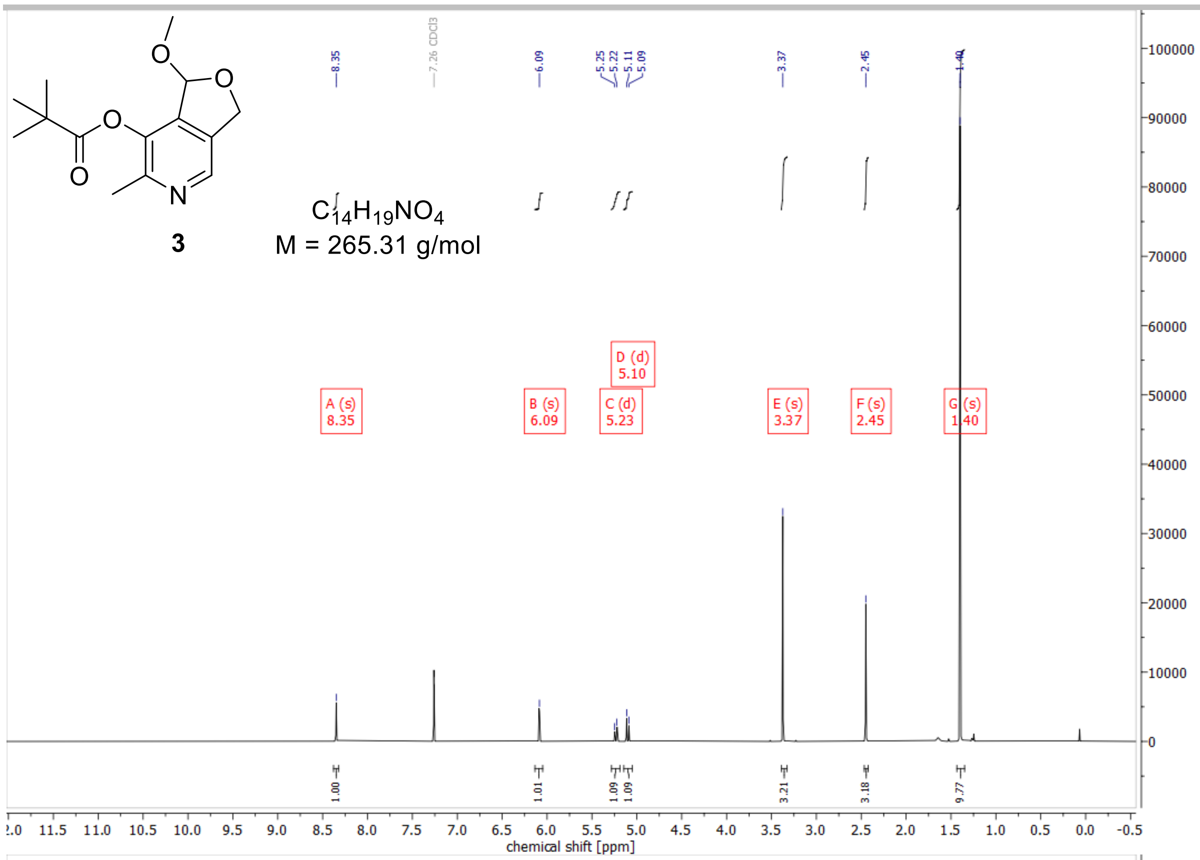
3
C14H19NO4
M = 265.31 g/mol
8.35
7.26 CDCl3
6.09
5.25
5.22
5.11
5.09
3.37
2.45
1.40
A (s) 8.35
B (s) 6.09
C (d) 5.23
D (d) 5.10
E (s) 3.37
F (s) 2.45
G (s) 1.40
1.00
1.01
1.09
1.09
3.21
3.18
9.77
chemical shift [ppm]

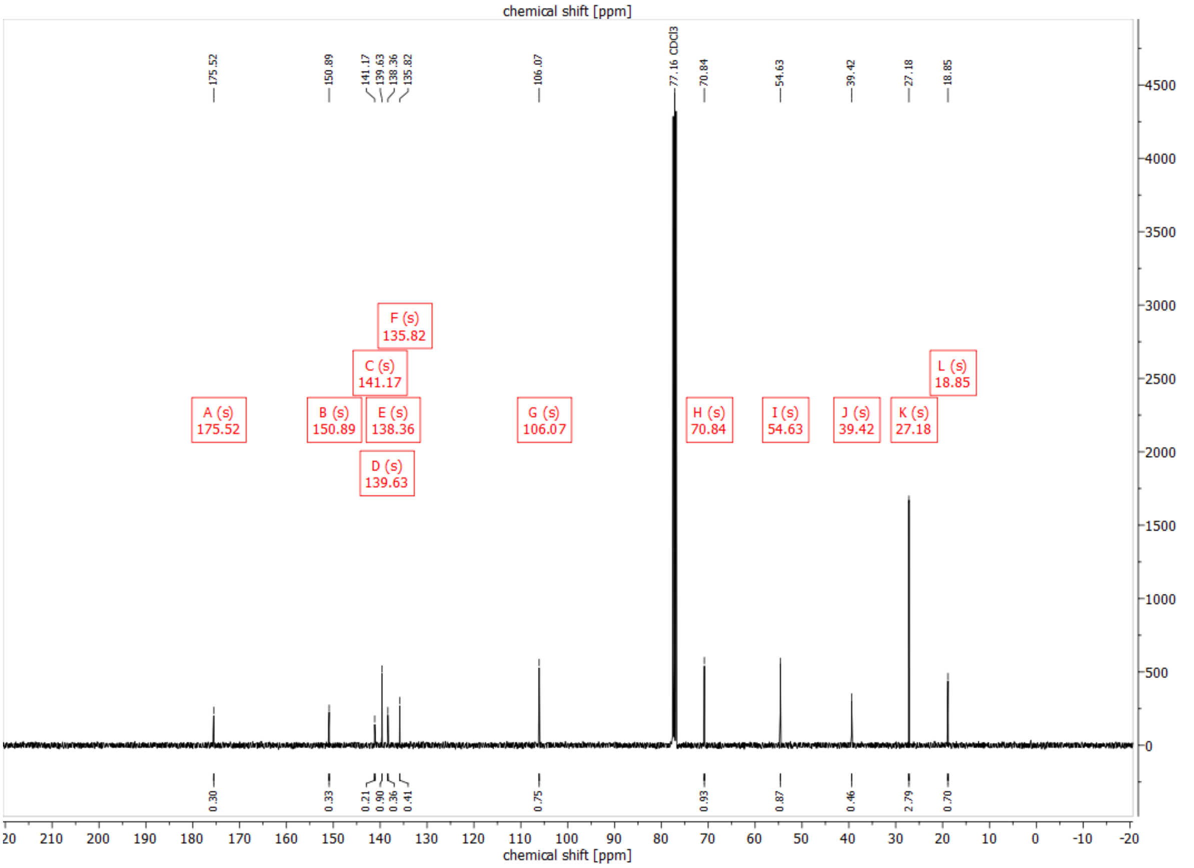
175.52
150.89
141.17
139.63
138.36
135.82
106.07
77.16 CDCl3
70.84
54.63
39.42
27.18
18.85
A (s) 175.52
B (s) 150.89
C (s) 141.17
D (s) 139.63
E (s) 138.36
F (s) 135.82
G (s) 106.07
H (s) 70.84
I (s) 54.63
J (s) 39.42
K (s) 27.18
L (s) 18.85
chemical shift [ppm]

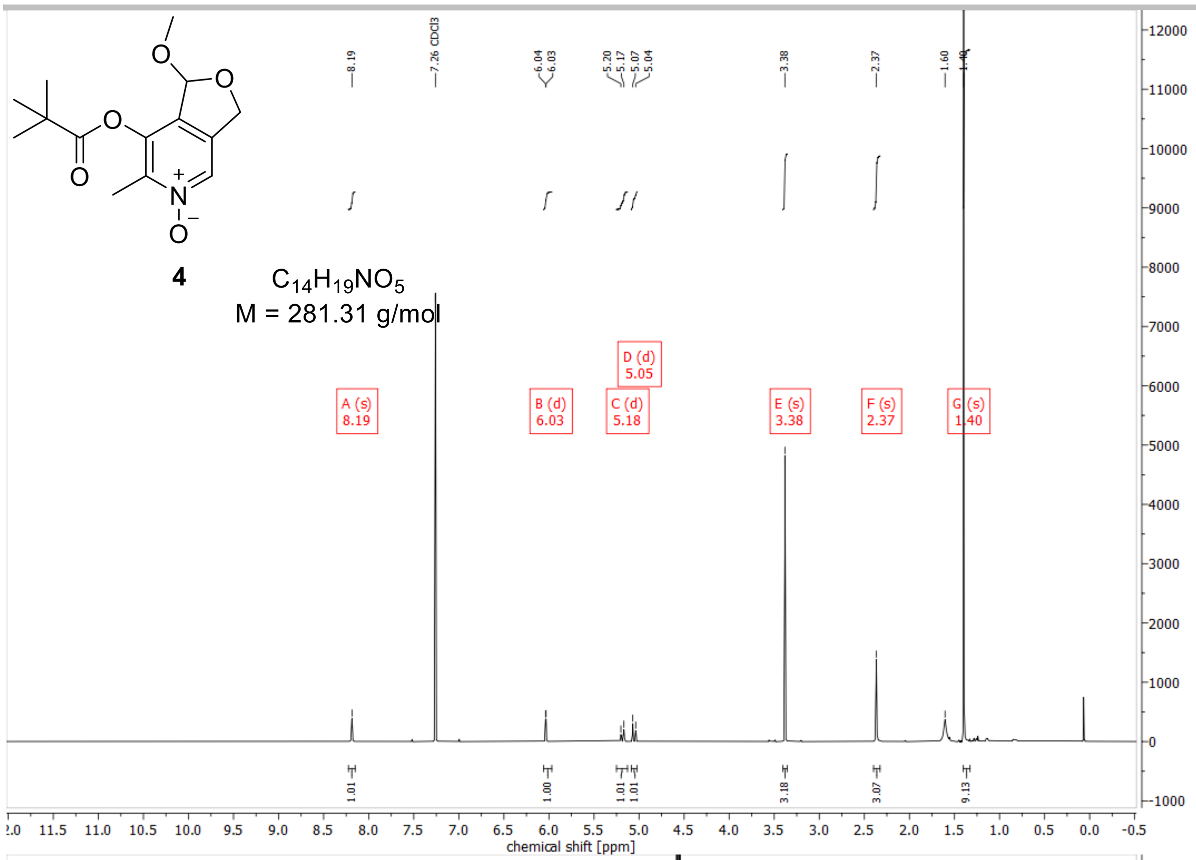
4
$C_{14}H_{19}NO_5$
M = 281.31 g/mol
A (s) 8.19
B (d) 6.03
C (d) 5.18
D (d) 5.05
E (s) 3.38
F (s) 2.37
G (s) 1.40
chemical shift [ppm]

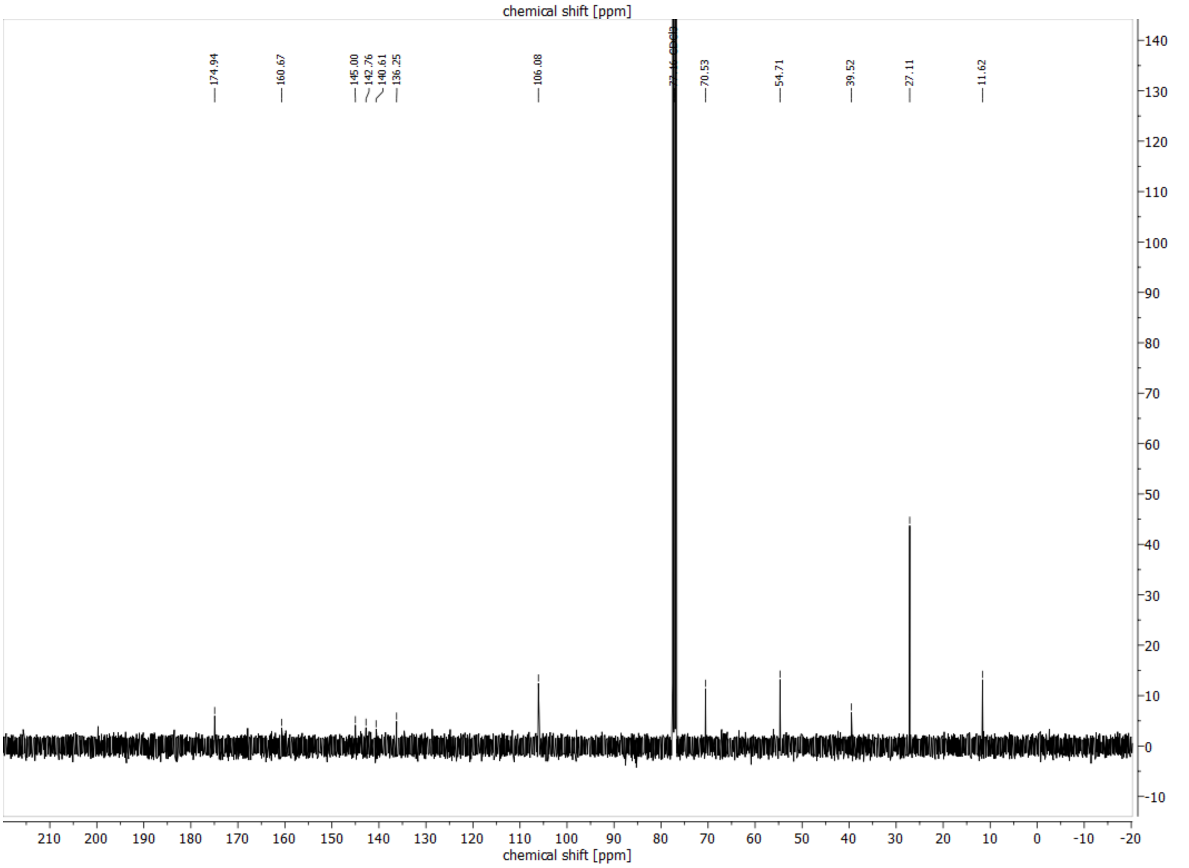
174.94
160.67
145.00
142.76
140.61
136.25
106.08
70.53
54.71
39.52
27.11
11.62
chemical shift [ppm]

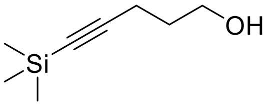

6 $C_8H_{16}OSi$
M = 156.30 g/mol

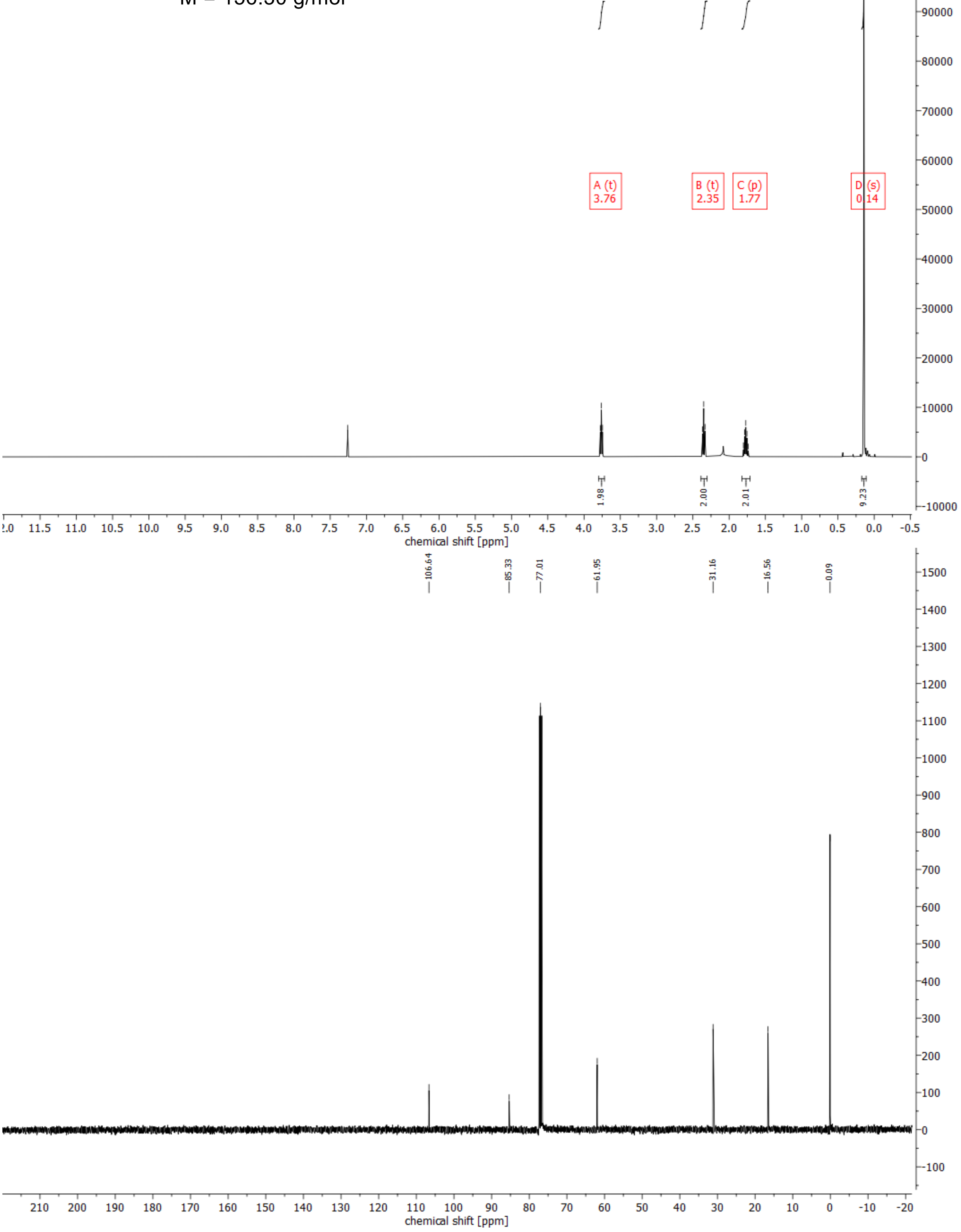

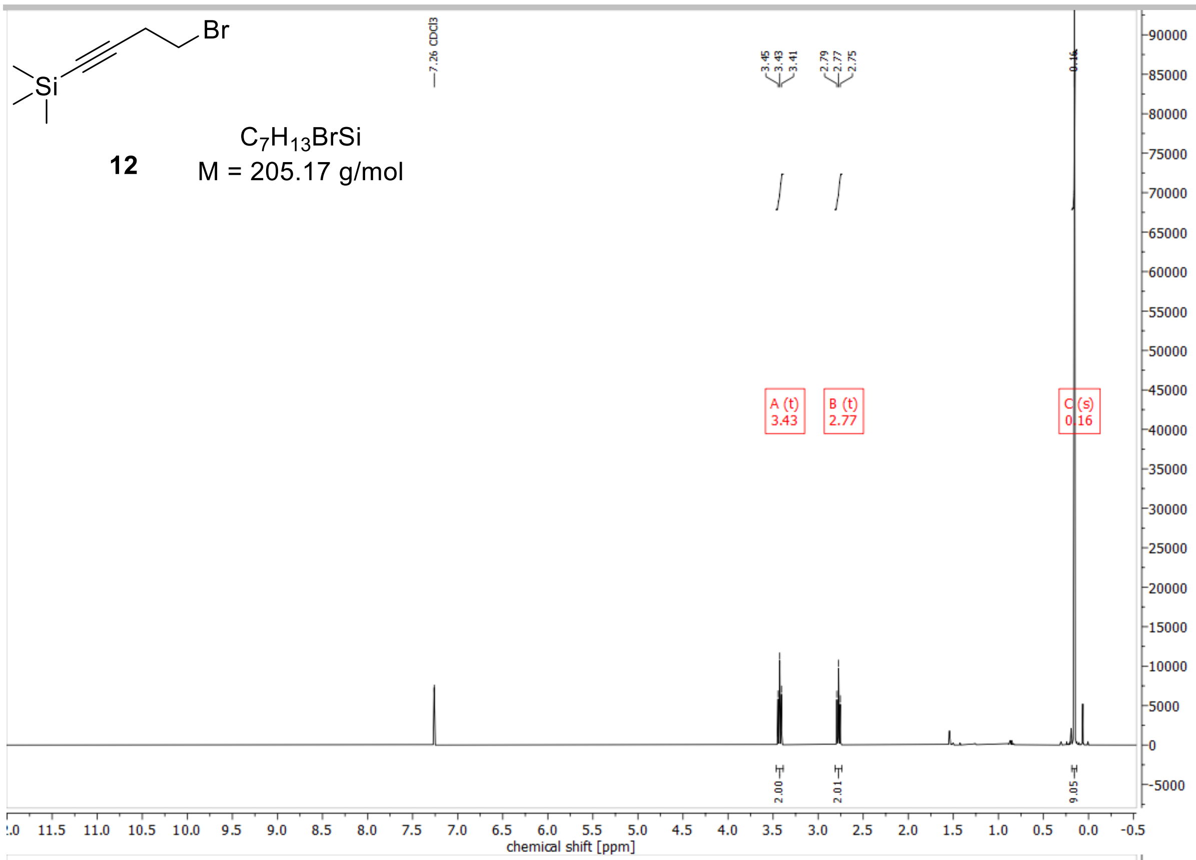
Br
Si
12
$C_7H_{13}BrSi$
M = 205.17 g/mol
7.26 CDCl3
3.45
3.43
3.41
2.79
2.77
2.75
0.16
A (t)
3.43
B (t)
2.77
C (s)
0.16
2.00
2.01
9.05
chemical shift [ppm]

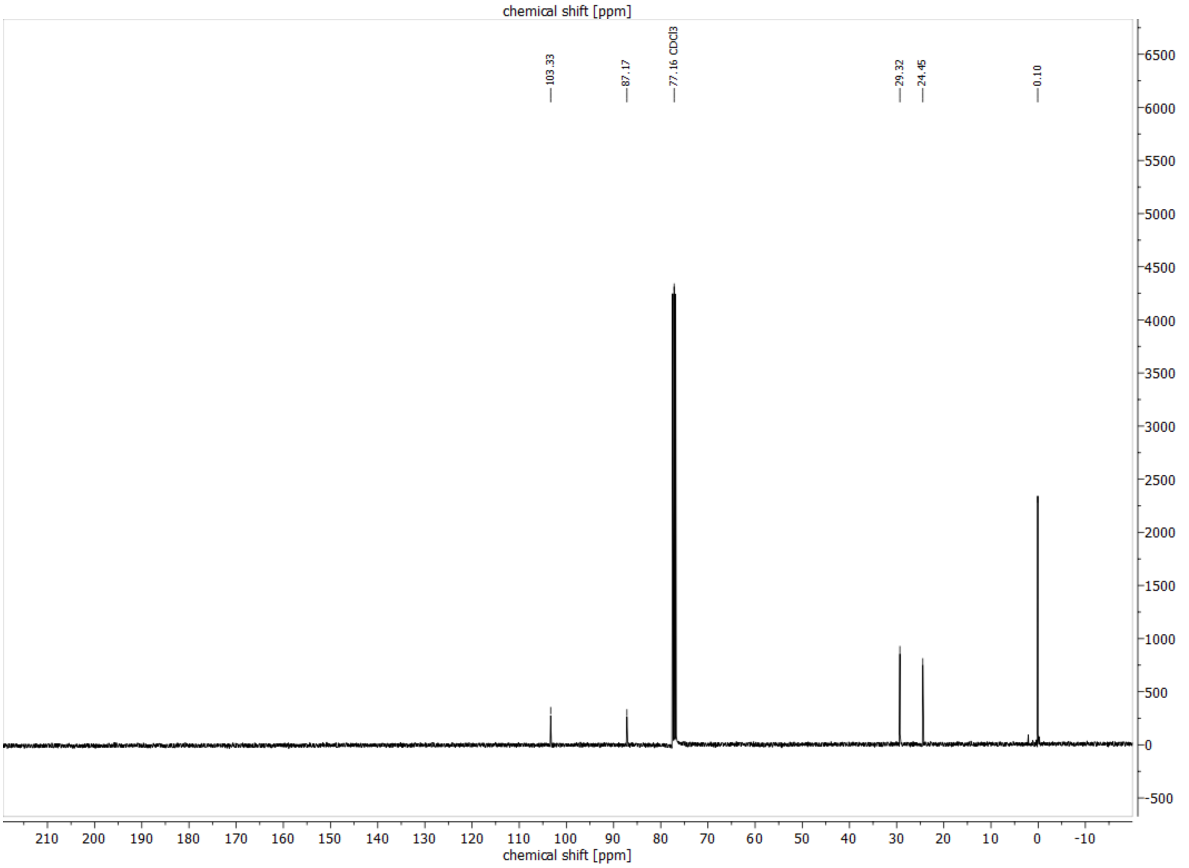
103.33
87.17
77.16 CDCl3
29.32
24.45
0.10
chemical shift [ppm]

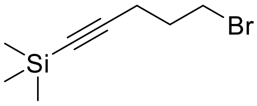

13

$C_8H_{15}BrSi$
M = 219.20 g/mol

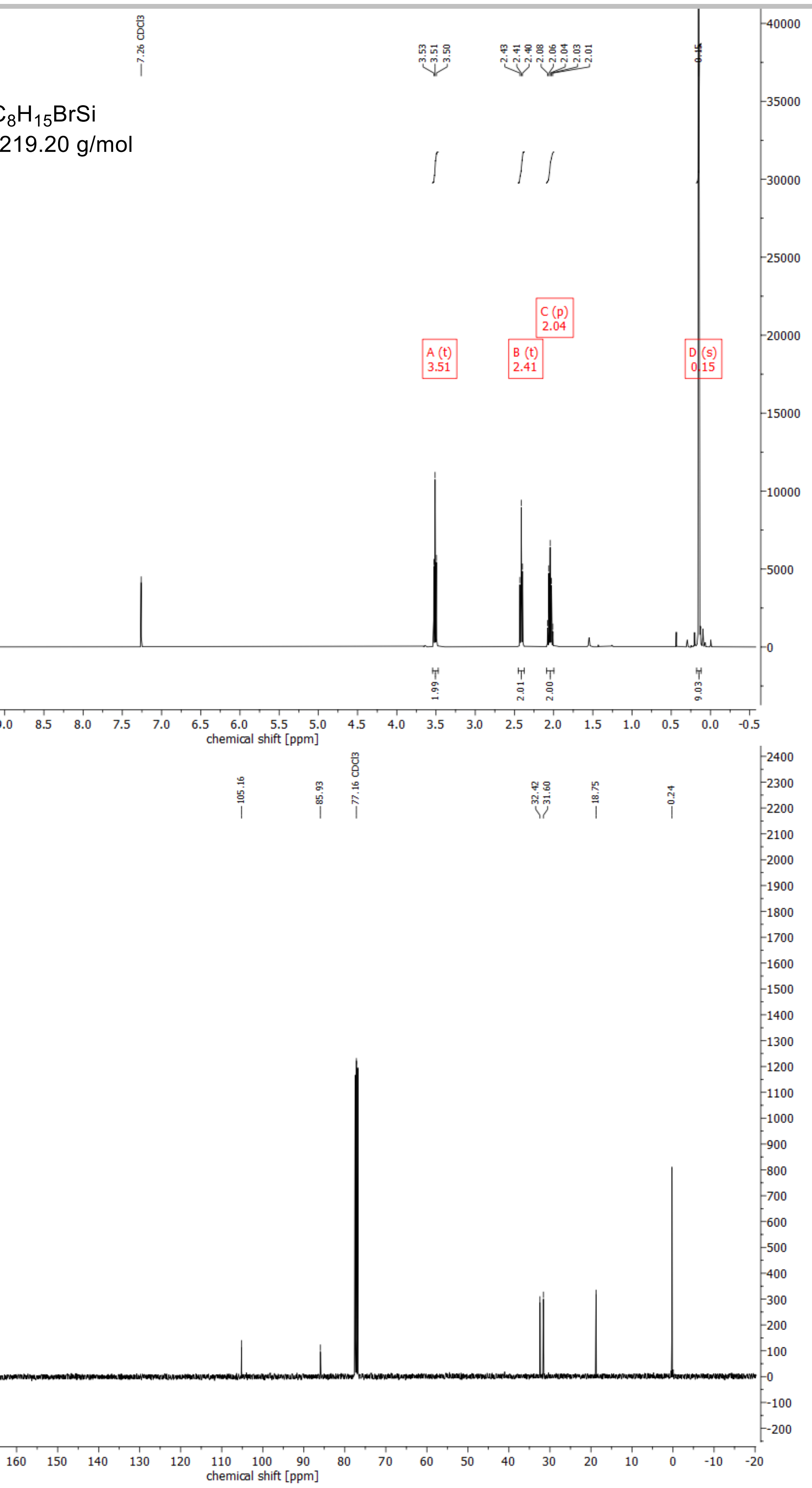

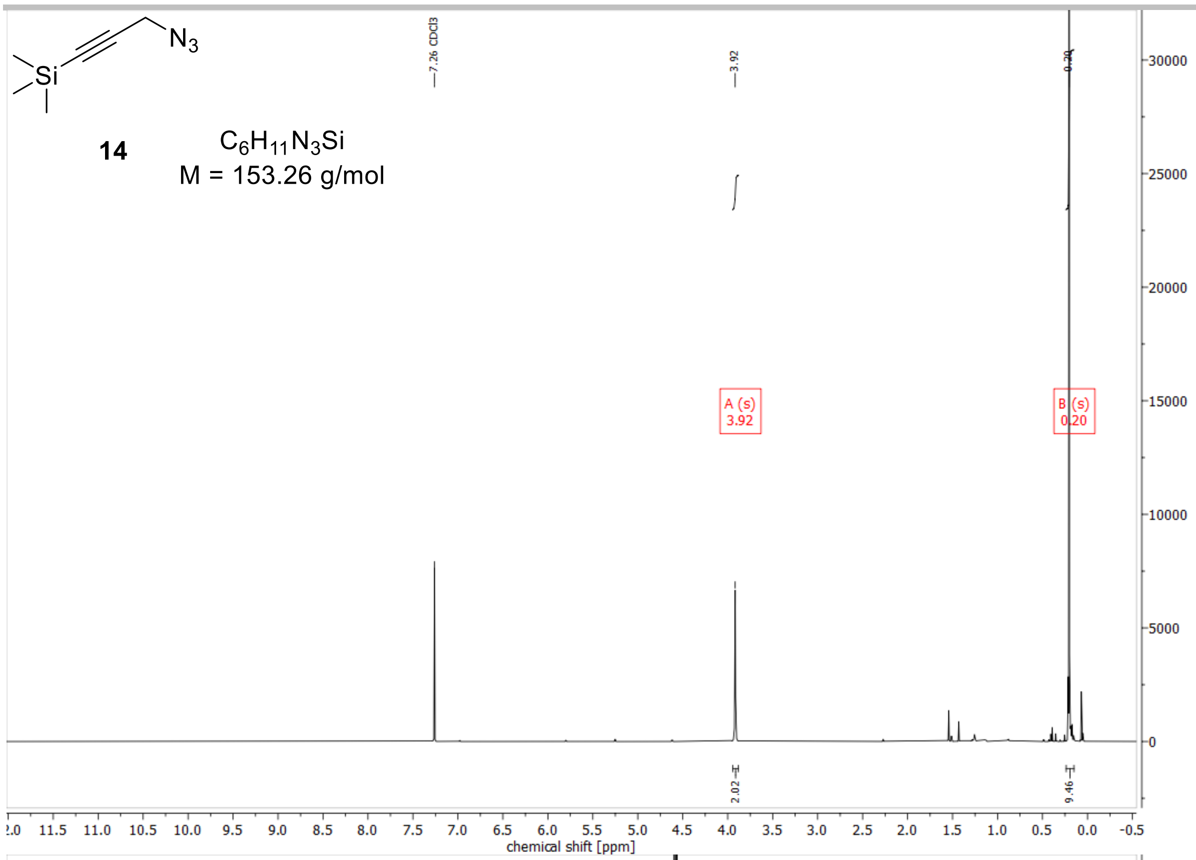
Si
N3
14
C6H11N3Si
M = 153.26 g/mol
7.26 CDCl3
3.92
0.20
A (s)
3.92
B (s)
0.20
2.02
9.46
chemical shift [ppm]

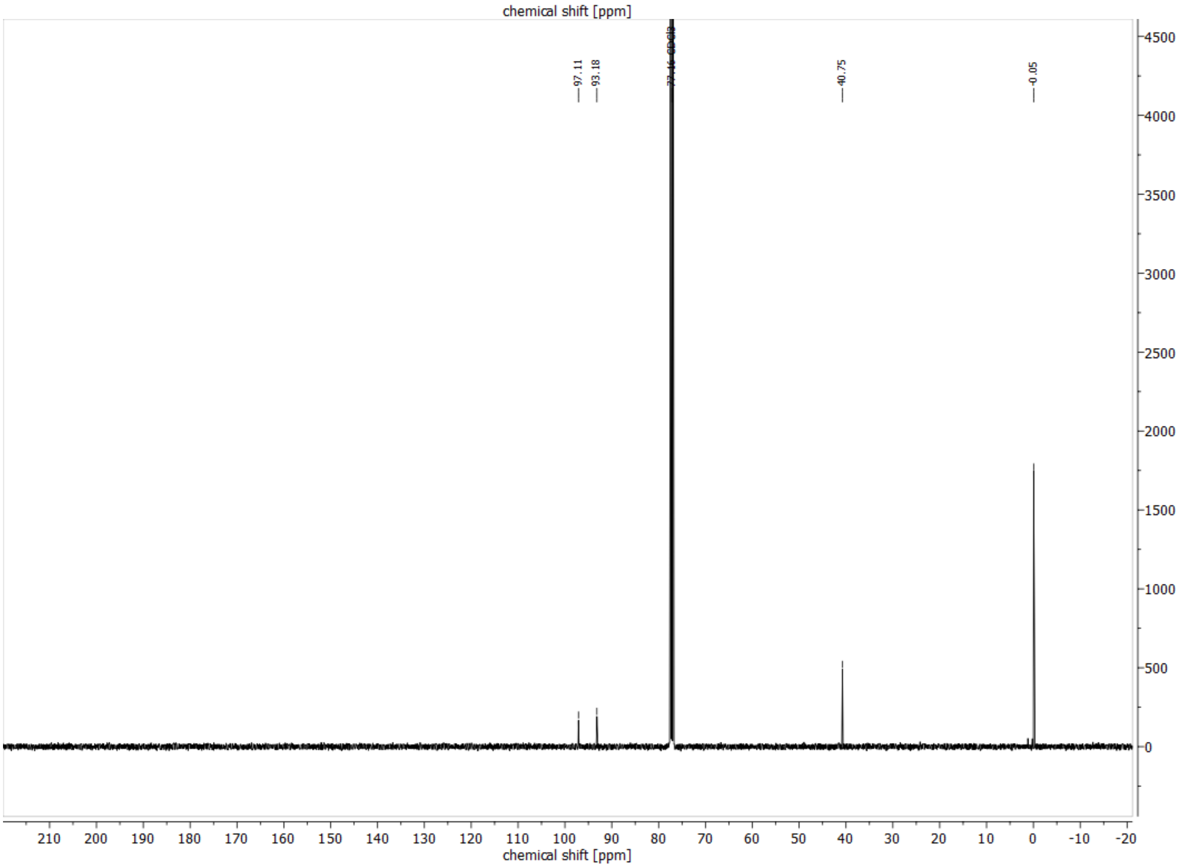
97.11
93.18
77.16 CDCl3
40.75
-0.05
chemical shift [ppm]

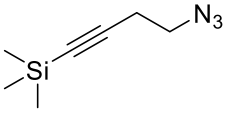

15

$C_7H_{13}N_3Si$
M = 167.29 g/mol

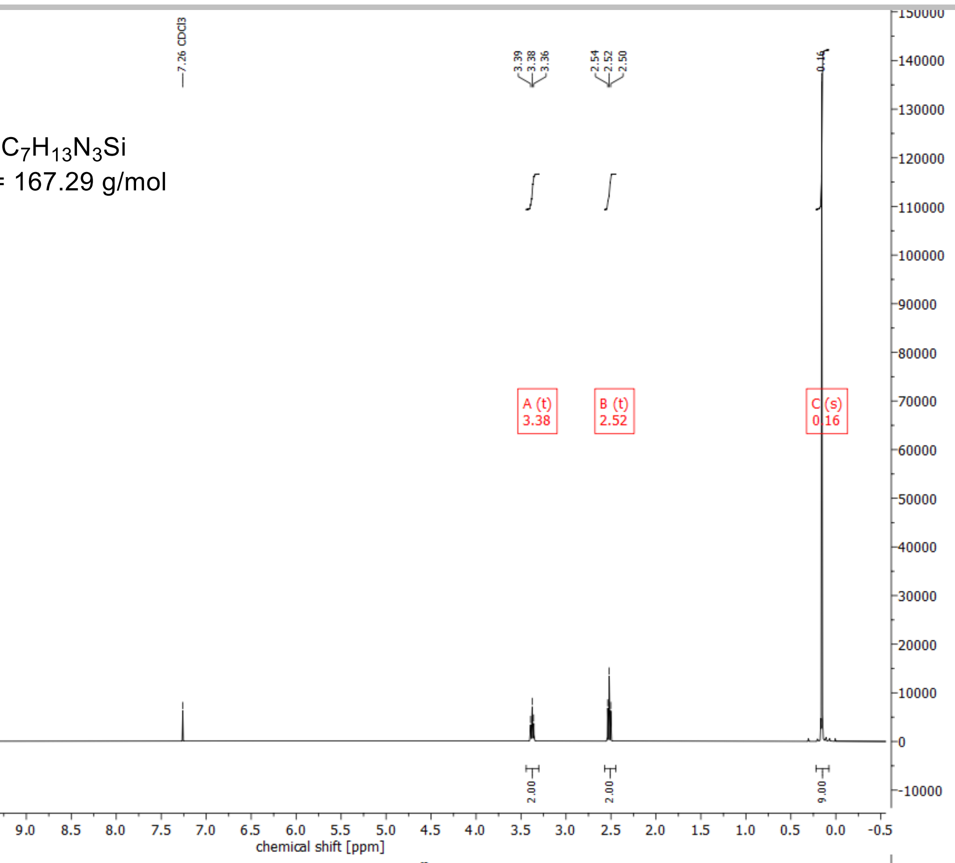

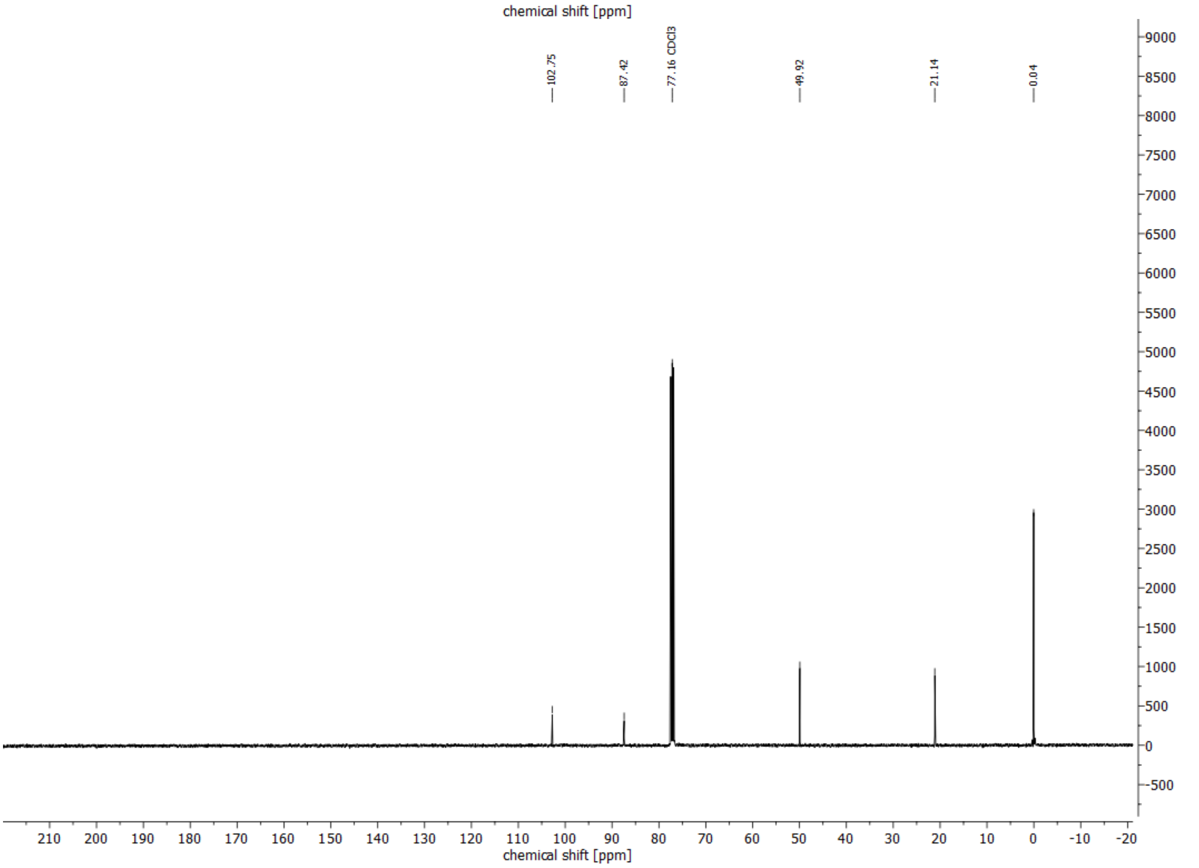

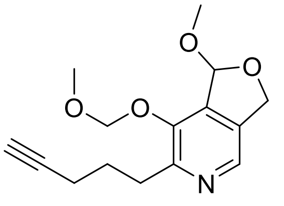

19 $C_{15}H_{19}NO_4$
M = 277.32 g/mol

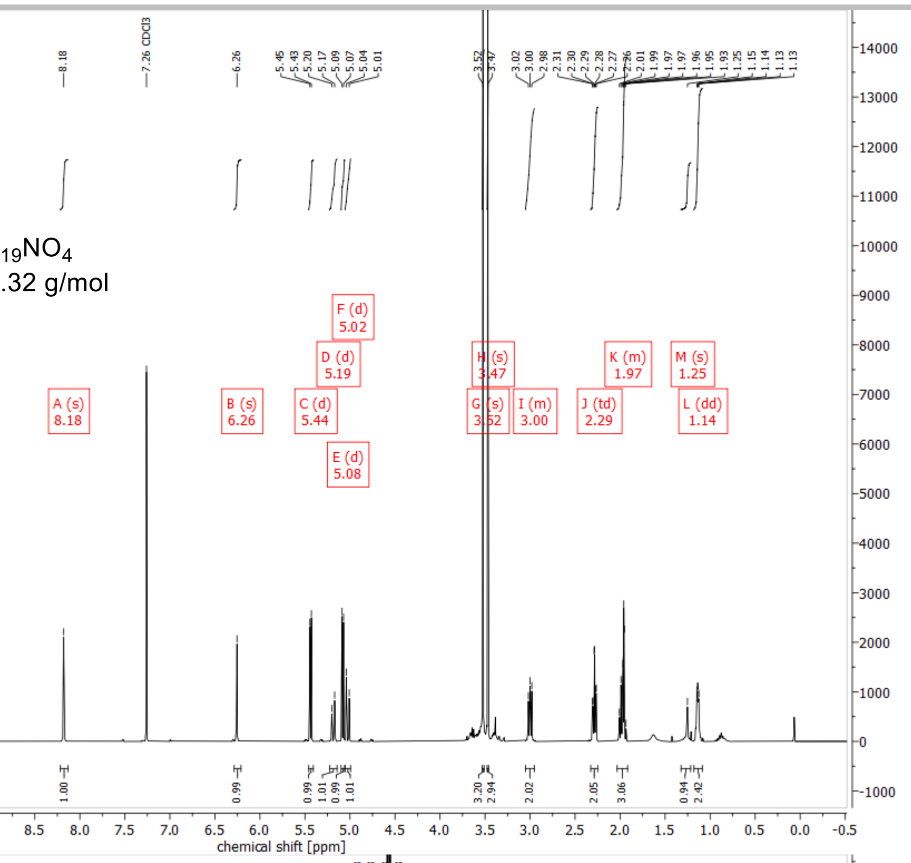

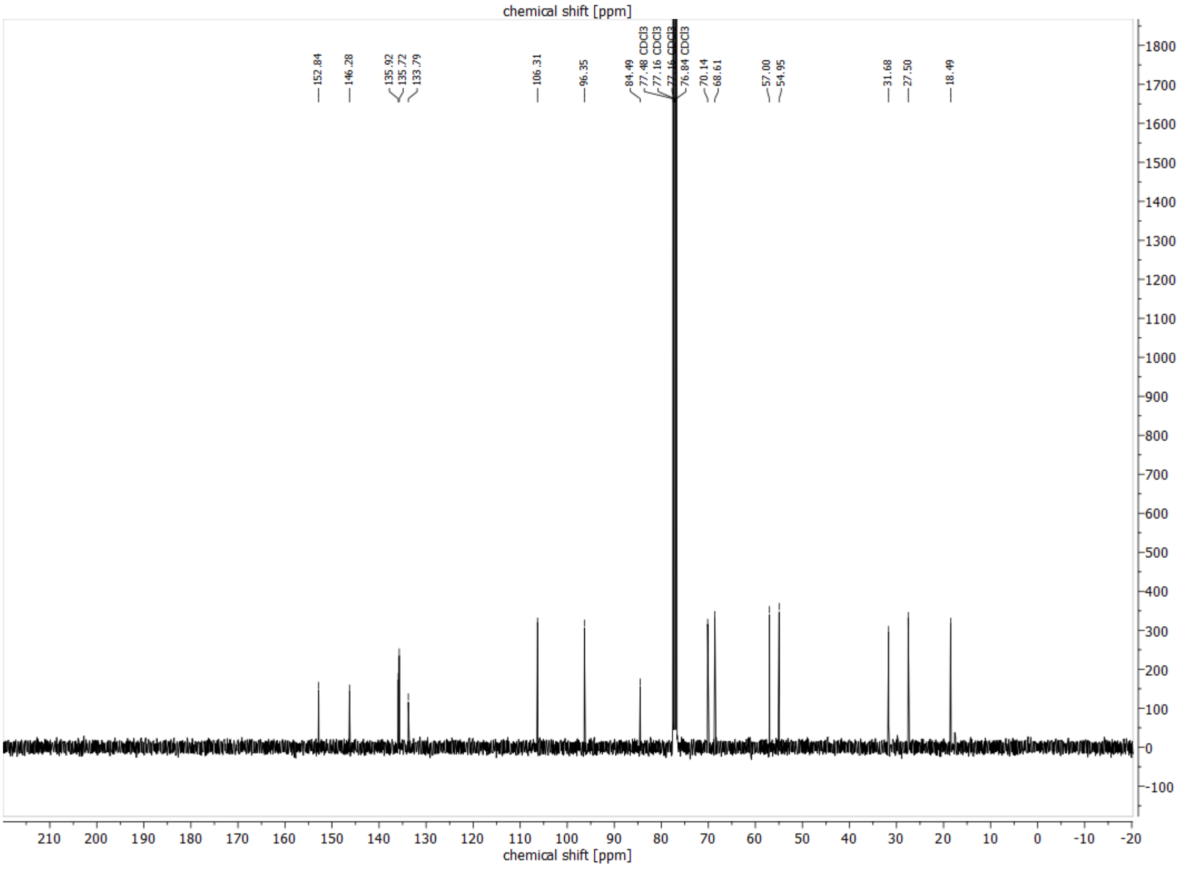

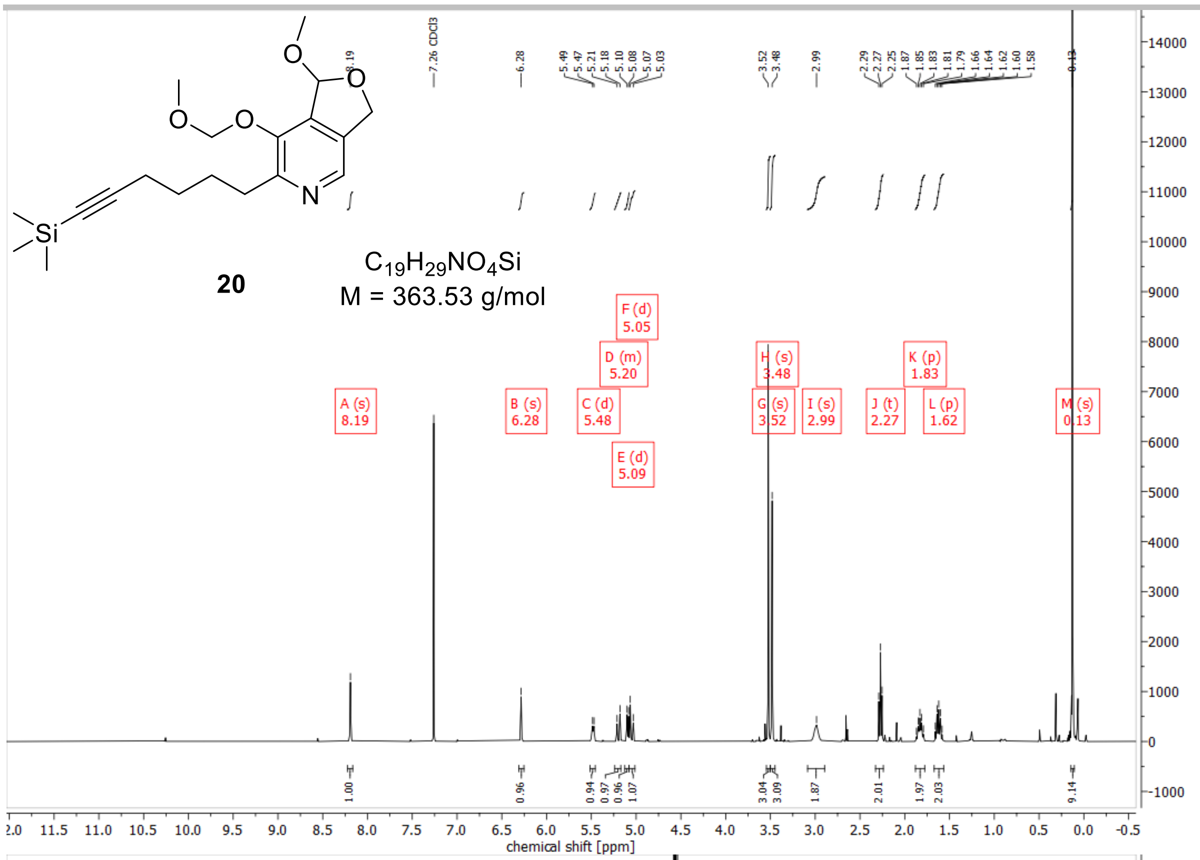
20
$C_{19}H_{29}NO_4Si$
M = 363.53 g/mol
A (s) 8.19
B (s) 6.28
C (d) 5.48
D (m) 5.20
E (d) 5.09
F (d) 5.05
G (s) 3.52
H (s) 3.48
I (s) 2.99
J (t) 2.27
K (p) 1.83
L (p) 1.62
M (s) 0.13
chemical shift [ppm]

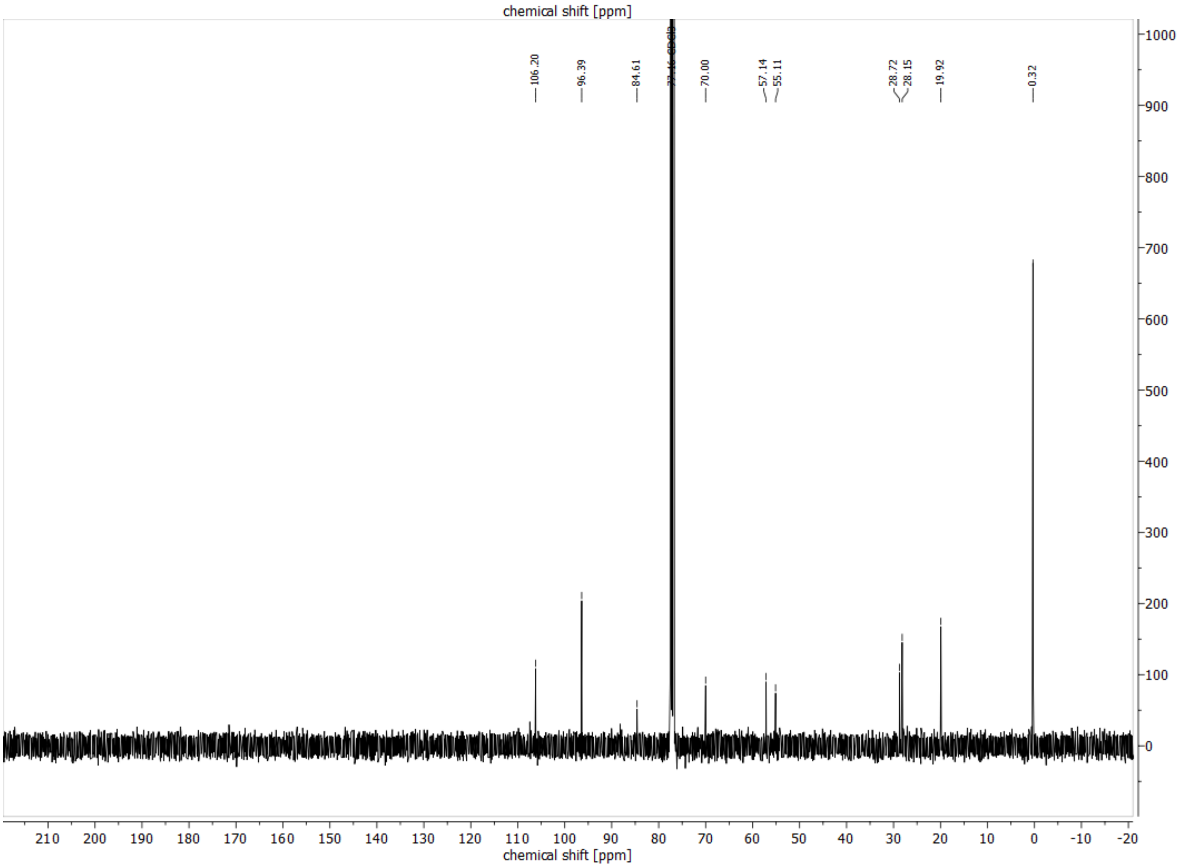
106.20
96.39
84.61
70.00
57.14
55.11
28.72
28.15
19.92
0.32
chemical shift [ppm]

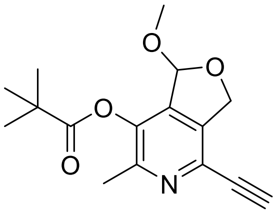

21 $C_{16}H_{19}NO_4$
M = 289.33 g/mol

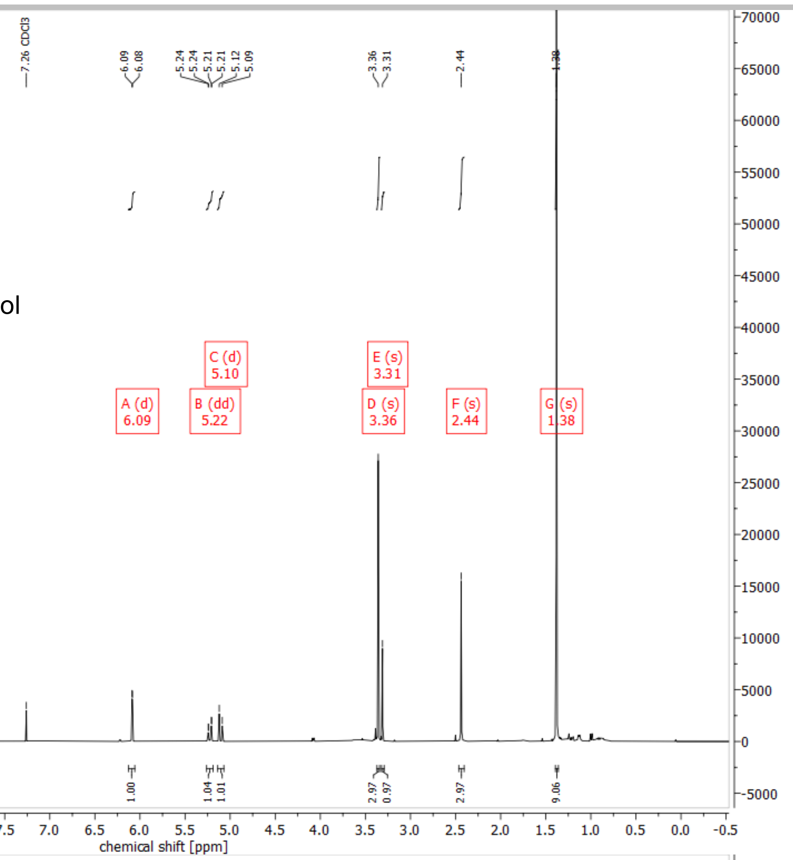

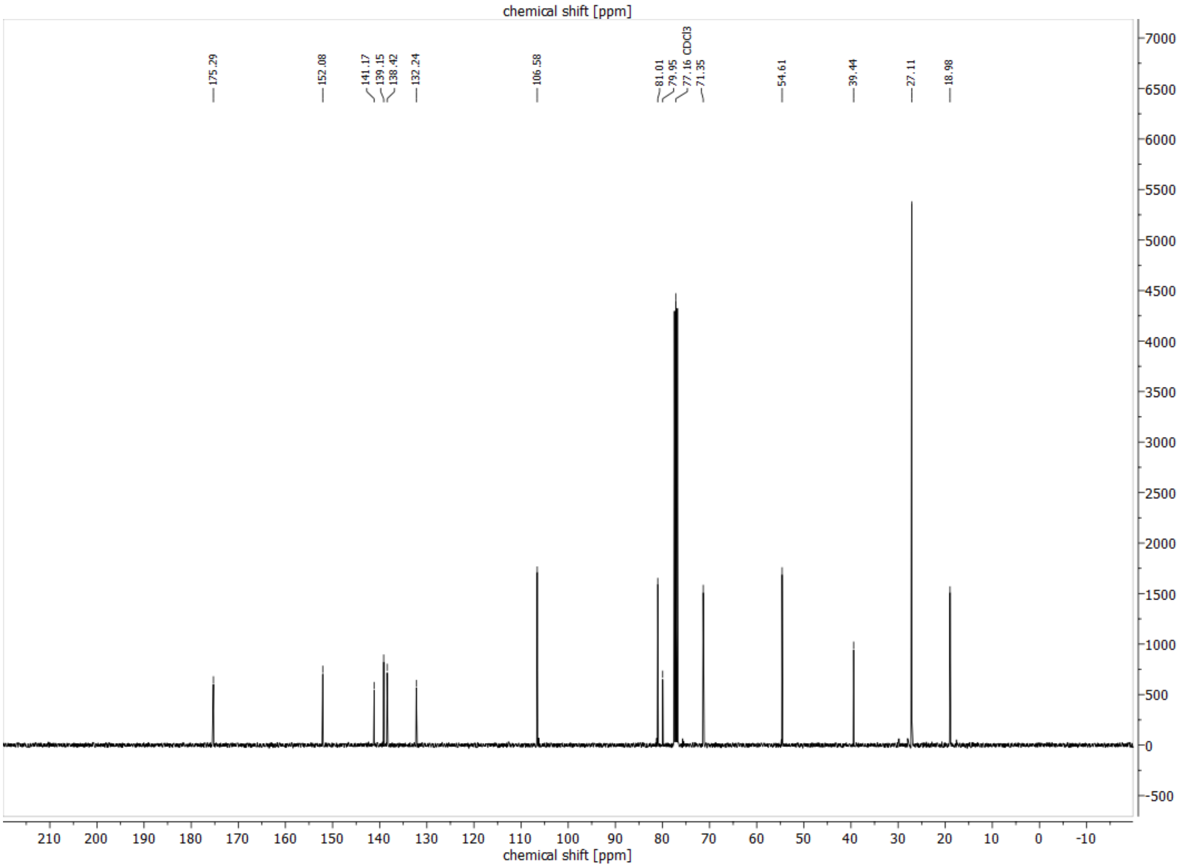

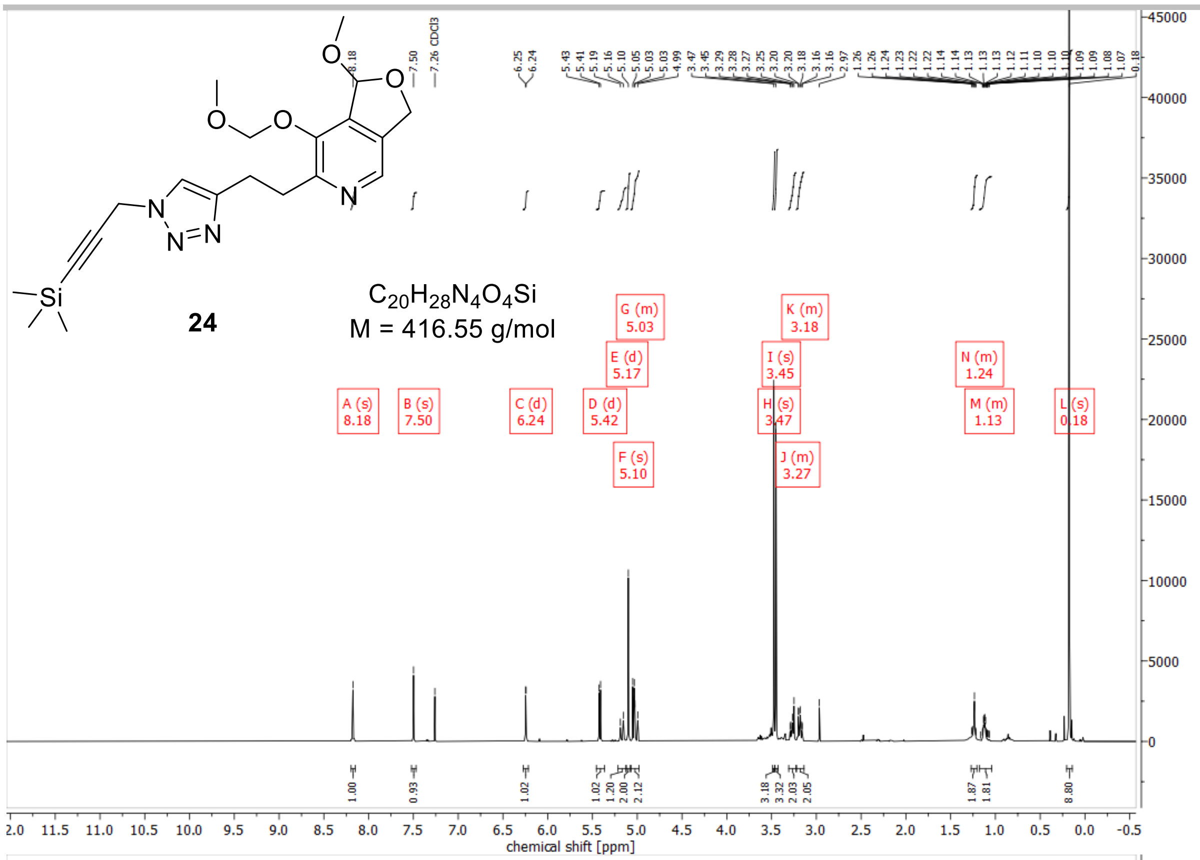
24
$C_{20}H_{28}N_4O_4Si$
M = 416.55 g/mol
A (s) 8.18
B (s) 7.50
C (d) 6.24
D (d) 5.42
E (d) 5.17
F (s) 5.10
G (m) 5.03
H (s) 3.47
I (s) 3.45
J (m) 3.27
K (m) 3.18
M (m) 1.13
N (m) 1.24
L (s) 0.18
chemical shift [ppm]

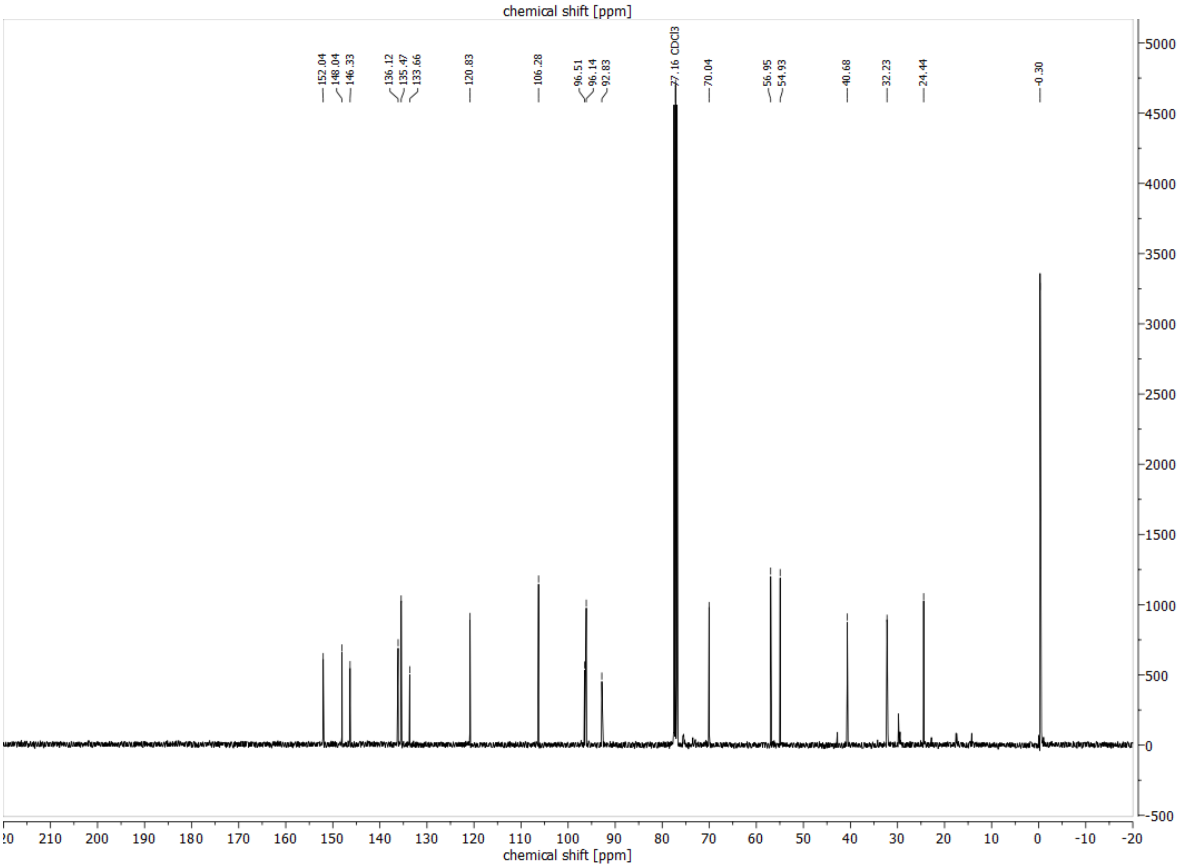
152.04
148.04
146.33
136.12
135.47
133.66
120.83
106.28
96.51
96.14
92.83
77.16 CDCl3
70.04
56.95
54.93
40.68
32.23
24.44
-0.30
chemical shift [ppm]

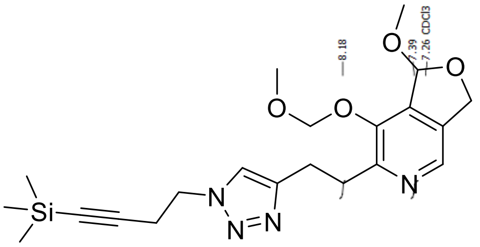

25

$C_{21}H_{30}N_4O_4Si$
M = 430.58 g/mol

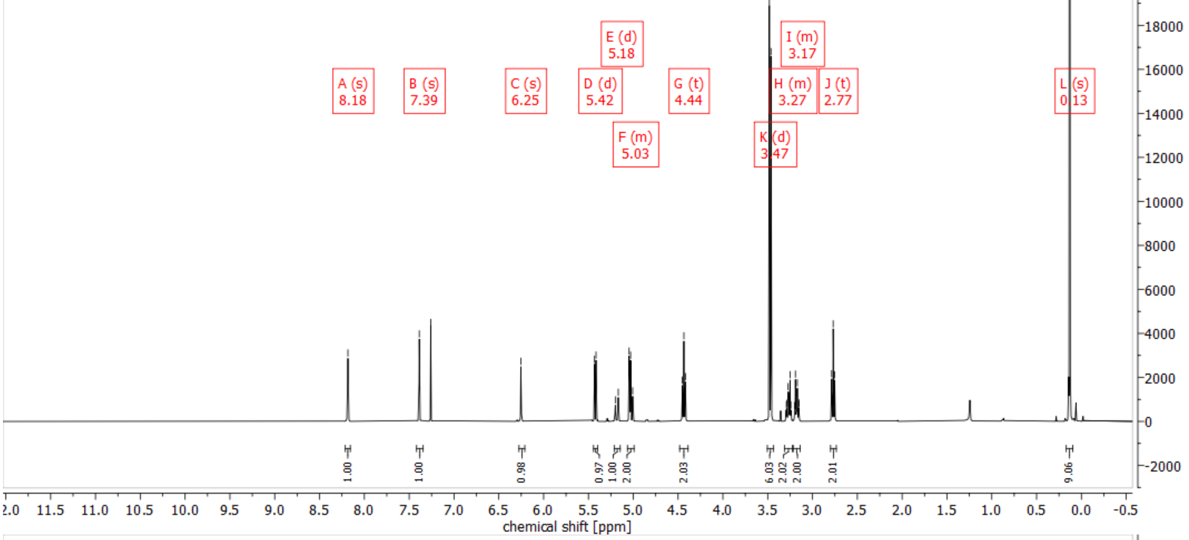

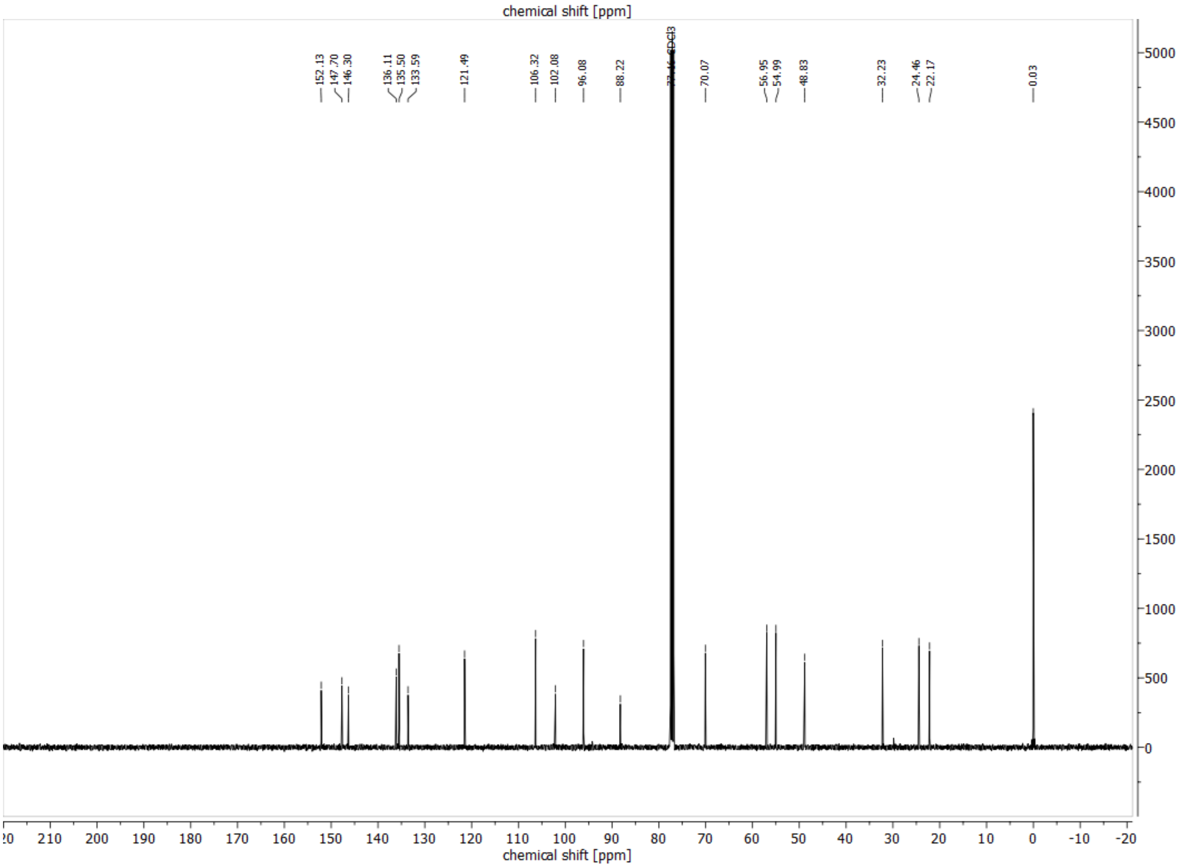

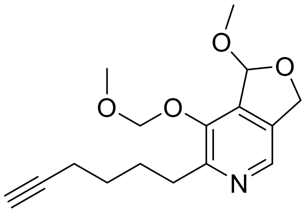

27

$C_{16}H_{21}NO_4$
M = 291.35 g/mol

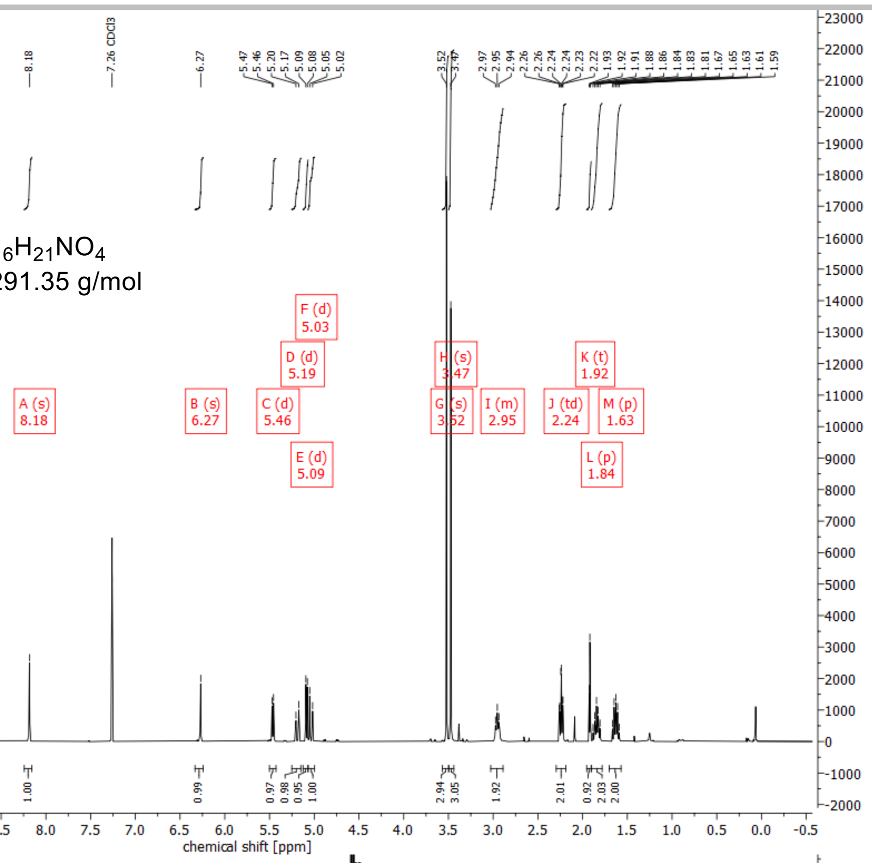

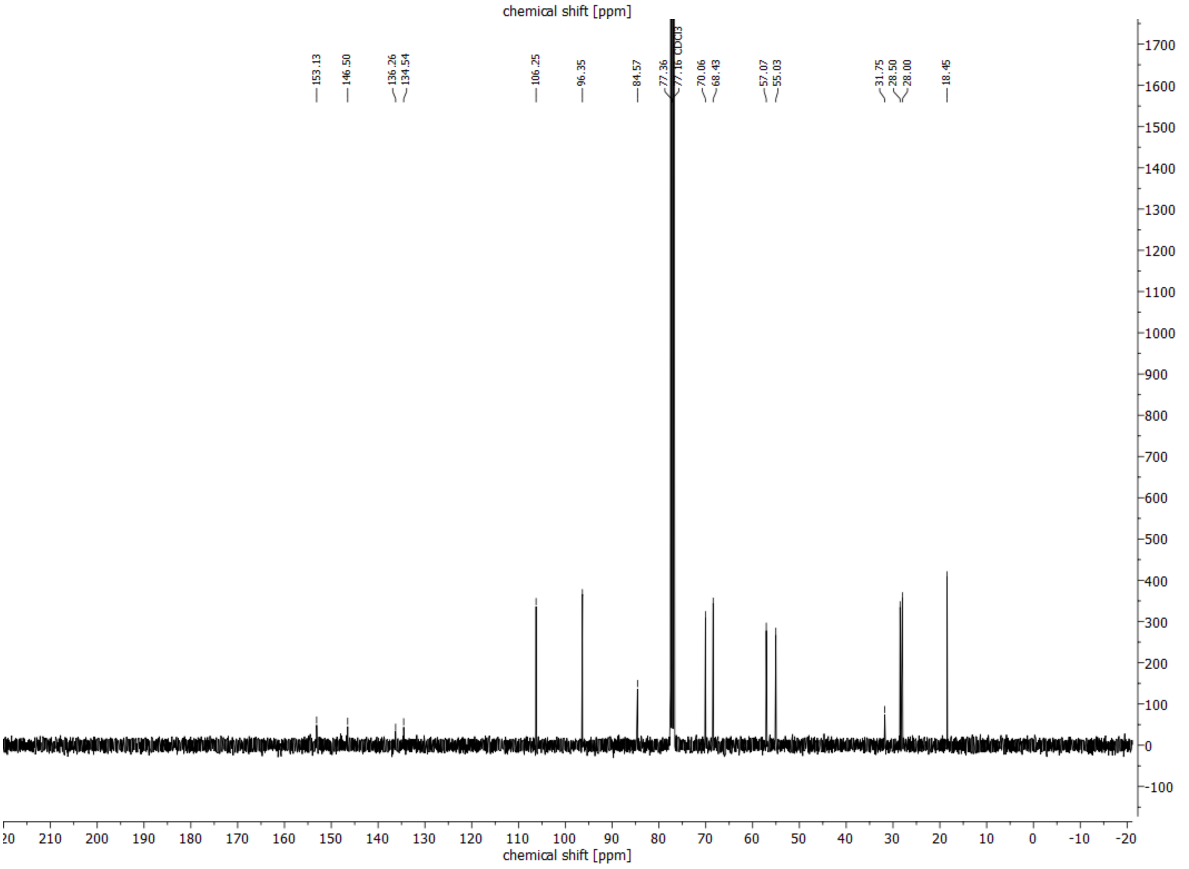

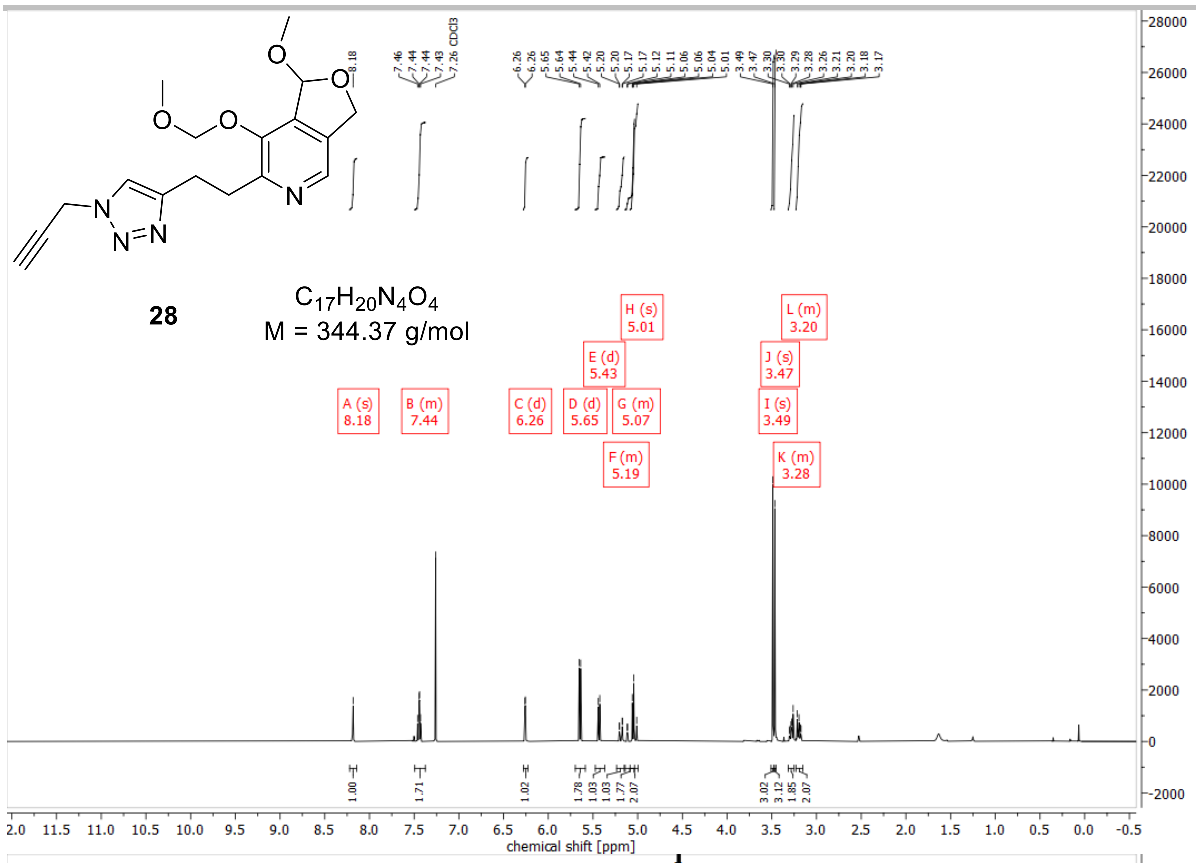
28
$C_{17}H_{20}N_4O_4$
M = 344.37 g/mol
A (s) 8.18
B (m) 7.44
C (d) 6.26
D (d) 5.65
E (d) 5.43
F (m) 5.19
G (m) 5.07
H (s) 5.01
I (s) 3.49
J (s) 3.47
K (m) 3.28
L (m) 3.20
chemical shift [ppm]

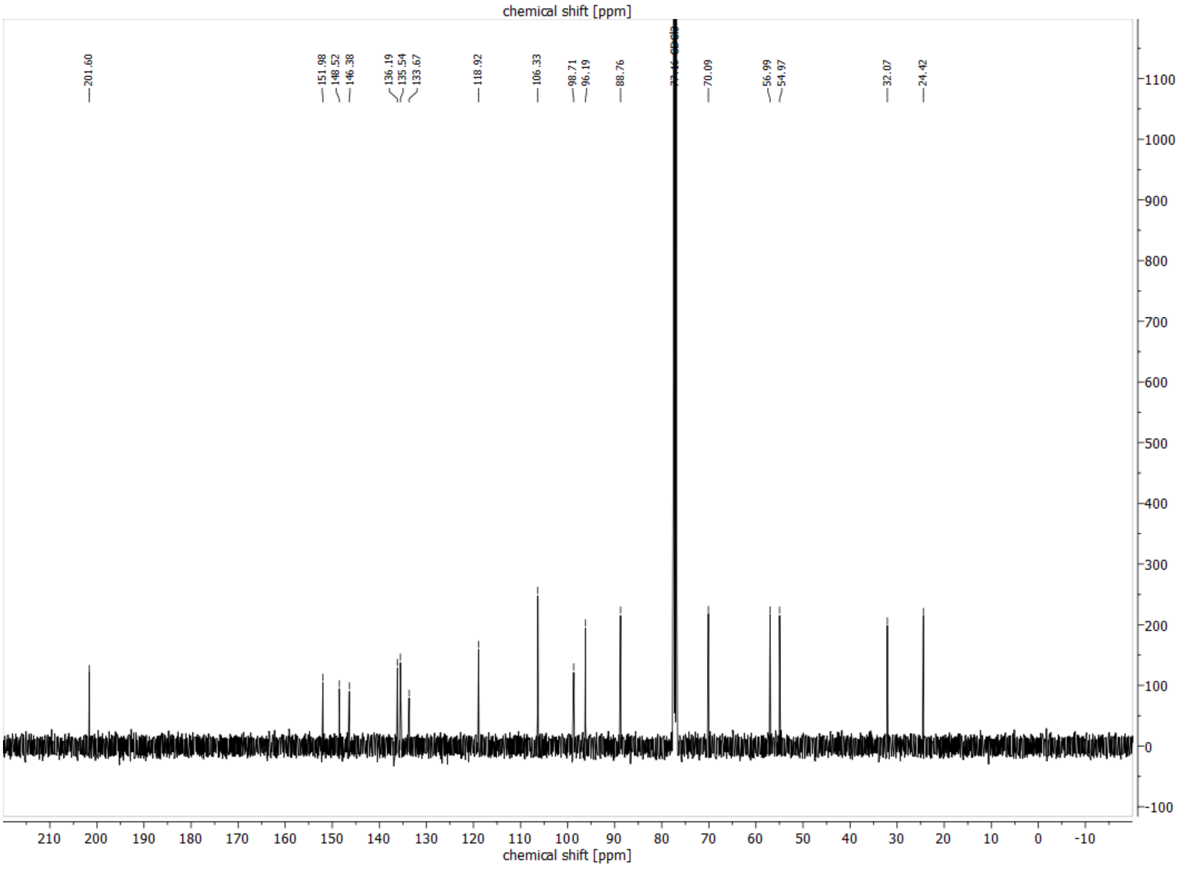
201.60
151.98
148.52
146.38
136.19
135.54
133.67
118.92
106.33
98.71
96.19
88.76
70.09
56.99
54.97
32.07
24.42
chemical shift [ppm]

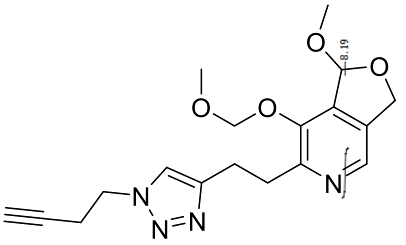

29

$C_{18}H_{22}N_4O_4$
M = 358.39 g/mol

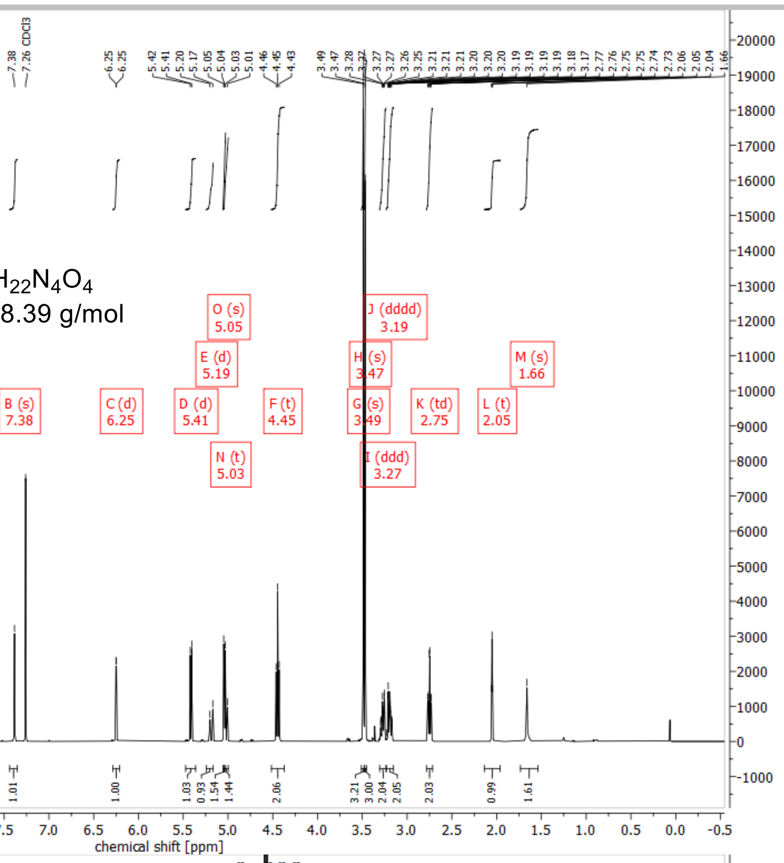

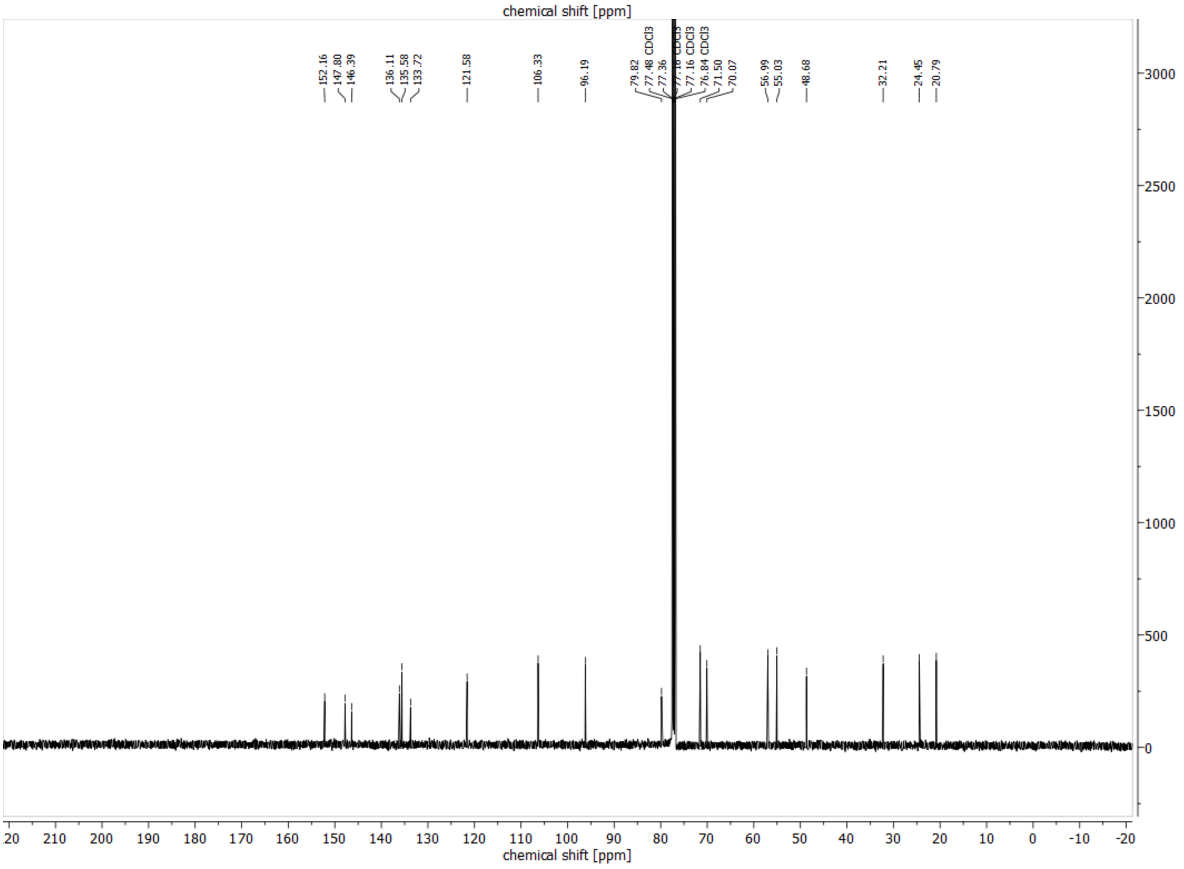

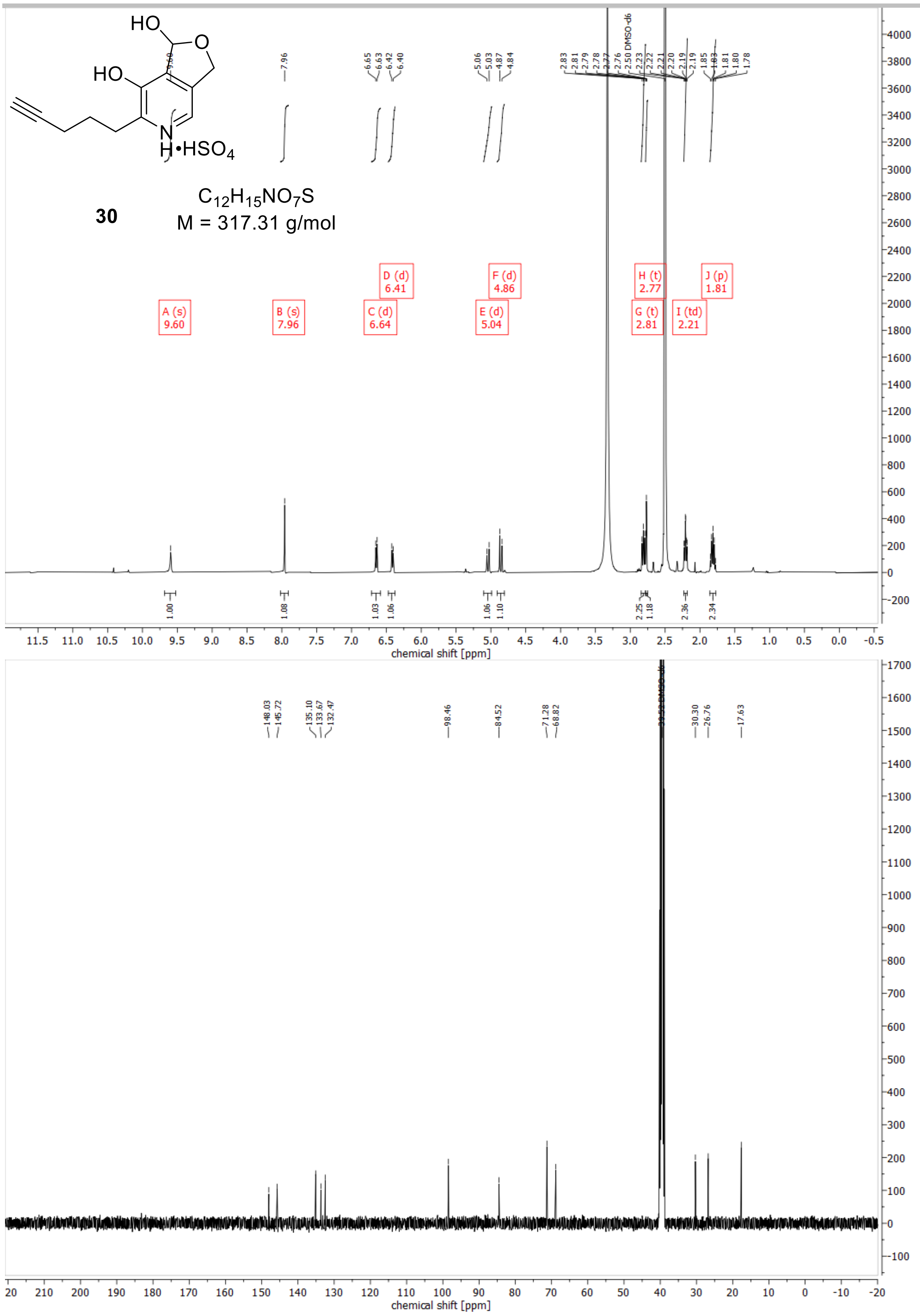
HO
O
HO
N
H•HSO4
30
C12H15NO7S
M = 317.31 g/mol
A (s) 9.60
B (s) 7.96
C (d) 6.64
D (d) 6.41
E (d) 5.04
F (d) 4.86
G (t) 2.81
H (t) 2.77
I (td) 2.21
J (p) 1.81
2.50 DMSO-d6
chemical shift [ppm]
148.03
145.72
135.10
133.67
132.47
98.46
84.52
71.28
68.82
30.30
26.76
17.63
chemical shift [ppm]

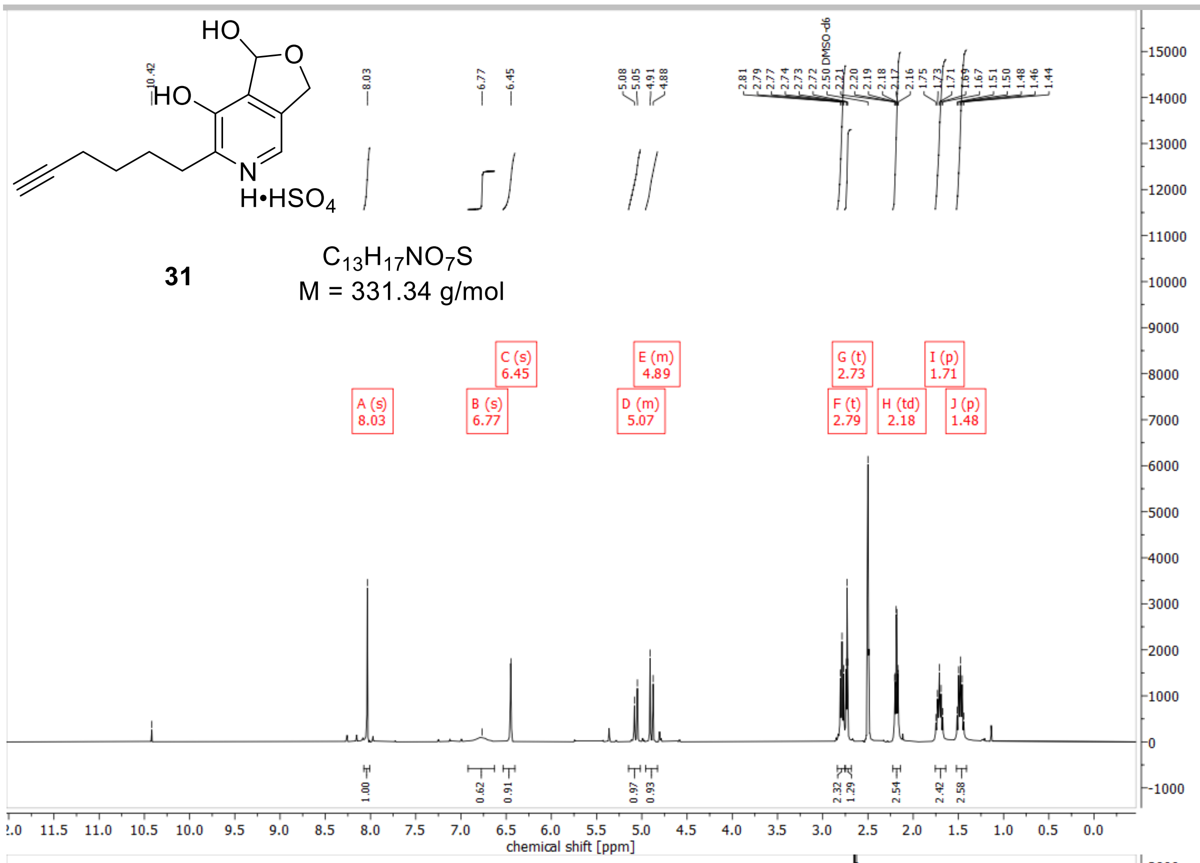

31

$C_{13}H_{17}NO_7S$
M = 331.34 g/mol

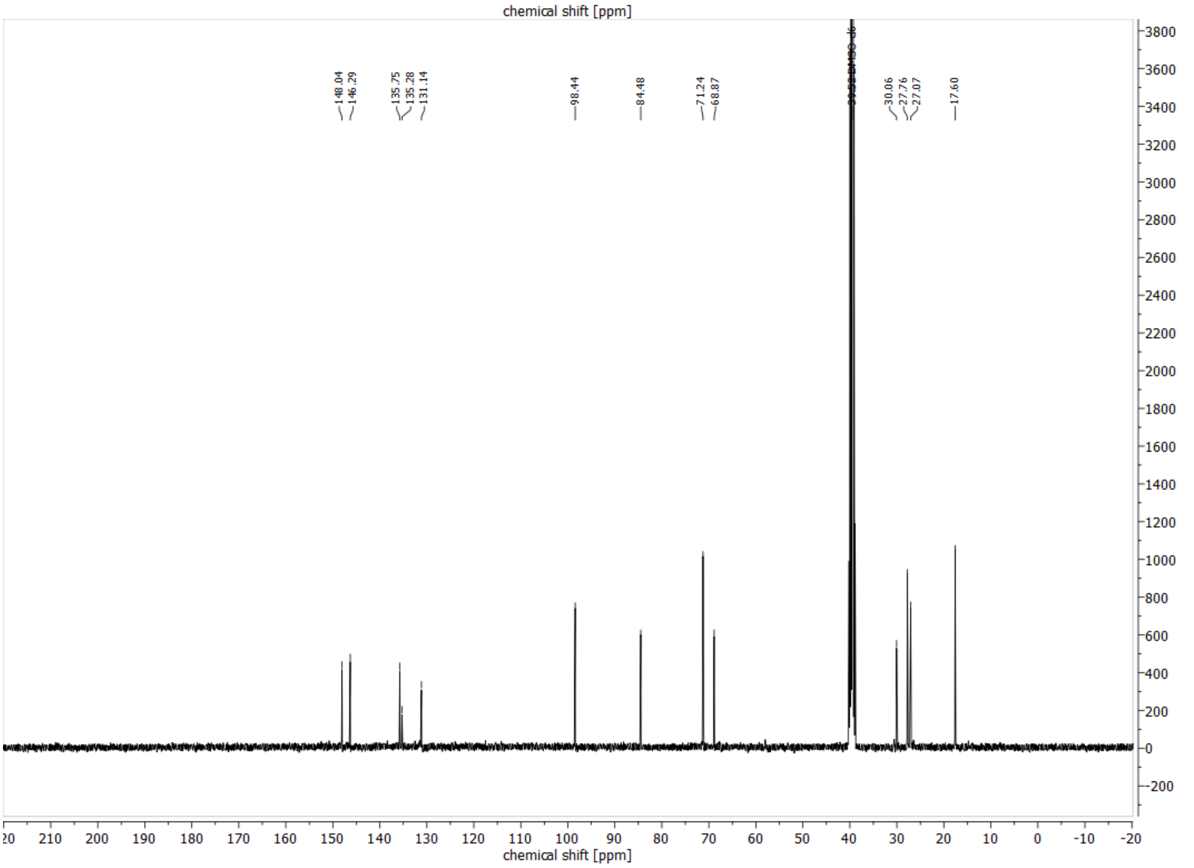

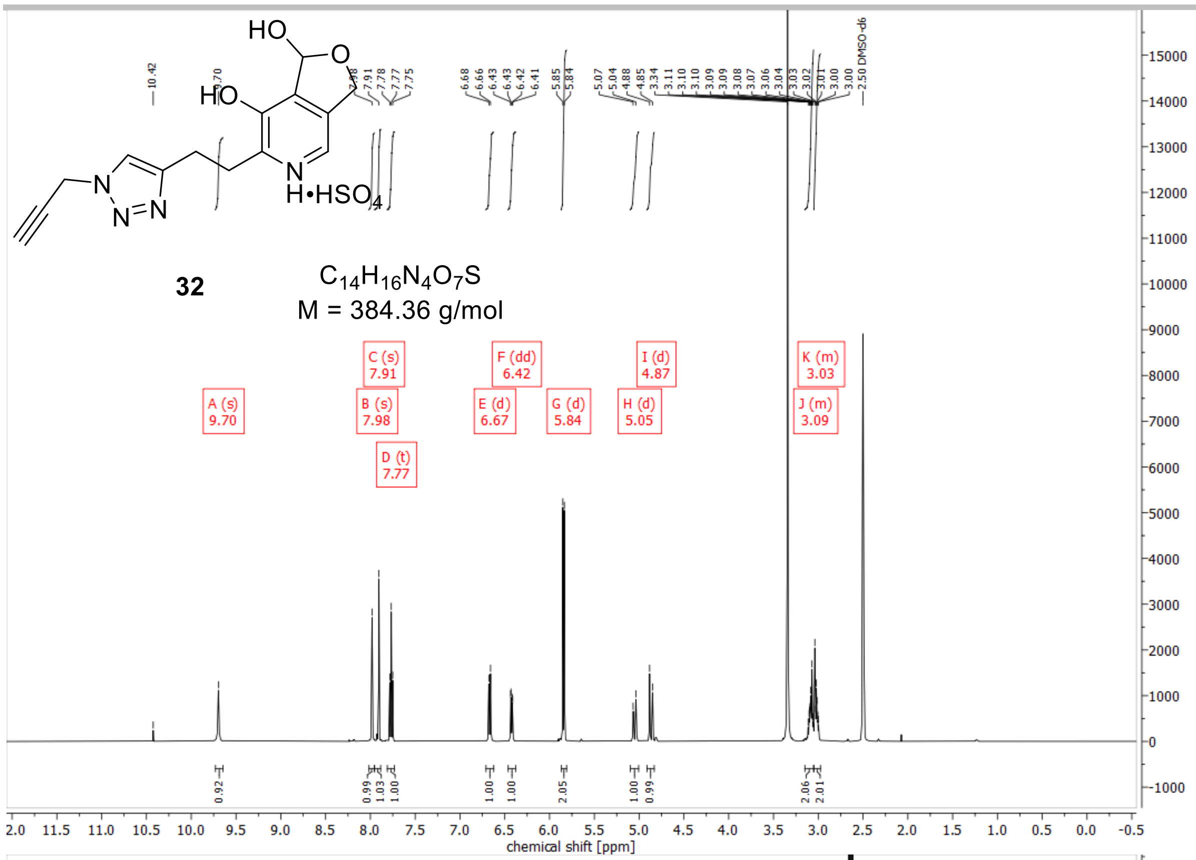
H•HSO4
32
C14H16N4O7S
M = 384.36 g/mol
A (s) 9.70
B (s) 7.98
C (s) 7.91
D (t) 7.77
E (d) 6.67
F (dd) 6.42
G (d) 5.84
H (d) 5.05
I (d) 4.87
J (m) 3.09
K (m) 3.03
2.50 DMSO-d6
chemical shift [ppm]

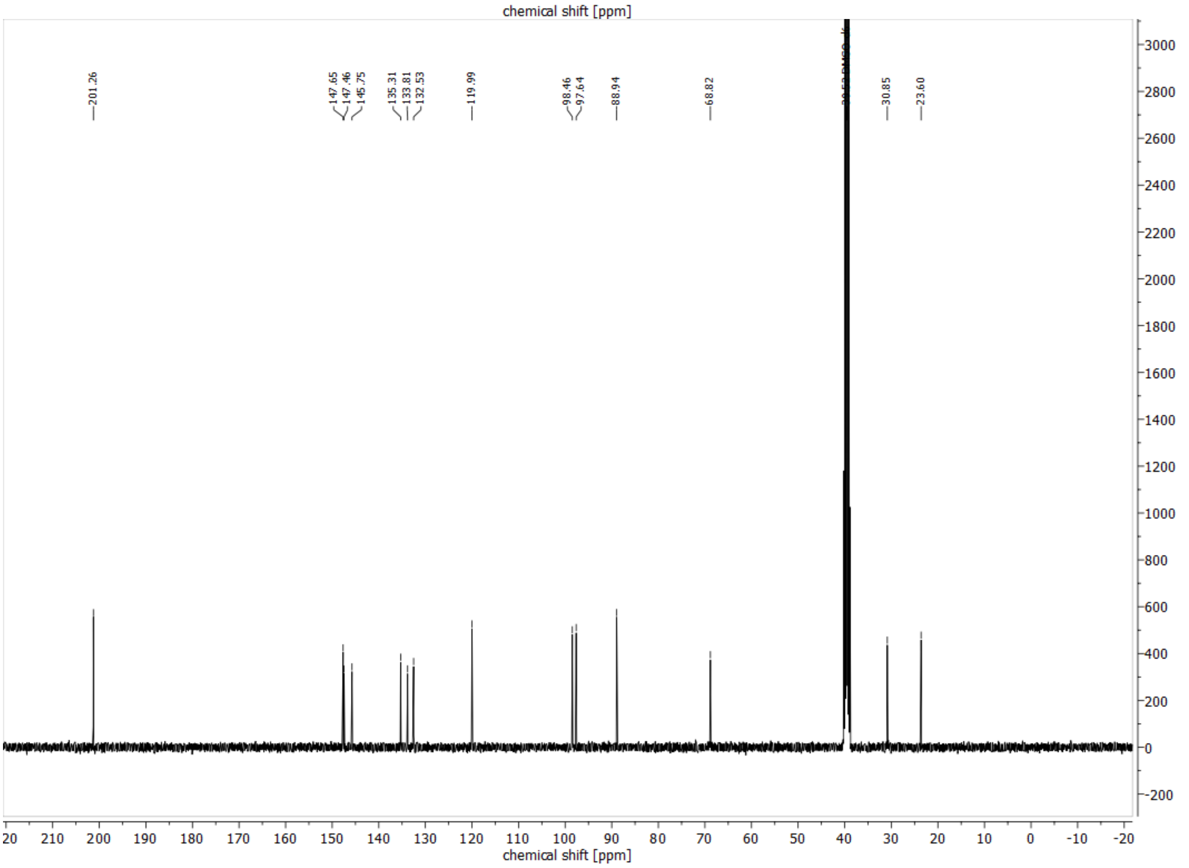
201.26
147.65
147.46
145.75
135.31
133.81
132.53
119.99
98.46
97.64
88.94
68.82
30.85
23.60
chemical shift [ppm]

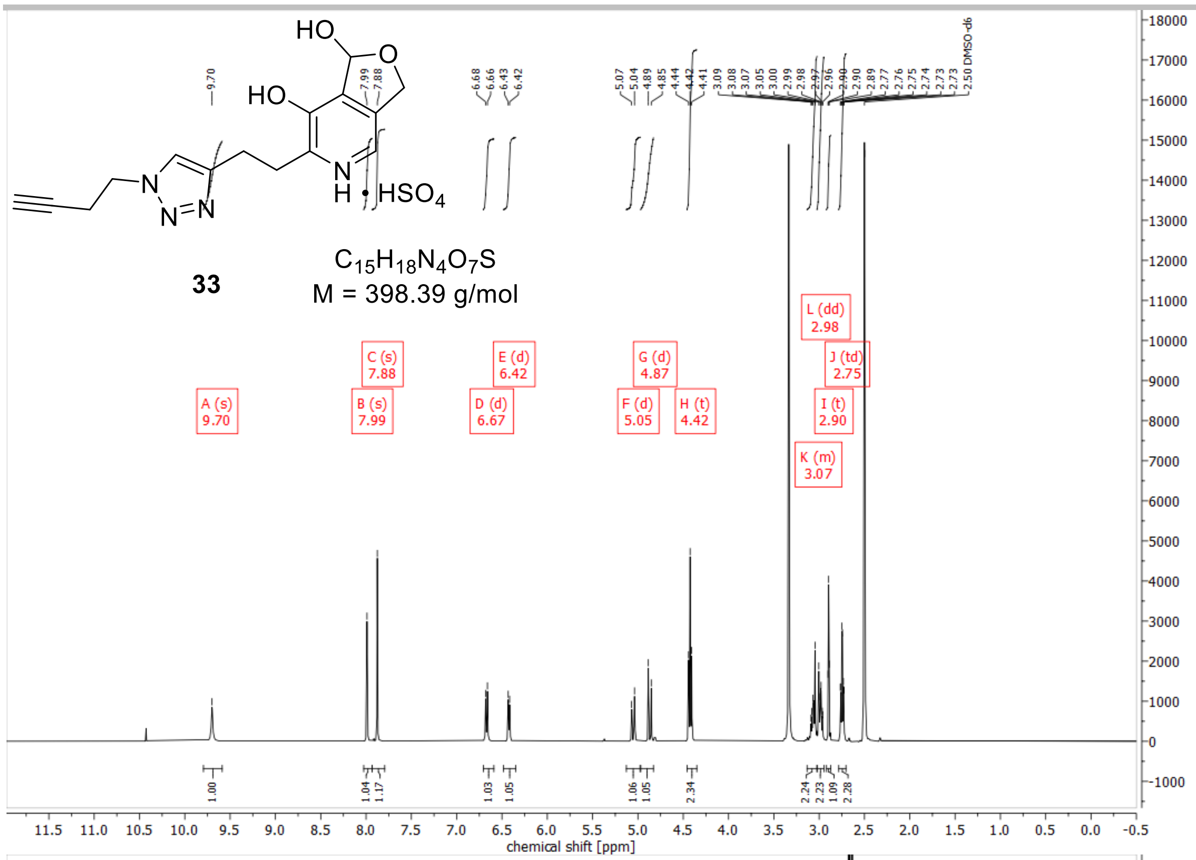

33

$C_{15}H_{18}N_4O_7S$
M = 398.39 g/mol

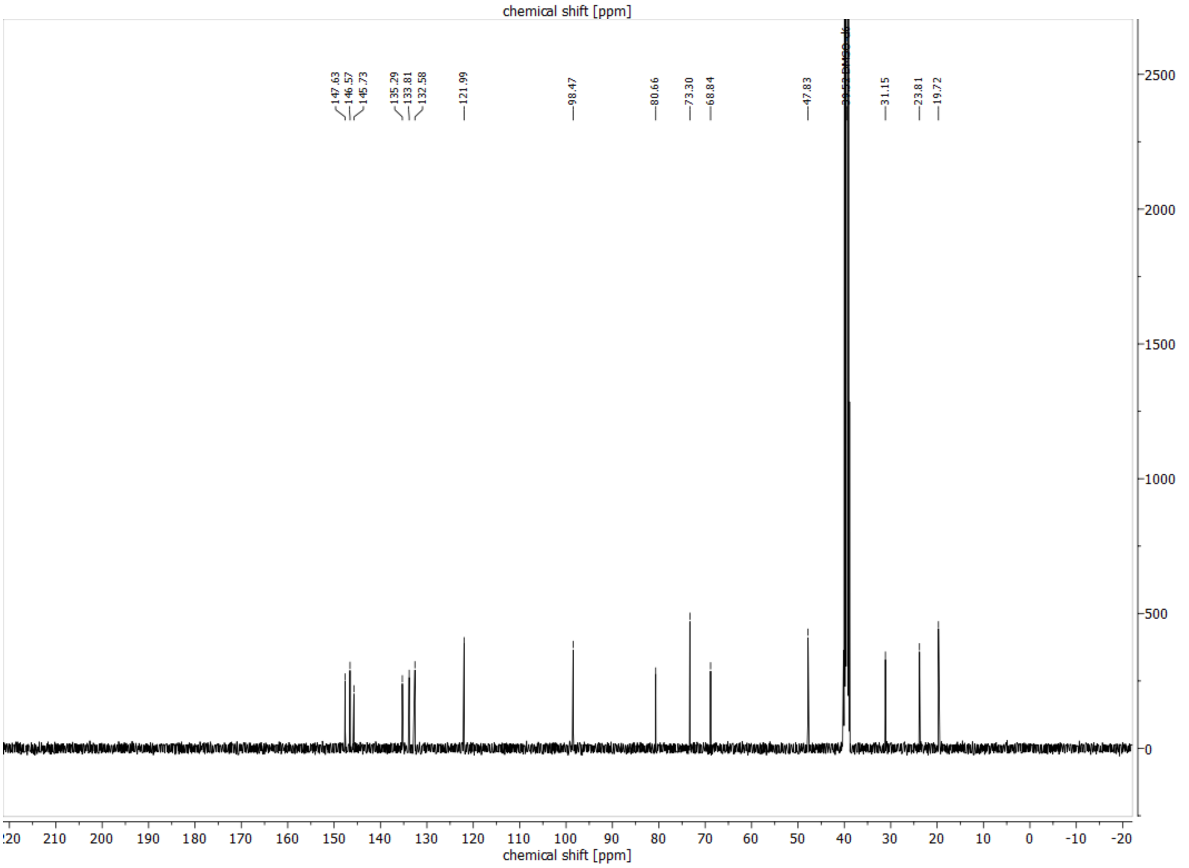

34

$C_{10}H_{11}NO_7S$

M = 289.26 g/mol

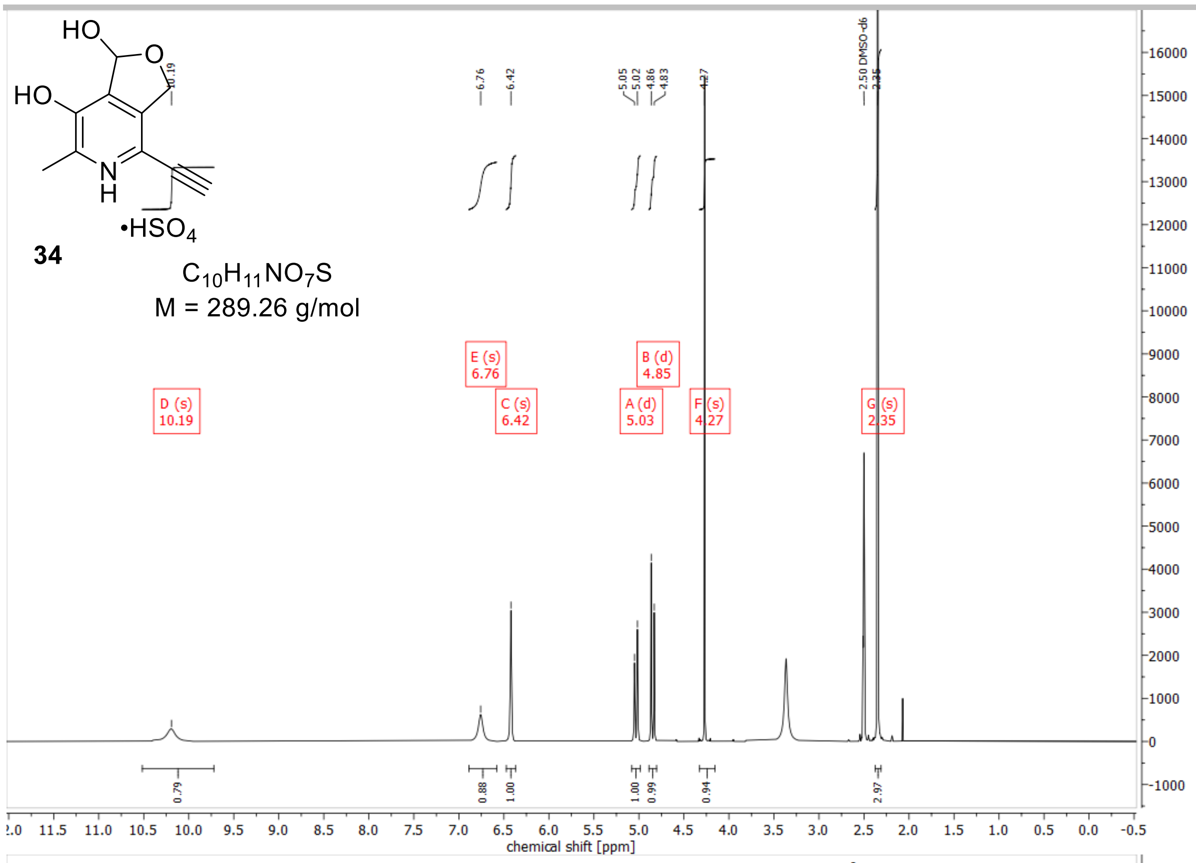

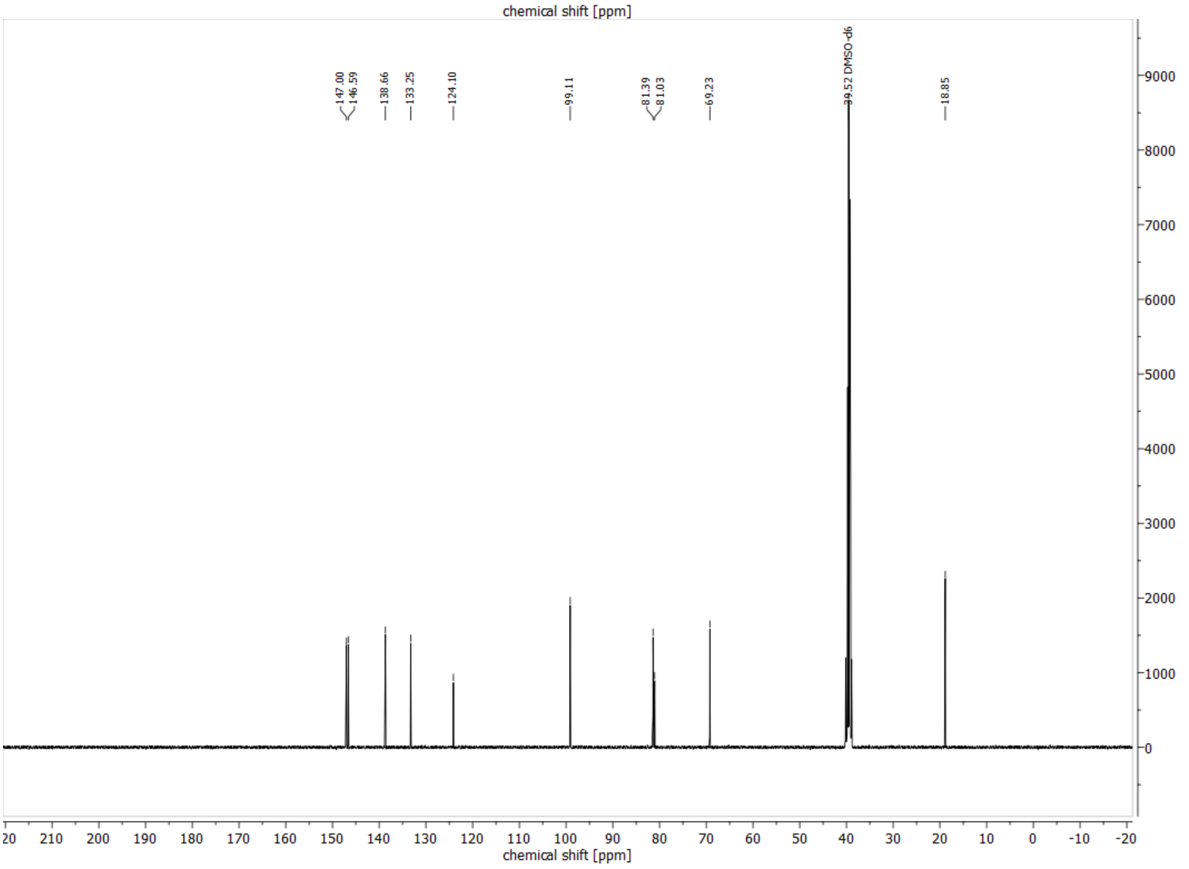

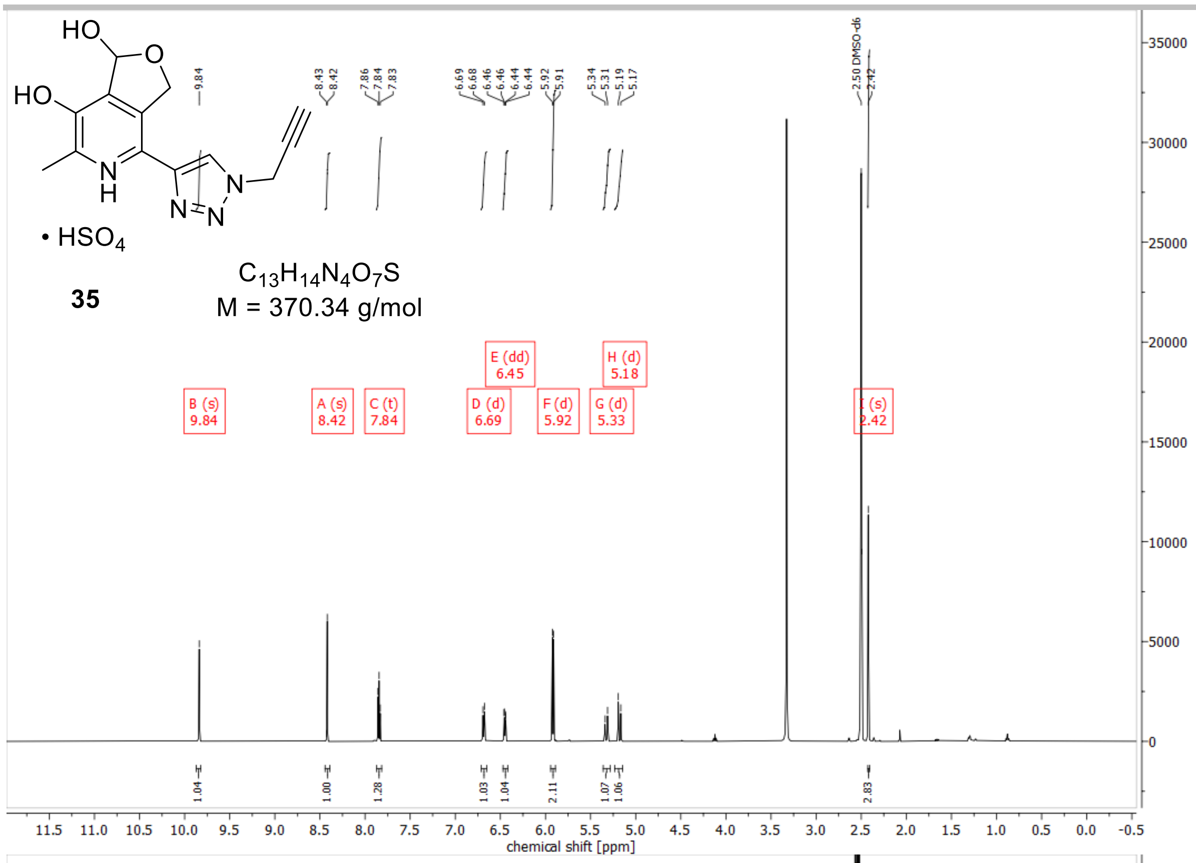
35
$C_{13}H_{14}N_4O_7S$
M = 370.34 g/mol
B (s) 9.84
A (s) 8.42
C (t) 7.84
D (d) 6.69
E (dd) 6.45
F (d) 5.92
G (d) 5.33
H (d) 5.18
I (s) 2.42
2.50 DMSO-d6
chemical shift [ppm]

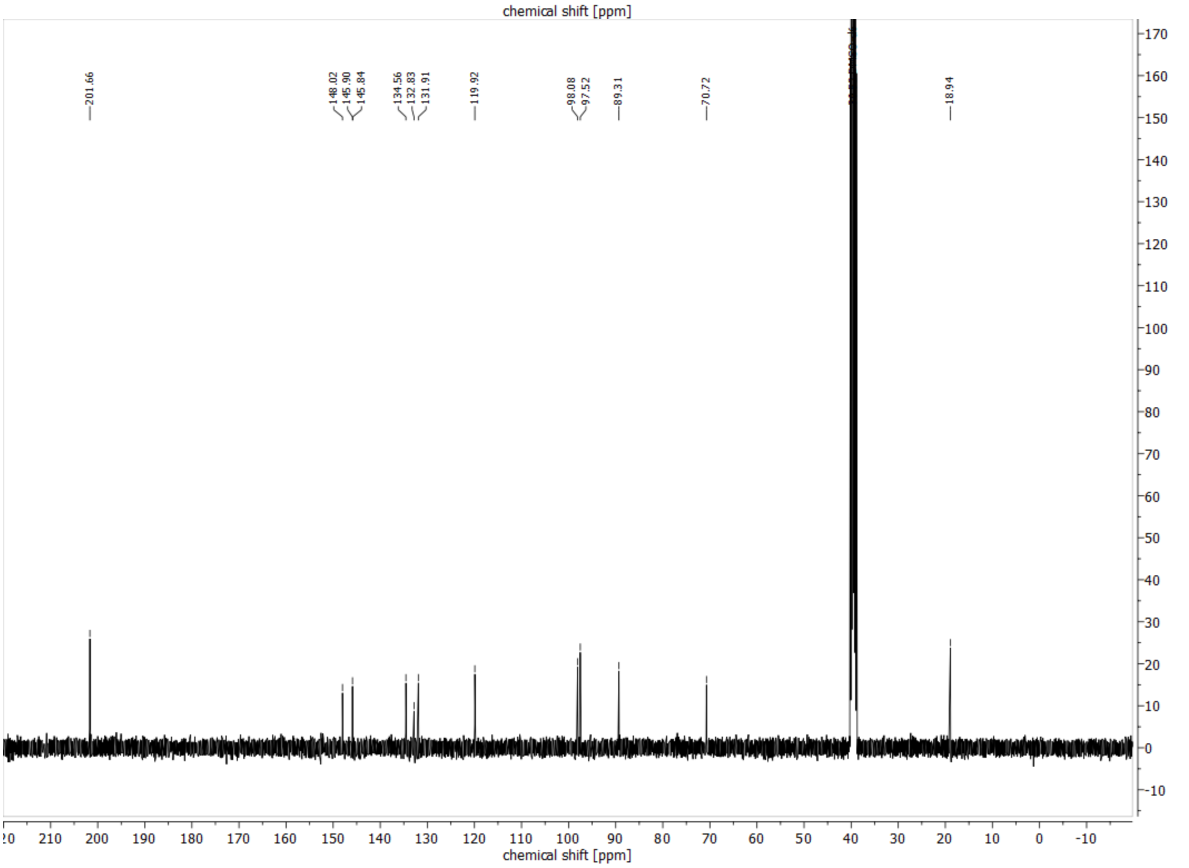
201.66
148.02
145.90
145.84
134.56
132.83
131.91
119.92
98.08
97.52
89.31
70.72
18.94
chemical shift [ppm]

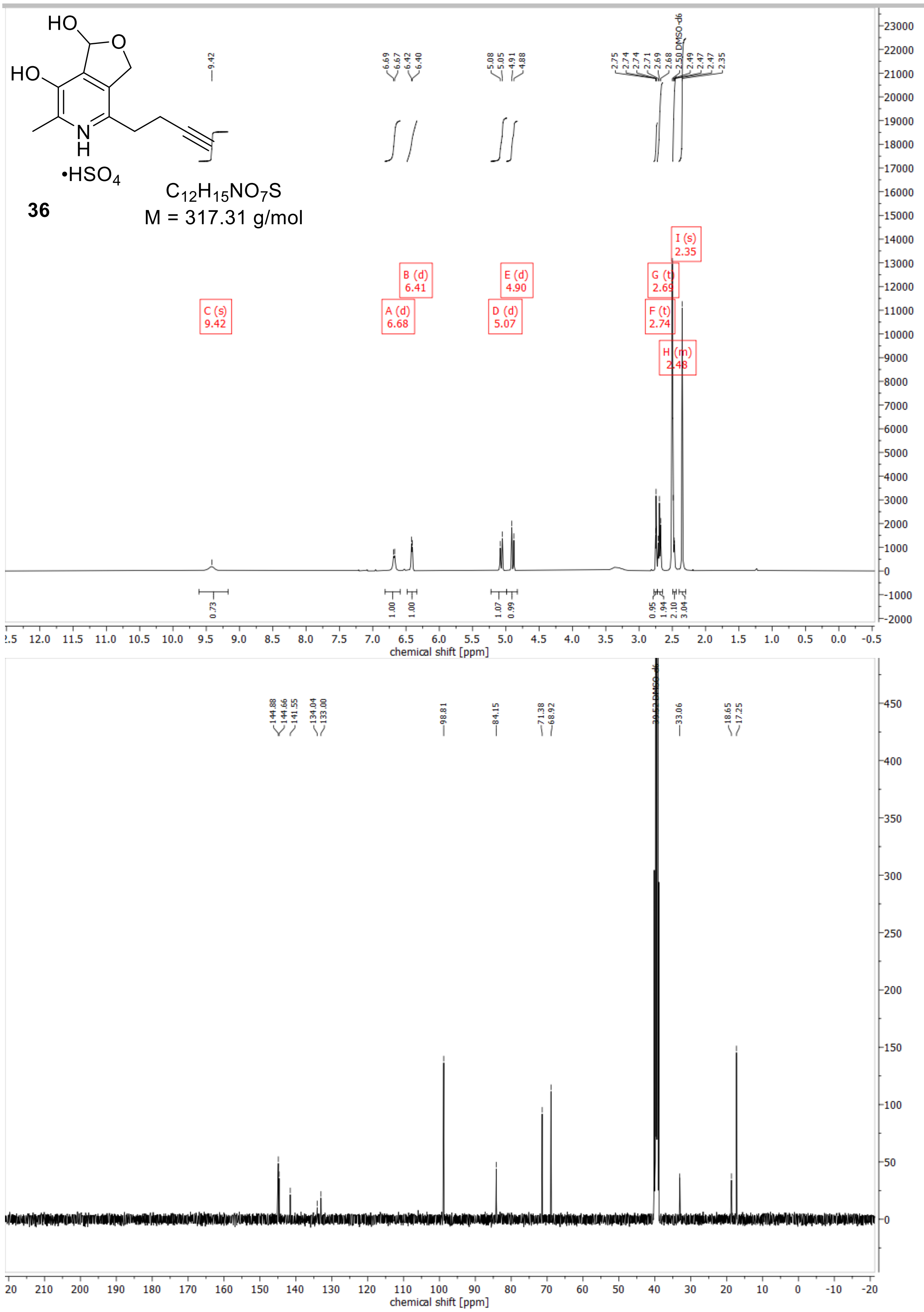
HO
O
HO
N
H
•HSO4
36
C12H15NO7S
M = 317.31 g/mol
C (s) 9.42
A (d) 6.68
B (d) 6.41
D (d) 5.07
E (d) 4.90
F (t) 2.74
G (t) 2.69
H (m) 2.48
I (s) 2.35
chemical shift [ppm]
chemical shift [ppm]

37

$C_{11}H_{11}NO_3$
M = 205.21 g/mol

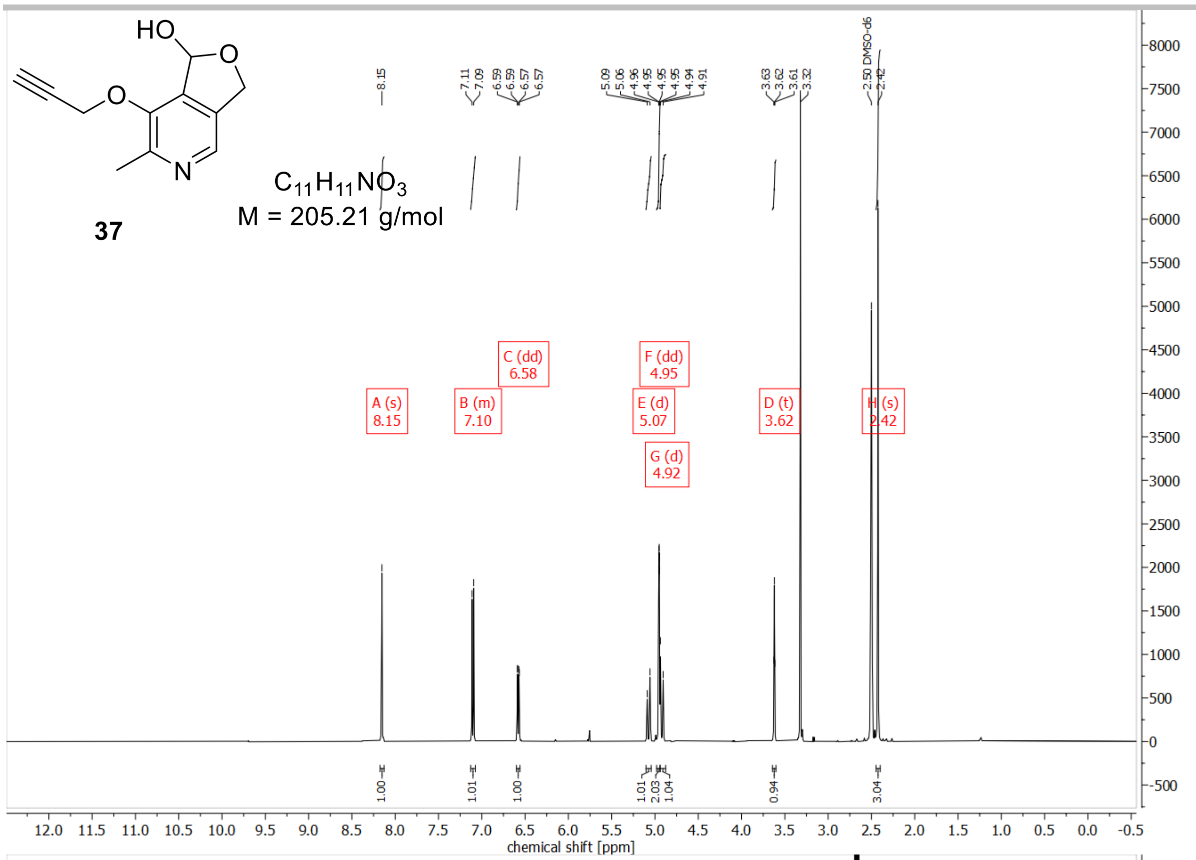

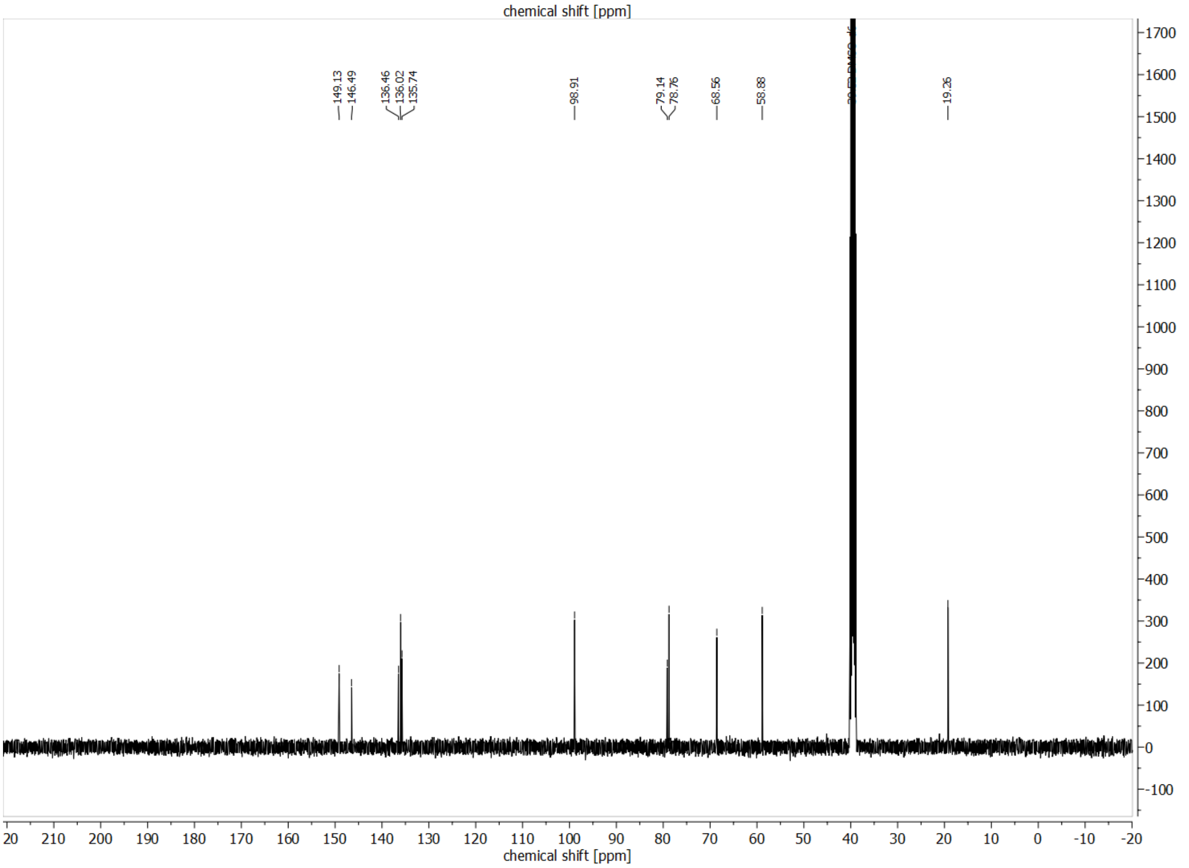

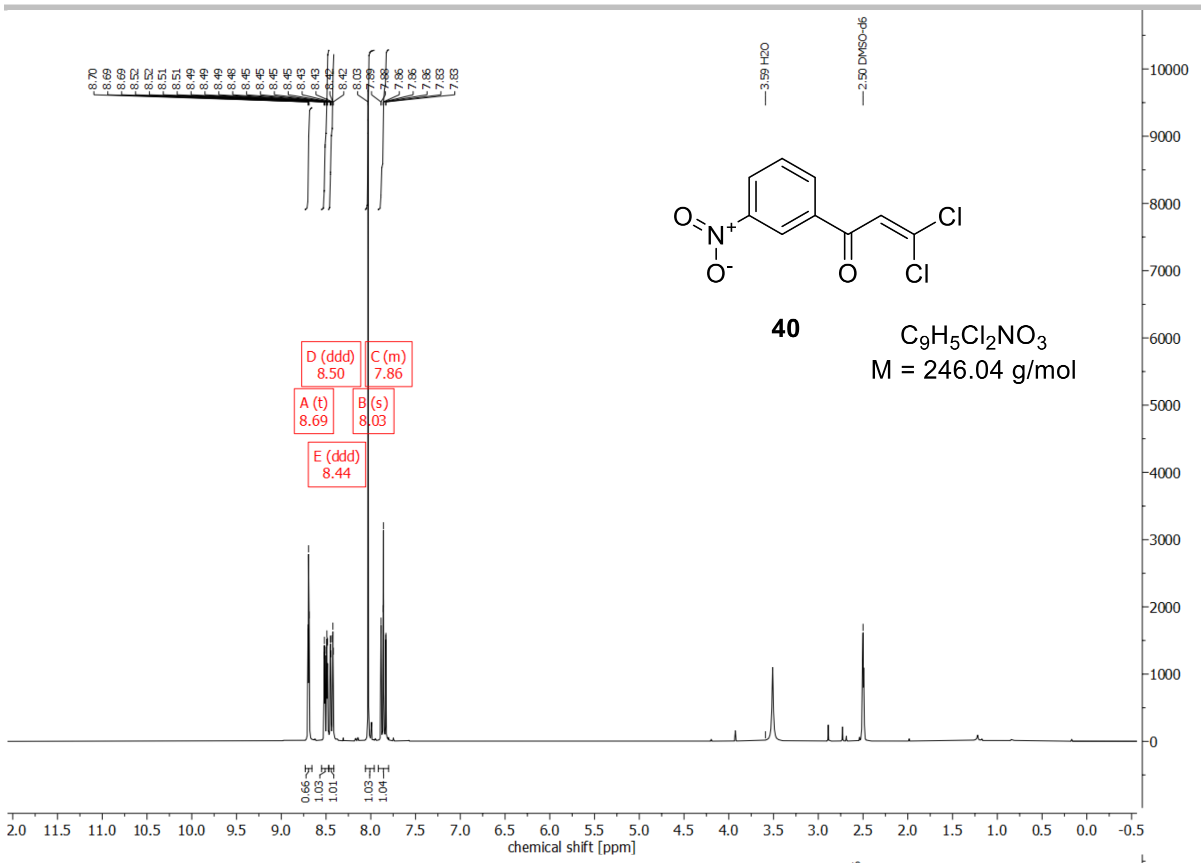
40
$C_9H_5Cl_2NO_3$
M = 246.04 g/mol
chemical shift [ppm]

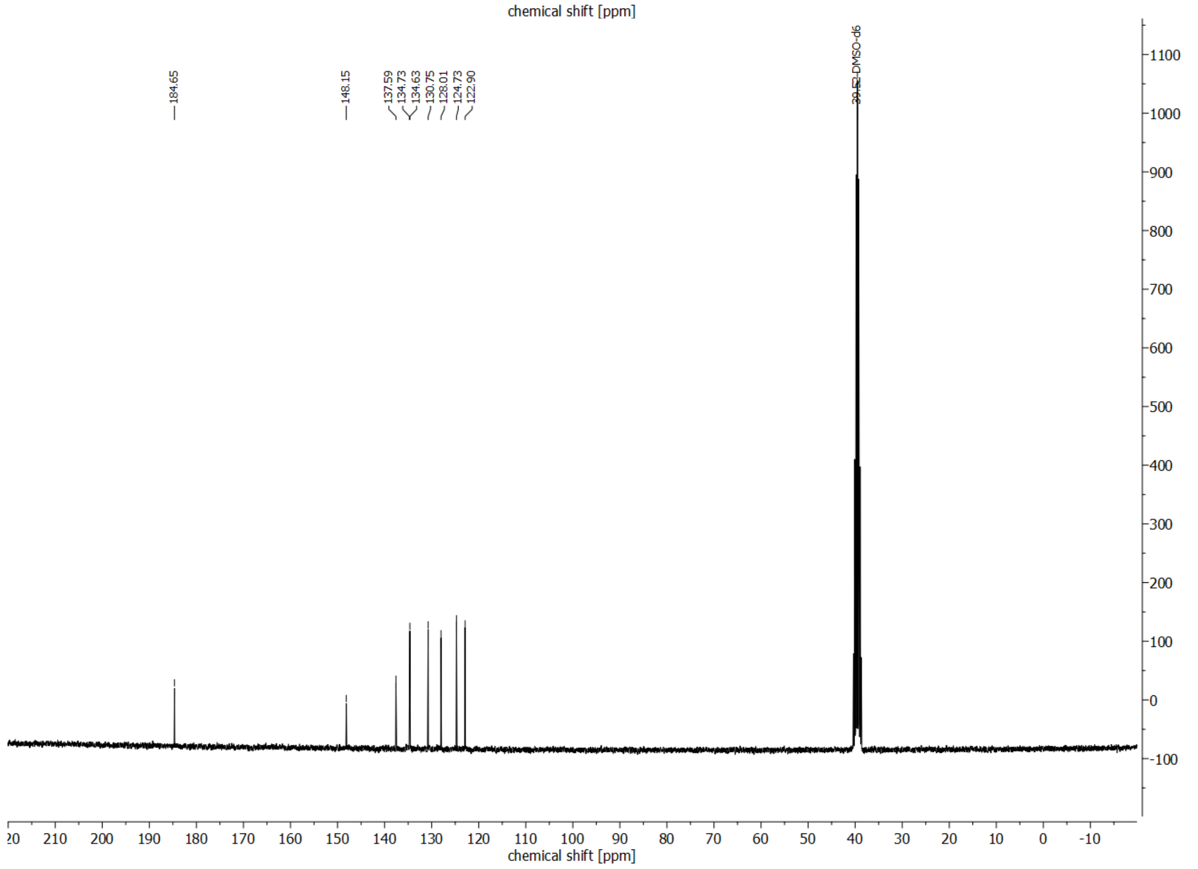
chemical shift [ppm]

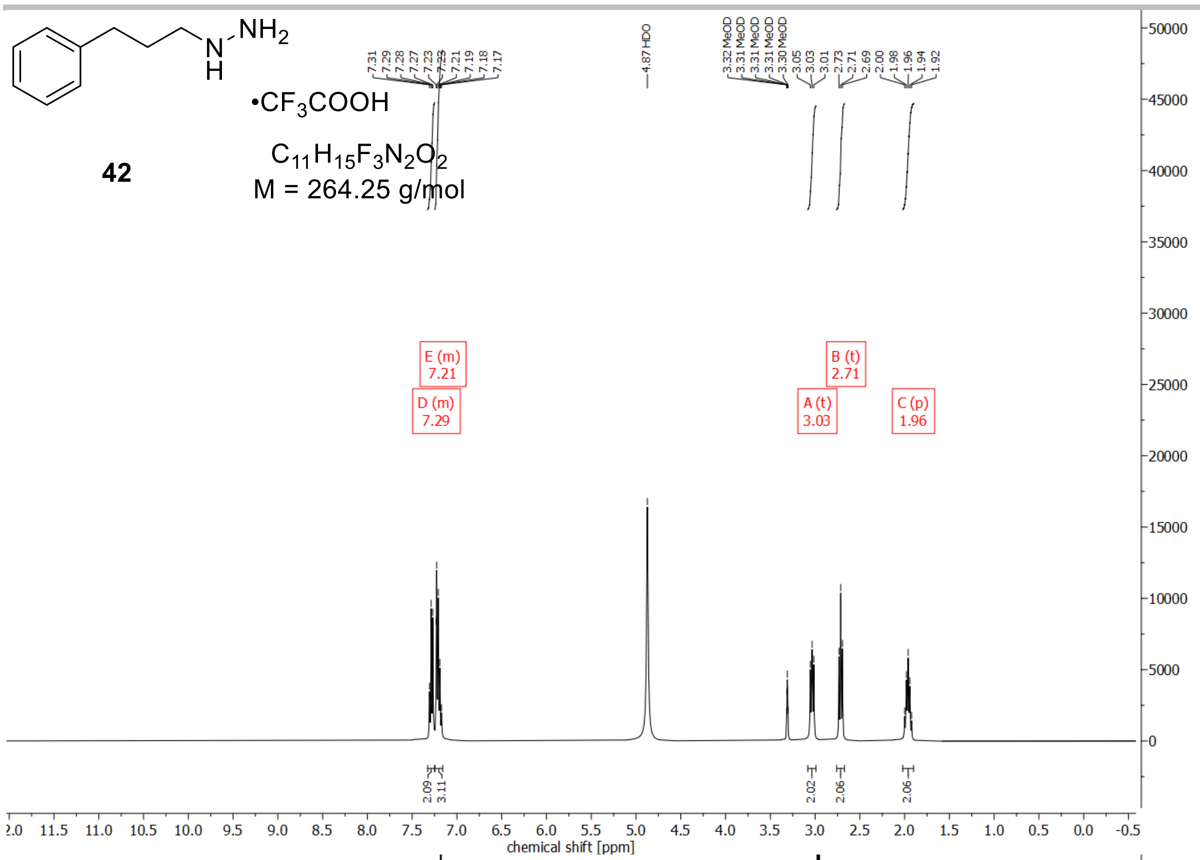
42
•CF3COOH
C11H15F3N2O2
M = 264.25 g/mol
7.31 7.29 7.28 7.27 7.23 7.23 7.21 7.19 7.18 7.17
4.87 HDO
3.32 MeOD 3.31 MeOD 3.31 MeOD 3.31 MeOD 3.30 MeOD 3.05 3.03 3.01 2.73 2.71 2.69 2.00 1.98 1.96 1.94 1.92
E (m) 7.21
D (m) 7.29
B (t) 2.71
A (t) 3.03
C (p) 1.96
2.09 3.11 2.02 2.06 2.06
chemical shift [ppm]

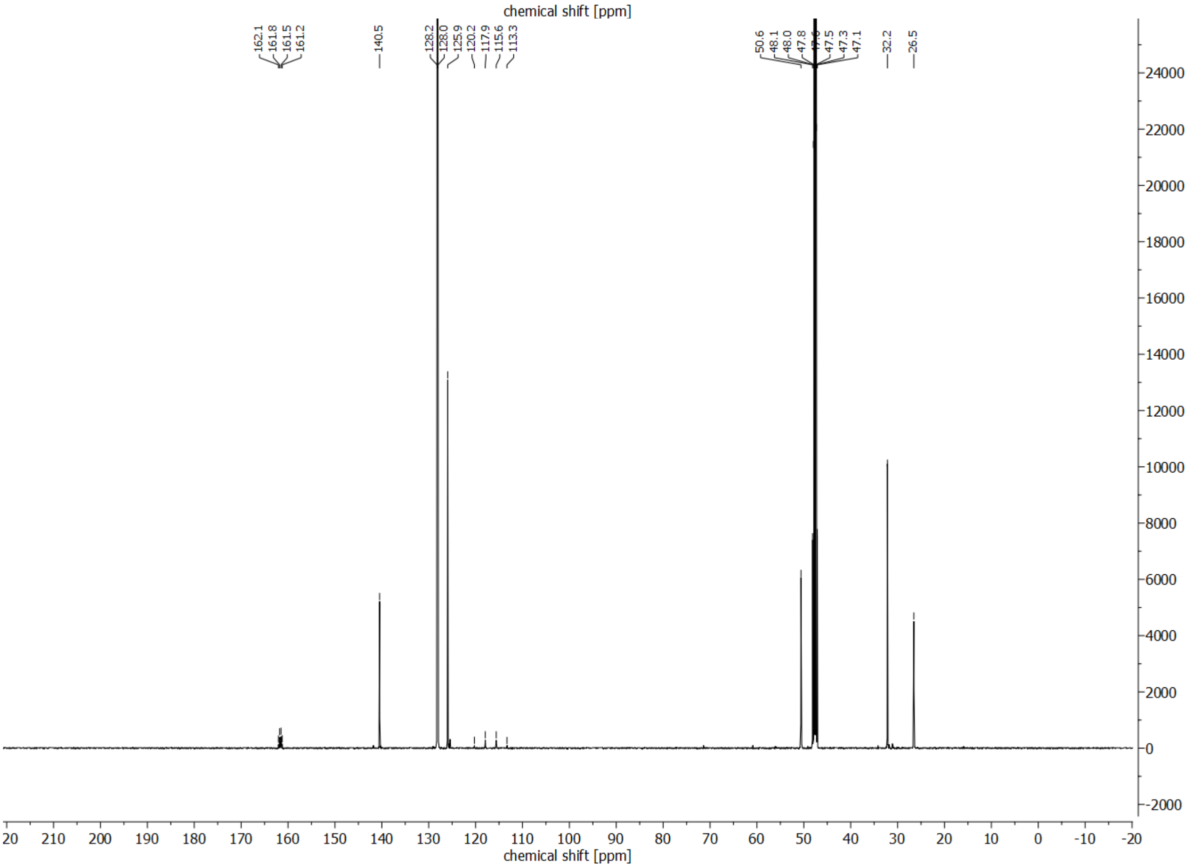
162.1 161.8 161.5 161.2
140.5
128.2 128.0 125.9 120.2 117.9 115.6 113.3
50.6 48.1 48.0 47.8 47.6 47.5 47.3 47.1
32.2
26.5
chemical shift [ppm]

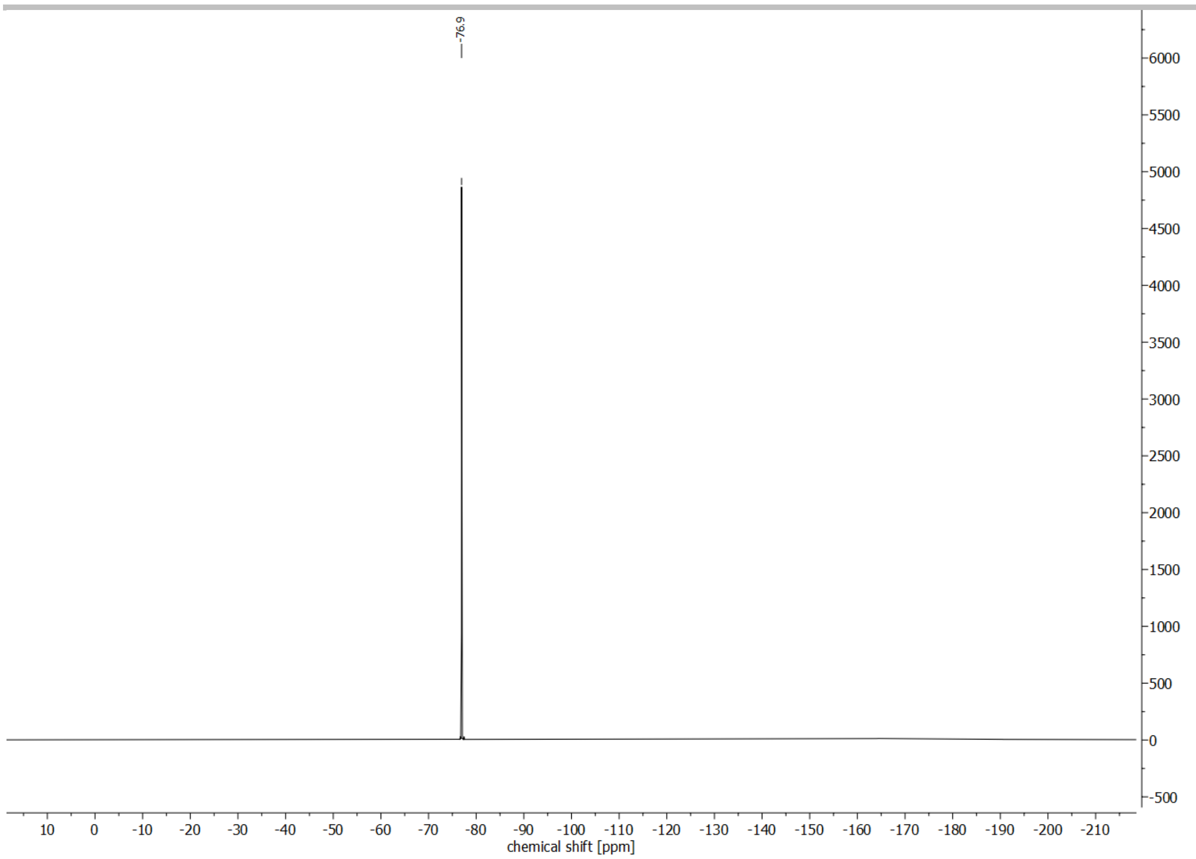
-76.9
chemical shift [ppm]

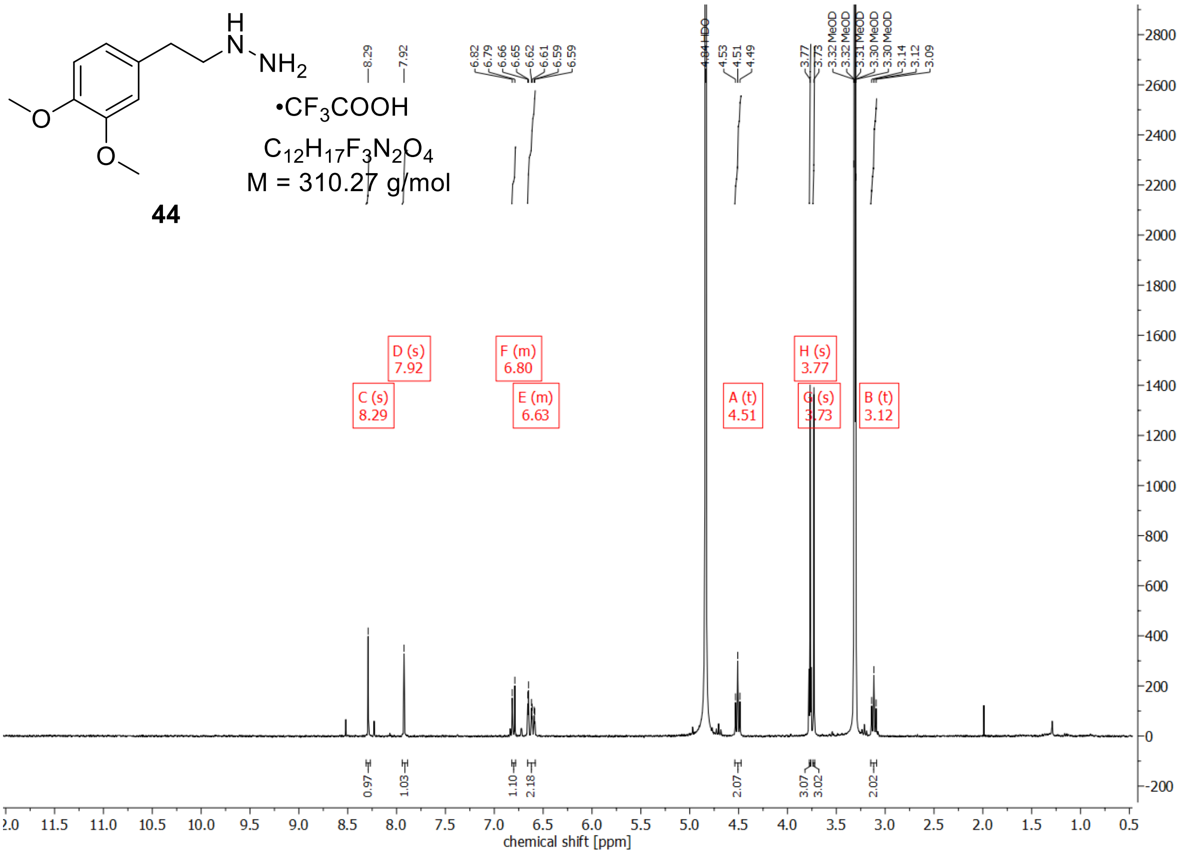
•CF3COOH
C12H17F3N2O4
M = 310.27 g/mol
44
C (s)
8.29
D (s)
7.92
F (m)
6.80
E (m)
6.63
A (t)
4.51
H (s)
3.77
G (s)
3.73
B (t)
3.12
4.84 HDO
3.32 MeOD
3.31 MeOD
3.30 MeOD
chemical shift [ppm]

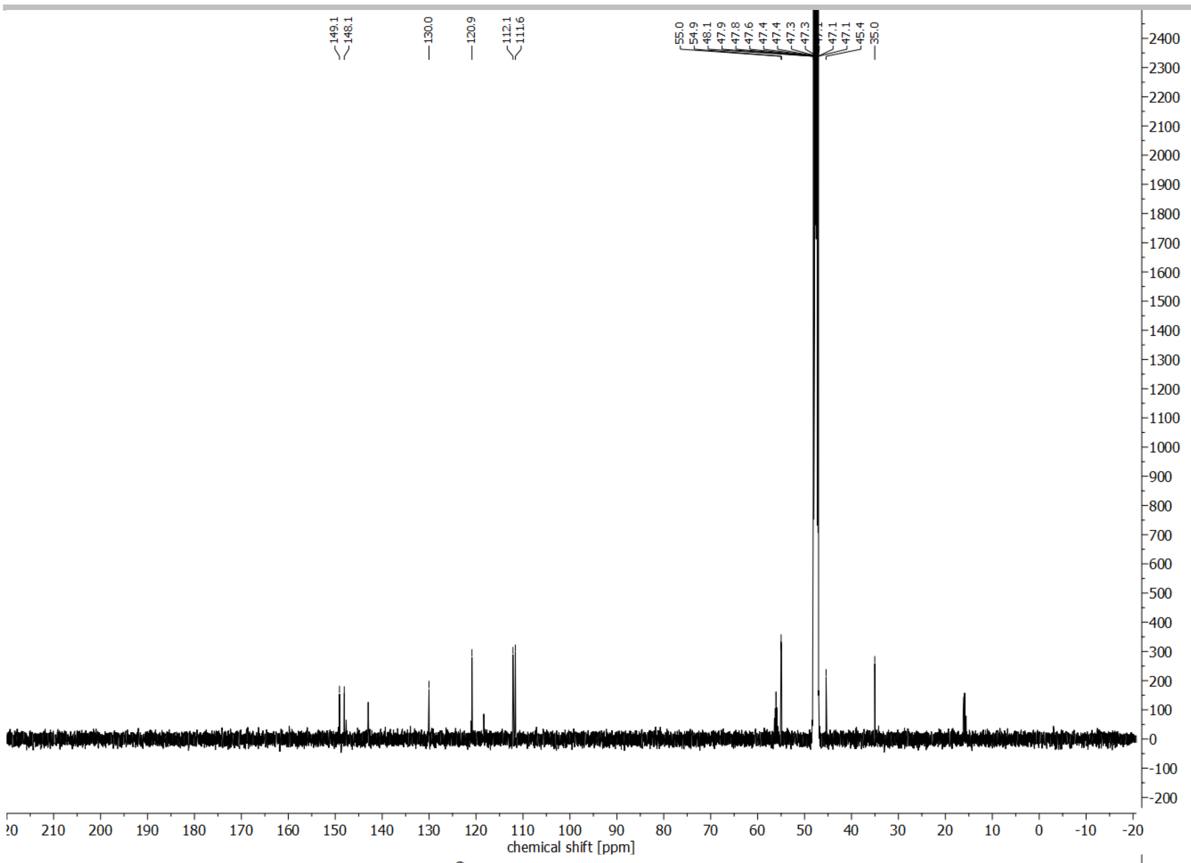
149.1
148.1
130.0
120.9
112.1
111.6
55.0
54.9
48.1
47.9
47.8
47.6
47.4
47.4
47.3
47.3
47.1
47.1
45.4
35.0
chemical shift [ppm]

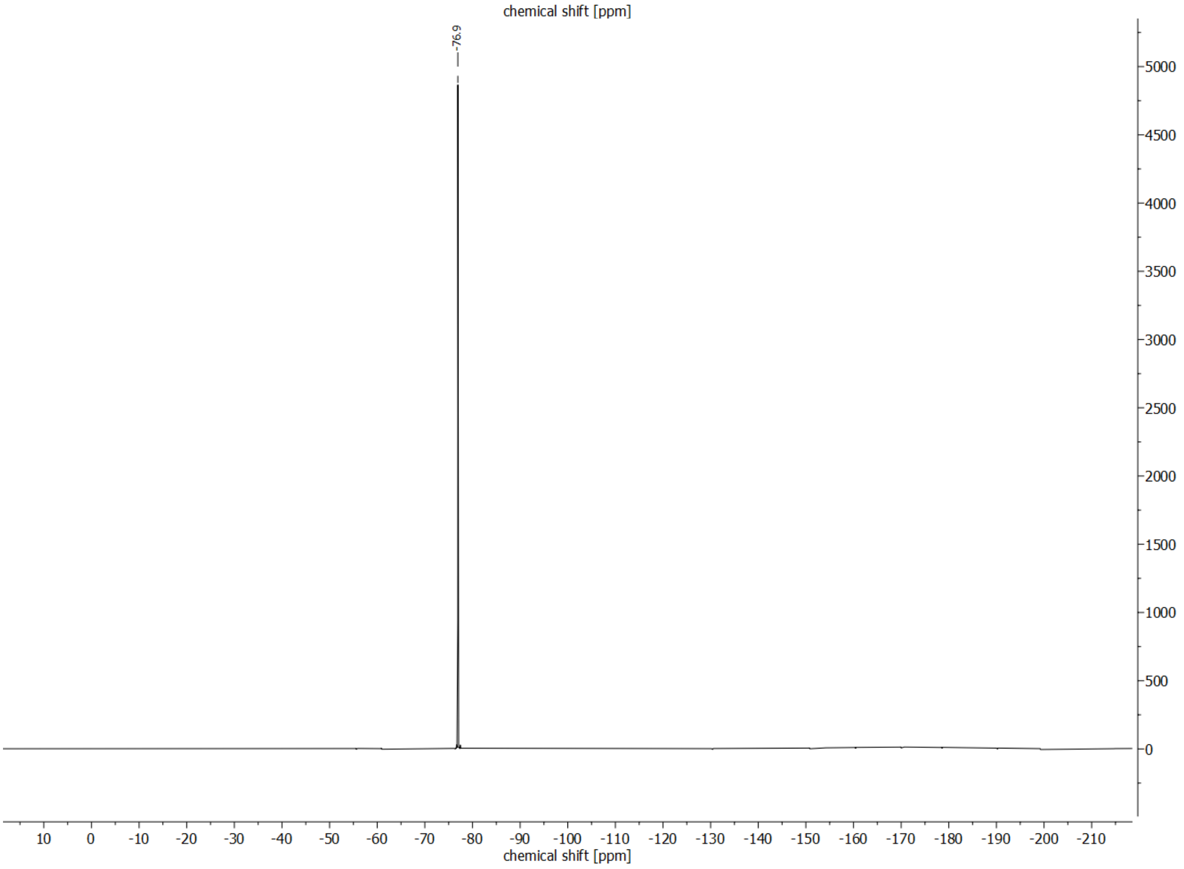
-76.9
chemical shift [ppm]

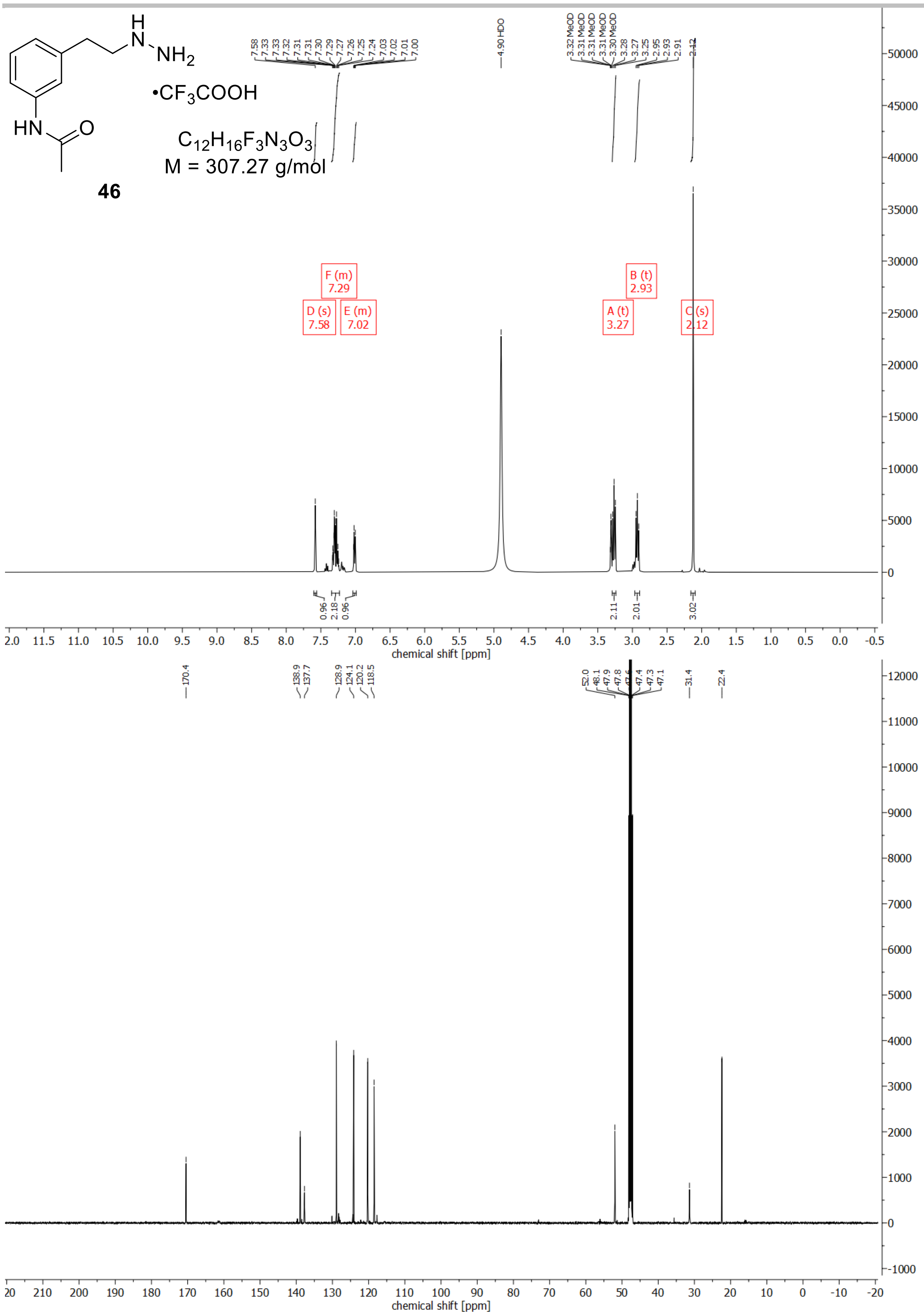
•CF3COOH
C12H16F3N3O3
M = 307.27 g/mol
46
F (m) 7.29
D (s) 7.58
E (m) 7.02
B (t) 2.93
A (t) 3.27
C (s) 2.12
chemical shift [ppm]
chemical shift [ppm]

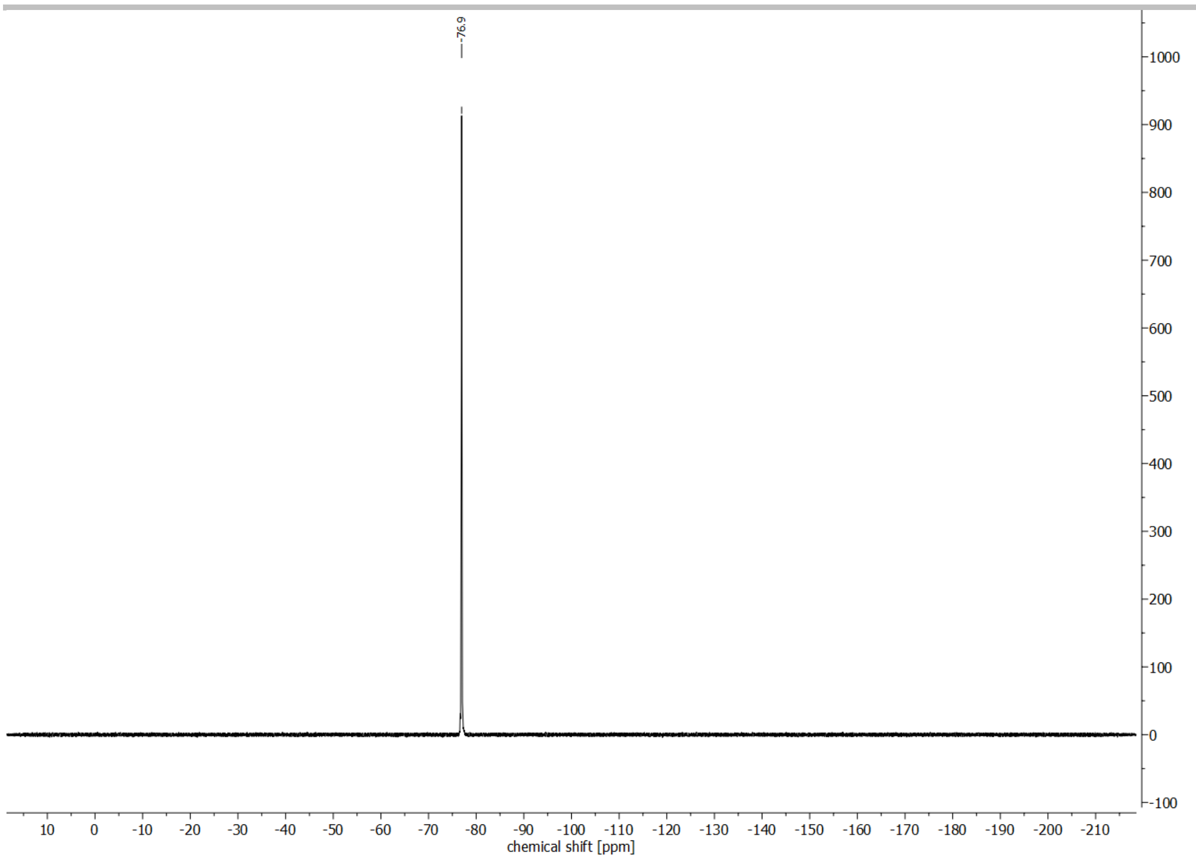

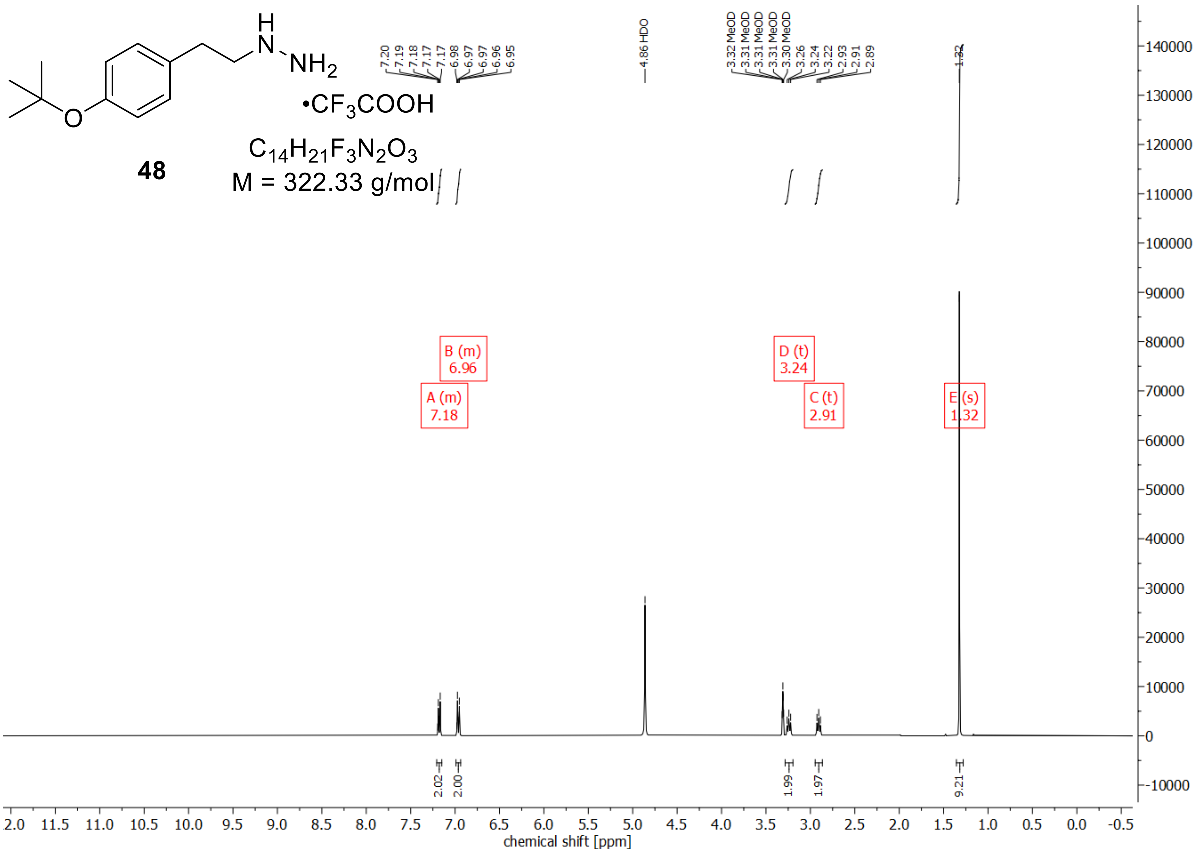
•CF3COOH
48
C14H21F3N2O3
M = 322.33 g/mol
A (m) 7.18
B (m) 6.96
C (t) 2.91
D (t) 3.24
E (s) 1.32
4.86 HDO
chemical shift [ppm]

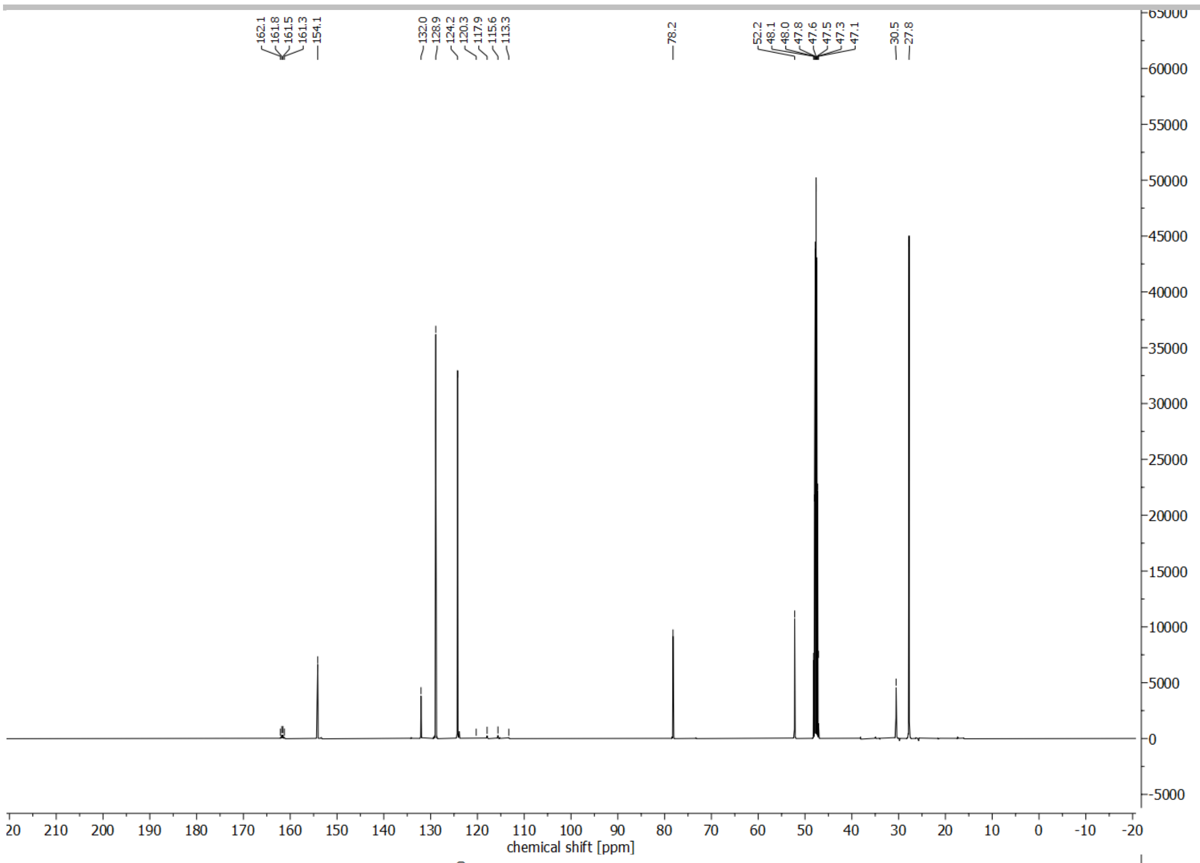
162.1
161.8
161.5
161.3
154.1
132.0
128.9
124.2
120.3
117.9
115.6
113.3
78.2
52.2
48.1
48.0
47.8
47.6
47.5
47.3
47.1
30.5
27.8
chemical shift [ppm]

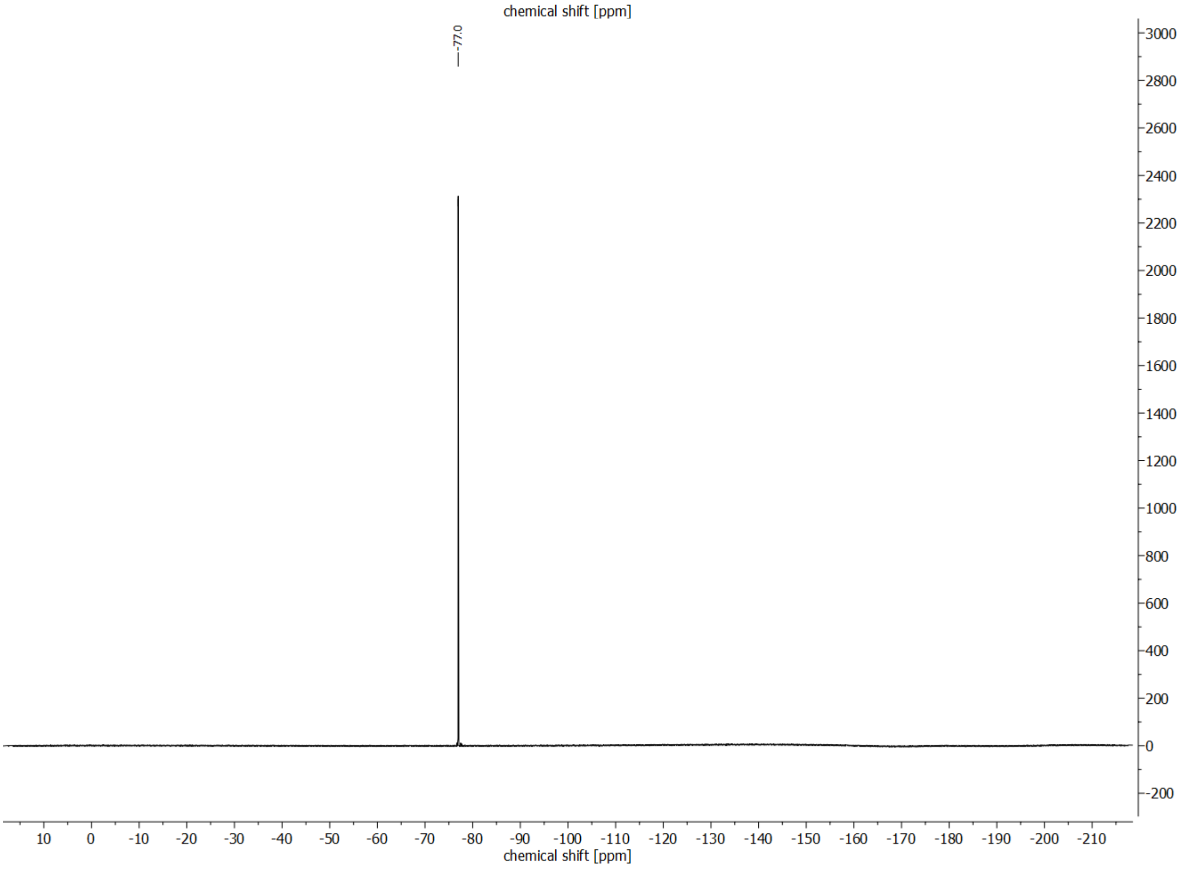
-77.0
chemical shift [ppm]

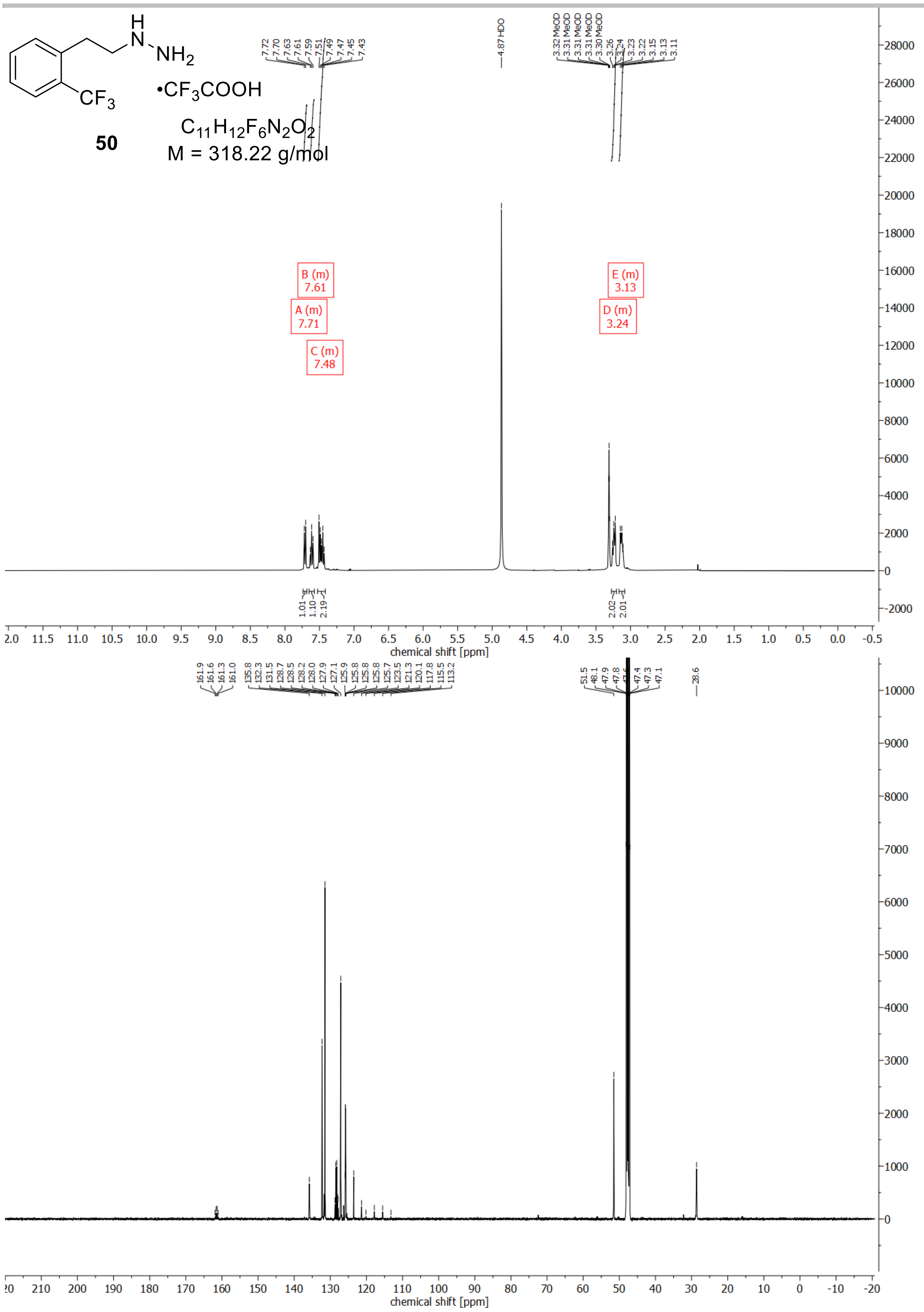
50
•CF3COOH
C11H12F6N2O2
M = 318.22 g/mol
4.87 HDO
B (m)
7.61
A (m)
7.71
C (m)
7.48
E (m)
3.13
D (m)
3.24
chemical shift [ppm]
chemical shift [ppm]

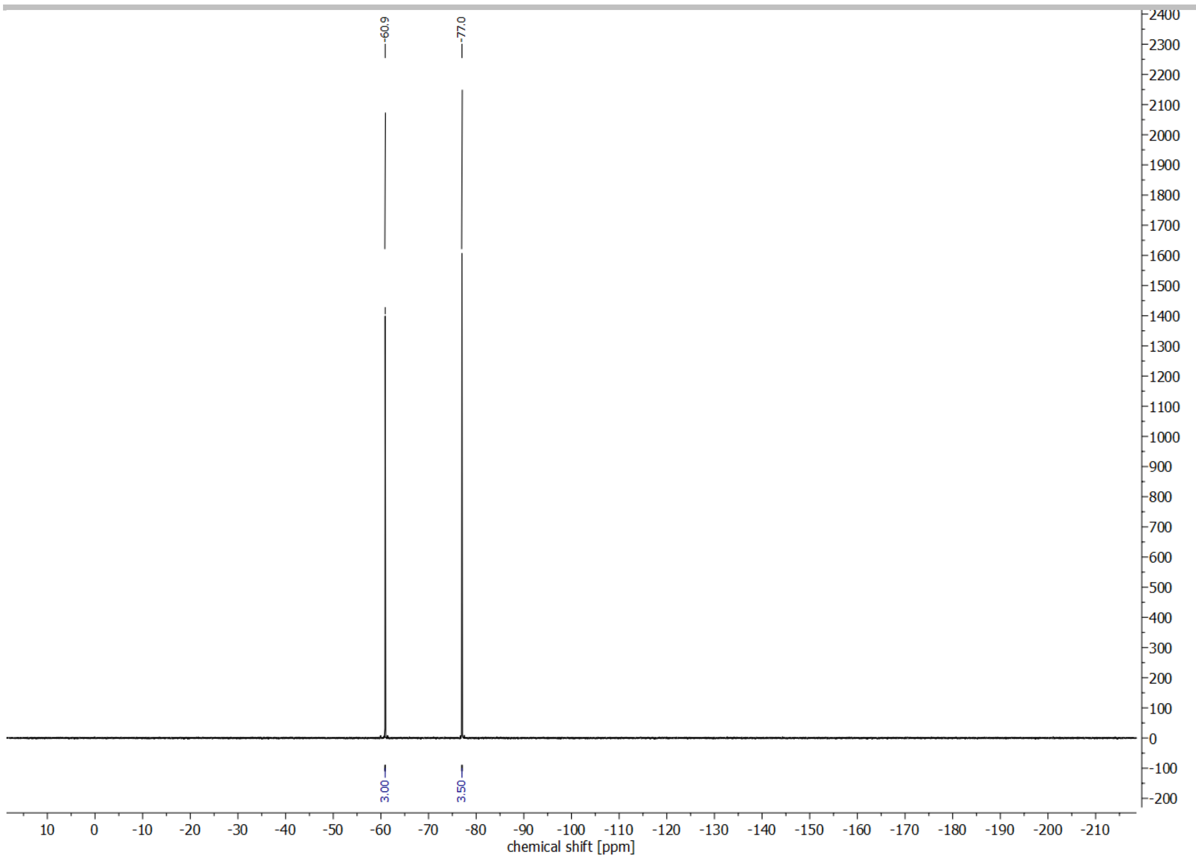
-60.9
-77.0
3.00
3.50
chemical shift [ppm]

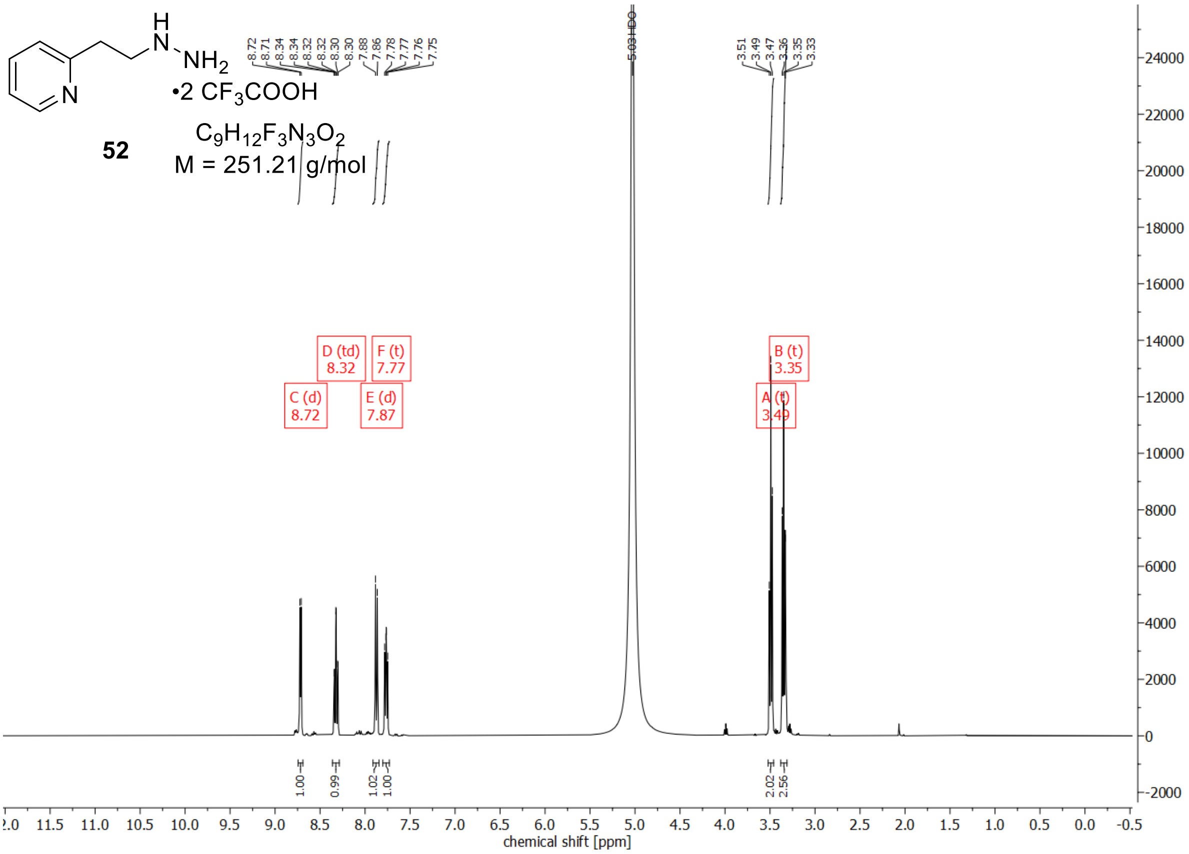
•2 CF_3COOH
52
$C_9H_{12}F_3N_3O_2$
M = 251.21 g/mol
D (td) 8.32
F (t) 7.77
C (d) 8.72
E (d) 7.87
B (t) 3.35
A (t) 3.49
chemical shift [ppm]

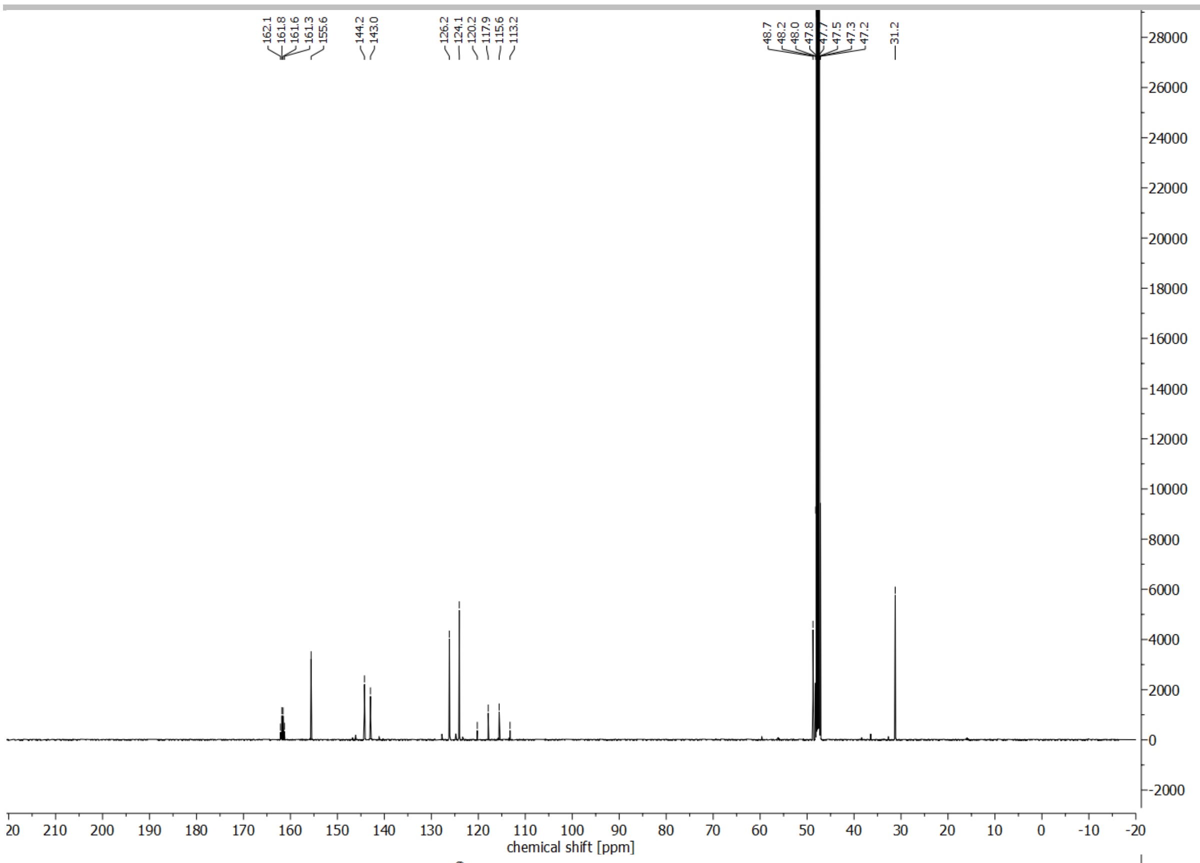
162.1
161.8
161.6
161.3
155.6
144.2
143.0
126.2
124.1
120.2
117.9
115.6
113.2
48.7
48.2
48.0
47.8
47.7
47.5
47.3
47.2
31.2
chemical shift [ppm]

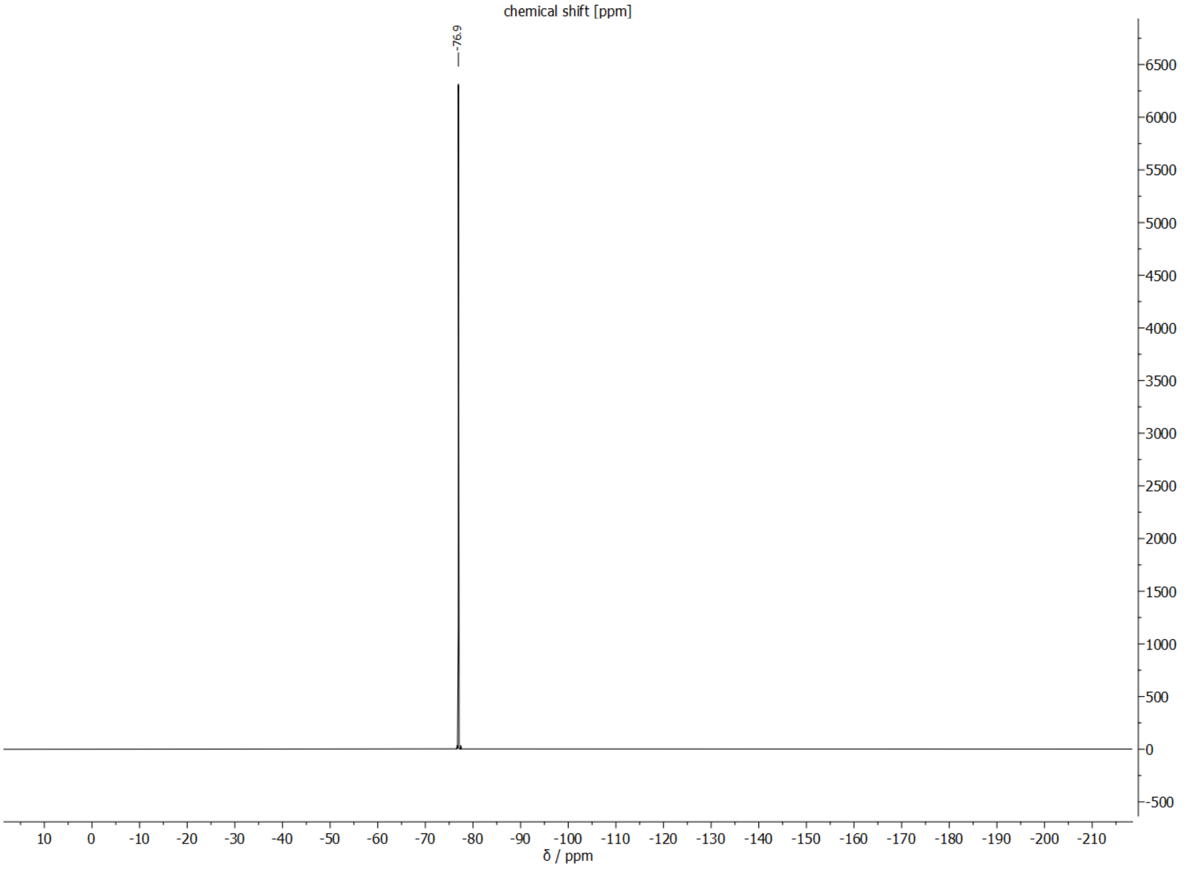
-76.9
δ / ppm

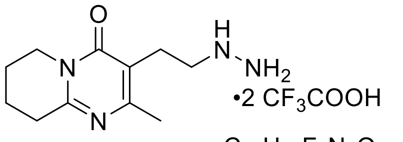

54

$C_{15}H_{20}F_6N_4O_5$
M = 450.34 g/mol

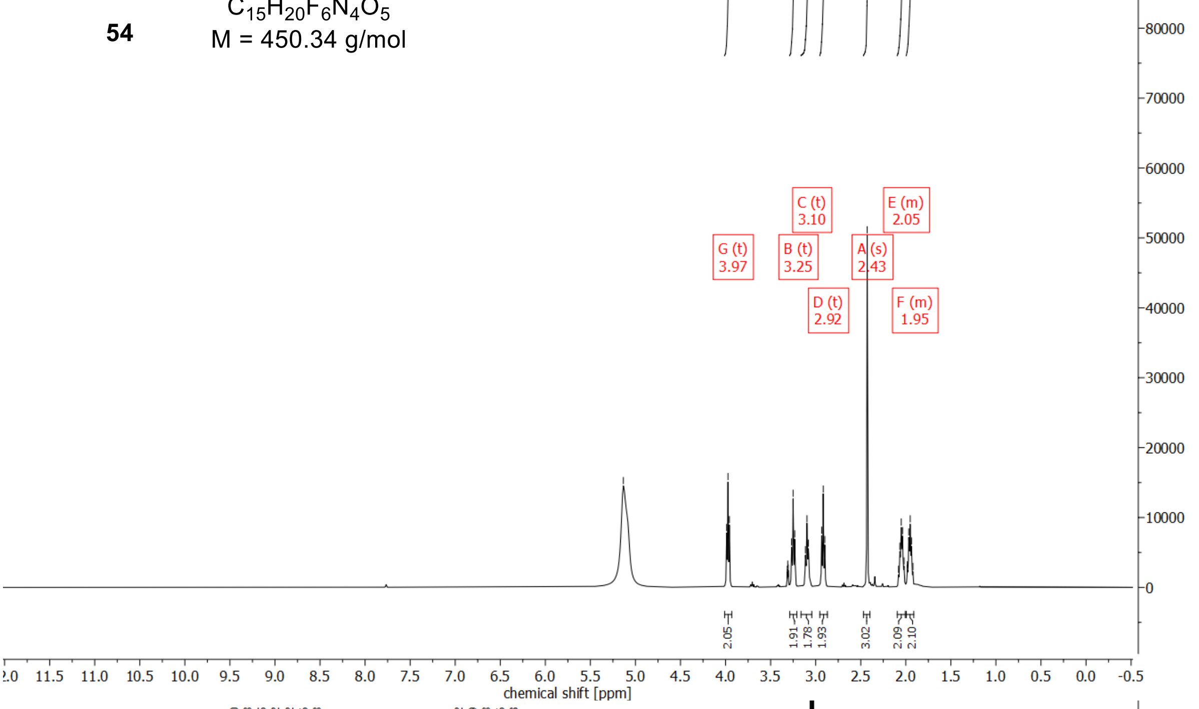

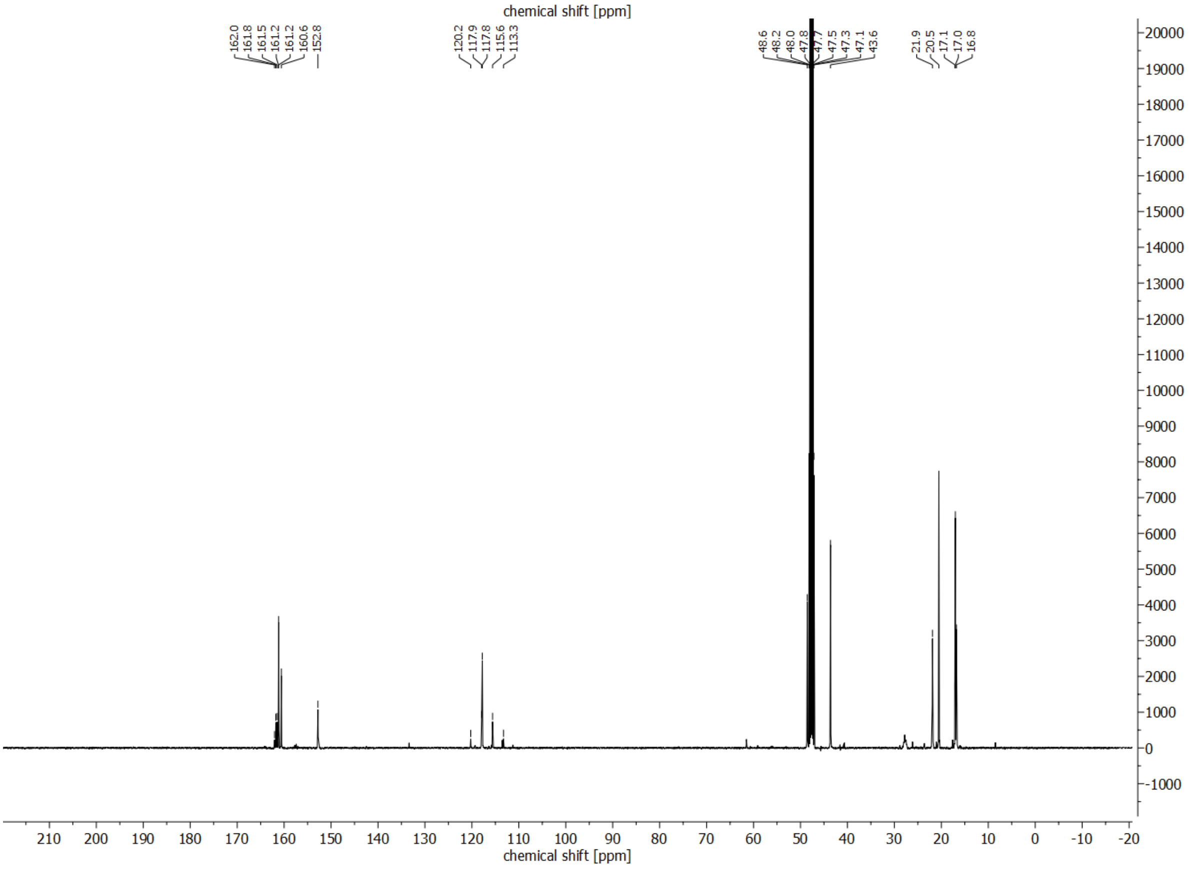

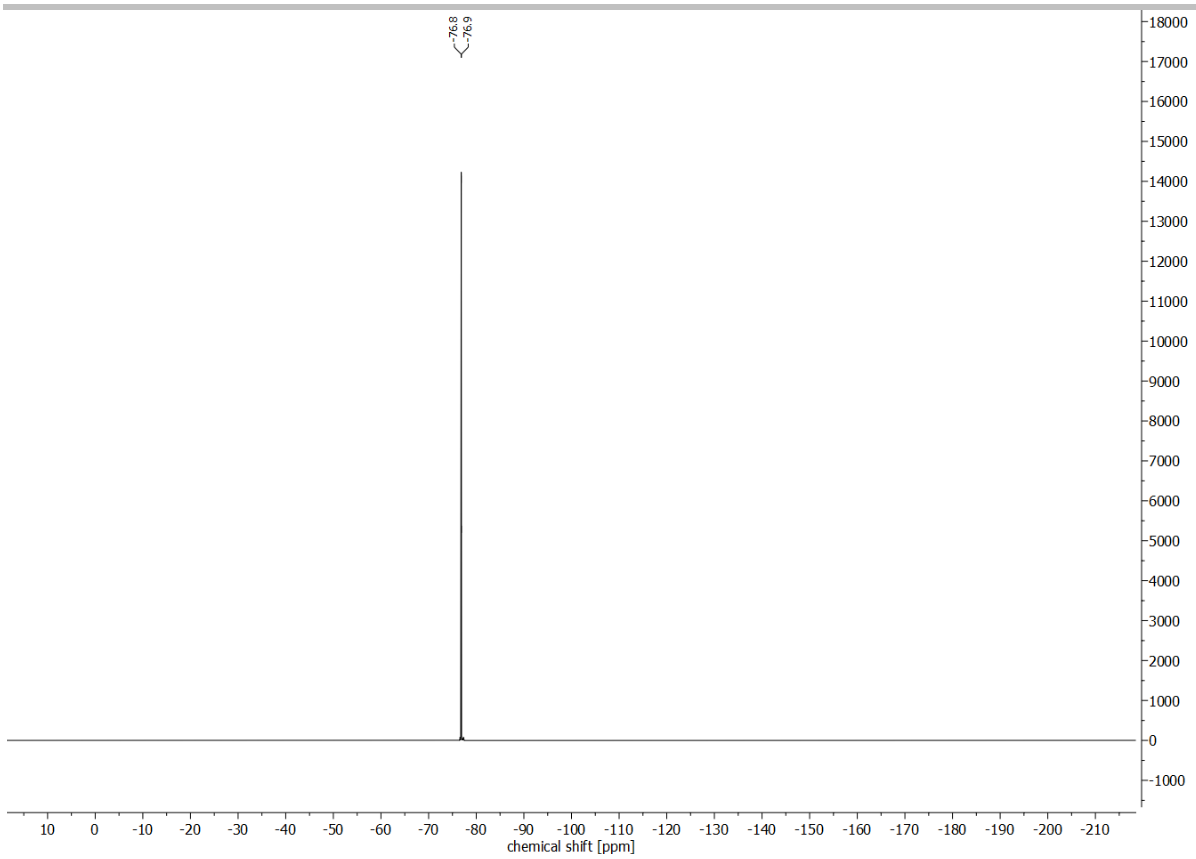
-76.8
-76.9
chemical shift [ppm]

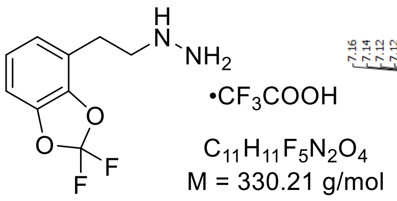
•CF3COOH
C11H11F5N2O4
M = 330.21 g/mol

56

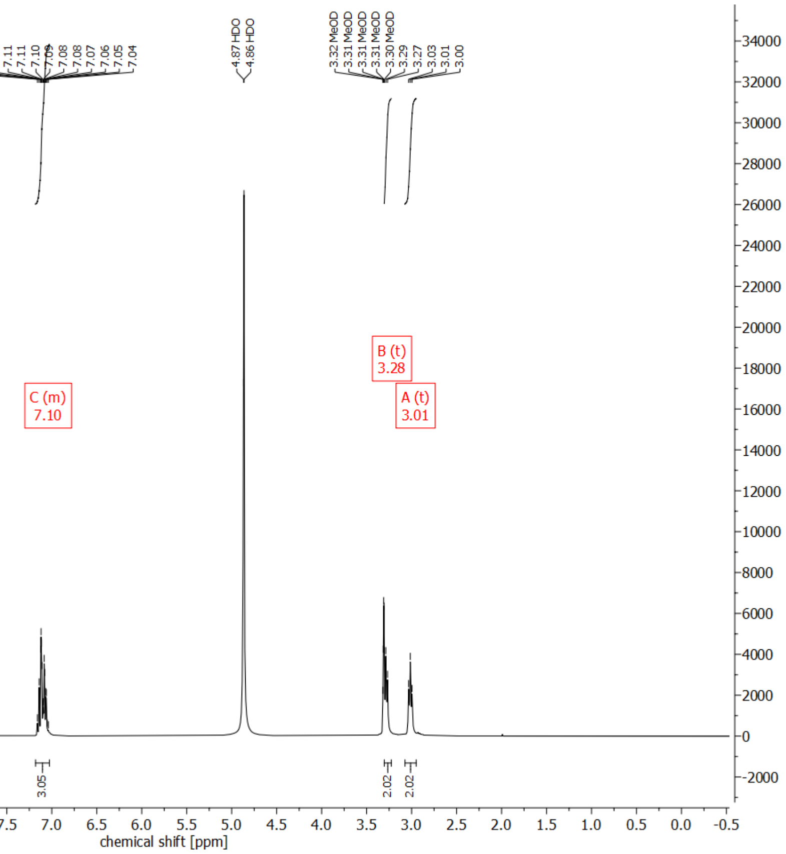
C (m)
7.10
B (t)
3.28
A (t)
3.01
4.87 HDO
4.86 HDO
3.05
2.02
2.02
chemical shift [ppm]

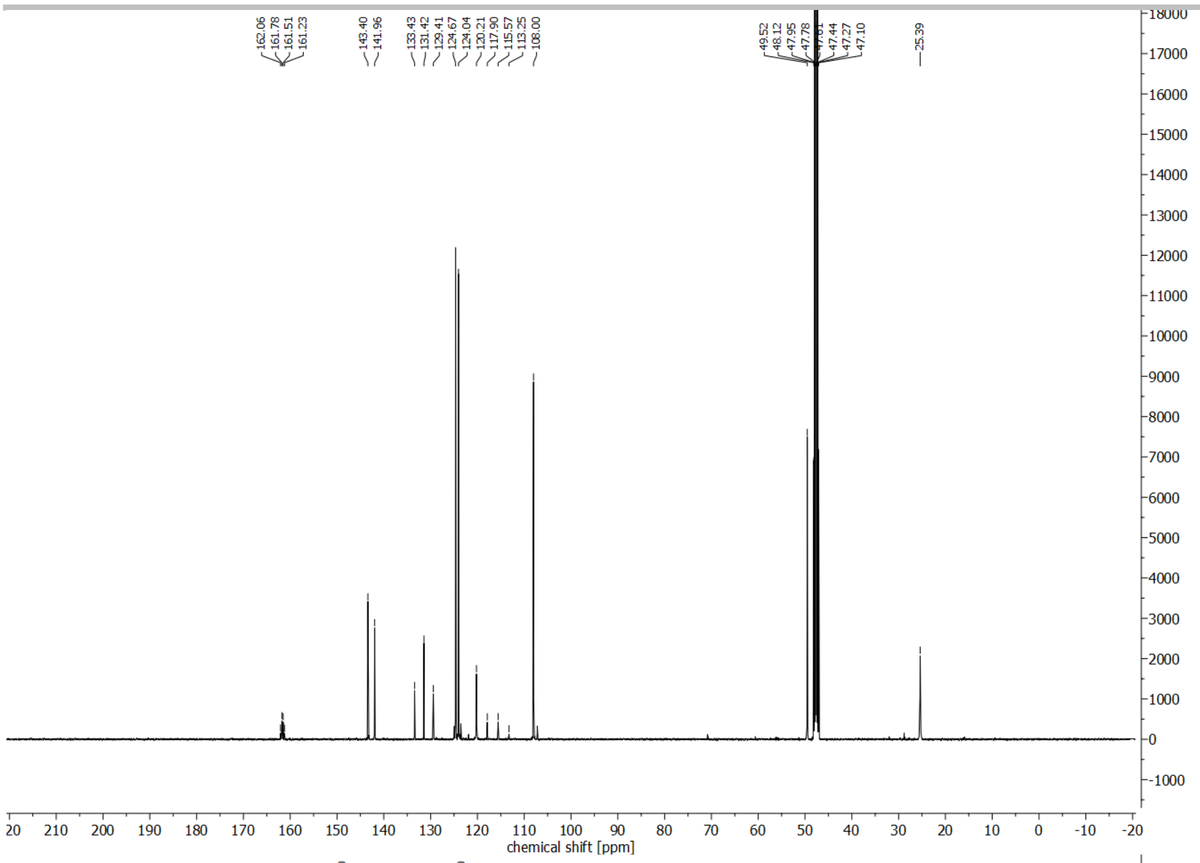
162.06
161.78
161.51
161.23
143.40
141.96
133.43
131.42
129.41
124.67
124.04
120.21
117.90
115.57
113.25
108.00
49.52
48.12
47.95
47.78
47.61
47.44
47.27
47.10
25.39
chemical shift [ppm]

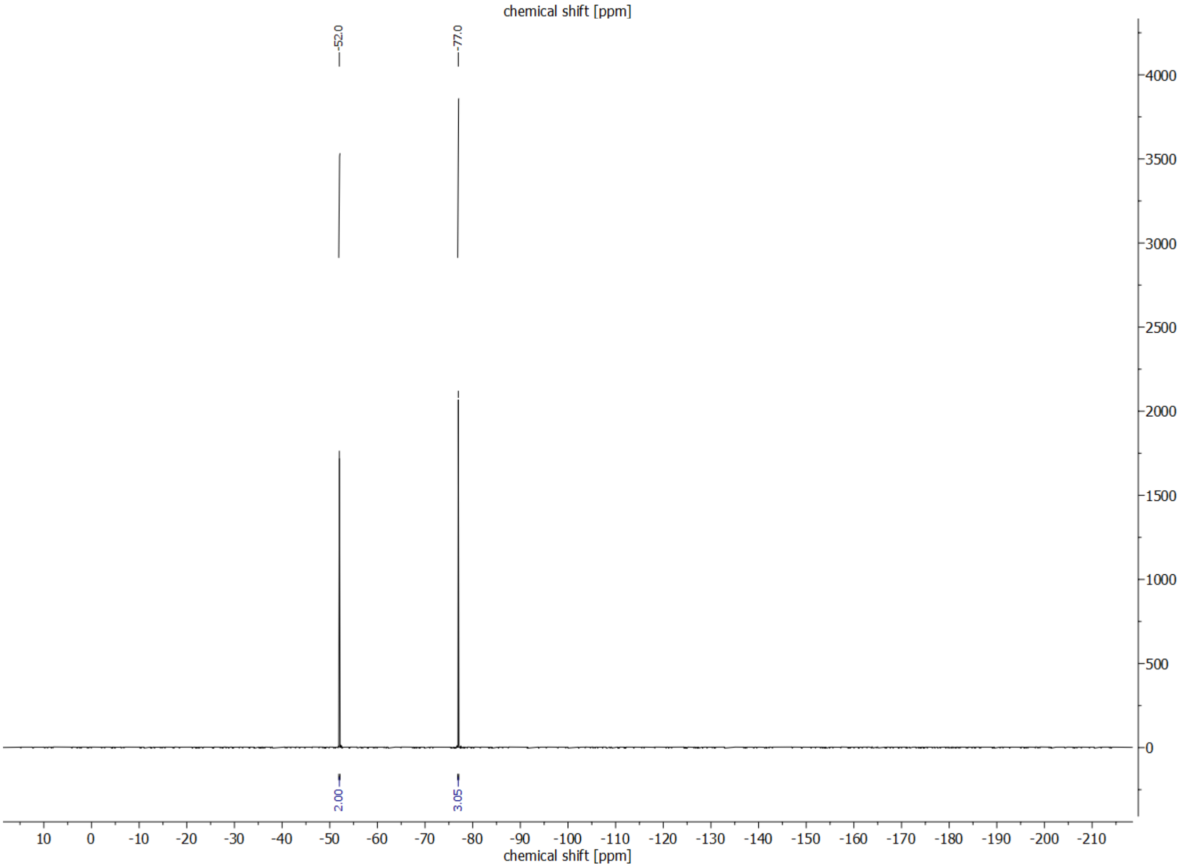
-52.0
-77.0
2.00
3.05
chemical shift [ppm]

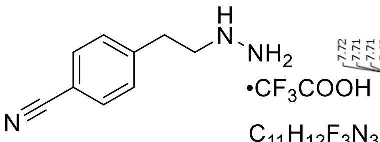

58

$C_{11}H_{12}F_3N_3O_2$
M = 275.23 g/mol

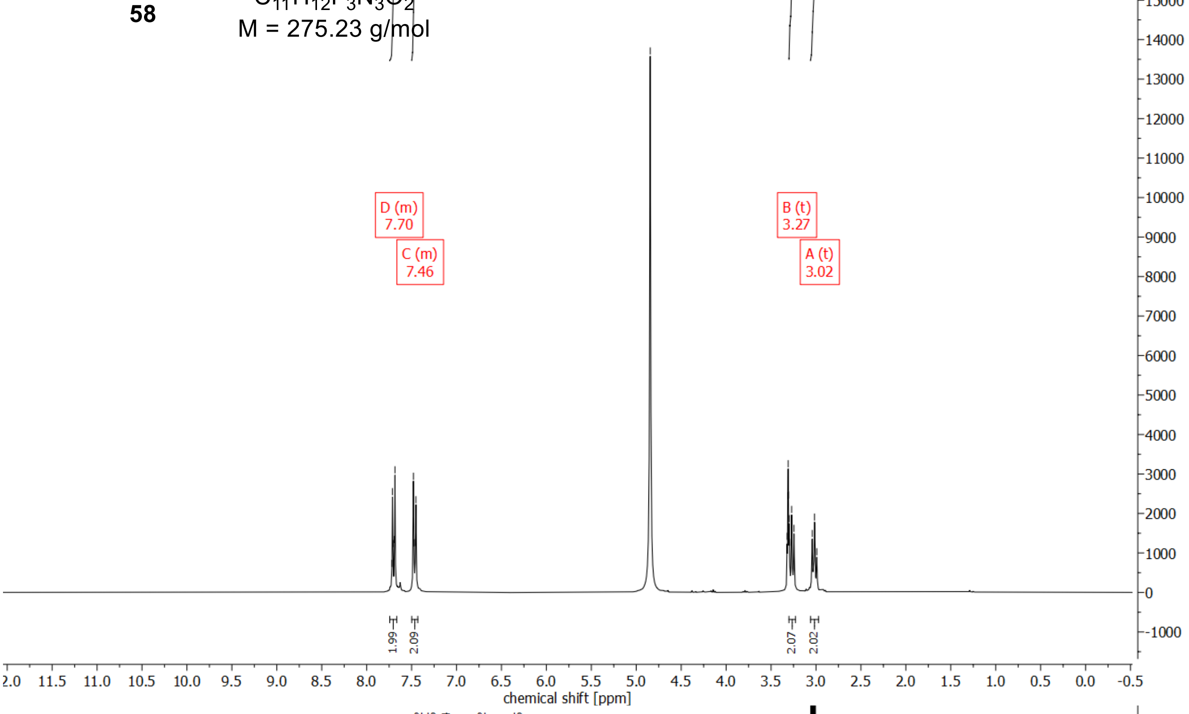

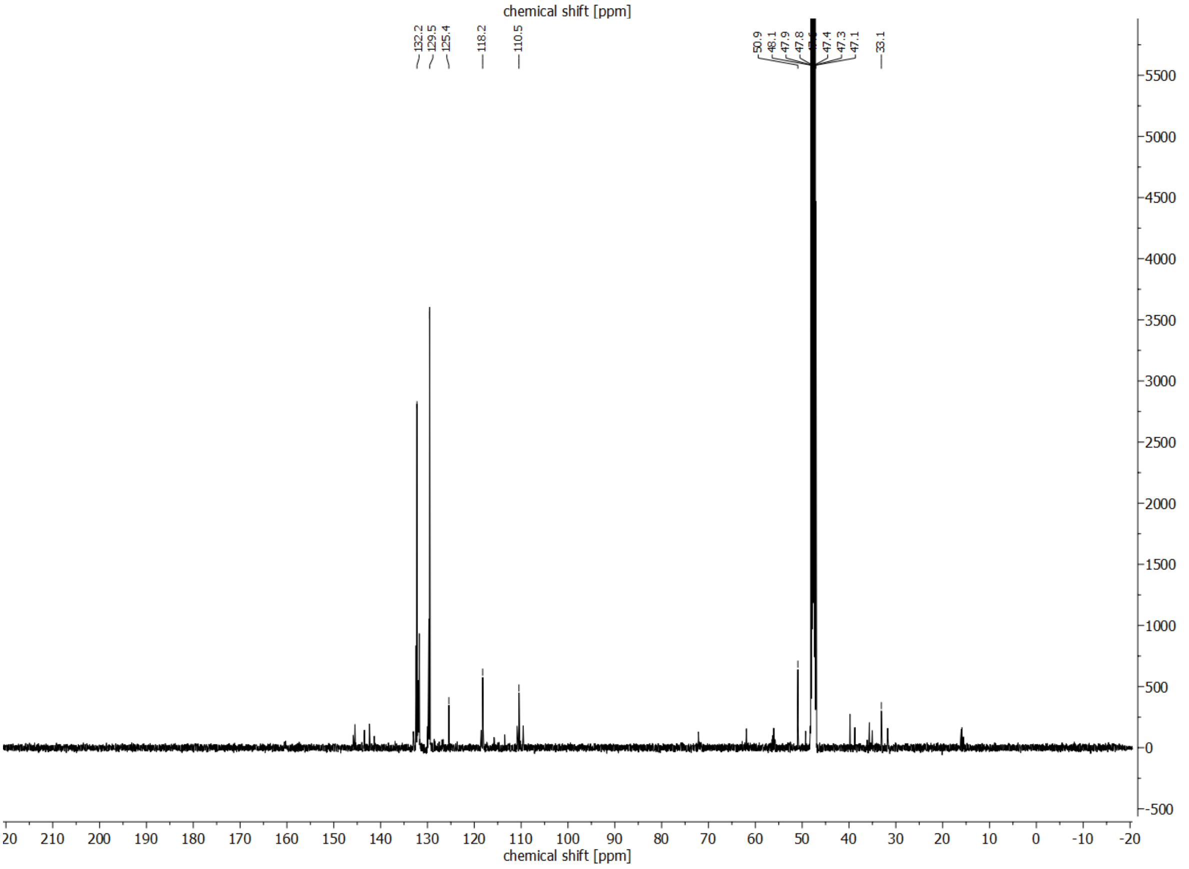

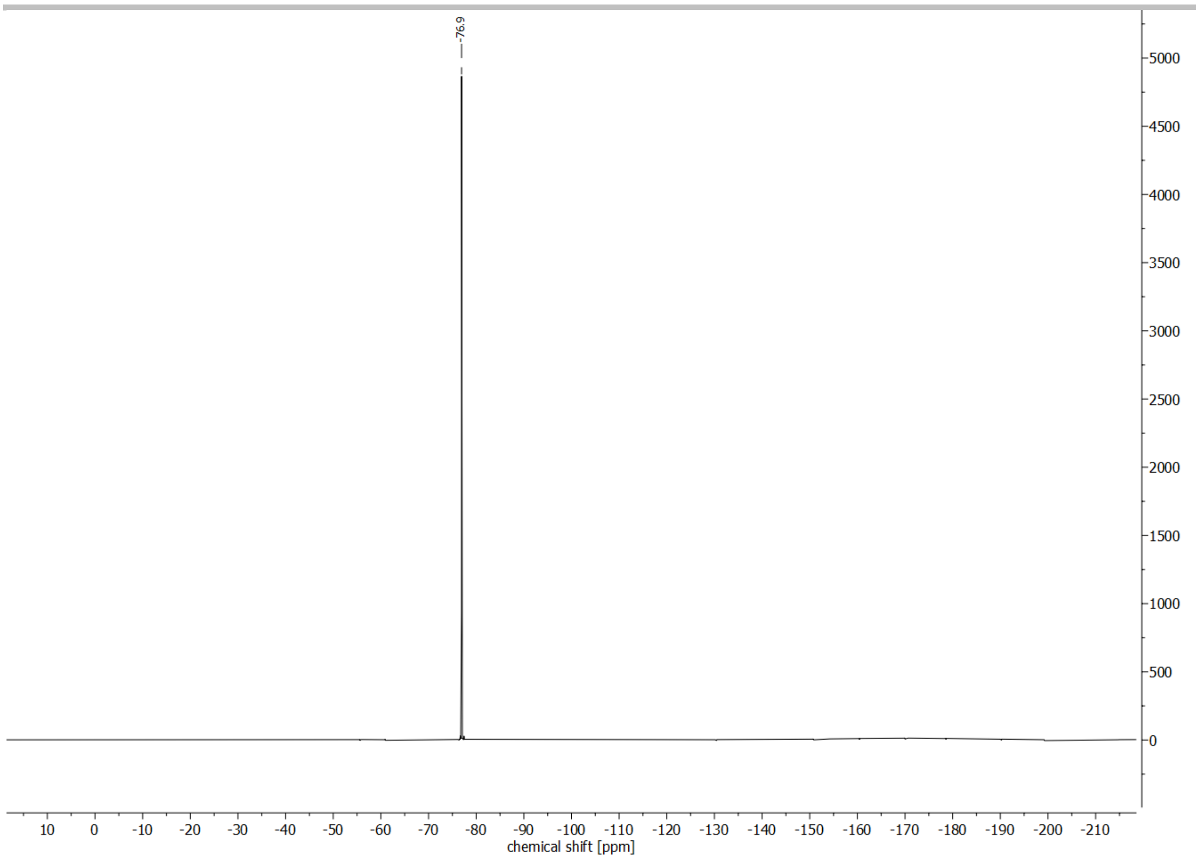

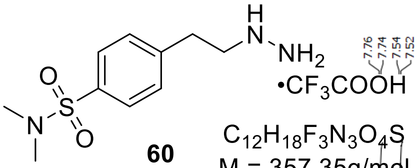

60

$C_{12}H_{18}F_3N_3O_4S$
M = 357.35g/mol

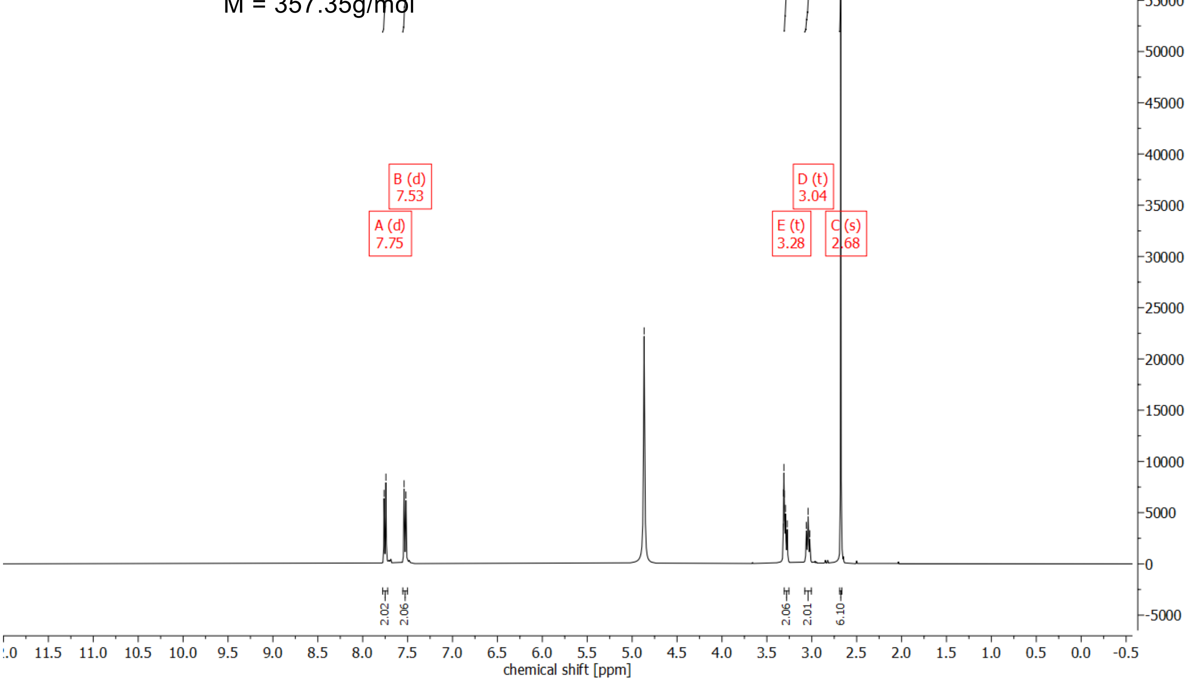

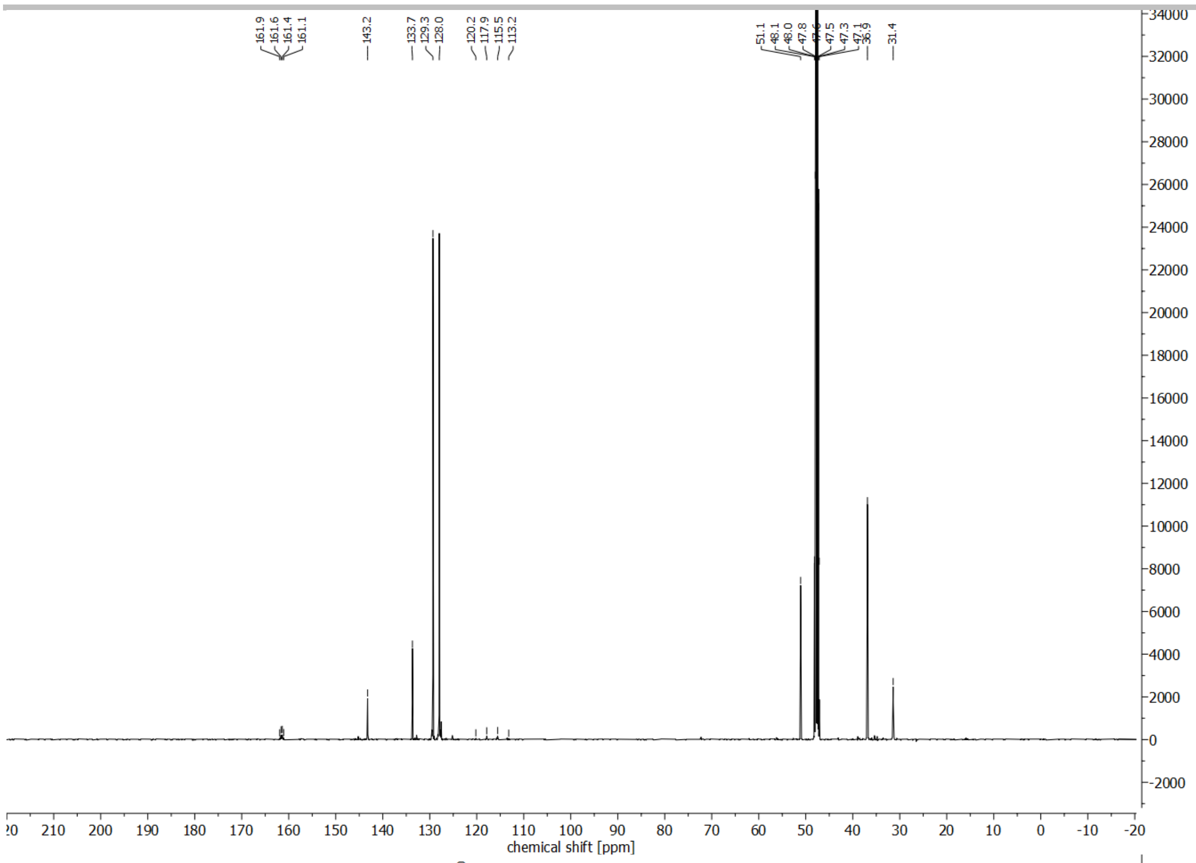
161.9
161.6
161.4
161.1
143.2
133.7
129.3
128.0
120.2
117.9
115.5
113.2
51.1
48.1
48.0
47.8
47.5
47.3
47.1
36.9
31.4
chemical shift [ppm]

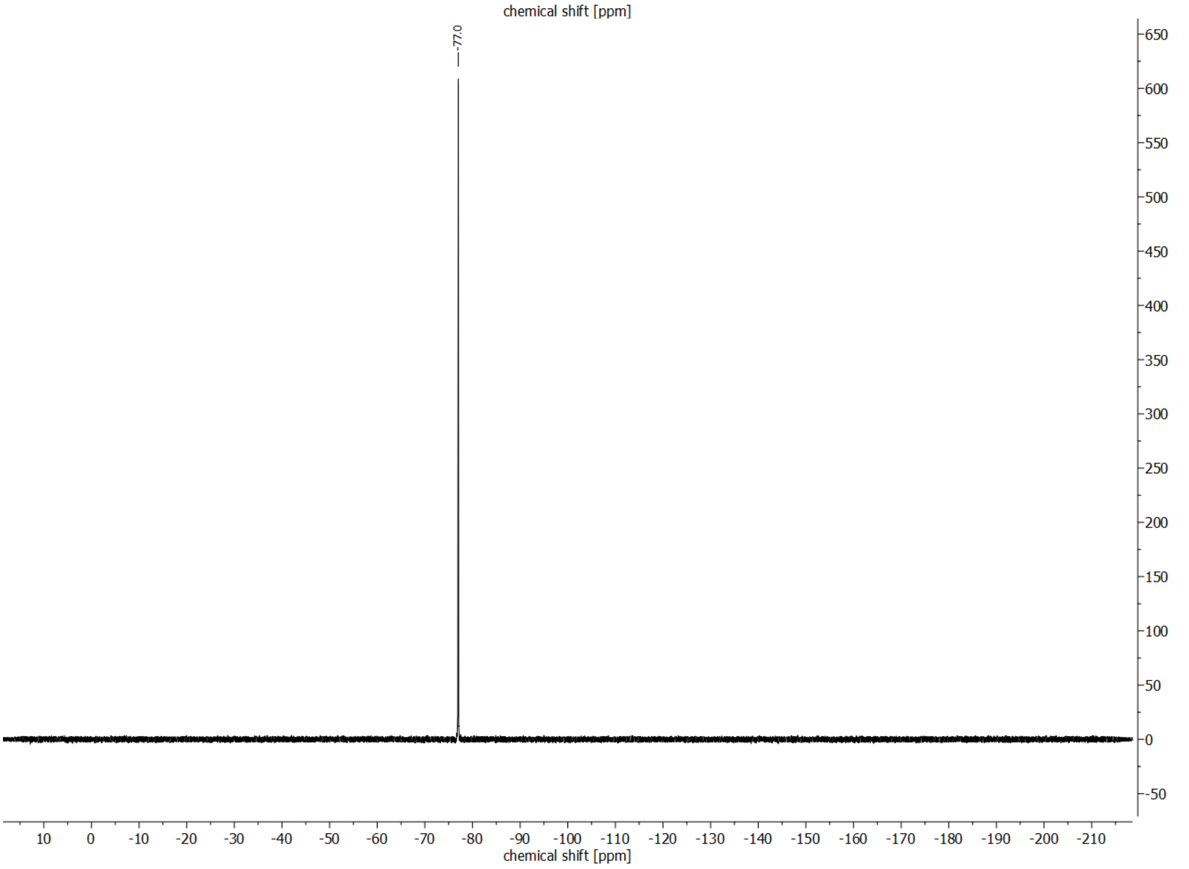
-77.0
chemical shift [ppm]

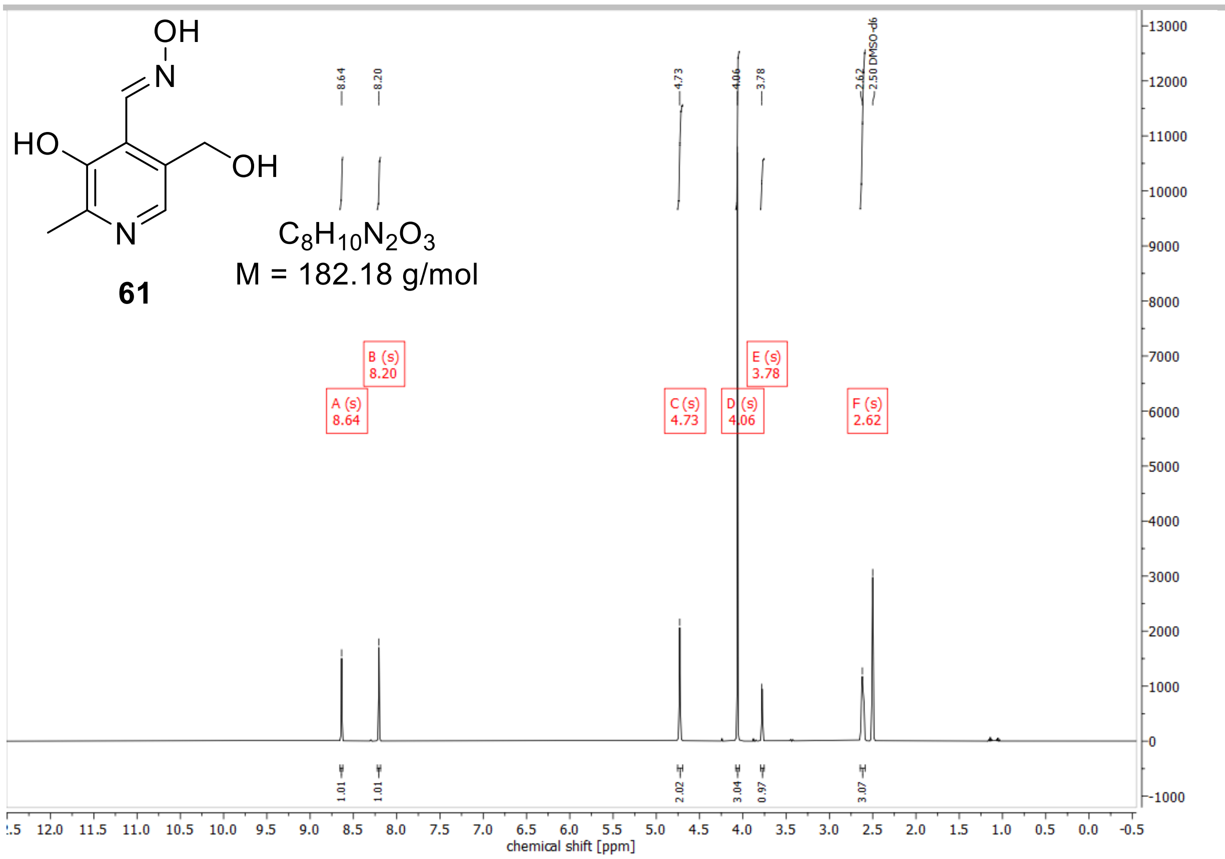

Author Contributions

M.P. designed, planned and conducted experiments including synthesis, proteomics sample preparation, as well as validation assays and analysis. M.P. performed proteomic and intact protein MS measurements and statistical analysis of the data. M.P., R.M.A. and M.S. cloned and expressed proteins. T.E.M. synthesized phenelzine derivatives and conducted competitive labelling experiments in *S. aureus*. S. A. S. devised the project and supervised experiments. M.P. and S. A. S. wrote the manuscript.
